# Expedient syntheses of *N*-heterocycles via intermolecular amphoteric diamination of allenes

**DOI:** 10.1038/s41467-018-03085-3

**Published:** 2018-02-19

**Authors:** Zhishi Ye, Sarju Adhikari, Yu Xia, Mingji Dai

**Affiliations:** 1 Department of Chemistry, Purdue University, West Lafayette, IN 47907 USA; 2 Center for Cancer Research and Institute for Drug Discovery, Purdue University, West Lafayette, IN 47907 USA; 3 Zhang Dayu School of Chemistry, Dalian University of Technology, Dalian, 116024 Liaoning China; 4 Department of Chemistry, Tsinghua University, 100084 Beijing, China

## Abstract

Saturated 1,4-diazo heterocycles including piperazines, 1,4-diazepanes, and 1,4-diazocanes, are highly important for therapeutic development, but their syntheses are often tedious. We describe here an amphoteric diamination strategy to unite readily available 1,2-, 1,3- or 1,4-diamine derivatives with electron-deficient allenes via a formal [*n* + 2] (*n* = 4, 5, 6) cyclization mode to produce the corresponding 1,4-diazo heterocycles in just one step. This strategy features mild reaction conditions, high functional group tolerance, and scalability (gram scale). The reagents used are cheap and readily available and no transition metal catalysts are needed. More sophisticated products containing trifluoromethyl group or bicyclic ring systems can be accessed via a one-pot procedure as well. Our mechanistic studies support that formation of mono-iodinated or chlorinated diamine intermediates is important for the desired transformation and the commonly proposed chloride-iodide exchange process and a radical N−C bond formation is unlikely when the combination of NCS/KI is used.

## Abstract (web summary)

Saturated *N*-heterocycle rings are common structural motifs of many FDA-approved drugs. Here, the authors show that a metal-free amphoteric diamination of allenes produces valuable six-, seven- and eight-membered *N*-heterocycles with high diversity and tolerance of functional groups on the ring.

## Introduction

More than half of the FDA-approved small-molecule therapeutics contain at least one *N*-heterocyclic ring. Among these *N*-heterocycles, saturated *N*-heterocycles are indispensable in drug discovery^1,2^. For example, piperidine and piperazine rank the first and third most common *N*-heterocycles appearing in small-molecule drugs, respectively. Over 60 FDA approved drugs contain a piperazine ring, several of which are within the top 100 best-selling pharmaceutical products. Structurally, piperazine features a six-membered ring with two nitrogen atoms in 1,4-relationship. This structural feature provides large polar surface area and relative structural rigidity. The piperazine moiety can often function as both hydrogen bond acceptor and donor and lead to improved potency, selectivity, water solubility, oral bioavailability, and ADME (absorption, distribution, metabolism, and excretion) properties^3^. Similarly, other 1,4-diazo heterocycles such as 1,4-diazepanes and 1,4-diazocanes are important structural motifs in bioactive molecules. Saturated 1,4-diazepane backbone has already emerged in several important FDA-approved small-molecule drugs^4–6^. However, most of the 1,4-diazo heterocycle-containing molecules, particularly piperazine-containing drugs, only have substitutions on one or both of the nitrogen atoms and the piperazine moiety is used as a linker to connect two portions for target binding or as an appendage to tune physicochemical properties. Only a small percentage of them have substitutions on the carbon atoms of the 1,4-diazo heterocycle and these substitutions are usually limited to methyl, aryl, or carboxylate groups. Therefore, there is lack of carbon-substitution diversity on these 1,4-diazo heterocycle scaffold in modern drug discovery and this is mainly due to the scarcity of available synthetic methods to efficiently provide carbon-substituted 1,4-diazo heterocycles with high regio-, stereo- and/or enantio-selectivity^7–9^.

So far, the most commonly used approaches to synthesize piperazines are the reduction of (di)ketopiperazines and stepwise *N*-alkylation of 1,2-diamines^10–13^. These methods generally use amino acids and 1,2-diamines as starting materials and require multiple steps. Therefore, the carbon-substitution pattern highly depends on the starting material availability. Recently, transition metal including Pd-^14–18^, Au-^19^, Zr-^20^, and Ir^21^-catalyzed amination cyclization methods have been developed to synthesize piperazines. These methods need to use precious and often air and moisture sensitive heavy metal catalysts and are very substrate dependent. Notably, a series of stannyl amine protocol (SnAP) reagents have been developed by Bode and co-workers to enable efficient and modular access of *N*-heterocycles including piperazines^22–25^. One limitation for the SnAP method is the involvement of toxic tin reagents which is partially solved by their development of a series of silicon amine protocol (SLAP) reagents^26^. Zhou and co-workers have developed an asymmetric reduction of pyrazine derivatives to provide substituted piperazine products in high enantioselectivity^27,28^. In this case, the pyrazine starting materials need to be pre-activated first. An appealing strategy to introduce substituents on piperazine is direct C−H functionalization^29^. Unfortunately, most of the available methods for α-C−H functionalization of amines including piperidines and pyrrolidines failed miserably on piperazine substrates due to the existence of the second nitrogen, which either creates various side reactions including elimination and dehydrogenation or diminishes the reactivity of the α-C−H bond and/or the catalyst used^30,31^. Progress has been made in direct α-C−H lithiation-trapping protocol of *N*-Boc-protected piperazines, but the reaction conditions are generally harsh and the results are highly substrate dependent^32^. Recently, pioneered by MacMillan and co-workers, direct α-C−H functionalization of piperazines via photoredox catalysis has emerged as a promising method^33–35^. In addition to transition metal catalysis, an interesting phosphine-catalyzed umpolung addition and intramolecular conjugative addition strategy has been developed by the Lu group to synthesize piperazines and 1,4-diazepanes. In this case, only symmetrical 1,2- or 1,3-diamine derivatives (tosyl sulfonamides) were explored^36^. In general, the syntheses of 1,4-diazepanes and 1,4-diazocanes are also very limited and far less studied than the piperazines^37–39^. 1,4-Diazocanes present an even higher level of synthetic challenge due to the existence of a strained eight-membered ring. Overall, there is a strong need for efficient and selective synthesis of substituted piperazines, 1,4-diazepanes and 1,4-diazocanes in order to unleash their full potential in novel therapeutic development.

**Fig. 1:**
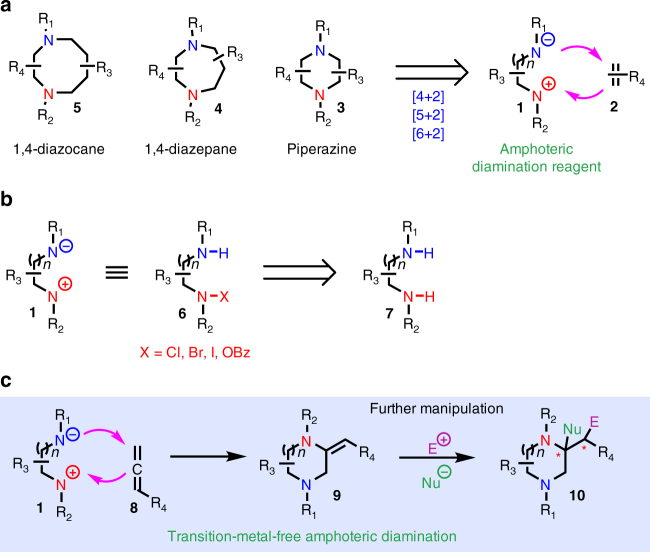
Amphoteric diamination cyclization. **a** A general design of an amphoteric diamination cyclization to an unsaturated π system. **b** Potential amphoteric diamination reagents and their syntheses. **c** This work: a transition-metal-free intermolecular amphoteric diamination of allenes to synthesize piperazines, 1,4-diazepanes, and 1,4-diazocanes

We wondered whether an amphoteric diamination reagent **1** would undergo cyclization with an unsaturated π-system **2** via a formal [*n* + 2] (*n* = 4, 5, 6) cyclization process to produce piperazine (**3**), 1,4-diazepane (**4**), and 1,4-diazocane (**5**) products in one step (Fig. 1). This amphoteric diamination strategy, once successfully developed, would provide rapid access to these privileged saturated *N*-heterocyclic structures and significantly enrich the toolbox of both medicinal chemists and synthetic chemists. In principle, 1,*n*-diamine (*n* = 2, 3, 4) derivative **6** could be viewed as an amphoteric reagent. In **6**, the two nitrogen atoms are electronically differentiated. Deprotonation of the blue nitrogen would generate the nucleophilic nitrogen species and the red halogen-amine (chloro-, bromo- or iodo-amine) or *O*-benzoyl hydroxylamine would function as the electrophilic part. Amphoteric reagent **6** could be synthesized or generated in situ readily from the 1,*n*-diamine (*n* = 2, 3, 4) **7** via selective halogenation or oxidation of the red nitrogen. Herein, we report our initial discovery in developing a transition-metal-free intermolecular amphoteric diamination of allenes to synthesize a wide range of carbon-substituted piperazines, 1,4-diazepanes, and 1,4-diazocanes in one step. This enabling method features mild reaction conditions and cheap and readily available reagents. The primary cyclization product **9** can be converted to more sophisticated product by exploiting the rich chemistry of its enamine moiety.

## Results

### Optimization of reaction conditions

Our investigation of the amphoteric diamination of allenes (Fig. 1c) started with chloroamine sulfonamide **11a** (Table 1) which was synthesized from selective chlorination of the more electron-rich alkylamine of **11** in presence of a more electron deficient sulfonamide. Chloroamine sulfonamide **11a** can be purified on silica gel column and stored in refrigerator for several months. Since copper catalyst has often been used in catalyzing electrophilic amination reactions^40,41^, we first treated a mixture of chloroamine sulfonamide **11a** and allenyl ester **12** (1.5 equiv.) with 10% CuI and Cs_2_CO_3_ (1.5 equiv.) in THF at room temperature (Table 1, entry 1). To our delight, cyclized vinylogous amide product **13** was obtained in 20% yield surprisingly as a single regioisomer. Since vinylogous amide **13** is not stable on silica gel, a one-pot reduction was conducted to convert it to piperazine **14**. No product **13** was observed without CuI (Table 1, entry 2), which indicates the importance of CuI. With this encouraging result, we investigated various copper catalysts, ligands, and other reaction parameters, but failed to improve the reaction yield. We then switched CuI to KI (0.2 equiv.) and desired product **13** was obtained in 35% yield (Table 1, entry 3). It turned out that iodide was catalyzing this transformation, not the copper part. Once we realized this key point, we quickly optimized the reaction conditions by simply increasing the amount of KI (Table 1, entries 4–6) and product **13** was produced in 79% yield with either 1.0 or 2.0 equiv. of KI. Other iodide salts such as NaI and tetrabutylammonium iodide (TBAI) are less effective than KI (Table 1, entries 8, 9). THF was approved to be superior to CH_3_CN (Table 1, entry 7) and other solvents and increasing the reaction temperature to 40 °C did not improve the reaction yield (Table 1, entry 10). Since chloroamine sulfonamide **11a** was produced simply by chlorination of **11** with *N*-Chlorosuccinimide (NCS), we explored the possibility of in situ generation of **11a** (Table 1, entry 11). This approach was effective to produce **13** in 79% yield. We speculated that the role of KI was presumably to generate a more reactive iodoamine via an iodide-chloride exchange^42^, which turned out not to be the case as we carried on this research further. Therefore, we replaced the combination of NCS/KI with *N*-Iodosuccinimide (NIS) (Table 1, entry 12). Satisfyingly, product **13** was obtained in 86% yield and a one-pot reduction gave piperazine **14** in 70% yield. Notably, this one-pot procedure can be scaled up to produce **14** in 76% yield at gram scale (Table 1, entry 13).

**Table 1:** Optimization of reaction conditions

			
Entry	X	Reaction conditions (equiv.)	Yield of **13**^a^ (yield of **14**^b^)
1	Cl	CuI (0.1), Cs_2_CO_3_ (1.5), THF, RT, 24 h	20%
2	Cl	Cs_2_CO_3_ (1.5), THF, RT, 24 h	0%
3	Cl	KI (0.2), Cs_2_CO_3_ (1.5), THF, RT, 24 h	35%
4	Cl	KI (0.5), Cs_2_CO_3_ (1.5), THF, RT, 24 h	75%
5	Cl	KI (1.0), Cs_2_CO_3_ (1.5), THF, RT, 24 h	79% (65%)
6	Cl	KI (2.0), Cs_2_CO_3_ (1.5), THF, RT, 24 h	79%
7	Cl	KI (1.0), Cs_2_CO_3_ (1.5), MeCN, RT, 24 h	63%
8	Cl	NaI (1.0), Cs_2_CO_3_ (1.5), THF, RT, 24 h	54%
9	Cl	TBAI (1.0), Cs_2_CO_3_ (1.5), THF, RT, 24 h	trace
10	Cl	KI (1.0), Cs_2_CO_3_ (1.5), THF, 40 °C, 24 h	78%
11	H	NCS (1.05), THF, 1 h, then **12**, KI (1.0), Cs_2_CO_3_ (1.5), RT, 24 h	79%
12	H	NIS (1.05), THF, 1 h, then **12**, Cs_2_CO_3_ (1.5), RT, 24 h	86% (70%)
13	H	as entry 12, but gram scale	--% (76%)

^a^ NMR yield of **13**

^b^ Isolated yield of **14**

### Substrate scope for the synthesis of piperazines

With the optimized reaction conditions in hand, the scope study of both the 1,2-diamines and allenes was subsequently conducted (Table 2). The one-pot amphoteric diamination procedure was approved to be very general in preparing various carbon-substituted piperazine products. Different 1,2-diamine derivatives underwent cyclization with allenyl ester **12** to give the desired piperazine products (**19**–**57**) which can also be viewed as β,γ-amino acid derivatives. Functional groups including bromide (**28**, **29**), CF_3_ (**21**), NO_2_ (**23**), free alcohol (**39**), Boc-carbamate (**40**), olefin (**44**), cyclopropyl group (**41**), and heteroaromatics (**30**–**35**, **45**, **51**, **58**) such as quinonine, pyridine, furan, pyrrole, indole, and thiophene are all well tolerated under the mild reaction conditions. The tosyl group can be switched to other sulfonamide groups (**45**–**52**) including the readily removable nosyl group (**48**–**50**). In addition to other allenyl esters (**53**–**57**), allenyl ketone (**58**–**64**), nitrile (**65**), sulfone (**66**), phosphine oxide (**67**), and phosphanate (**68**) can be used as the unsaturated π-system for the amphoteric diamination cyclization to afford piperazines with a diverse substituents on C2. The introduction of sulfone, phosphine oxide, and phosphanate into the piperazine products offers opportunities for further structural diversifications via various olefination reactions.

**Table 2:** Substrate scope for piperazine synthesis

	

Piperazines with more than one carbon-substitutions can be produced as well (**69**–**81**). Notably, for the case of 2,6-disubstituted piperazines, after the one-pot NaBH_3_CN reduction, instead of getting the 2,6-*cis* products with two equatorial substituents, which are often produced as the predominant products especially with nitrogen-unprotected cases^43^, the more challenging 2,6-*trans* substituted products were obtained as the major products (**69**, **70**; dr > 15:1). The *trans*-selectivity observed in the above reduction is presumably due to an axial hydride attack on the iminium ion intermediate formed under acidic conditions. Iminium ion conformer **70a** is favored over **70b** because the latter suffers from strong steric interaction between the *C*-Me and *N*-Me groups (A^1,2^ type interaction)^44^. An axial hydride attack on **70a** delivers the 2,6-*trans* substituted product as the major stereoisomer. For the case of 2,5-substituted piperazines, instead of getting the 2,5-*trans* substitution patterns where the two substituents are equatorially oriented, 2,5-*cis* substituted products were obtained in our cases (**71**–**76**, **79**, **80**). Based on the crystal structures of **71** and **75**′, an intermediate we isolated before NaBH_3_CN reduction (the corresponding primary amphoteric cyclization product isomerized to **75**′ on silica gel column), we hypothesized that the phenyl group in iminium ion intermediate **75a** prefers to occupy the axial position to avoid strong steric repulsion with the tosyl group and an axial delivery of the hydride provides the 2,5-*cis* product as the dominant stereoisomer. These results enable ready access of 2,6-*trans* or 2,5-*cis* substituted piperazines, which are otherwise challenging to synthesize. Interestingly, for the cases of bicyclic piperazine products **77**, **78** and **81**, the major products (dr > 15:1) turned out to have a 2,6-*cis* and/or 2,5-*trans* stereochemical relationship. In these cases, the bicyclic ring systems locked the conformation and an axial hydride attack gave the major product. The relative stereochemistry of **70**, **71**, **76**, **77**, and **81** were unambiguously established by X-ray crystallography of themselves (**70**, **71**, **77**) or their derivatives (**76**, **81**, see the Supplementary Figs. 1 and 2).

### Scope for the synthesis of 1,4-diazepanes and 1,4-diazocanes

We then explored the possibility of expanding the amphoteric diamination cyclization strategy to synthesize 1,4-diazepanes and 1,4-diazocanes with readily available 1,3- or 1,4-diamine derivatives (Table 3). With the NIS reaction conditions at an elevated reaction temperature (60 °C, Conditions **A**), 1,4-diazepane products **86**–**102** were produced smoothly in modest to good yield. For the products containing an all-carbon quaternary center (**103**–**106**), the combination of NCS (1.05 equiv.) and KI (2.0 equiv.) at 60 °C (Conditions **C**) is superior to the NIS conditions. For the case of 1,4-diazocane synthesis, a highly strained eight-membered ring needs to be constructed and it has been a long standing synthetic challenge. It turned out that the amphoteric diamination cyclization strategy is highly efficient for this task. Simply heating the mixture of mono-*N*-iodinated 1,4-diamines generated in situ with NIS treatment and the corresponding allene substrates at 100 °C followed by one-pot reduction, desired 1,4-diazocanes were produced in modest to good yield (**107**–**116**). For the syntheses of 1,4-diazepanes and 1,4-diazocanes, like the case of piperazine synthesis, allenyl ester (**86**–**97**, **103**–**107**, **112**–**115**), ketone (**100**), nitrile (**98**, **116**), sulfone (**102**, **108**–**111**), phosphine oxide (**99**), and phosphanate (**101**) are all effective substrates. Despite the elevated reaction temperatures, a broad range of functional groups are tolerated. The necessity of an elevated reaction temperature is presumably due to the difficulties involved in closing the seven- or eight-membered ring systems. The amphoteric iodoamine intermediates derived from 1,4-diamines are believed to have lower reactivity in comparison to the ones derived from 1,2- and 1,3-diamines presumably due to the weakened or lack of intramolecular hydrogen bonding interaction between the sulfonamide hydrogen atom and the iodoamine nitrogen atom.

**Table 3:** Substrate scope for the synthesis of 1,4-diazepanes and 1,4-diazocanes

	

### Diversification of the vinylogous amide products

**Fig. 2:**
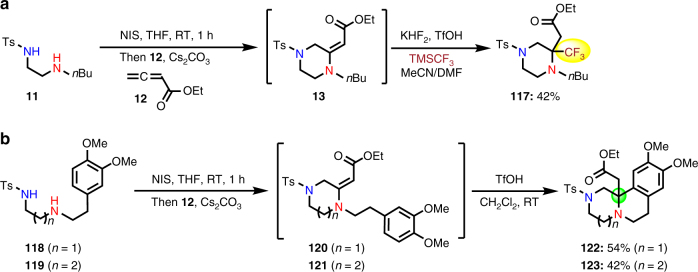
Diversification of the vinylogous amide products. **a** Synthesis of α-CF_3_-substituted piperazine **117** via a one-pot trap of the vinylogous amide intermediate **13** with TMSCF_3_. **b** Synthesis of bicyclic piperazine products via an intramolecular Mannich-type reaction

Instead of directly reducing the vinylogous amide products generated from the amphoteric diamination cyclization of allenes, we further explored the possibility of harnessing their intrinsic reactivity to introduce other substituents and/or to increase product structural complexity. Due to the importance of trifluoromethyl (CF_3_) group in medicinal chemistry and the current synthetic challenges of installing CF_3_ at the α-position of piperazines^45^, we started to develop a protocol to synthesize α-CF_3_-substituted piperazines (Fig. 2a). After the formation of the crude primary vinylogous amide product **13**, trifluoromethanesulfonic acid (TfOH) was used to isomerize the vinylogous amide to an iminium intermediate, which was then trapped by a nucleophilic addition of trifluoromethyl anion derived from a combination of KHF_2_ and the Ruppert−Prakash reagent TMSCF_3_. Desired product **117** was obtained in 42% yield from **11**. On the other hand, the vinylogous amide products **120** or **121** synthesized from the amphoteric diamination cyclization of **118** or **119** with allene **12** can be readily converted to a 6,6- or 6,7-fused bicyclic product (**122** or **123**) in 54 or 42% yield, respectively, via an intramolecular Mannich-type reaction (Fig. 2b). These two diversification reactions demonstrate the synthetic potential of the amphoteric diamination cyclization of allenes to synthesize complex *N*-heterocycles and highlight the advantages of using allene substrates.

### Removal of tosyl and nosyl groups

**Fig. 3:**
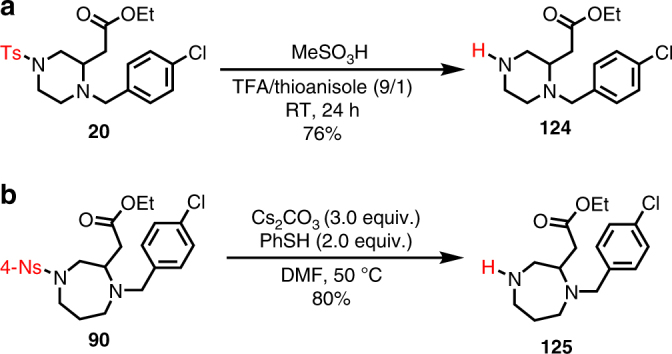
Removal of the tosyl and nosyl groups. **a** Deprotection of tosyl (Ts) group with MeSO_3_H in a mixture of TFA and thioanisole at room temperature. **b** Deprotection of nosyl (4-Ns) group with PhSH and Cs_2_CO_3_ in DMF at 50 °C

While sulfonamides prevalently exist in many drug molecules and other biofunctional molecules, it would significantly enhance the impact of the amphoteric diamination cyclization strategy in medicinal chemistry if the sulfonamide groups such as tosyl and nosyl groups can be removed easily to allow further functionalization of these privileged *N*-heterocycles. We have successfully demonstrated that both the tosyl and nosyl groups can be removed readily (Fig. 3). For example, upon treatment of piperazine **20** with MeSO_3_H in a mixture of trifluoroacetic acid (TFA) and thioanisole, desired product **124** was obtained in 76% yield^46^. The nosyl group of 1,4-diazepane **90** can be removed under basic conditions (Cs_2_CO_3_) with PhSH as nucleophile and product **125** was obtained in 80% yield^47^. The ease of removing both the tosyl and nosyl group enables the introduction of other functionalities on the nitrogen atoms, which carries important value in applying this method in medicinal chemistry.

## Discussion

**Fig. 4:**
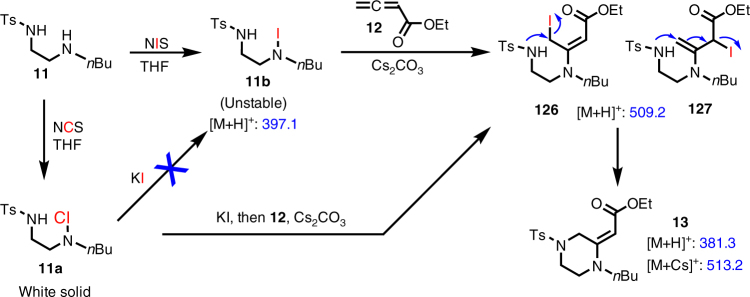
ESI-MS study of the reaction process. Both the NIS and NCS/KI procedures were monitored. The blue numbers are observed ion signals (*m*/*z*). MS analysis was performed by nanoelectrospray ionization-mass spectrometry (nanoESI-MS), using a 4000 QTRAP mass spectrometer, equipped with a home-built nanoESI source. NanoESI tips (~10 µm o.d.) were pulled from borosilicate glass capillary tips (1.5 mm o.d. and 0.86 mm i.d.) using a micropipette puller

To provide insights about the reaction mechanism of this amphoteric diamination cyclization, we first used ESI-MS to monitor the reaction process. The reaction between **11** and **12** was studied under both the NIS and NCS/KI reaction conditions (Fig. 4 and Supplementary Figs. 3−6). Upon the treatment of **11** with NIS, we observed the formation of iodoamine **11b** ([M + H]^+^: 397.1), but it cannot be isolated due to its instability under light and heat and on silica gel column. After introduction of allene **12** and Cs_2_CO_3_ to **11b**, the formation of an iodide intermediate with molecular weight of 508.1 was observed. This iodide intermediate was tentatively assigned as **126** or **127**, which cannot be differentiated by the ESI MS data. We also tried to isolate these two proposed allylic iodide intermediates, but these attempts were unsuccessful due to their high reactivity despite their clear existence in the ESI-MS systems. Longer reaction time led to the formation of **13**, observed at 381.3 ([M+H]^+^) or 513.2 [M+Cs]^+^, via a substitution process to form the second carbon−nitrogen bond and closed the six-membered ring. An aziridinium ion intermediate is unlikely to be involved in the ring closing process, but cannot be ruled out completely at this stage. Interestingly, when *N*-chloroamine **11a**, which is bench stable and can be isolated as a white solid, was treated with KI, we did not observe the formation of **11b** via the ESI-MS study, but **126** or **127** was observed upon the treatment with KI, **12**, and Cs_2_CO_3_. This result suggests that the formation of **126** or **127** from a combination of **11**/NIS or **11**/NCS/KI might occur via different reaction mechanisms and chloroamine **11a** did not undergo a simple chloride-iodide exchange process to form iodoamine **11b** while such chloride-iodide exchange process is commonly proposed in literature ^42,48^.

**Fig. 5:**
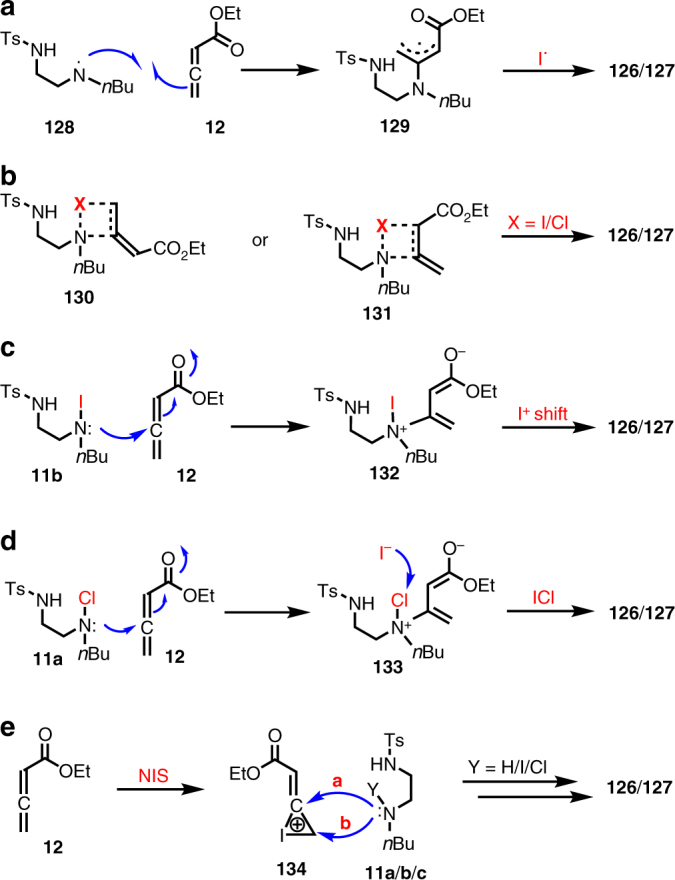
Proposed pathways for the first C−N bond formation. **a** A radical process for the C−N bond formation. **b** A concerted process for the C−N bond formation. **c** A stepwise ionic mechanism followed by I^+^ shift with iodoamine **11b**. **d** A stepwise ionic mechanism with chloroamine **11a** followed by ICl formation. **e** A stepwise ionic mechanism involving the formation of iodonium ion intermediate **134**

The detailed mechanism for the formation of the first carbon−nitrogen bond has not been well understood yet. This net addition of an iodo/chloroamine to the double bond of an allene substrate might go through a stepwise radical process^50,51^, a concerted process, or a stepwise ionic process^52,53^ (Fig. 5). Both the radical process and ionic process have been proposed for the reactions of *N*-iodo/chloroamine with olefins. In our case, a radical process would involve a homolytic cleavage of the N-I/Cl bond of **11b**/**11a** to generate nitrogen radical **128**, which would add on the sp carbon of allene **12** to form an allylic radical. The latter would be trapped by iodide to produce allylic iodide **126**/**127** (Fig. 5a). A concerted mechanism resembling the hydroboration process via a four-membered ring transition state (**130** or **131**) would give **126**/**127** as well (Fig. 5b). In this case, the more nucleophilic nitrogen atom would align up with the sp carbon of allene **12** to give the observed regiochemistry. Additionally, stepwise ionic process could be operational (Fig. 5c–e). For example, the iodoamine (**11b**) or chloroamine (**11a**) nitrogen could use its electron pair to undergo nucleophilic attack on the sp carbon of allene **12** to form **132** or **133** respectively. For **132**, an intramolecular or intermolecular I^+^ shift could happen to lead to **126**/**127** (Fig. 5c); for **133**, iodide from KI could pick up the chloride to form ICl, which could in turn serve as the I^+^ source to form **126**/**127** (Fig. 5d). A three-membered iodonium ion intermediate (**134**, Fig. 5e) generated from reacting iodoamine with allene **12** is unlikely because **12** is electron deficient and the nucleophilic ring opening step would prefer to attack the terminal carbon (path b) to give the opposite regioselectivity as observed.

**Fig. 6:**
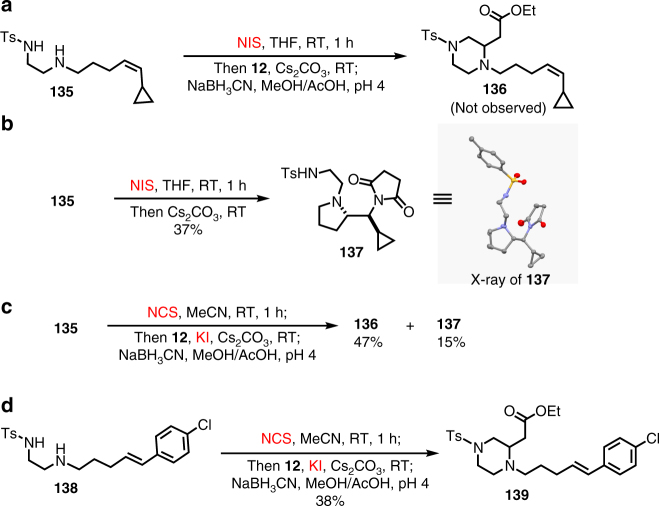
Probe reaction mechanism via **135** and **138**. **a** Using vinyl cyclopropane-containing substrate **135** to probe the radical mechanism for the first C−N bond formation under the NIS conditions. **b** Synthesis of **137** from **135** without the NaBH_3_CN reduction step and a crystal structure of **137**. **c** Using **135** to probe the radical mechanism for the first C−N bond formation under the NCS/KI conditions. **d** Using **138** to probe the radical mechanism for the first C−N bond formation under the NCS/KI conditions

To probe the addition process of *N*-iodo/chloroamine to allene substrate such as **12**, we prepared 1,2-diamine derivative **135** containing a vinyl cyclopropane moiety, a commonly used radical clock (Fig. 6a). When **135** was treated with the NIS reaction conditions, the reaction turned out to be quite complex and we did not observe the formation of desired piperazine product **136**; instead the formation of pyrrolidine product **137** was observed. We then removed the NaBH_3_CN reduction step and were able to identify **137** as the major product in 37% yield from a complex mixture (Fig. 6b). The structure of **137** was unambiguously confirmed by X-ray crystallographic analysis. The formation of **137** is presumably via an NIS promoted intramolecular iodoamination cyclization followed by replacement of the resulting iodide with succinimide. NIS could activate the *cis*-double bond directly or it could iodinate the secondary alkyl amine first and the resulting iodoamine would activate the double bond via an intramolecular I^+^ transfer process^54^. Interestingly, when we switched the NIS conditions to the NCS/KI conditions, desired piperazine product **136** was obtained as the major product in 47% yield along with **137** in 15% yield (Fig. 6c). Again, these results indicate that the reaction mechanism for the amphoteric diamination cyclizations under the NIS conditions is different from the ones under the NCS/KI conditions. The latter might involve the formation of iodine monochloride (ICl, Fig. 5d). Why the NCS/KI conditions are able to produce **136** and suppress the formation of **137** requires further investigation. Similar results were observed when substrate **138** was used. The NIS conditions gave a complex mixture, but the NCS/KI conditions provided desired product **139** in 38% (Fig. 6d). The formation of both **136** and **139** under the NCS/KI conditions suggests that a radical mechanism to form allylic iodide intermediates such as **126**/**127** observed in ESI-MS is unlikely because the nitrogen radical can be readily intercepted by the intramolecularly tethered olefins via a 5-*exo*-*trig* cyclization process.

**Fig. 7:**
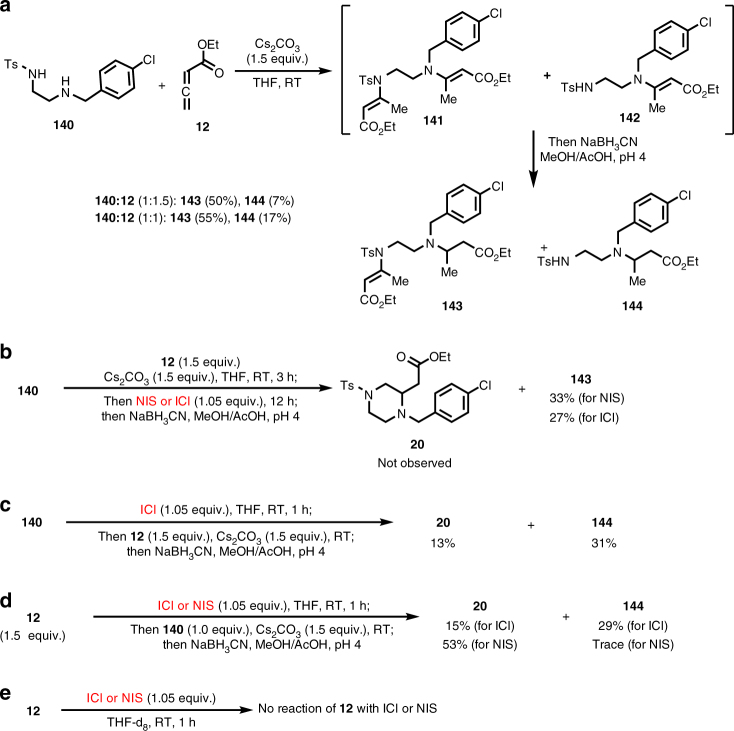
Probe reaction mechanism with **140** and **12** with NIS and ICl. **a** Reaction of **140** and **12** under Cs_2_CO_3_ conditions without NIS. **b** Reaction of **140** and **12** under Cs_2_CO_3_ conditions followed by addition of NIS or ICl. **c** Reaction of **140** and **12** under ICl conditions instead of the NIS conditions. **d** Pre-treatment of **12** with NIS or ICl before the addition of **140**. **e** Reaction of **12** with NIS or ICl in THF-d_8_

Additionally, in order to explore the roles of NIS and NCS/KI in these transformations, a few more experiments were explored (Fig. 7). First, a reaction between diamine **140** and allene **12** was conducted under basic conditions (Cs_2_CO_3_) in THF at room temperature. The formation of mono- and bis-nucleophilic addition products **142** and **141** were observed by ^1^H NMR analysis of the reaction mixture, which was further confirmed by a one-pot reduction of the corresponding vinylogous amide intermediates to **144** and **143**, respectively (Fig. 7a). Further treatment of the reaction mixture of **140** and **12** with NIS or ICl followed by NaBH_3_CN reduction did not result in the formation of **20**, but **143** in 33 or 27% yield, respectively (Fig. 7b). This result ruled out the possibility of **142** as a potential intermediate for the formation of **20**. Since ICl was proposed as a plausible intermediate when the combination of NCS/KI was employed, we used it to replace NIS and NCS/KI for the reaction of **140** with **12** and desired product **20** was formed in 13% yield along with **144** in 31% yield (Fig. 7c), which suggests that ICl is not as good as NIS or NCS to generate the corresponding iodo or chloroamine intermediate. This notion was corroborated by the fact that an unidentifiable mixture was generated when **140** was treated with ICl in THF-d_8_, but clear formation of the iodoamine intermediate was observed by ^1^H NMR analysis when NIS was used (Supplementary Fig. 7). Additionally, pre-treatment of allene **12** with NIS for 1 h before the addition of **140** or simply adding **12**, NIS and **140** at the same time was able to produce **20** in 53 or 56% yield, respectively, which is slightly lower than the yield (62%) of pre-treatment of **140** with NIS for 1 h before the addition of **12** (Table 2 and Fig. 7d). Interestingly, while pre-treatment of **140** with ICl for 1 h before the addition of **12** or pre-treatment of **12** with ICl for 1 h before the addition of **140** could form **20** in 13 or 15% yield, respectively, the operation of simply mixing **12**, ICl and **140** at the same time only afforded a trace amount of **20** (~2%). Our ^1^H NMR experiments showed that **12** did not react with NIS or ICl in THF-d_8_ after 1 h (Fig. 7e and Supplementary Fig. 8) and ICl reacted with THF solvent to produce a new product, which might be a THF polymerization or ring opening product, but need more experiments to elucidate. Overall, these experiments indicate that the successful formation of iodo or chloroamine intermediates is important for the desired amphoteric diamination and whether or not ICl is involved when NCS/KI was used remains unanswered.

In summary, an amphoteric diamination strategy has been developed to rapidly synthesize medicinally important carbon-substituted 1,4-diazo heterocycles including piperazines, 1,4-diazepanes, and 1,4-diazocanes. These *N*-heterocycles are otherwise difficult to access. This highly modular and convergent strategy utilizes readily available 1,2-, 1,3- or 1,4-diamine derivatives and electron-deficient allenes as the two coupling partners, which are assemble into saturated 1,4-diazo heterocycles via a formal [*n* + 2] (*n* = 4, 5, 6) cyclization mode. It does not require any precious transition metal catalysts. The reagents used are cheap and the reaction features mild reaction conditions, high functional group tolerance, and scalability (gram scale). In addition to a direct one-pot reduction, the vinylogous amide products can be converted to more complex products including α-CF_3_ substituted product and bicyclic products via a trifluoromethyl addition or an intromolecular Mannich-type reaction, respectively. We also demonstrated that both the tosyl group and nosyl group could be removed readily to allow further functionalization of the nitrogen atoms. Given the prestigious importance of these saturated *N*-heterocycles in medicines, the transformations reported here are expected to have broad applications in novel therapeutic discovery and development processes. Our current mechanistic studies showed that the formation of mono-iodinated or chlorinated amphoteric diamine intermediates is important for the success of the desired cyclization and a commonly proposed chloride-iodide exchange process and a radical N−C bond formation is unlikely when the combination of NCS/KI is used. Further detailed mechanistic studies and developing an enantioselective version of this reaction are currently undergoing in our laboratory.

## Methods

### General procedure for piperazine synthesis

To a 2-dram vial wrapped with aluminum foil was added a 1,2-diamine substrate (0.1 mmol), NIS (0.105 mmol), and dry THF (1 mL). The reaction mixture was stirred for 1 h under argon before an allene substrate (0.15 mmol) and Cs_2_CO_3_ (0.15 mmol) was added. After the reaction mixture was stirred for 24 h, NaBH_3_CN (0.2 mmol) and a co-solvent of MeOH/AcOH (pH = 4, 1 mL) were added to the reaction mixture. After 3 h, the reaction was quenched with a saturated aqueous solution of NaHCO_3_, extracted with CH_2_Cl_2_ for three times. The combined organic extracts were dried over Na_2_SO_4_ and concentrated in vacuo. The crude mixture was purified on silica gel column with hexane/EtOAc as eluents to give the desired piperazine product.

### Data availability

The X-ray crystallographic coordinates for the structures of **70** (CCDC 1561430), **71** (CCDC 1561427), **77** (CCDC 1561428), **75′** (CCDC 1583717), **137** (CCDC 1582123), a derivative of **76** (CCDC 1561429), and a derivative of **81** (CCDC 1561431) reported in this article have been deposited at the Cambridge Crystallographic Data Centre (CCDC). The data can be obtained free of charge from the Cambridge Crystallographic Data Centre via http://www.ccdc.cam.ac.uk/data_request/cif. The authors declare that the data supporting the findings of this study are available within the article and its Supplementary Information Files. All other data are available from the authors upon reasonable request.

## Electronic supplementary material

Supplementary Information
