# Supplementary material for: Expedient syntheses of N-heterocycles via intermolecular amphoteric diamination of allenes
Source: Nat Commun. 2018 Feb 19;9:721. doi: 10.1038/s41467-018-03085-3 (PMC5818626; doi:10.1038/s41467-018-03085-3)
Supplement: Supplementary file 1 — Supplementary Information [file 41467_2018_3085_MOESM1_ESM.docx]

**a**

**b**


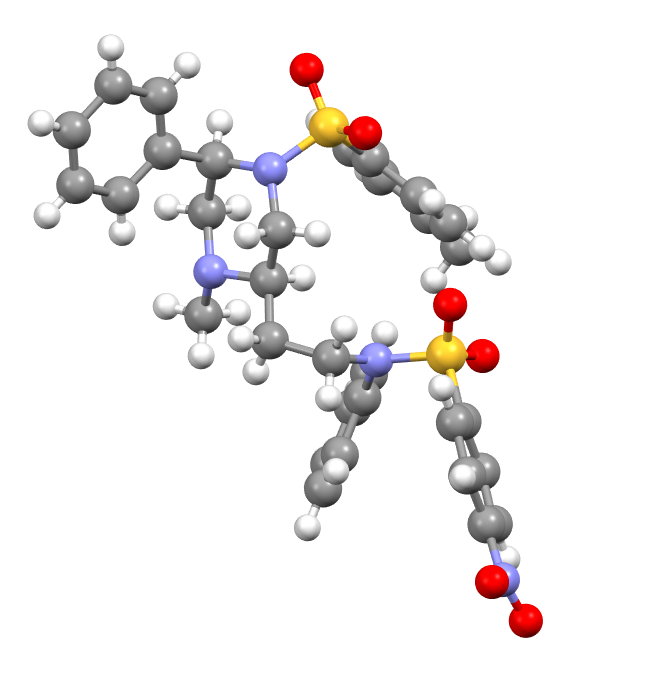


**Supplementary Figure 1.** Relative stereochemistry determination of **76**. **a** Synthesis of derivative **76b**. **b** X-ray crystal structure of **76b**.

**a**

**b**


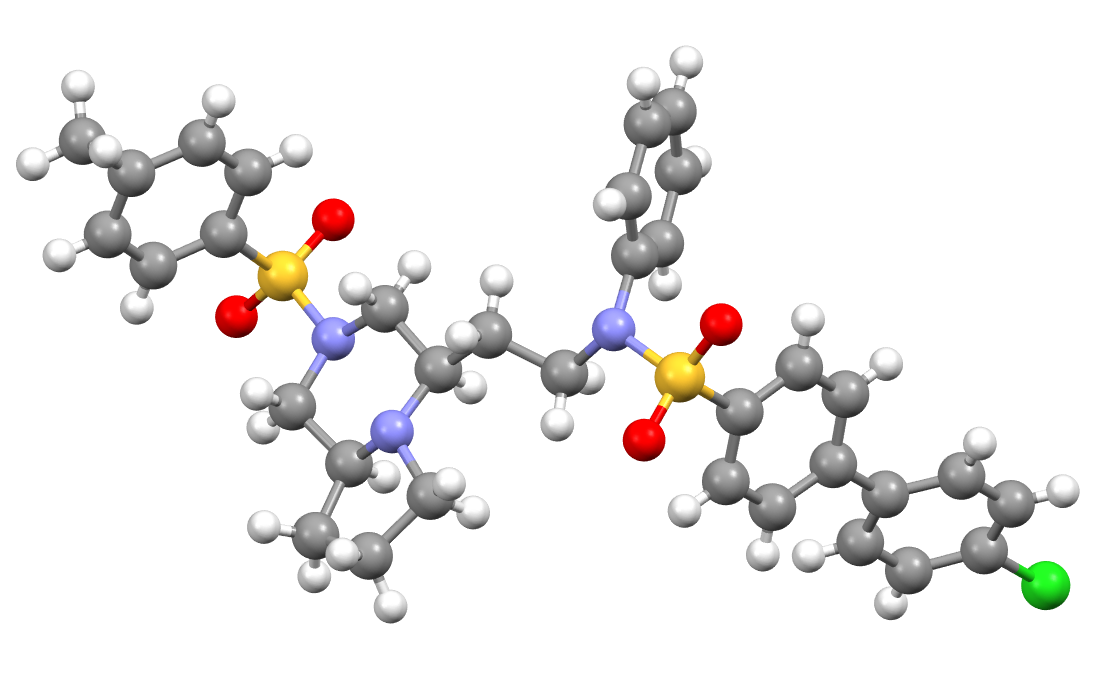


**Supplementary Figure 2.** Relative stereochemistry determination of **81**. **a** Synthesis of derivative **81b**. **b** X-ray crystal structure of **81b**.

**a**

**b**


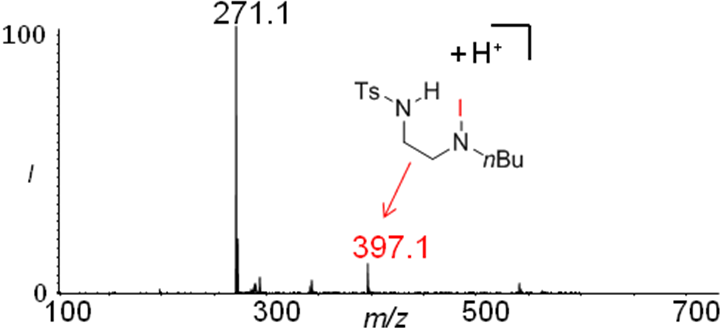


**c**


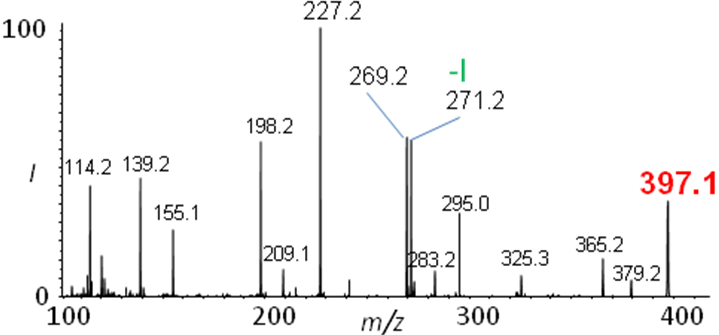


**Supplementary Figure 3.** ESI-MS study for the formation of **11b**. **a** Reaction of **11** with NIS to form **11b**. **b** NanoESI-MS result for the reaction. **c** MS/MS of m/z 397.1.

Note: MS analysis was performed by nanoelectrospray ionization-mass spectrometry (nanoESI-MS), using a 4000 QTRAP mass spectrometer (SCIEX, Toronto, ON, CA), equipped with a home-built nanoESI source. NanoESI tips (~10µm o.d.) were pulled from borosilicate glass capillary tips (1.5 mm o.d. and 0.86 mm i.d.) using a micropipette puller (P-1000 Flaming/Brown; Sutter Instrument, Novato, CA, USA). Instrument control, data acquisition, and processing were performed using Analyst software 1.6.2 (Applied Biosystems/Sciex). Typical MS parameters during the study were: nanoESI spray voltage of + (1400-1800) V; curtain gas of 10 psi; declustering potential of 20 V; and scan rate of 1000 Da/s, unless otherwise specified. As indicated, the instrument was used either in an linear ion trap mass analysis mode or in the tandem MS mode.

**a**

**b**


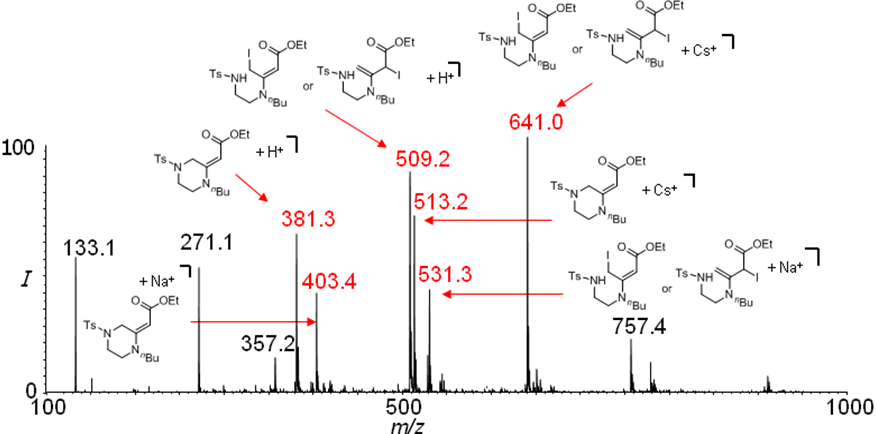


**c**

**
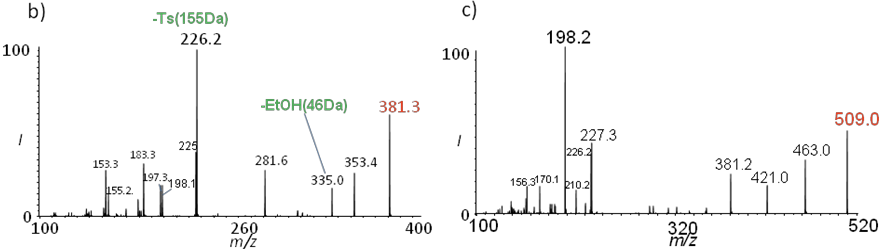
**

**d**

**
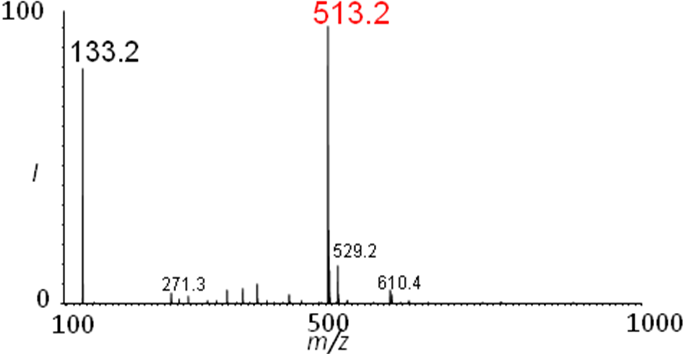
**

**e**


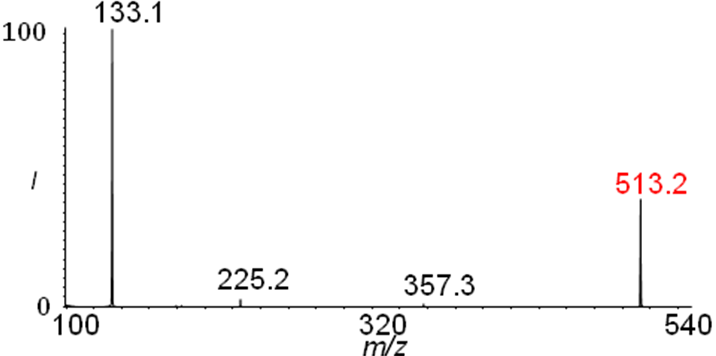


**Supplementary Figure 4.** ESI-MS study for the reaction of **11b** with **12**. **a** Proposed reaction pathways of **11b** and **12**. **b** NanoESI-MS result for the reaction at 1 h time point. **c** MS/MS of m/z 381.3 and 509.0. **d** NanoESI-MS result for the reaction at 24 h time point. **e** MS/MS of m/z 513.2.

**a**

**b**

**
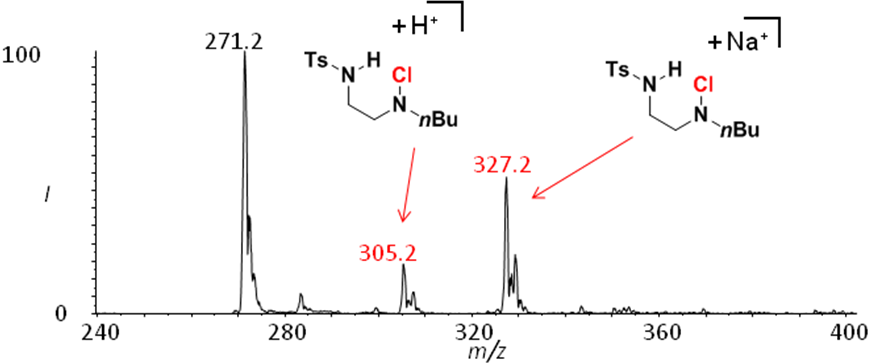
**

**c**

**
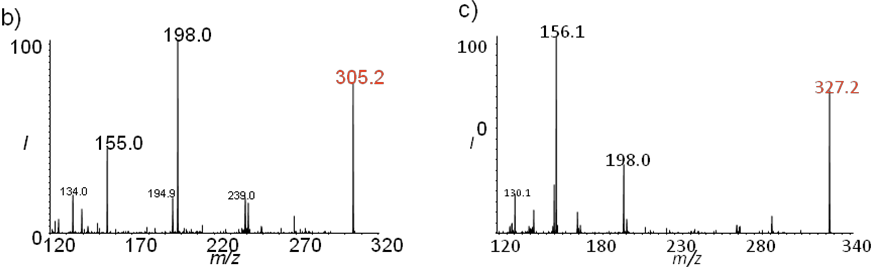
**

**Supplementary Figure 5.** ESI-MS study for the reaction of **11** with NCS. **a** Reaction of **11** with NCS. **b** NanoESI-MS result for the reaction at 1 h time point. **c** MS/MS of m/z 305.2 and 327.2.

**a**

**b**

**
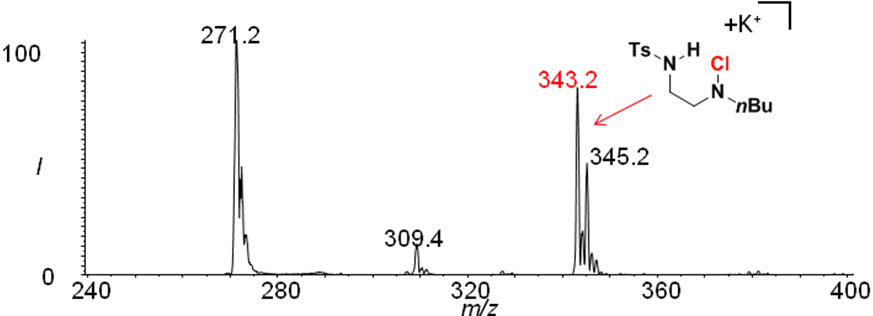
**

**c**

**
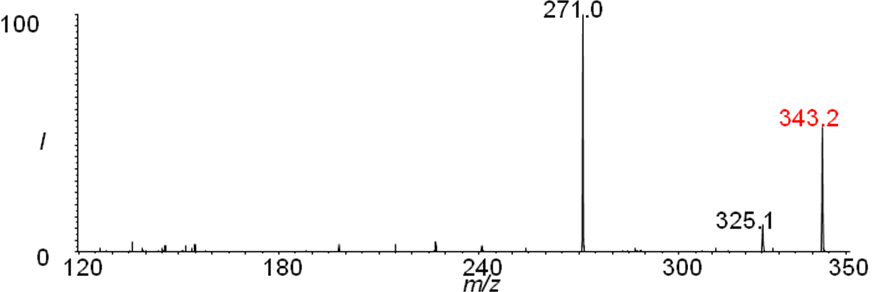
**

**Supplementary Figure 6.** ESI-MS study for the reaction of **11a** with KI. **a** Reaction of **11a** with KI. **b** NanoESI-MS result for the reaction at 1 h time point. **c** MS/MS of m/z 343.2.

**a**

**__**

**b**


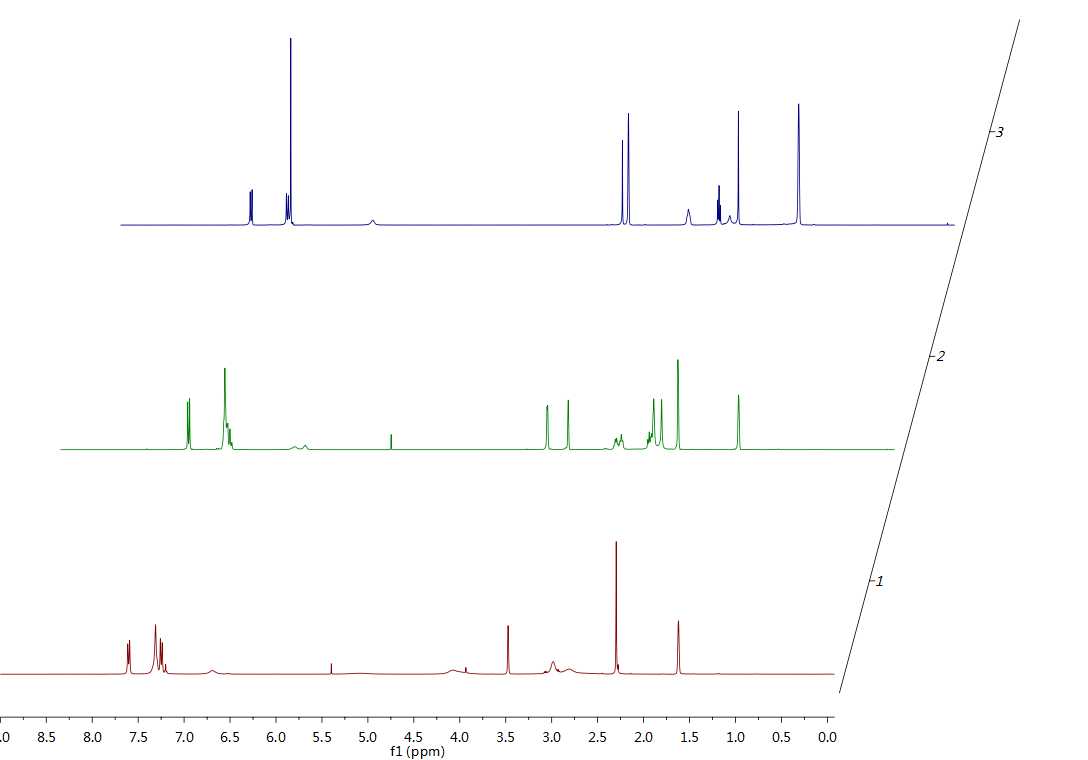


**Supplementary Figure 7.** ^1^H NMR experiments of diamine **140** with NIS or ICl in THF-d_8_. **a** Reaction of **140** with NIS or ICl: In a glove box and under dark condition, the reaction mixture of diamine (0.1 mmol), ICl (0.105mmol) or NIS (0.105mmol) in dry *d_8_*-THF (1 mL) was stirred for 1 h. Note: the product is very sensitive to light and very unstable. All the operation process must be under dark condition. **b** ^1^H NMR spectra of **140** (blue), **140** with NIS (green) and **140** with ICl (red) in THF-d_8_.

**a**

**__**

**b**


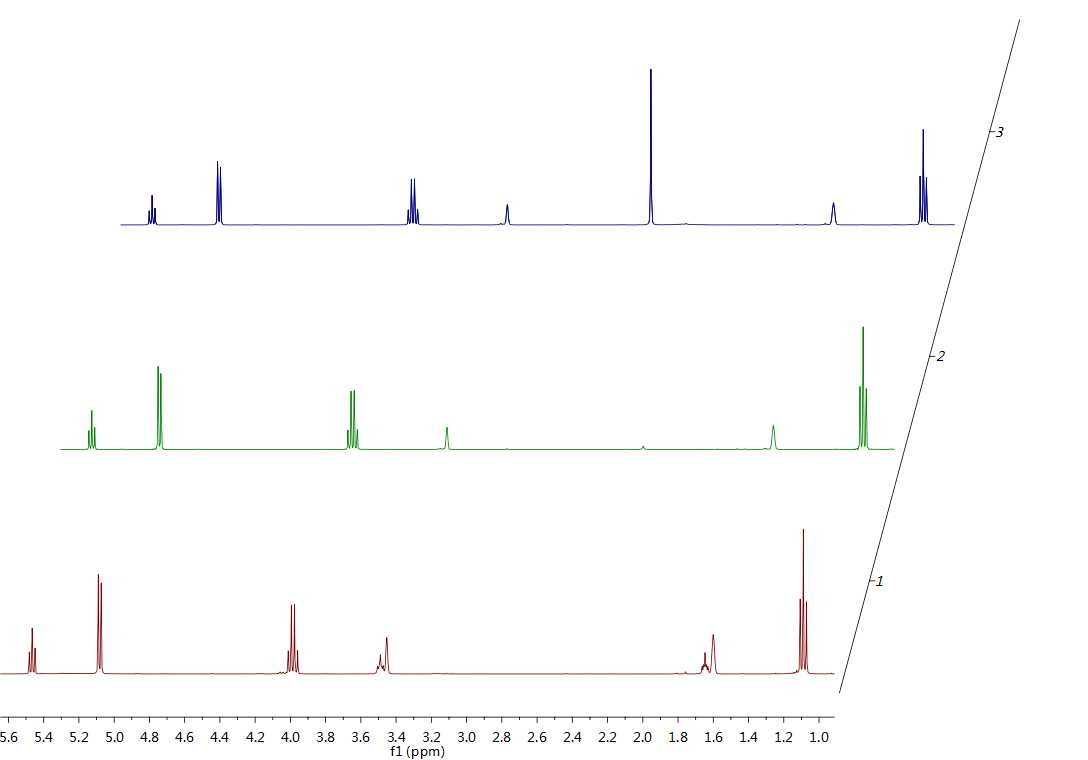


**Supplementary Figure 8.** ^1^H NMR experiments of allene **12** with NIS or ICl in THF-d_8_. **a** Reaction of **12** with NIS or ICl: In a glove box and under dark condition, the reaction mixture of allene **12** (0.1 mmol), ICl (0.105mmol) or NIS (0.105mmol) in dry *d_8_*-THF (1 mL) was stirred for 1 h. **b** ^1^H NMR spectra of **12** with NIS (blue), **12** (green) and **12** with ICl (red) in THF-d_8_. Note: The blue arrows in the ^1^H NMR spectra of **12** with the treatment of ICl in *d_8_*-THF indicate new peaks potentially derived from THF, but their identify remains unknown at the stage. Clearly, there was not reaction between **12** with NIS or ICl.

**Supplementary Methods**

**General Information**

General Methods: NMR spectra were recorded on Bruker spectrometers (^1^H at 400 MHz, 500 MHz, 800 MHz and ^13^C at 100 MHz, 125 MHz, 200 MHz). Chemical shifts (δ) were given in ppm with reference to solvent signals [^1^H NMR: CHCl_3_ (7.26); ^13^C NMR: CDCl_3_ (77.2), C_6_D_6_ (128.02), CD_3_OD (49.0)]. Column chromatography was performed on silica gel. All reactions sensitive to air or moisture were conducted under argon atmosphere in dry and freshly distilled solvents under anhydrous conditions, unless otherwise noted. Anhydrous THF and toluene were distilled over sodium benzophenone ketyl under argon. Anhydrous CH_2_Cl_2_ was distilled over calcium hydride under Argon. All other solvents and reagents were used as obtained from commercial sources without further purification.

**General procedure for syntheses of starting material 1,2-diamines^[1]^**

The reaction mixture of diamine (10 mmol), aldehyde (10 mmol) or ketone (10 mmol) and dry 4Å molecular sieve (5.0 g) in dry MeOH (20 mL) was stirred for overnight under argon at room temperature. Then the reaction was then cooled to 0 ^o^C before NaBH_4_ (15 mmol) was added slowly. The reaction process was monitored by TLC. Upon full conversion of the starting material, the 4Å molecular sieve was removed by filtration. DCM (100 mL) and water were added to the filtrate. The organic layer was separated and the aqueous phase was further extracted with DCM three times. The combined organic extracts were dried over Na_2_SO_4_, filtered, and concentrated in vacuo. The crude residue was purified on a silica gel column (the silica gel was pre-treated with Et_3_N) with hexane/EtOAc as the eluents to give the desired products.

**Synthesis of 11a:**

To a solution of **11** (2.7 g, 10 mmol) in 20 mL THF was added NCS (1.46 g, 11 mmol). The reaction mixture was stirred for 1 h under dark and argon atmosphere before it was quenched by water and diluted with DCM. The organic layer was separated and the aqueous phase was further extracted with DCM three times. The combined organic extracts were dried over Na_2_SO_4_ and concentrated in vacuo. Purification was performed quickly by a silica gel column eluted with hexane/EtOAc to give desire product (2.69 g, 88% yield, white solid).

^1^H NMR (500 MHz, CDCl_3_) δ 7.78 (d, *J* = 8.3 Hz, 2H), 7.33 (d, *J* = 8.2 Hz, 2H), 4.96 (s, 1H), 3.23 (dd, *J* = 10.9, 5.7 Hz, 2H), 3.00 – 2.93 (m, 2H), 2.92 – 2.83 (m, 2H), 2.45 (s, 3H), 1.60 (dt, *J* = 12.6, 7.4 Hz, 2H), 1.35 (dt, *J* = 15.0, 7.4 Hz, 2H), 0.94 (t, *J* = 7.4 Hz, 3H); ^13^C NMR (125 MHz, CDCl_3_) δ 143.6, 136.9, 129.9, 127.3, 64.4, 62.0, 40.9, 29.8, 21.7, 20.0, 14.0; ESI-MS: 305.2 [M+H]^+^.

**Genaral procedure for syntheses of piperazines**

To a 2-dram vial wrapped with aluminum foil was added a 1,2-diamine substrate (0.1 mmol), NIS (0.105 mmol), and dry THF (1 mL). The reaction mixture was stirred for 1 h under argon before an allene substrate (0.15 mmol) and Cs_2_CO_3_ (0.15 mmol) was added. After the reaction mixture was stirred for 24 h, NaBH_3_CN (0.2 mmol) and a co-solvent of MeOH/AcOH (pH = 4, 1mL) were added to the reaction mixture. After 3 h, the reaction was quenched with a saturated aqueous solution of NaHCO_3_, extracted with CH_2_Cl_2_ for three times. The combined organic extracts were dried over Na_2_SO_4_, filtered, and concentrated in vacuo. The crude mixture was purified on silica gel column with hexane/EtOAc as eluents to give the desired piperazine product.

**14:** 26.9 mg, 71% yield (**4 mmol diamine, 1.105 g product, 76% yield**), colorless oil; ^1^H NMR (500 MHz, CDCl_3_) δ 7.61 (d, *J* = 8.2 Hz, 2H), 7.31 (d, *J* = 8.0 Hz, 2H), 4.23 – 4.09 (m, 2H), 3.29 – 3.11 (m, 3H), 2.82 – 2.30 (m, 11H), 1.37 – 1.18 (m, 7H), 0.85 (t, *J* = 7.3 Hz, 3H); ^13^C NMR (125 MHz, CDCl_3_) δ 172.4, 143.8, 132.6, 129.8, 128.0, 60.8, 54.4, 53.5, 50.0, 47.3, 46.0, 30.3, 29.1, 21.6, 20.6, 14.3, 14.0; IR (neat): 1727, 1350, 1166, 1107, 949, 663, 548 cm^-1^; HRMS (ESI) Calculated for C_19_H_31_N_2_O_4_S [M+H]^+^ 383.1999, found 383.1998.

**19:** 27.4 mg, 63% yield, colorless oil; ^1^H NMR (500 MHz, CDCl_3_) δ 7.62 (d, *J* = 8.1 Hz, 2H), 7.32 (d, *J* = 8.0 Hz, 2H), 7.19 – 7.17(m, 2H), 6.94 (t, *J* = 8.6 Hz, 2H), 4.15 (q, *J* = 7.1 Hz, 2H), 3.63 (d, *J* = 13.3 Hz, 1H), 3.41 (d, *J* = 13.3 Hz, 1H), 3.23 – 3.14 (m, 3H), 2.90 – 2.88 (m, 1H), 2.79 – 2.53 (m, 4H), 2.44 (s, 3H), 2.42 – 2.34 (m, 1H), 1.26 (t, *J* = 7.1 Hz, 3H); ^13^C NMR (125 MHz, CDCl_3_) δ 172.1, 162.2 (d, *J* = 245.3 Hz), 143.8, 133.8, 132.7, 130.3 (d, *J* = 7.9 Hz), 129.8, 127.9, 115.3 (d, *J* = 21.2 Hz), 60.9, 57.5, 55.1, 50.0, 46.9, 45.9, 31.2, 21.7, 14.3; ^19^F NMR (470 MHz, CDCl_3_) δ -115.47; IR (neat): 1727, 1508, 1350, 1219, 1166, 959, 670, 549 cm^-1^; HRMS (ESI) Calculated for C_22_H_28_FN_2_O_4_S [M+H]^+^ 435.1748, found 435.1753.

**20:** 27.7 mg, 62% yield, colorless oil; ^1^H NMR (500 MHz, CDCl_3_) δ 7.64 (d, *J* = 8.2 Hz, 2H), 7.35 (d, *J* = 8.0 Hz, 2H), 7.26 – 7.22 (m, 2H), 7.17 (d, *J* = 8.4 Hz, 2H), 4.17 (q, *J* = 7.1 Hz, 2H), 3.65 (d, *J* = 13.5 Hz, 1H), 3.43 (d, *J* = 13.5 Hz, 1H), 3.28 – 3.14 (m, 3H), 2.92 – 2.97 (m, 1H), 2.75 – 2.58 (m, 4H), 2.46 (s, 3H), 2.43 – 2.39 (m, 1H), 1.28 (t, *J* = 7.1 Hz, 3H); ^13^C NMR (125 MHz, CDCl_3_) δ 172.0, 143.8, 136.7, 133.0, 132.7, 130.0, 129.8, 128.6, 127.9, 60.9, 57.5, 55.1, 50.0, 47.0, 45.9, 31.2, 21.7, 14.3. IR (neat): 1727, 1350, 1165, 1089, 959, 670, 545 cm^-1^; HRMS (ESI) Calculated for C_22_H_28_ClN_2_O_4_S [M+H]^+^ 451.1453, found 451.1450.

**21:** 28.3 mg, 57% yield, colorless oil;^1^H NMR (500 MHz, CDCl_3_) δ 7.62 (d, *J* = 7.9 Hz, 2H), 7.51 (d, *J* = 7.8 Hz, 2H), 7.38 – 7.27 (m, 4H), 4.15 (q, *J* = 7.1 Hz, 2H), 3.72 (d, *J* = 13.7 Hz, 1H), 3.52 (d, *J* = 13.8 Hz, 1H), 3.24 –3.15 (m, 3H), 2.92 – 2.91 (d, *J* = 8.7 Hz, 1H), 2.80 – 2.56 (m, 4H), 2.46 – 2.31 (m, 4H), 1.25 (t, *J* = 7.1 Hz, 3H); ^13^C NMR (125 MHz, CDCl_3_) δ 172.0, 143.9, 142.5, 132.7, 129.9, 129.6, 128.9, 127.9, 125.4, 123.2, 60.9, 57.8, 55.3, 50.0, 47.1, 45.9, 31.4, 21.7, 14.3; ^19^F NMR (470 MHz, CDCl_3_) δ -62.48. IR (neat): 1729, 1350, 1324, 1165, 1121, 1105, 1065, 950, 816, 671, 549 cm^-1^; HRMS (ESI) Calculated for C_23_H_28_F_3_N_2_O_4_S [M+H]^+^ 485.1716, found 485.1716.

**22:** 21.3 mg, 50% yield, colorless oil; ^1^H NMR (500 MHz, CDCl_3_) δ 7.61 (d, *J* = 7.9 Hz, 2H), 7.32 (d, *J* = 7.8 Hz, 2H), 7.10 – 7.06 (m, 4H), 4.15 (q, *J* = 7.1 Hz, 2H), 3.62 (d, *J* = 13.1 Hz, 1H), 3.43 – 3.37 (m, 1H), 3.26 – 3.03 (m, 3H), 2.91 – 2.89 (m, 1H), 2.79 – 2.56 (m, 4H), 2.47 – 2.38 (m, 4H), 2.27 (s, 3H), 1.26 (t, *J* = 7.1 Hz, 3H); ^13^C NMR (125 MHz, CDCl_3_) δ 172.1, 143.8, 137.0, 135.0 132.8, 129.8, 129.3, 129.1, 129.0, 128.8, 127.9, 60.8, 58.0, 55.1, 50.04, 46.9, 46.0, 31.2, 21.7, 21.2, 14.3; IR (neat): 1729, 1456 1351, 1166, 958, 815, 670, 550 cm^-1^; HRMS (ESI) Calculated for C_23_H_31_N_2_O_4_S [M+H]^+^ 431.1999, found 431.2007.

**23:** 33.4 mg, 72% yield, colorless oil; ^1^H NMR (500 MHz, CDCl_3_) δ 8.11 (d, *J* = 8.6 Hz, 2H), 7.62 (d, *J* = 8.2 Hz, 2H), 7.40 (d, *J* = 8.6 Hz, 2H), 7.34 (d, *J* = 8.0 Hz, 2H), 4.14 (q, *J* = 7.1 Hz, 2H), 3.76 (d, *J* = 14.3 Hz, 1H), 3.56 (d, *J* = 14.3 Hz, 1H), 3.33 – 3.13 (m, 3H), 2.93 –2.91(m, 1H), 2.75 – 2.56 (m, 4H), 2.47 – 2.37 (m, 4H), 1.25 (t, *J* = 7.1 Hz, 3H); ^13^C NMR (125 MHz, CDCl_3_) δ 171.8, 147.4, 146.1, 143.9, 132.7, 129.9, 129.2, 127.9, 123.7, 61.0, 57.6, 55.3, 50.0, 47.3, 45.8, 31.4, 21.7, 14.3; IR (neat): 1727, 1518, 1343, 1164, 1108, 951, 738, 670, 560, 548 cm^-1^; HRMS (ESI) Calculated for C_23_H_28_N_3_O_6_S [M+H]^+^ 462.1693, found 462.1698.

**24:** 22.1 mg, 53% yield, colorless oil; ^1^H NMR (500 MHz, CDCl_3_) δ 7.64 (d, *J* = 8.2 Hz, 2H), 7.37 – 7.20 (m, 7H), 4.17 (q, *J* = 7.1 Hz, 2H), 3.69 (d, *J* = 13.3 Hz, 1H), 3.47 (d, *J* = 13.3 Hz, 1H), 3.27 – 3.15 (m, 3H), 2.95 – 2.93 (m, 1H), 2.81 – 2.61 (m, 4H), 2.51 – 2.39 (m, 4H), 1.28 (t, *J* = 7.1 Hz, 3H); ^13^C NMR (125 MHz, CDCl_3_) δ 172.1, 143.8, 138.1, 132.8, 129.8, 128.8, 128.5, 127.9, 127.4, 60.9, 58.3, 55.2, 50.1, 47.0, 46.0, 31.3, 21.7, 14.3; IR (neat): 1728, 1454, 1351, 1166, 959, 758, 670, 548 cm^-1^; HRMS (ESI) Calculated for C_22_H_29_N_2_O_4_S [M+H]^+^ 417.1843, found 417.1853.

**25:** 30.7 mg, 68% yield, colorless oil; ^1^H NMR (500 MHz, CDCl_3_) δ 7.62 (d, *J* = 8.2 Hz, 2H), 7.32 (d, *J* = 8.0 Hz, 2H), 7.17 (t, *J* = 7.8 Hz, 1H), 6.82 – 6.72 (m, 3H), 4.15 (q, *J* = 7.1 Hz, 2H), 3.79 – 3.73 (m, 3H), 3.64 (d, *J* = 13.4 Hz, 1H), 3.42 (d, *J* = 13.4 Hz, 1H), 3.24 – 3.13 (m, 3H), 2.92 – 2.90 (m, 1H), 2.79 – 2.56 (m, 4H), 2.48 – 2.39 (m, 4H), 1.26 (t, *J* = 7.1 Hz, 3H); ^13^C NMR (125 MHz, CDCl_3_) δ 172.1, 159.8, 143.8, 139.9, 132.7, 129.8, 129.4, 127.9, 121.1, 114.6, 112.4, 60.9, 58.2, 55.3, 55.2, 50.0, 47.0, 45.9, 31.3, 21.7, 14.3; IR (neat): 1729, 1585, 1489, 1350, 1262, 1167, 1046, 960, 549 cm^-1^; HRMS (ESI) Calculated for C_23_H_31_N_2_O_5_S [M+H]^+^ 447.1948, found 447.1943.

**26:** 30.9 mg, 67% yield, colorless oil; ^1^H NMR (500 MHz, CDCl_3_) δ 7.62 (d, *J* = 8.2 Hz, 2H), 7.32 (d, *J* = 8.2 Hz, 2H), 7.16 (t, *J* = 7.9 Hz, 1H), 6.74 – 6.65 (m, 3H), 4.14 (q, *J* = 7.1 Hz, 2H), 3.76 (s, 3H), 3.32 – 3.15 (m, 3H), 2.89 – 2.47 (m, 10H), 2.43 (s, 3H), 1.26 (t, *J* = 7.1 Hz, 3H); ^13^C NMR (125 MHz, CDCl_3_) δ 172.2, 159.7, 143.8, 141.5, 132.6, 129.8, 129.5, 128.0, 121.1, 114.6, 111.5, 60.9, 55.6, 55.3, 54.4, 49.9, 47.4, 45.9, 33.9, 30.6, 21.7, 14.3; IR (neat): 1728, 1600, 1456, 1350, 1259, 1167, 756, 667, 549 cm^-1^; HRMS (ESI) Calculated for C_24_H_33_N_2_O_4_S [M+H]^+^ 461.2105, found 461.2100.

**27:** 31.0 mg, 65% yield, colorless oil; ^1^H NMR (500 MHz, CDCl_3_) δ 7.62 (d, *J* = 8.2 Hz, 2H), 7.32 (d, *J* = 8.0 Hz, 2H), 6.39 – 6.38 (m, 2H), 6.32 – 6.31 (m, 1H), 4.14 (q, *J* = 7.1 Hz, 2H), 3.74 (s, 6H), 3.60 (d, *J* = 13.5 Hz, 1H), 3.39 (d, *J* = 13.5 Hz, 1H), 3.26 – 3.08 (m, 3H), 2.94 – 2.92 (m, 1H), 2.79 – 2.56 (m, 4H), 2.48 – 2.40 (m, 4H), 1.25 (t, *J* = 7.1 Hz, 3H); ^13^C NMR (125 MHz, CDCl_3_) δ 172.0, 161.0, 143.7, 140.8, 133.1, 129.8, 127.9, 106.8, 99.0, 60.8, 58.4, 55.4, 55.2, 50.0, 47.2, 45.9, 31.5, 21.6, 14.3; IR (neat): 1728, 1596, 1458, 1350, 1204, 1155, 1063, 549 cm^-1^; HRMS (ESI) Calculated for C_24_H_33_N_2_O_6_S [M+H]^+^ 477.2054, found 477.2051.

**28:** 33.4 mg, 66% yield, colorless oil; ^1^H NMR (500 MHz, CDCl_3_) δ 7.62 (d, *J* = 8.2 Hz, 2H), 7.49 (d, *J* = 7.6 Hz, 1H), 7.33 – 7.19 (m, 3H), 7.21 (t, *J* = 7.3 Hz, 1H), 7.11 – 7.02 (m, 1H), 4.15 (q, *J* = 7.1 Hz, 2H), 3.68 (d, *J* = 14.2 Hz, 1H), 3.59 (d, *J* = 14.3 Hz, 1H), 3.38 – 3.20 (m, 3H), 2.92 – 2.60 (m, 5H), 2.52 – 2.41 (m, 4H), 1.26 (t, *J* = 7.1 Hz, 3H); ^13^C NMR (125 MHz, CDCl_3_) δ 172.2, 143.8, 137.3, 133.0, 132.9, 130.6, 129.8, 128.8, 127.9, 127.3, 124.6, 60.9, 57.7, 55.2, 49.9, 46.7, 45.9, 31.0, 21.7, 14.3; IR (neat): 1729, 1351, 1167, 1026, 955, 757, 670, 549 cm^-1^; HRMS (ESI) Calculated for C_22_H_28_BrN_2_O_4_S [M+H]^+^ 495.0948, found 495.0958.

**29:** 31.4 mg, 64% yield, colorless oil; ^1^H NMR (500 MHz, CDCl_3_) δ 7.62 (d, *J* = 8.2 Hz, 2H), 7.37 – 7.32 (m, 4H), 7.17 – 7.08 (m, 2H), 4.15 (q, *J* = 7.1 Hz, 2H), 3.63 (d, *J* = 13.6 Hz, 1H), 3.41 (d, *J* = 13.6 Hz, 1H), 3.25 – 3.18 (m, 2H), 3.14 – 3.13 (m, 1H), 2.92 – 2.90 (d, *J* = 8.7 Hz, 1H), 2.73 – 2.58 (m, 4H), 2.46 – 2.36 (m, 4H), 1.26 (t, *J* = 7.1 Hz, 3H); ^13^C NMR (123 MHz, CDCl_3_) δ 172.0, 143.9, 140.7, 132.7, 131.6, 130.5, 130.0, 129.9, 127.9, 127.3, 122.7, 60.9, 57.7, 55.2, 50.0, 47.1, 45.8, 21.7, 14.3. IR (neat): 1729, 1351, 1184, 1167, 960, 670, 549 cm^-1^; HRMS (ESI) Calculated for C_22_H_28_BrN_2_O_4_S [M+H]^+^ 495.0948, found 495.0957.

**30:** 17.5 mg, 42% yield, colorless oil; ^1^H NMR (500 MHz, CDCl_3_) δ 8.53 (d, *J* = 4.8 Hz, 1H), 7.67 – 7.59 (m, 3H), 7.35 – 7.30 (m, 3H), 7.17 – 7.14 (m, 1H), 4.17 (q, *J* = 7.1 Hz, 2H), 3.81 (d, *J* = 14.0 Hz, 1H), 3.68 (d, *J* = 14.0 Hz, 1H), 3.32 – 3.18 (m, 3H), 2.96 – 2.94 (m, 1H), 2.84 – 2.66 (m, 4H), 2.57 – 2.50 (m, 1H), 2.46 (s, 3H), 1.28 (t, *J* = 7.1 Hz, 3H); ^13^C NMR (125 MHz, CDCl_3_) δ 172.0, 158.3, 149.5, 143.8, 136.7, 132.6, 129.8, 128.0, 123.1, 122.4, 60.9, 59.9, 55.1, 49.6, 47.4, 45.6, 31.4, 21.7, 14.3; IR (neat): 1729, 1590, 1351, 1167, 1092, 952, 762, 671, 549 cm^-1^; HRMS (ESI) Calculated for C_21_H_28_N_3_O_4_S [M+H]^+^ 418.1795, found 418.1794.

**31:** 21.7 mg, 47% yield, colorless oil; ^1^H NMR (500 MHz, CDCl_3_) δ 8.80 (d, *J* = 4.3 Hz, 1H), 8.09 (d, *J* = 8.4 Hz, 1H), 8.04 (d, *J* = 8.4 Hz, 1H), 7.68 (t, *J* = 7.4 Hz, 1H), 7.62 (d, *J* = 8.1 Hz, 2H), 7.50 (t, *J* = 7.5 Hz, 1H), 7.35 – 7.30 (m, 3H), 4.12 – 4.08 (m, 3H), 3.94 (d, *J* = 14.4 Hz, 1H), 3.38 – 3.20 (m, 3H), 2.93 – 2.91 (m, 1H), 2.84 – 2.68 (m, 4H), 2.51 – 2.48 (m, 1H), 2.43 (s, 3H), 1.24 (t, *J* = 7.1 Hz, 3H); ^13^C NMR (125 MHz, CDCl_3_) δ 172.0, 150.2, 148.5, 143.9, 143.4, 132.8, 130.3, 129.9, 129.3, 127.9, 127.4, 126.6, 123.8, 121.1, 61.0, 55.6, 55.2, 50.0, 47.3, 45.8, 31.2, 21.7, 14.3; IR (neat): 1728, 1457, 1350, 1184, 1167, 955, 759, 549 cm^-1^; HRMS (ESI) Calculated for C_25_H_30_N_3_O_4_S [M+H]^+^ 468.1952, found 468.1967.

**32:** 24.4 mg, 60% yield, colorless oil; ^1^H NMR (500 MHz, CDCl_3_) δ 7.60 (d, *J* = 8.2 Hz, 2H), 7.34 – 7.28 (m, 3H), 6.28 – 6.27 (m, 1H), 6.16 (d, *J* = 3.1 Hz, 1H), 4.15 (q, *J* = 7.1 Hz, 2H), 3.67 – 3.57 (m, 2H), 3.21 – 2.95 (m, 4H), 2.92 – 2.84 (m, 1H), 2.72 – 2.49 (m, 4H), 2.41 (s, 3H), 1.27 (t, *J* = 7.2 Hz, 3H); ^13^C NMR (125 MHz, CDCl_3_) δ 171.8, 150.9, 143.8, 142.6, 132.4, 129.8, 128.0, 110.3, 109.3, 60.9, 54.5, 50.5, 50.1, 47.8, 45.9, 31.5, 21.6, 14.3; IR (neat): 1728, 1349, 1166, 1090, 1017, 955, 750, 672, 659, 548 cm^-1^; HRMS (ESI) Calculated for C_20_H_27_N_2_O_5_S [M+H]^+^ 407.1635, found 407.1630.

**33:** 38.6 mg, 76% yield, colorless oil; ^1^H NMR (500 MHz, CDCl_3_) δ 7.61 (d, *J* = 8.2 Hz, 2H), 7.31 (d, *J* = 8.1 Hz, 2H), 7.12 – 7.11 (m, 1H), 6.04 – 6.01 (m, 2H), 4.14 (q, *J* = 7.1 Hz, 2H), 3.78 (s, 2H), 3.36 – 3.26 (m, 3H), 2.74 – 2.56 (m, 6H), 2.42 (s, 3H), 1.51 (s, 9H), 1.26 (t, *J* = 7.1 Hz, 3H); ^13^C NMR (125 MHz, CDCl_3_) δ 172.3, 149.2, 143.67, 132.7, 132.1, 129.7, 127.9, 121.9, 113.2, 109.8, 83.4, 60.7, 54.7, 51.4, 49.9, 46.5, 46.1, 30.4, 28.1, 21.6, 14.3; IR (neat): 1732, 1370, 1334, 1316, 1167, 1129, 953, 725, 549 cm^-1^; HRMS (ESI) Calculated for C_25_H_35_N_3_O_6_S [M+H]^+^ 506.2319, found 506.2320.

**34:** 36.5 mg, 66% yield, colorless oil; ^1^H NMR (500 MHz, CDCl_3_) δ 7.94 (d, *J* = 8.3 Hz, 1H), 7.63 (d, *J* = 8.2 Hz, 2H), 7.42 (d, *J* = 7.5 Hz, 1H), 7.33 (d, *J* = 8.0 Hz, 2H), 7.23 – 7.15 (m, 2H), 6.48 (s, 1H), 4.15 (q, *J* = 7.1 Hz, 2H), 3.98 (d, J = 15.7 Hz, 1H), 3.92 (d, J = 15.7 Hz, 1H), 3.45 – 3.27 (m, 3H), 2.85 – 2.72 (m, 3H), 2.68 – 2.57 (m, 3H), 2.45 (s, 3H), 1.61 (s, 8H), 1.26 (t, *J* = 7.1 Hz, 3H); ^13^C NMR (125 MHz, CDCl_3_) δ 172.2, 150.4, 143.7, 138.5, 136.8, 132.7, 129.7, 128.9, 127.9, 123.7, 122.7, 120.3, 115.2, 108.8, 83.9, 60.8, 54.9, 52.8, 50.9, 46.6, 46.1, 30.6, 28.3, 21.6, 14.3; IR (neat): 1730, 1454, 1370, 1352, 1329, 1167, 1119, 1089, 953, 748, 548 cm^-1^; HRMS (ESI) Calculated for C_29_H_38_N_3_O_6_S [M+H]^+^ 556.2476, found 556.2467.

**35:** 36.6 mg, 66% yield, colorless oil; ^1^H NMR (500 MHz, CDCl_3_) δ 8.07 (s, 1H), 7.61 (d, *J* = 8.2 Hz, 2H), 7.56 (d, *J* = 7.8 Hz, 1H), 7.43 (s, 1H), 7.32 – 7.26 (m, 3H), 7.16 (t, *J* = 7.5 Hz, 1H), 4.15 (q, *J* = 7.1 Hz, 2H), 3.80 (d, *J* = 13.5 Hz, 1H), 3.59 (d, *J* = 13.5 Hz, 1H), 3.36 – 3.10 (m, 3H), 2.92 – 2.90 (m, 1H), 2.78 – 2.64 (m, 4H), 2.51 – 2.49 (m, 1H), 2.43 (s, 3H), 1.66 (s, 9H), 1.26 (t, *J* = 7.1 Hz, 3H); ^13^C NMR (125 MHz, CDCl_3_) δ 172.1, 149.8, 143.8, 135.8, 132.9, 130.2, 129.8, 127.9, 124.7, 124.6, 122.5, 119.9, 117.1, 115.3, 83.9, 60.9, 55.1, 49.9, 49.4, 47.1, 45.9, 31.1, 28.3, 21.7, 14.3; IR (neat): 1730, 1452, 1370, 1350, 1256, 1166, 1087, 1017, 960, 749, 548 cm^-1^; HRMS (ESI) Calculated for C_29_H_38_N_3_O_6_S [M+H]^+^ 556.2476, found 556.2477.

**36:** 25.9 mg, 61% yield, colorless oil; ^1^H NMR (500 MHz, CDCl_3_) δ 7.61 (d, *J* = 8.2 Hz, 2H), 7.31 (d, *J* = 8.1 Hz, 2H), 4.14 (q, *J* = 7.1 Hz, 2H), 3.32 – 3.10 (m, 3H), 2.77 – 2.56 (m, 4H), 2.51 – 2.40 (m, 5H), 2.22 – 2.07 (m, 2H), 1.64 – 1.62 (m, 5H), 1.39 – 1.22 (m, 4H), 1.20 – 1.05 (m, 3H), 0.83 – 0.69 (m, 2H); ^13^C NMR (125 MHz, CDCl_3_) δ 171.6, 142.7, 131.8, 128.8, 126.9, 59.8, 53.9, 48.8, 46.4, 45.0, 34.5, 30.7, 30.6, 29.3, 25.9, 25.1, 20.7, 13.3; IR (neat): 17.29, 1351, 1168, 1120, 1091, 953, 671, 549 cm^-1^; HRMS (ESI) Calculated for C_22_H_35_N_2_O_4_S [M+H]^+^ 423.2312, found 423.2316.

**37:** 25.0 mg, 65% yield, colorless oil; ^1^H NMR (500 MHz, CDCl_3_) δ 7.62 (d, *J* = 8.2 Hz, 2H), 7.32 (d, *J* = 8.0 Hz, 2H), 4.14 (q, *J* = 7.1 Hz, 2H), 3.33 – 3.12 (m, 3H), 2.76 – 2.58 (m, 4H), 2.53 – 2.41 (m, 5H), 2.18 – 2.04 (m, 2H), 1.65 – 1.60 (m, 1H), 1.27 (t, *J* = 7.1 Hz, 3H), 0.79 (t, *J* = 6.0 Hz, 6H); ^13^C NMR (125 MHz, CDCl_3_) δ 172.6, 143.7, 132.8, 129.8, 127.9, 62.1, 60.8, 54.9, 49.8, 47.2, 45.9, 30.2, 26.0, 21.7, 20.8, 20.6, 14.3; IR (neat): 1730, 1352, 1168, 1112, 953, 671, 562 cm^-1^; HRMS (ESI) Calculated for C_19_H_31_N_2_O_4_S [M+H]^+^ 383.1999, found 383.1996.

**38:** 29.9 mg, 70% yield, colorless oil; ^1^H NMR (500 MHz, CDCl_3_) δ 7.61 (d, *J* = 8.2 Hz, 2H), 7.31 (d, *J* = 8.0 Hz, 2H), 4.20 – 4.08 (m, 2H), 3.26 – 3.14 (m, 3H), 2.81 – 2.26 (m, 11H), 1.39 – 1.19 (m, 13H), 0.84 (t, *J* = 6.9 Hz, 3H); ^13^C NMR (125 MHz, CDCl_3_) δ 172.3, 143.7, 132.6, 129.8, 128.0, 60.8, 54.4, 53.8, 50.0, 47.3, 46.0, 31.9, 30.3, 29.2, 27.4, 27.0, 22.7, 21.6, 14.3, 14.2; IR (neat): 1730, 1458, 1352, 1167, 1111, 1091, 1031, 950, 664, 549 cm^-1^; HRMS (ESI) Calculated for C_22_H_37_N_2_O_4_S [M+H]^+^ 425.2469, found 425.2470.

**39:** 18.4 mg, 50% yield, colorless oil; ^1^H NMR (500 MHz, CDCl_3_) δ 7.62 (d, *J* = 8.2 Hz, 2H), 7.33 (d, *J* = 8.3 Hz, 2H), 4.15 (q, *J* = 7.2 Hz, 2H), 3.53 – 3.52 (m, 2H), 3.36 – 3.26 (m 3H), 2.83– 2.54 (m, 8H), 2.48 – 2.40 (m, 4H), 1.27 (t, *J* = 7.1 Hz, 3H); ^13^C NMR (125 MHz, CDCl_3_) δ 172.1, 144.0, 132.5, 129.9, 127.9, 61.0, 58.1, 55.1, 54.6, 49.2, 46.4, 45.4, 31.2, 21.7, 14.3; IR (neat): 3446, 1726, 1348, 1164, 1090, 1030, 951, 663, 548 cm^-1^; HRMS (ESI) Calculated for C_17_H_27_N_2_O_5_S [M+H]^+^ 371.1635, found 371.1630.

**40:** 31.5 mg, 67% yield, colorless oil; ^1^H NMR (500 MHz, CDCl_3_) δ 7.64 (d, *J* = 8.1 Hz, 2H), 7.35 (d, *J* = 8.1 Hz, 2H), 4.78 (s, 1H), 4.16 (q, *J* = 7.1 Hz, 2H), 3.34 – 3.07 (m, 5H), 2.84 – 2.42 (m, 11H), 1.40 (s, 9H), 1.28 (t, *J* = 7.1 Hz, 3H); ^13^C NMR (125 MHz, CDCl_3_) δ 172.1, 156.0, 143.9, 132.6, 129.8, 127.9, 79.3, 60.9, 54.8, 53.0, 49.3, 46.4, 45.4, 37.5, 31.1, 28.5, 21.7, 14.3; IR (neat): 3404, 1712, 1508, 1365, 1351, 1250, 1167, 957, 662, 550 cm^-1^; HRMS (ESI) Calculated for C_22_H_36_N_3_O_6_S [M+H]^+^ 470.2319, found 470.2318.

**41:** 23.8 mg, 63% yield, colorless oil; ^1^H NMR (500 MHz, CDCl_3_) δ 7.62 (d, *J* = 8.2 Hz, 2H), 7.31 (d, *J* = 8.0 Hz, 2H), 4.14 (q, *J* = 7.1 Hz, 2H), 3.34 – 3.10 (m, 3H), 2.89 – 2.55 (m, 5H), 2.49 – 2.39 (m, 4H), 2.30 – 2.29 (m, 2H), 1.26 (t, *J* = 7.1 Hz, 3H), 0.79 – 0.67 (m, 1H), 0.50 – 0.43 (m, 2H), 0.08 – 0.03 (m, 2H); ^13^C NMR (125 MHz, CDCl_3_) δ 172.2, 143.8, 132.5, 129.8, 128.0, 60.8, 58.7, 54.4, 50.0, 47.6, 46.0, 30.6, 21.6, 14.3, 8.7, 4.0, 3.9; IR (neat): 1729, 1350, 1167, 1111, 1020, 670, 549 cm^-1^; HRMS (ESI) Calculated for C_19_H_29_N_2_O_4_S [M+H]^+^ 381.1843, found 381.1836.

**42:** 28.8 mg, 65% yield, colorless oil; ^1^H NMR (500 MHz, CDCl_3_) δ 7.62 (d, *J* = 8.2 Hz, 2H), 7.32 (d, *J* = 8.1 Hz, 2H), 7.24 (t, *J* = 7.5 Hz, 2H), 7.17 – 7.09 (m, 3H), 4.14 (q, *J* = 7.1 Hz, 2H), 3.30 – 3.18 (m, 3H), 2.78 – 2.76 (m, 1H), 2.72 – 2.34 (m, 13H), 1.74 – 1.68 (m, 2H), 1.27 (t, *J* = 7.1 Hz, 3H); ^13^C NMR (125 MHz, CDCl_3_) δ 172.3, 143.8, 141.9, 132.6, 129.8, 128.4, 127.9, 125.9, 60.8, 54.3, 53.0, 49.9, 47.1, 45.9, 33.4, 30.2, 28.5, 21.7, 14.3; IR (neat): 1729, 1454, 1351, 1167, 1091, 1029, 961, 749, 665, 549 cm^-1^; HRMS (ESI) Calculated for C_24_H_33_N_2_O_4_S [M+H]^+^ 445.2156, found 445.2149.

**43:** 16.2 mg, 40% yield, colorless oil; ^1^H NMR (500 MHz, CDCl_3_) δ 7.61 (d, *J* = 8.1 Hz, 2H), 7.31 (d, *J* = 8.0 Hz, 2H), 4.15 (qd, *J* = 7.1, 2.1 Hz, 2H), 3.46 – 3.45 (m, 1H), 3.22 – 3.09 (m, 2H), 2.83 – 2.56 (m, 5H), 2.46 – 2.31 (m, 5H), 1.80 – 1.56 (5H), 1.32 – 1.01 (m, 9H); ^13^C NMR (125 MHz, CDCl_3_) δ 172.4, 143.7, 132.5, 129.8, 128.0, 60.8, 58.2, 51.5, 50.5, 46.6, 43.1, 31.5, 31.2, 28.5, 26.2, 25.6, 25.5, 21.7, 14.3; IR (neat): 1729, 1350, 1168, 974, 950, 758, 549 cm^-1^; HRMS (ESI) Calculated for C_21_H_33_N_2_O_4_S [M+H]^+^ 409.2156, found 409.2155.

**44:** 30.9 mg, 65% yield, colorless oil; ^1^H NMR (500 MHz, CDCl_3_) δ 7.61 (d, *J* = 8.2 Hz, 2H), 7.31 (d, *J* = 8.0 Hz, 2H), 5.88 – 5.75 (m, 1H), 5.05 – 4.85 (m, 2H), 4.14 (q, *J* = 7.1 Hz, 2H), 3.26 – 3.12 (m, 3H), 2.81 – 2.27 (m, 11H), 2.03 – 1.99 (m, 2H), 1.37 – 1.22 (m, 18H); ^13^C NMR (125 MHz, CDCl_3_) δ 172.3, 143.7, 139.3, 132.6, 129.8, 128.0, 114.2, 60.8, 54.4, 53.8, 50.0, 47.3, 46.0, 33.9, 30.3, 29.6, 29.6, 29.5, 29.2, 29.0, 27.4, 27.0, 21.6, 14.3; IR (neat): 1731, 1458, 1353, 1305, 1168, 1154, 1091, 953, 665, 549 cm^-1^; HRMS (ESI) Calculated for C_26_H_33_N_2_O_4_S [M+H]^+^ 479.2938, found 479.2943.

**45:** 27.3 mg, 58% yield, colorless oil; ^1^H NMR (500 MHz, CDCl_3_) δ 9.06 – 9.05 (m, 1H), 8.46 – 8.44 (m, 1H), 8.24 – 8.22 (m, 1H), 8.07 – 7.98 (m, 1H), 7.60 (t, *J* = 7.8 Hz, 1H), 7.53 – 7.51 (m, 1H), 7.24 – 7.12 (m, 2H), 6.93 (t, *J* = 8.4 Hz, 2H), 4.11 (q, *J* = 7.0 Hz, 2H), 3.77 – 3.20 (s, 7H), 2.74 – 2.49 (m, 3H), 2.38 – 2.36 (m, 1H), 1.24 (t, *J* = 7.1 Hz, 3H); ^13^C NMR (125 MHz, CDCl_3_) δ 172.2, 162.1 (d, *J* = 245.2 Hz), 151.4, 144.4, 136.7, 136.5, 134.0, 133.6, 133.3, 130.3 (d, *J* = 7.3 Hz), 129.1, 125.6, 122.2, 115.2 (d, *J* = 21.2 Hz), 60.8, 57.6, 55.4, 50.2, 47.6, 46.2, 31.0, 14.3; ^19^F NMR (470 MHz, CDCl_3_) δ -115.67; IR (neat): 1728, 1508, 1337, 1219, 1162, 1146, 966, 833, 791, 584 cm^-1^; HRMS (ESI) Calculated for C_24_H_27_FN_3_O_4_S [M+H]^+^ 472.1701, found 472.1700.

**46:** 30.2 mg, 57% yield, colorless oil; ^1^H NMR (500 MHz, CDCl_3_) δ 7.80 (d, *J* = 8.4 Hz, 2H), 7.70 (d, *J* = 8.4 Hz, 2H), 7.54 (d, *J* = 8.5 Hz, 2H), 7.46 (d, *J* = 8.5 Hz, 2H), 7.18 (dd, *J* = 8.3, 5.6 Hz, 2H), 6.94 (t, *J* = 8.6 Hz, 2H), 4.15 (q, *J* = 7.1 Hz, 2H), 3.64 (d, *J* = 13.3 Hz, 1H), 3.42 (d, *J* = 13.3 Hz, 1H), 3.36 – 3.15 (m, 3H), 2.97 – 2.95 (m, 1H), 2.86 – 2.55 (m, 4H), 2.48 – 2.36 (m, 1H), 1.26 (t, *J* = 7.1 Hz, 3H); ^13^C NMR (125 MHz, CDCl_3_) δ 172.0, 162.2 (d, *J* = 245.4 Hz), 144.6, 137.8, 135.0, 134.7, 133.7, 130.3 (d, *J* = 7.8 Hz), 129.4, 128.7, 128.5, 127.7, 115.3 (d, *J* = 21.3 Hz) 60.9, 57.5, 55.1, 50.1, 46.9, 46.0, 31.1, 14.3; ^19^F NMR (470 MHz, CDCl_3_) δ -115.37; IR (neat): 1728, 1508, 1351, 1220, 1168, 1093, 960, 817, 782, 599 cm^-1^; HRMS (ESI) Calculated for C_27_H_29_ClFN_2_O_4_S [M+H]^+^ 531.1515, found 531.1530.

**47:** 23.2 mg, 65% yield, colorless oil; ^1^H NMR (500 MHz, CDCl_3_) δ 7.77 – 7.71 (m, 2H), 7.62 – 7.57 (m, 1H), 7.52 (t, *J* = 7.5 Hz, 2H), 4.14 (q, *J* = 7.1 Hz, 2H), 3.29 – 3.17 (m, 3H), 2.86 – 2.29 (m, 8H), 1.37 – 1.22 (m, 7H), 0.86 (t, *J* = 7.1 Hz, 3H); ^13^C NMR (125 MHz, CDCl_3_) δ 172.3, 135.6, 132.9, 129.2, 127.9, 60.8, 54.3, 53.5, 50.0, 47.3, 46.0, 30.3, 29.1, 20.6, 14.3, 14.0; IR (neat): 1730, 1447, 1353, 1172, 1102, 951, 753, 692, 578 cm^-1^; HRMS (ESI) Calculated for C_18_H_29_N_2_O_4_S [M+H]^+^ 369.1843, found 369.1842.

**48:** 28.4 mg, 69% yield, colorless oil; ^1^H NMR (500 MHz, CDCl_3_) δ 8.37 (d, *J* = 8.8 Hz, 2H), 7.93 (d, *J* = 8.8 Hz, 2H), 4.15 (q, *J* = 7.1 Hz, 2H), 3.37 – 3.15 (m, 3H), 2.94 – 2.30 (m, 8H), 1.45 – 1.17 (m, 7H), 0.85 (t, *J* = 7.3 Hz, 3H); ^13^C NMR (125 MHz, CDCl_3_) δ 172.1, 150.3, 141.9, 129.0, 124.4, 60.9, 54.2, 53.4, 49.8, 47.0, 46.0, 30.0, 29.1, 20.5, 14.3, 14.0; IR (neat): 1728, 1530, 1350, 1310, 1171, 960, 855, 759, 600 cm^-1^; HRMS (ESI) Calculated for C_18_H_28_N_3_O_6_S [M+H]^+^ 414.1693, found 414.1698.

**49:** 64 mg (0.2 mmol scale), 69%, light-yellow oil; ^1^H NMR (400 MHz, CDCl_3_) δ 8.38 (d, *J* = 8.0 Hz, 2H), 7.93 (d, *J* = 8.1 Hz, 2H), 7.23 – 7.10 (m, 2H), 6.96 – 6.92 (m, 2H), 4.15 (q, *J* = 7.1 Hz, 2H), 3.63 (d, *J* = 13.3 Hz, 1H), 3.43 (d, *J* = 13.3 Hz, 1H), 3.33 – 3.20 (m, 3H), 2.96 – 2.93 (m, 1H), 2.80 – 2.56 (m, 4H), 2.42 – 2.40 (m, 1H), 1.26 (t, *J* = 7.0 Hz, 3H); ^13^C NMR (100 MHz, CDCl_3_) δ 171.8, 162.2 (d, *J* = 246.0 Hz), 150.4, 142.0, 133.5, 130.2 (d, *J* = 7.4 Hz), 129.0, 124.5, 115.4 (d, *J* = 21.1 Hz), 100.1, 61.0, 57.4, 54.8, 49.9, 46.7, 45.9, 30.8, 14.3; IR(neat): 1726, 1529, 1349, 1169, 854, 731, 624, 461; HRMS (ESI) Calculated for C_21_H_24_FN_3_O_6_S [M+H]^+^ 466.1443 found 466.1444.

**50:** 63 mg (0.2 mmol scale), 65% yield, light-yellow oil;^1^ H NMR (400 MHz, CDCl_3_) δ 8.38 (d, *J* = 8.7 Hz, 2H), 7.92 (d, *J* = 8.7 Hz, 2H), 7.22 (d, *J* = 8.3 Hz, 2H), 7.14 (d, *J* = 8.3 Hz, 2H), 4.14 (q, *J* = 7.1 Hz, 2H), 3.63 (d, *J* = 13.5 Hz, 1H), 3.43 (d, *J* = 13.5 Hz, 1H), 3.36 – 3.19 (m, 3H), 2.96 – 2.94 (m, 1H), 2.83 – 2.54 (m, 4H), 2.49 – 2.35 (m, 1H), 1.25 (t, *J* = 7.1 Hz, 3H); ^13^C NMR (100 MHz, CDCl_3_) δ 171.8, 150.4, 142.0, 136.4, 133.4, 130.0, 129.0, 128.6, 124.5, 61.0, 57.4, 54.9, 49.8, 46.7, 45.9, 30.8, 14.3; IR(neat): 1727, 1532, 1350, 1171, 907, 727, 687, 599; HRMS (ESI) Calculated for C_21_H_24_ClN_3_O_6_S [M+H]^+^ 482.1147 found 482.1150.

**51:** 28.4 mg, 67% yield, colorless oil; ^1^H NMR (500 MHz, CDCl_3_) δ 7.63 (d, *J* = 4.9 Hz, 1H), 7.55 – 7.49 (m, 1H), 7.24 – 7.12 (m, 3H), 6.96 (t, *J* = 8.4 Hz, 2H), 4.15 (q, *J* = 7.1 Hz, 2H), 3.65 (d, *J* = 13.2 Hz, 1H), 3.43 (d, *J* = 13.2 Hz, 1H), 3.45 – 3.20 (m, 3H), 2.98 – 2.42 (m, 6H), 1.26 (t, *J* = 7.1 Hz, 3H); ^13^C NMR (125 MHz, CDCl_3_) δ 172.0, 162.2 (d, *J* = 245.4 Hz) 136.1, 133.7, 132.7, 132.4, 130.3 (d, *J* = 7.7 Hz), 127.9, 115.34 (d, *J* = 21.4 Hz) 61.0, 57.5, 55.0, 50.0, 46.8, 46.0, 31.2, 14.3; ^19^F NMR (470 MHz, CDCl_3_) δ -115.39; IR (neat): 1727, 1508, 1355, 1220, 1162, 959, 580 cm^-1^; HRMS (ESI) Calculated for C_19_H_24_FN_2_O_4_S_2_ [M+H]^+^ 427.1156, found 427.1157.

**52:** 29.3 mg, 61% yield, colorless oil; ^1^H NMR (500 MHz, CDCl_3_) δ 7.34 – 7.16 (m, 4H), 7.03 – 6.91 (m, 3H), 4.45 – 4.26 (m, 4H), 4.17 (q, *J* = 7.1 Hz, 2H), 3.66 (d, *J* = 13.3 Hz, 1H), 3.44 (d, *J* = 13.3 Hz, 1H), 3.24 – 3.15 (m, 3H), 2.93 – 2.92 (m, 1H), 2.80 – 2.57 (m, 4H), 2.47 – 2.35 (m, 1H), 1.28 (t, *J* = 7.1 Hz, 3H); ^13^C NMR (125 MHz, CDCl_3_) δ 171.9, 162.0 (d, *J* = 245.2 Hz), 147.6, 143.6, 133.7, 130.2 (d, *J* = 7.9 Hz), 128.0, 121.5, 117.7, 117.3, 115.2 (d, *J* = 21.3 Hz), 64.5, 64.2, 60.8, 57.3, 55.0, 50.0, 46.8, 45.8, 31.1, 14.2; ^19^F NMR (470 MHz, CDCl_3_) δ -115.49; IR (neat): 1729, 1508, 1495, 1287, 1254, 1161, 1063, 961, 567 cm^-1^; HRMS (ESI) Calculated for C_23_H_28_FN_2_O_6_S [M+H]^+^ 479.1647, found 479.1642.

**53:** 27.3 mg, 74% yield, colorless oil; ^1^H NMR (500 MHz, CDCl_3_) δ 7.61 (d, *J* = 8.2 Hz, 2H), 7.31 (d, *J* = 8.2 Hz, 2H), 3.69 (s, 3H), 3.24 – 3.19 (m, 3H), 2.83 – 2.28 (m, 12H), 1.42 – 1.31 (m, 2H), 1.27 – 2.00 (m, 2H), 0.85 (t, *J* = 7.3 Hz, 3H); ^13^C NMR (125 MHz, CDCl_3_) δ 172.8, 143.8, 132.5, 129.8, 128.0, 54.3, 53.5, 52.0, 49.9, 47.2, 45.9, 29.9, 29.1, 21.6, 20.5, 14.0; IR (neat): 1732, 1350, 1166, 1108, 950, 815, 754, 664, 549 cm^-1^; HRMS (ESI) Calculated for C_19_H_29_N_2_O_4_S [M+H]^+^ 369.1843, found 369.1834.

**54:** 33.8 mg, 76% yield, colorless oil; ^1^H NMR (500 MHz, CDCl_3_) δ 7.60 (d, *J* = 8.2 Hz, 2H), 7.47 – 7.31 (m, 5H), 7.30 (d, *J* = 8.0 Hz, 2H), 5.16 (d, *J* = 12.3 Hz, 1H), 5.12 (d, *J* = 12.3 Hz, 1H), 3.21 – 3.20 (m, 3H), 2.88 – 2.20 (m, 11H), 1.43 – 1.10 (m, 4H), 0.84 (t, *J* = 7.3 Hz, 3H); ^13^C NMR (125 MHz, CDCl_3_) δ 172.2, 143.7, 135.9, 132.6, 129.8, 128.7, 128.4, 127.9, 66.7, 54.4, 53.5, 49.9, 47.3, 45.9, 30.4, 29.1, 21.6, 20.5, 14.0; IR (neat): 1731, 1350, 1167, 1107, 953, 751, 737, 664, 549 cm^-1^; HRMS (ESI) Calculated for C_24_H_33_N_2_O_4_S [M+H]^+^ 445.2156, found 445.2169.

**55:** 20.5 mg, 50% yield, colorless oil; ^1^H NMR (500 MHz, CDCl_3_) δ 7.61 (d, *J* = 8.2 Hz, 2H), 7.31 (d, *J* = 8.0 Hz, 2H), 3.27 – 3.06 (m, 3H), 2.86 – 2.60 (m, 3H), 2.55 – 2.26 (m, 8H), 1.46 (s, 9H), 1.40 – 1.22 (m, 4H), 0.86 (t, *J* = 7.3 Hz, 3H); ^13^C NMR (125 MHz, CDCl_3_) δ 171.7, 143.7, 132.6, 129.8, 128.0, 81.1, 54.6, 53.5, 50.0, 47.4, 46.1, 31.7, 29.2, 28.2, 21.6, 20.6, 14.1; IR (neat): 1724, 1366, 1352, 1169, 1150, 951, 664, 550 cm^-1^; HRMS (ESI) Calculated for C_21_H_35_N_2_O_4_S [M+H]^+^ 411.2312, found 411.2316.

**56:** 40.6 mg, 85% yield, colorless oil; ^1^H NMR (500 MHz, CDCl_3_) δ 7.63 (d, *J* = 8.2 Hz, 2H), 7.31 (d, *J* = 8.1 Hz, 2H), 7.27 (t, *J* = 6.7 Hz, 1H), 6.82 – 6.76 (m, 1H), 6.71 – 6.68 (m, 2H), 3.80 (s, 3H), 3.36 – 3.33 (m, 3H), 3.00 – 2.67 (m, 5H), 2.62 – 2.34 (m, 6H), 1.48 – 1.22 (m, 4H), 0.87 (t, *J* = 7.3 Hz, 3H); ^13^C NMR (125 MHz, CDCl_3_) δ 170.9, 160.6, 151.6, 143.8, 132.6, 129.9, 129.8, 127.9, 113.9, 112.0, 107.6, 55.6, 54.4, 53.6, 49.8, 47.0, 45.9, 30.1, 29.2, 21.6, 20.5, 14.0; IR (neat): 1752, 1609, 1490, 1167, 1139, 1041, 952, 664, 549 cm^-1^; HRMS (ESI) Calculated for C_24_H_33_N_2_O_5_S [M+H]^+^ 461.2105, found 461.2106.

**57:** 38.6 mg, 81% yield, colorless oil; ^1^H NMR (500 MHz, CDCl_3_) δ 7.62 (d, *J* = 8.2 Hz, 2H), 7.36 – 7.27 (m, 3H), 7.01 – 6.89 (m, 3H), 5.15 (d, *J* = 12.3 Hz, 1H), 5.11 (d, *J* = 12.3 Hz, 1H), 3.85 (s, 3H), 3.25 (s, 3H), 2.89 – 2.28 (m, 11H), 1.43 – 1.16 (m, 4H), 0.86 (t, *J* = 7.3 Hz, 3H); ^13^C NMR (125 MHz, CDCl_3_) δ 172.2, 159.9, 143.8, 137.3, 132.6, 129.8, 127.9, 120.6, 114.1, 113.7, 66.6, 55.4, 54.4, 53.5, 49.9, 47.2, 45.9, 30.3, 29.1, 21.6, 20.5, 14.0; IR (neat): 1731, 1598, 1457, 1350, 1268, 1168, 955, 664, 549 cm^-1^; HRMS (ESI) Calculated for C_25_H_35_N_2_O_5_S [M+H]^+^ 475.2261, found 475.2262.

**58:** 22.1 mg, 55% yield, colorless oil; ^1^H NMR (500 MHz, CDCl_3_) δ 7.61 – 7.56 (m, 3H), 7.33 – 7.27 (m, 3H), 6.57 – 6.56 (m, 1H), 3.55 – 3.39 (m, 2H), 3.32 – 3.21 (m, 2H), 2.93 – 2.89 (m, 1H), 2.76 – 2.54 (m, 4H), 2.41 (s, 3H), 2.38 (t, *J* = 7.4 Hz, 2H), 1.44 – 1.19 (m, 2H), 0.84 (t, *J* = 7.3 Hz, 3H); ^13^C NMR (125 MHz, CDCl_3_) δ 188.1, 152.8, 146.9, 143.8, 132.4, 129.8, 127.9, 118.1, 112.6, 53.8, 53.3, 50.2, 46.9, 46.2, 32.8, 29.3, 21.6, 20.5, 14.0; IR (neat): 1670, 1467, 1167, 950, 763, 671, 549 cm^-1^; HRMS (ESI) Calculated for C_21_H_29_N_2_O_4_S [M+H]^+^ 405.1843, found 405.1842.

**59:** 22.2 mg, 54% yield, colorless oil; ^1^H NMR (500 MHz, CDCl_3_) δ 7.99 (d, *J* = 7.6 Hz, 2H), 7.59 (t, *J* = 8.7 Hz, 3H), 7.49 (t, *J* = 7.7 Hz, 2H), 7.29 (d, *J* = 8.1 Hz, 2H), 3.62 – 3.26 (m, 4H), 3.06 – 3.02 (m, 1H), 2.79 – 2.55 (m, 4H), 2.50 – 2.31 (m, 5H), 1.40 – 1.32 (m, 2H), 1.32 – 1.17 (m, 2H), 0.84 (t, *J* = 7.3 Hz, 3H); ^13^C NMR (125 MHz, CDCl_3_) δ 199.2, 143.8, 137.2, 133.4, 132.5, 129.8, 128.8, 128.3, 128.0, 53.8, 53.1, 50.3, 47.1, 46.2, 32.5, 29.4, 21.6, 20.6, 14.1; IR (neat): 1681, 1449, 1350, 1167, 950, 756, 670, 549 cm^-1^; HRMS (ESI) Calculated for C_23_H_31_N_2_O_3_S [M+H]^+^ 415.2050, found 415.2047.

**60:** 21.1 mg, 47% yield, colorless oil; ^1^H NMR (500 MHz, CDCl_3_) δ 7.94 (d, *J* = 8.6 Hz, 2H), 7.58 (d, *J* = 8.2 Hz, 2H), 7.46 (d, *J* = 8.6 Hz, 2H), 7.29 (d, *J* = 8.0 Hz, 2H), 3.61 – 3.28 (m, 4H), 2.96 (d, *J* = 15.8 Hz, 1H), 2.76 – 2.54 (m, 4H), 2.40 (s, 3H), 2.36 (t, *J* = 7.4 Hz, 2H), 1.41 – 1.32 (m, 2H), 1.29 – 1.19 (m, 2H), 0.84 (t, *J* = 7.3 Hz, 3H); ^13^C NMR (125 MHz, CDCl_3_) δ 198.1, 143.9, 139.9, 135.5, 132.3, 129.8, 129.1, 127.9, 53.8, 53.0, 50.2, 46.9, 46.1, 32.1, 29.4, 21.7, 20.5, 14.1; IR (neat): 1682, 1599, 1350, 1166, 1097, 995, 948, 815, 752, 665, 549 cm^-1^; HRMS (ESI) Calculated for C_23_H_30_ClN_2_O_3_S [M+H]^+^ 449.1660, found 449.1672.

**61:** 24.2 mg, 56% yield, colorless oil; ^1^H NMR (500 MHz, CDCl_3_) δ 8.07 – 8.00 (m, 2H), 7.58 (d, *J* = 8.2 Hz, 2H), 7.29 (d, *J* = 8.0 Hz, 2H), 7.15 (t, *J* = 8.6 Hz, 2H), 3.66 – 3.24 (m, 4H), 2.98 – 2.94 (m, 1H), 2.80 – 2.52 (m, 4H), 2.41 (s, 3H), 2.37 (t, *J* = 7.4 Hz, 2H), 1.43 – 1.30 (m, 2H), 1.27 – 1.20 (m, 2H), 0.84 (t, *J* = 7.3 Hz, 3H); ^13^C NMR (125 MHz, CDCl_3_) δ 197.7, 166.0 (d, *J* = 255.3 Hz), 143.8, 133.6, 132.3, 131.0 (d, *J* = 9.4 Hz), 129.8, 127.9, 115.9 (d, *J* = 21.8 Hz), 53.84, 53.1, 50.2, 46.9, 46.2, 32.0, 29.4, 21.7, 20.5, 14.1; ^19^F NMR (470 MHz, CDCl_3_) δ -104.84; IR (neat): 1682, 1597, 1350, 1232, 1168, 950, 670, 549 cm^-1^; HRMS (ESI) Calculated for C_23_H_30_FN_2_O_3_S [M+H]^+^ 433.1956, found 433.1956.

**62:** 25.7 mg, 58% yield, colorless oil; ^1^H NMR (500 MHz, CDCl_3_) δ 8.01 – 7.95 (m, 2H), 7.58 (d, *J* = 8.2 Hz, 2H), 7.28 (d, *J* = 8.0 Hz, 2H), 6.97 – 6.93 (m, 2H), 3.87 (s, 3H), 3.66 – 3.29 (m, 4H), 2.97 – 2.93 (m, 1H), 2.76 – 2.56 (m, 4H), 2.40 (s, 3H), 2.37 (t, *J* = 7.5 Hz, 2H), 1.44 – 1.20 (m, 4H), 0.83 (t, *J* = 7.3 Hz, 3H); ^13^C NMR (125 MHz, CDCl_3_) δ 197.7, 163.8, 143.7, 132.4, 130.6, 130.3, 129.7, 127.9, 113.9, 55.6, 53.8, 53.28, 50.4, 47.1, 46.2, 32.1, 29.3, 21.6, 20.6, 14.0; IR (neat): 1672, 1599, 1350, 1259, 1168, 950, 671, 549 cm^-1^; HRMS (ESI) Calculated for C_24_H_33_N_2_O_4_S [M+H]^+^ 445.2156, found 445.2140.

**63:** 25.7 mg, 53% yield, colorless oil; ^1^H NMR (500 MHz, CDCl_3_) δ 8.10 (d, *J* = 8.2 Hz, 2H), 7.75 (d, *J* = 8.3 Hz, 2H), 7.59 (d, *J* = 8.2 Hz, 2H), 7.29 (d, *J* = 8.1 Hz, 2H), 3.61–3.33 (m, 4H), 3.07 – 2.97 (m, 1H), 2.77 – 2.54 (m, 4H), 2.41 (s, 3H), 2.37 (t, *J* = 7.4 Hz, 2H), 1.39 – 1.22 (m, 2H), 0.84 (t, *J* = 7.3 Hz, 3H); ^13^C NMR (125 MHz, CDCl_3_) δ 198.5, 143.9, 139.9, 134.7 (q, *J* = 32.5 Hz),, 132.5, 129.8, 128.7, 125.9, 125.8, 123.72 (q, *J* = 272.6 Hz), 53.9, 53.1, 50.1, 46.9, 46.1, 32.5, 29.4, 21.6, 20.5, 14.0; ^19^F NMR (470 MHz, CDCl_3_) δ -63.10; IR (neat): 1689, 1350, 1166, 1128, 1065, 950, 666, 549 cm^-1^; HRMS (ESI) Calculated for C_24_H_30_F_3_N_2_O_3_S [M+H]^+^ 483.1924, found 483.1924.

**64:** 14.6 mg, 33% yield, colorless oil; ^1^H NMR (500 MHz, CDCl_3_) δ 7.62 (d, *J* = 8.3 Hz, 2H), 7.35 – 7.29 (m, 4H), 7.25 – 7.19 (m, 3H), 3.49 – 3.34 (m, 2H), 3.24 – 3.22 (m, 1H), 2.98 – 2.77 (m, 5H), 2.64 – 2.52 (m, 4H), 2.49 – 2.43 (m, 4H), 2.33 – 2.25 (m, 2H), 1.37 – 1.22 (m, 2H), 0.87 (t, *J* = 7.3 Hz, 3H); ^13^C NMR (125 MHz, CDCl_3_) δ 209.3, 143.8, 141.0, 132.6, 129.8, 128.6, 128.5, 127.9 126.2, 53.7, 52.7, 50.0, 46.83, 46.06, 45.69, 36.43, 29.82, 29.37, 21.65, 20.54, 14.06; IR (neat): 1710, 1455, 1350, 1167, 1091, 950, 748, 661, 549 cm^-1^; HRMS (ESI) Calculated for C_25_H_35_N_2_O_3_S [M+H]^+^ 443.2363, found 443.2373.

**65:** 28.1 mg, 84% yield, colorless oil; ^1^H NMR (500 MHz, CDCl_3_) δ 7.63 (d, *J* = 8.2 Hz, 2H), 7.33 (d, *J* = 8.0 Hz, 2H), 3.22 – 2.86 (m, 5H), 2.79 – 2.63 (m, 2H), 2.58 – 2.31 (m, 7H), 1.42 – 1.34 (m, 2H), 1.31 – 1.24 (m, 2H), 0.87 (t, *J* = 7.3 Hz, 3H); ^13^C NMR (125 MHz, CDCl_3_) δ 144.2, 132.3, 130.0, 127.9, 117.9, 55.0, 53.5, 49.5, 47.3, 45.9, 28.8, 21.7, 20.4, 15.4, 14.0; IR (neat): 1350, 1167, 1090, 952, 816, 751, 662, 549 cm^-1^; HRMS (ESI) Calculated for C_17_H_26_N_3_O_2_S [M+H]^+^ 336.1740, found 336.1741.

**66:** 36.6 mg, 81% yield, colorless oil; ^1^H NMR (500 MHz, CDCl_3_) δ 7.96 (d, *J* = 7.6 Hz, 2H), 7.68 (t, *J* = 7.4 Hz, 1H), 7.60 (t, *J* = 7.3 Hz, 4H), 7.31 (d, *J* = 8.1 Hz, 2H), 3.66 – 3.48 (m, 4H), 3.19 – 3.09 (m, 1H), 2.56 – 2.24 (m, 9H), 1.34 – 1.20 (m, 4H), 0.83 (t, *J* = 7.3 Hz, 3H); ^13^C NMR (125 MHz, CDCl_3_) δ 144.0, 139.9, 133.9, 132.7, 129.9, 129.6, 128.0, 127.8, 53.6, 51.6, 50.4, 50.2, 46.5, 45.9, 29.1, 21.7, 20.4, 14.0; IR (neat): 1350, 1303, 1168, 1147, 1085, 946, 750, 663, 548 cm^-1^; HRMS (ESI) Calculated for C_22_H_31_N_2_O_4_S_2_ [M+H]^+^ 451.1720, found 451.1721.

**67:** 28.0 mg, 55% yield, colorless oil; ^1^H NMR (500 MHz, CDCl_3_) δ 7.93 – 7.72 (m, 4H), 7.64 – 7.38 (m, 8H), 7.31 – 7.21 (m, 2H), 3.52 – 3.26 (m, 2H), 2.79 – 2.73 (m, 1H), 2.65 – 2.41 (m, 4H), 2.39 (s, 3H), 2.34 – 2.13 (m, 3H), 1.19 – 1.05 (m, 4H), 0.75 (t, *J* = 7.0 Hz, 3H); ^13^C NMR (125 MHz, CDCl_3_) δ 143.6, 133.4 (d, *J* = 58.2 Hz), 132.6 (d, *J* = 58.1 Hz) 132.4, 131.9, 131.8, 131.0 (d, *J* = 9.5 Hz), 130.8 (d, *J* = 9.1 Hz), 129.6, 128.8 (d, *J* = 11.8 Hz), 128.7 (d, *J* = 11.6 Hz), 127.7, 53.5, 51.0, 50.8, 45.9, 29.5 (d, *J* = 56.2 Hz), 29.1, 23.4 (d, *J* = 69.6 Hz) 21.5, 20.3, 13.9; ^31^P NMR (202 MHz, CDCl_3_) δ 30.82; IR (neat): 1350, 1336, 1184, 1167, 1119, 1103, 945, 732, 696, 661, 535 cm^-1^; HRMS (ESI) Calculated for C_28_H_36_N_2_O_3_PS [M+H]^+^ 511.2179, found 511.2178.

**68:** 26.1 mg, 58% yield, colorless oil; ^1^H NMR (500 MHz, CDCl_3_) δ 7.61 (d, *J* = 8.1 Hz, 2H), 7.31 (d, *J* = 8.0 Hz, 2H), 4.25 – 4.05 (m, 4H), 3.53 – 3.22 (m, 3H), 2.75 – 2.46 (m, 4H), 2.42 (s, 3H), 2.38 – 2.28 (m, 2H), 2.23 – 2.09 (m, 1H), 1.90 – 1.82 (m, 1H), 1.46 – 1.30 (m, 8H), 1.32 – 1.23 (m, 2H), 0.86 (t, *J* = 7.2 Hz, 3H); ^13^C NMR (125 MHz, CDCl_3_) δ 143.8, 132.5, 129.8, 128.0, 62.02 (d, *J* = 5.8 Hz), 61.83 (d, *J* = 6.3 Hz), 53.6, 52.2, 50.2, 46.4, 46.2, 29.1, 21.7, 20.6, 19.74 (d, *J* = 138.8 Hz), 16.58 (d, *J* = 6.0 Hz), 16.54 (d, *J* = 5.8 Hz), 14.1; ^31^P NMR (202 MHz, CDCl_3_) δ 30.64; IR (neat): 1351, 1246, 1169, 1054, 1028, 962, 663, 550 cm^-1^; HRMS (ESI) Calculated for C_20_H_36_N_2_O_5_PS [M+H]^+^ 447.2077, found 447.2077.

**69:** 19.0 mg, 43% yield, colorless oil; ^1^H NMR (500 MHz, CDCl_3_) δ 7.95 (d, *J* = 7.5 Hz, 2H), 7.73 – 7.52 (m, 5H), 7.32 (d, *J* = 7.9 Hz, 2H), 3.63 (d, *J* = 11.4 Hz, 1H), 3.58 – 3.47 (m, 3H), 3.22 – 2.18 (m, 1H), 2.51 (d, *J* = 11.5 Hz, 1H), 2.42 (s, 3H), 2.33 – 2.24 (m, 1H), 2.20 (s, 3H), 2.03 (t, *J* = 10.7 Hz, 1H), 1.54 – 1.49 (m, 1H), 1.24 – 1.15 (m, 1H), 0.79 (t, *J* = 7.5 Hz, 3H); ^13^C NMR (125 MHz, CDCl_3_) δ 144.0, 130.0, 133.9, 132.7, 129.9, 129.5, 128.0, 127.8, 56.2, 54.9, 50.9, 49.7, 48.9, 38.2, 23.3, 21.7, 9.4; IR (neat): 1447, 1341, 1305, 1168, 1149, 1087, 930, 816, 665, 597, 553 cm^-1^; HRMS (ESI) Calculated for C_21_H_29_N_2_O_4_S_2_ [M+H]^+^ 437.1563 found 437.1553.

**70:** 16.5 mg, 41% yield, foam solid; Single crystal was grown in refrigerator, using acetone and hexane as solvents (**CCDC Number: 1561430**); ^1^H NMR (500 MHz, CDCl_3_) δ 7.97 – 7.95 (m, 2H), 7.69 – 7.67 (m, 1H), 7.64 – 7.55 (m, 4H), 7.32 (d, *J* = 8.0 Hz, 2H), 3.69 (d, *J* = 11.4 Hz, 1H), 3.60 – 3.45 (m, 3H), 3.24 – 3.13 (m, 1H), 2.52 – 2.50 (m, 1H), 2.43 (s, 3H), 2.42 – 2.35 (m, 1H), 2.22 (s, 3H), 1.96 (t, *J* = 10.8 Hz, 1H), 0.92 (d, *J* = 6.2 Hz, 3H); ^13^C NMR (125 MHz, CDCl_3_) δ 144.0, 139.9, 134.0, 132.6, 129.9, 129.6, 128.0, 127.8, 54.7, 52.1, 51.0, 50.5, 50.2, 38.5, 21.7, 17.2; IR (neat): 1447, 1340, 1304, 1167, 1148, 1086, 814, 734, 664, 548 cm^-1^; HRMS (ESI) Calculated for C_20_H_27_N_2_O_4_S_2_ [M+H]^+^ 423.1407 found 423.1409.

**71:** 41.6 mg, 79% yield, foam solid; Single crystal was grown in the refrigerator, using the acetone and hexane as solvent (**CCDC Number: 1561427**); ^1^H NMR (500 MHz, CDCl_3_) δ 7.97 – 7.88 (m, 2H), 7.69 (t, *J* = 7.5 Hz, 1H), 7.65 – 7.52 (m, 4H), 7.25 (d, *J* = 7.7 Hz, 2H), 7.17 (t, *J* = 7.9 Hz, 1H), 6.79 – 6.70 (m, 2H), 6.66 (s, 1H), 4.09 (br s, 1H), 3.94 – 3.91 (m, 1H), 3.76 (s, 3H), 3.46 – 3.43 (m, 1H), 3.15 – 2.95 (m, 3H), 2.74 (dd, *J* = 13.0, 5.5 Hz, 1H), 2.59 – 2.50 (m, 1H), 2.42 (s, 3H), 2.35 (br s, 1H), 2.01 (br s, 1H), 1.98 (s, 3H); ^13^C NMR (125 MHz, CDCl_3_) δ 159.8, 143.5, 140.0 139.4, 137.6, 134.2, 129.9, 129.6, 128.2, 127.3, 121.8, 115.0, 112.0, 57.5, 56.9, 56.7, 55.3, 54.7, 45.4, 42.9, 35.5, 21.7; IR (neat): 1600, 1447, 1342, 1304, 1261, 1150, 1086, 969, 734, 672, 568 cm^-1^; HRMS (ESI) Calculated for C_27_H_33_N_2_O_5_S_2_ [M+H]^+^ 529.1825 found 529.1813.

**72:** 20.8 mg, 45% yield, colorless oil; ^1^H NMR (500 MHz, CDCl_3_) δ 7.62 (d, *J* = 7.7 Hz, 2H), 7.30 – 7.22 (m, 2H), 7.18 (t, *J* = 7.8 Hz, 1H), 6.76 – 6.69 (m, 3H), 4.17 (q, *J* = 6.9 Hz, 2H), 4.07 (s, 1H), 3.77 (s, 3H), 3.77 – 3.69 (m, 1H), 3.22 – 3.04 (m, 2H), 2.72 – 2.68 (m, 1H), 2.63 – 2.48 (m, 2H), 2.46 – 2.27 (m, 5H), 2.15 (s, 3H), 2.06 – 2.04 (m, 1H), 1.32 – 1.24 (m, 3H); ^13^C NMR (125 MHz, CDCl_3_) δ 171.2, 159.8, 143.3, 140.3, 138.0, 129.8, 129.6, 127.3, 121.9, 115.0, 112.0, 60.9, 58.8, 56.5, 55.3, 55.0, 45.2, 42.8, 36.9, 35.4, 21.6, 14.4; IR (neat): 1731, 1343, 1262, 1155, 1094, 975, 672, 547 cm^-1^; HRMS (ESI) Calculated for C_24_H_33_N_2_O_5_S [M+H]^+^ 461.2105 found 461.2105.

**73:** 43.4 mg, 91% yield, colorless oil; ^1^H NMR (500 MHz, CDCl_3_) δ 7.92 (d, *J* = 8.1 Hz, 2H), 7.69 – 7.57 (m, 5H), 7.30 (d, *J* = 8.1 Hz, 2H), 3.54 – 3.13 (m, 4H), 2.91 (s, 1H), 2.42 (br s, 3H), 2.41 – 2.22 (m, 3H), 2.07 – 2.01 (m, 1H), 1.27 – 1.09 (m, 7H), 0.81 (t, *J* = 7.0 Hz, 3H); ^13^C NMR (125 MHz, CDCl_3_) δ 143.5, 139.6, 136.3, 134.1, 129.9, 129.6, 128.1, 127.4, 56.0, 54.7, 53.9, 53.2, 50.7, 47.9, 28.5, 21.6, 20.4, 16.9, 14.0; IR (neat): 1447, 1340, 1305, 1149, 1086, 999, 671, 549 cm^-1^; HRMS (ESI) Calculated for C_23_H_33_N_2_O_4_S_2_ [M+H]^+^ 465.1876 found 465.1877.

**74:** 27.2 mg, 52% yield, colorless oil; ^1^H NMR (500 MHz, CDCl_3_) δ 7.93 (d, *J* = 7.4 Hz, 2H), 7.70 (t, *J* = 7.4 Hz, 1H), 7.64 – 7.52 (m, 4H), 7.33 – 7.23 (m, 7H), 4.56 (s, 1H), 3.60 – 3.12 (m, 6H), 2.45 (s, 3H), 2.42 – 2.26 (m, 2H), 2.04 – 2.00 (m, 1H), 1.24 – 1.08 (m, 4H), 0.81 (t, *J* = 7.0 Hz, 3H); ^13^C NMR (125 MHz, CDCl_3_) δ 143.6, 139.5, 138.7, 136.3, 134.1, 129.8, 129.6, 128.3, 128.1, 128.0, 127.8, 57.6, 55.2, 54.1, 53.6, 53.4, 48.0, 28.6, 21.7, 20.5, 14.0; IR (neat): 1447, 1342, 1304, 1158, 1149, 953, 734, 690, 572, 543 cm^-1^; HRMS (ESI) Calculated for C_28_H_35_N_2_O_4_S_2_ [M+H]^+^ 527.2033 found 527.2023.

**75:** 40.3 mg, 83% yield, colorless oil; ^1^H NMR (500 MHz, CDCl_3_) δ 7.93 – 7.85 (m, 2H), 7.70 – 7.67 (m, 3H), 7.60 – 7.58 (m, 2H), 7.41 (d, *J* = 7.3 Hz, 2H), 7.36 – 7.19 (m, 5H), 4.91 (s, 1H), 3.90 – 3.82 (m, 1H), 3.34 – 3.30 (m, 1H), 3.09 – 2.88 (m, 3H), 2.57 – 2.49 (m, 1H), 2.45 (s, 3H), 2.33 (dd, *J* = 12.5, 4.4 Hz, 1H), 2.01 (s, 3H); ^13^C NMR (125 MHz, CDCl_3_) δ 143.6, 139.4, 138.3, 137.4, 134.2, 129.9, 129.6, 128.4, 128.1, 128.0, 127.6, 127.5, 56.9, 56.2, 56.0, 55.7, 46.6, 42.7, 21.7; IR (neat): 1446, 1344, 1304, 1168, 1149, 1087, 689, 550 cm^-1^; HRMS (ESI) Calculated for C_25_H_29_N_2_O_4_S_2_ [M+H]^+^ 485.1563 found 485.1562.

**76:** 32.4 mg, 77% yield, colorless oil; ^1^H NMR (500 MHz, CDCl_3_) δ 7.69 (d, *J* = 8.2 Hz, 2H), 7.52 (d, *J* = 7.4 Hz, 2H), 7.33 – 7.19 (m, 5H), 5.03 (s, 1H), 4.14 (q, *J* = 7.1, 2H), 3.79 – 3.69 (m, 1H), 3.14 (dd, *J* = 12.3, 1.8 Hz, 1H), 3.02 (dd, *J* = 13.8, 10.9 Hz, 1H), 2.54 (dd, *J* = 15.4, 4.3 Hz, 1H), 2.44 (s, 3H), 2.39 – 2.37 (m, 2H), 2.25 – 2.19 (m, 1H), 2.18 (s, 3H), 1.24 (t, *J* = 7.1 Hz, 3H); ^13^C NMR (125 MHz, CDCl_3_) δ 171.2, 143.3, 138.8, 138.0, 129.8, 128.3, 128.3, 127.4, 127.3, 60.8, 58.1, 57.29, 55.4, 45.9, 42.9, 36.4, 21.6, 14.3; IR (neat): 1730, 1343, 1158, 1097, 972, 957, 692, 567, 541 cm^-1^; HRMS (ESI) Calculated for C_22_H_29_N_2_O_4_S [M+H]^+^ 417.1843 found 417.1846.

**77:** 26.6 mg, 53% yield, foam solid; Single crystal was grown in the refrigerator, using the acetone and hexane as solvent (**CCDC Number: 1561428**); ^1^H NMR (500 MHz, CDCl_3_) δ 8.00 (d, *J* = 7.6 Hz, 2H), 7.65 (d, *J* = 8.2 Hz, 3H), 7.58 (t, *J* = 7.6 Hz, 2H), 7.31 – 7.20 (m, 2H), 3.78 – 3.59 (m, 3H), 3.44 – 3.34 (m, 1H), 3.27 (t, *J* = 8.8 Hz, 1H), 3.06 (d, *J* = 14.4 Hz, 1H), 2.72 – 2.53 (m, 2H), 2.49 – 2.33 (m, 4H), 2.17 – 1.95 (m, 2H), 1.77 – 1.62 (m, 2H), 1.34 – 1.08 (m, 7H), 1.03 – 0.97 (m, 1H), 0.87 (t, *J* = 7.2 Hz, 3H); ^13^C NMR (125 MHz, CDCl_3_) δ 143.1, 140.0, 139.3, 133.8, 129.7, 129.4, 128.2, 126.8, 62.7, 59.1, 58.7, 54.4, 49.2, 45.3, 30.7, 30.5, 26.5, 24.9, 24.8, 21.6, 20.6, 14.1; IR (neat): 1446, 1305, 1151, 1085, 1033, 750, 547 cm^-1^; HRMS (ESI) Calculated for C_26_H_37_N_2_O_4_S_2_ [M+H]^+^ 505.2189 found 505.2199.

**78:** 37.1 mg, 80% yield, colorless oil; ^1^H NMR (500 MHz, CDCl_3_) δ 8.03 – 7.97 (m, 2H), 7.71 – 7.64 (m, 3H), 7.63 – 7.56 (m, 2H), 7.29 – 7.28 (m, 2H), 3.73 – 3.45 (m, 3H), 3.26 – 3.11 (m, 2H), 3.05 – 3.01 (m, 1H), 2.42 (s, 3H), 2.32 (td, *J* = 10.8, 3.4 Hz, 1H), 2.17 – 2.14 (m, 4H), 2.08 – 1.99 (m, 1H), 1.75 – 1.60 (m, 2H), 1.34 – 1.14 (m, 3H), 1.07 – 0.94 (m, 1H); ^13^C NMR (125 MHz, CDCl_3_) δ 143.2, 139.9, 138.7, 133.9, 129.7, 129.4, 128.1, 126.9, 63.1, 62.3, 58.4, 58.4, 45.6, 38.4, 30.4, 30.2, 24.8, 21.6; IR (neat): 1447, 1305, 1150, 1066, 1022, 741, 600, 546 cm^-1^; HRMS (ESI) Calculated for C_23_H_31_N_2_O_4_S_2_ [M+H]^+^ 505.2189 found 505.2199.

**79:** 40.3 mg, 82% yield, colorless oil; ^1^H NMR (500 MHz, CDCl_3_) δ 7.70 (d, *J* = 8.2 Hz, 2H), 7.54 (d, *J* = 7.3 Hz, 2H), 7.39 – 7.21 (m, 6H), 6.88 – 6.78 (m, 1H), 6.64 – 6.62 (m, 2H), 5.08 (br s, 1H), 3.92 – 3.80 (m, 4H), 3.26 – 3.11 (m, 2H), 2.80 – 2.78 (m, 1H), 2.57 – 2.44 (m, 3H), 2.42 (s, 3H), 2.28 (s, 3H); ^13^C NMR (125 MHz, CDCl_3_) δ 169.7, 160.7, 151.4, 143.4, 138.7, 137.9, 130.0, 129.8, 128.4, 128.2, 127.5, 127.3, 113.8, 111.9, 107.6, 58.2, 57.3, 55.6, 55.4, 45.8, 43.1, 36.4, 21.6; IR (neat): 1755, 1607, 1489, 1187, 1139, 1097, 1039, 956, 690, 562 cm^-1^; HRMS (ESI) Calculated for C_27_H_31_N_2_O_5_S [M+H]^+^ 495.1948 found 495.1938.

**80:** 37.4 mg, 74% yield, colorless oil; ^1^H NMR (500 MHz, CDCl_3_) δ 7.64 (d, *J* = 8.2 Hz, 2H), 7.52 (d, *J* = 7.3 Hz, 2H), 7.35 – 7.21 (m, 6H), 6.95 – 6.90 (m, 3H), 5.13 (d, *J* = 12.3 Hz, 1H), 5.08 (d, *J* = 12.3 Hz, 1H), 5.03 (s, 1H), 3.83 (s, 3H), 3.76 (dd, *J* = 13.8, 2.1 Hz, 1H), 3.13 (dd, *J* = 12.3, 1.9 Hz, 1H), 3.02 (dd, *J* = 13.8, 10.8 Hz, 1H), 2.62 (dd, *J* = 15.6, 4.4 Hz, 1H), 2.48 – 2.36 (m, 5H), 2.27 (dd, *J* = 15.6, 7.5 Hz, 1H), 2.16 (s, 3H); ^13^C NMR (125 MHz, CDCl_3_) δ 171.0, 159.9, 143.3, 138.8, 138.0, 137.1, 129.8, 129.8, 128.3, 128.3, 127.5, 127.3, 120.7, 114.1, 113.9, 66.7, 58.0, 57.3, 55.4, 55.4, 45.9, 43.0, 36.3, 21.6; IR (neat): 1733, 1456, 1343, 1269, 1157, 970, 692, 567, 542 cm^-1^; HRMS (ESI) Calculated for C_28_H_33_N_2_O_5_S [M+H]^+^ 509.2105 found 509.2100.

**81:** 14.7 mg, 40% yield, colorless oil; ^1^H NMR (500 MHz, CDCl_3_) δ 7.63 (d, *J* = 8.2 Hz, 2H), 7.31 (d, *J* = 8.0 Hz, 2H), 4.15 (q, *J* = 7.1 Hz, 2H), 3.84 (d, *J* = 10.6 Hz, 1H), 3.76 (d, *J* = 10.2 Hz, 1H), 3.13 – 3.04 (m, 1H), 2.82 – 2.71 (m, 1H), 2.57 – 2.53 (m, 1H), 2.42 (s, 3H), 2.26 –2.02 (m, 5H), 1.86 – 1.66 (m, 3H), 1.37 – 1.29 (m, 1H), 1.26 (t, *J* = 7.1 Hz, 3H); ^13^C NMR (125 MHz, CDCl_3_) δ 170.9, 143.7, 133.0, 129.8, 127.9, 62.1, 60.9, 57.6, 50.5, 50.1, 37.8, 27.3, 21.7, 20.8, 14.3; IR (neat): 1733, 1346, 1166, 1090, 968, 662, 551 cm^-1^; HRMS (ESI) Calculated for C_18_H_27_N_2_O_4_S [M+H]^+^ 367.1686 found 367.1686.

**General procedure for syntheses of 1,4-diazepanes and 1,4-diazocanes**

**Method A for syntheses of 1,4-diazepanes**: To a 2-dram vial wrapped with aluminum foil was added a 1,3-diamine substrate (0.1 mmol), NIS (0.105 mmol), and dry THF (1 mL). The reaction mixture was stirred for 1 h under argon before an allene substrate (0.15 mmol) and Cs_2_CO_3_ (0.15 mmol) was added. The reaction mixture was stirred at 60 ^o^C for 12 h, then cooled down to room temperature. NaBH_3_CN (0.2 mmol) and a co-solvent of MeOH/AcOH (pH = 4, 1mL) were added to the reaction mixture. After 3 h, the reaction was quenched with a saturated aqueous solution of NaHCO_3_, extracted with CH_2_Cl_2_ for three times. The combined organic extracts were dried over Na_2_SO_4_, filtered, and concentrated in vacuo. The crude mixture was purified on silica gel column with hexane/EtOAc as eluents to give the desired product.

**Method B for syntheses of 1,4-diazocanes**:

To a 2-dram vial wrapped with aluminum foil was added a 1,4-diamine substrate (0.1 mmol), NIS (0.105 mmol), and dry THF (4 mL). The reaction mixture was stirred for 1 h under argon before an allene substrate (0.15 mmol) and Cs_2_CO_3_ (0.15 mmol) was added. The reaction mixture was stirred at 100 ^o^C for 12 h, then cooled down to room temperature. NaBH_3_CN (0.2 mmol) and a co-solvent of MeOH/AcOH (pH = 4, 1mL) were added to the reaction mixture. After 3 h, the reaction was quenched with a saturated aqueous solution of NaHCO_3_, extracted with CH_2_Cl_2_ for three times. The combined organic extracts were dried over Na_2_SO_4_, filtered, and concentrated in vacuo. The crude mixture was purified on silica gel column with hexane/EtOAc as eluents to give the desired product.

**Method C for syntheses of 1,4-diazepanes:** To a 2-dram vial wrapped with aluminum foil was added a 1,3-diamine substrate (0.1 mmol), NCS (0.105 mmol), and dry MeCN (1 mL). The reaction mixture was stirred for 1 h under argon before an allene substrate (0.15 mmol), KI (0.2 mmol) and Cs_2_CO_3_ (0.15 mmol) was added. The reaction mixture was stirred at 60 ^o^C for 12 h, then cooled down to room temperature. NaBH_3_CN (0.2 mmol) and a co-solvent of MeOH/AcOH (pH = 4, 1mL) were added to the reaction mixture. After 3 h, the reaction was quenched with a saturated aqueous solution of NaHCO_3_, extracted with CH_2_Cl_2_ for three times. The combined organic extracts were dried over Na_2_SO_4_, filtered, and concentrated in vacuo. The crude mixture was purified on silica gel column with hexane/EtOAc as eluents to give the desired product.

**86: Method A**, 27.1 mg, 68% yield, colorless oil; ^1^H NMR (500 MHz, CDCl_3_) δ 7.64 (d, *J* = 8.2 Hz, 2H), 7.29 (d, *J* = 8.0 Hz, 2H), 4.13 (q, *J* = 7.1 Hz, 2H), 3.49 – 3.35 (m, 3H), 3.13 – 2.91 (m, 3H), 2.75 – 2.70 (m, 1H), 2.59 – 2.49 (m, 4H), 2.41 (s, 3H), 1.82 – 1.69 (m, 2H), 1.43 – 1.31 (m, 2H), 1.29 – 1.19 (m, 5H), 0.87 (t, *J* = 7.3 Hz, 3H); ^13^C NMR (125 MHz, CDCl_3_) δ 172.3, 143.3, 135.9, 129.8, 127.2, 60.5, 58.5, 51.0, 51.0, 48.4, 47.6, 36.3, 30.6, 26.8, 21.6, 20.4, 14.3, 14.2; IR (neat): 1729, 1336, 1159, 1091, 815, 735, 665, 548 cm^-1^; HRMS (ESI) Calculated for C_20_H_33_N_2_O_4_S [M+H]^+^ 397.2156 found 397.2166.

**87: Method A**, 29.8 mg, 65% yield, colorless oil; ^1^H NMR (500 MHz, CDCl_3_) δ 7.65 (d, *J* = 8.1 Hz, 2H), 7.30 – 7.24 (m, 4H), 7.16 (d, *J* = 7.8 Hz, 3H), 4.13 (q, *J* = 7.1 Hz, 2H), 3.50 – 3.36 (m, 3H), 3.13 (d, *J* = 10.5 Hz, 1H), 3.07 – 2.91 (m, 2H), 2.76 – 2.71 (m, 1H), 2.67 – 2.48 (m, 6H), 2.41 (s, 3H), 1.86 – 1.68 (m, 4H), 1.25 (t, *J* = 7.1 Hz, 3H); ^13^C NMR (125 MHz, CDCl_3_) δ 172.3, 143.3, 142.5, 135.8, 129.8, 128.5, 128.4, 127.2, 125.8, 60.5, 58.5, 51.0, 50.8, 48.4, 47.5, 36.3, 33.3, 30.1, 26.7, 21.6, 14.3; IR (neat): 1730, 1453, 1337, 1161, 1091, 700, 549 cm^-1^; HRMS (ESI) Calculated for C_25_H_35_N_2_O_4_S [M+H]^+^ 459.2312 found 459.2311.

**88: Method A**, 24.5 mg, 58% yield, colorless oil; ^1^H NMR (500 MHz, CDCl_3_) δ 7.69 – 7.60 (m, 2H), 7.33 – 7.26 (m, 3H), 6.29 – 6.28 (m, 1H), 6.15 (d, *J* = 2.9 Hz, 1H), 4.14 (q, *J* = 7.1 Hz, 2H), 3.82 (d, *J* = 14.7 Hz, 1H), 3.75 (d, *J* = 14.7 Hz, 1H), 3.54 – 3.40 (m, 3H), 3.17 – 3.08 (m, 1H), 3.05 – 2.97 (m, 1H), 2.92 – 2.90 (m, 1H), 2.78 – 2.74 (m, 1H), 2.62 – 2.47 (m, 2H), 2.41 (s, 3H), 1.83 – 1.79 (m, 2H), 1.25 (t, *J* = 7.1 Hz, 3H); ^13^C NMR (125 MHz, CDCl_3_) δ 172.1, 153.0, 143.4, 142.0, 135.7, 129.8, 127.2, 110.2, 108.2, 60.6, 58.1, 51.1, 49.2, 48.5, 48.5, 36.4, 27.3, 21.6, 14.3; IR (neat): 1728, 1335, 1158, 1090, 1012, 814, 734, 664, 548 cm^-1^; HRMS (ESI) Calculated for C_21_H_29_N_2_O_5_S [M+H]^+^ 421.1792 found 421.1793.

**89: Method A**, 35.0 mg, 75% yield, colorless oil; ^1^H NMR (500 MHz, CDCl_3_) δ 7.66 (d, *J* = 8.2 Hz, 2H), 7.31 (d, *J* = 8.1 Hz, 2H), 7.31 – 7.19 (m, 4H), 4.13 (q, *J* = 7.1 Hz, 2H), 3.80 (d, *J* = 14.1 Hz, 1H), 3.72 (d, *J* = 14.1 Hz, 1H), 3.57 – 3.41 (m, 3H), 3.20 – 3.09 (m, 1H), 3.12 – 3.04 (m, 1H), 2.85 – 2.81 (m, 1H), 2.66 – 2.49 (m, 3H), 2.42 (s, 3H), 1.82 – 1.72 (m, 2H), 1.24 (t, *J* = 7.1 Hz, 3H); ^13^C NMR (125 MHz, CDCl_3_) δ 172.0, 143.4, 138.2, 135.8, 132.6, 129.8, 128.5, 127.1, 60.7, 59.0, 54.3, 51.5, 48.4, 47.5, 36.6, 26.9, 21.6, 14.3; IR (neat): 1730, 1490, 1336, 1159, 1089, 814, 665, 549 cm^-1^; HRMS (ESI) Calculated for C_23_H_30_ClN_2_O_4_S [M+H]^+^ 465.1609 found 465.1604.

**90: Method A**, 75 mg (0.2 mmol scale), 78%, light-yellow oil; ^1^H NMR (400 MHz, CDCl_3_) δ 8.41 – 8.33 (m, 2H), 8.03 – 7.93 (m, 2H), 7.27 – 7.19 (m, 4H), 4.15 (q, *J* = 7.1 Hz, 2H), 3.83 (d, *J* = 14.1 Hz, 1H), 3.75 (d, *J* = 14.1 Hz, 1H), 3.57 – 3.45 (m, 3H), 3.31 – 3.12 (m, 2H), 2.95 – 2.63 (m, 2H), 2.62 – 2.49 (m, 2H), 1.88 – 1.75 (m, 2H), 1.26 (t, *J* = 7.1 Hz, 3H); ^13^C NMR (101 MHz, CDCl_3_) δ 171.8, 150.1, 144.6, 137.9, 132.8, 129.8, 128.5, 128.3, 124.6, 60.7, 58.8, 54.6, 51.6, 48.6, 47.4, 36.1, 27.0, 14.3; IR(neat): 1727, 1529, 1160, 1087, 653, 739, 600, 461; HRMS (ESI) Calculated for C_22_H_27_ClN_3_O_6_S [M+H]^+^ 496.1304 found 496.1306.

**91: Method A**, 30.8 mg, 66% yield, colorless oil; ^1^H NMR (500 MHz, CDCl_3_) δ 7.67 (d, *J* = 8.2 Hz, 2H), 7.31 (d, *J* = 8.0 Hz, 2H), 7.18 (t, *J* = 7.8 Hz, 1H), 6.84 (d, *J* = 7.8 Hz, 2H), 6.79 – 6.72 (m, 1H), 4.14 (q, *J* = 7.1 Hz, 2H), 3.85 – 3.68 (m, 5H), 3.59 – 3.42 (m, 3H), 3.19 – 3.17 (m, 1H), 3.12 – 3.03 (m, 1H), 2.90 – 2.84 (m, 1H), 2.72 – 2.50 (m, 3H), 2.43 (s, 3H), 1.83 – 1.73 (m, 2H), 1.24 (t, *J* = 7.1 Hz, 3H); ^13^C NMR (125 MHz, CDCl_3_) δ 172.1, 159.8, 143.4, 141.4, 135.8, 129.8, 129.2, 127.2, 120.8, 113.9, 112.5, 60.6, 59.0, 55.3, 55.1, 51.4, 48.4, 47.6, 36.5, 27.0, 21.6, 14.3; IR (neat): 1730, 1335, 1261, 1157, 1090, 1047, 734, 664, 548 cm^-1^; HRMS (ESI) Calculated for C_24_H_33_N_2_O_5_S [M+H]^+^ 461.2105 found 461.2101.

**92: Method A**, 40.9 mg, 72% yield, colorless oil; ^1^H NMR (500 MHz, CDCl_3_) δ 8.09 (br s, 1H), 7.68 – 7.64 (m, 3H), 7.44 (brs, 1H), 7.34 – 7.26 (m, 3H), 7.22 – 7.12 (m, 1H), 4.15 – 4.04 (m, 2H), 3.97 – 3.87 (m, 2H), 3.65 – 3.41 (m, 3H), 3.21 – 3.18 (m, 1H), 3.11 – 3.01 (m, 1H), 2.93 – 2.88 (m, 1H), 2.77 – 2.73 (m, 1H), 2.67 – 2.57 (m, 2H), 2.43 (s, 3H), 1.87 – 1.71 (m, 2H), 1.66 (s, 9H), 1.23 (t, *J* = 7.1 Hz, 3H); ^13^C NMR (125 MHz, CDCl_3_) δ 172.2, 149.8, 143.4, 135.6, 130.3, 129.82 127.2, 124.5, 124.3, 122.4, 120.3, 118.4, 115.2, 83.6, 60.6, 58.5, 51.0, 48.5, 47.3, 47.1, 36.0, 28.3, 26.7, 21.6, 14.3; IR (neat): 1730, 1451, 1369, 1338, 1256, 1159, 1086, 748, 549 cm^-1^; HRMS (ESI) Calculated for C_30_H_40_N_3_O_6_S [M+H]^+^ 570.2632 found 570.2631.

**93: Method A**, 25.5 mg, 65% yield, colorless oil; ^1^H NMR (500 MHz, CDCl_3_) δ 7.66 (d, *J* = 8.3 Hz, 2H), 7.31 (d, *J* = 8.0 Hz, 2H), 4.24 – 4.13 (m, 2H), 3.60 – 3.41 (m, 3H), 3.18 – 2.99 (m, 3H), 2.91 – 2.80 (m, 1H), 2.62 – 2.49 (m, 3H), 2.48 – 2.35 (m, 4H), 1.82 – 1.78 (m, 2H), 1.36 – 1.19 (m, 4H), 0.82 – 0.70 (m, 1H), 0.58 – 0.39 (m, 2H), 0.12 – 0.42 (m, 2H); ^13^C NMR (125 MHz, CDCl_3_) δ 172.3, 143.3, 135.7, 129.8, 127.2, 60.5, 58.1, 56.0, 50.8, 48.3, 36.4, 26.8, 21.6, 14.4, 10.2, 4.5, 3.2; IR (neat): 1732, 1372, 1234, 1160, 1042, 517, 472 cm^-1^; HRMS (ESI) Calculated for C_20_H_31_N_2_O_4_S [M+H]^+^ 395.1999 found 395.1991.

**94: Method A**, 37.0 mg, 73% yield, colorless oil; ^1^H NMR (500 MHz, CDCl_3_) δ 7.63 (d, *J* = 8.2 Hz, 2H), 7.28 (d, *J* = 8.1 Hz, 2H), 6.77 – 6.68 (m, 3H), 4.16 – 4.08 (m, 2H), 3.86 (s, 3H), 3.84 (s, 3H), 3.53 – 3.34 (m, 3H), 3.13 – 3.09 (m, 1H), 3.03 – 2.96 (m, 2H), 2.88 – 2.72 (m, 3H), 2.69 – 2.46 (m, 4H), 2.40 (s, 3H), 1.80 – 1.77 (m, 2H), 1.25 (t, *J* = 7.1 Hz, 3H); ^13^C NMR (125 MHz, CDCl_3_) δ 172.2, 148.7, 147.3, 143.3, 135.6, 133.0, 129.8, 127.1, 120.6, 112.7, 111.1, 60.5, 58.4, 56.0, 55.9, 53.6, 50.9, 48.4, 48.0, 36.4, 35.0, 28.4, 26.9, 21.6, 14.3; IR (neat): 1729, 1515, 1335, 1260, 1236, 1158, 1028, 814, 664, 549 cm^-1^; HRMS (ESI) Calculated for C_26_H_37_N_2_O_6_S [M+H]^+^ 505.2367 found 505.2386.

**95: Method A**, 33.7 mg, 71% yield, colorless oil; ^1^H NMR (500 MHz, CDCl_3_) δ 7.64 (d, *J* = 8.2 Hz, 2H), 7.28 (d, *J* = 8.1 Hz, 2H), 7.18 (t, *J* = 7.7 Hz, 1H), 6.77 – 6.68 (m, 3H), 4.15 – 4.10 (m, 2H), 3.79 (s, 3H), 3.50 – 3.38 (m, 3H), 3.13 – 3.11 (m, 1H), 3.07 – 2.94 (m, 2H), 2.87 – 2.59 (m, 5H), 2.57 – 2.46 (m, 2H), 2.41 (s, 3H), 1.85 – 1.74 (m, 2H), 1.26 (t, *J* = 7.1 Hz, 3H); ^13^C NMR (125 MHz, CDCl_3_) δ 172.2, 159.6, 143.4, 142.0, 135.7, 129.8, 129.3, 127.2, 121.2, 114.6, 111.3, 60.6, 58.4, 55.3, 53.4, 50.9, 48.4, 48.0, 36.4, 35.5, 26.8, 21.6, 14.4; IR (neat): 1730, 1454 1336, 1258, 1159, 1091, 1052, 664, 548 cm^-1^; HRMS (ESI) Calculated for C_25_H_35_N_2_O_5_S [M+H]^+^ 475.2261 found 475.2251.

**96: Method A**, 33.1 mg, 68% yield, colorless oil; ^1^H NMR (500 MHz, CDCl_3_) δ 7.64 (d, *J* = 8.2 Hz, 2H), 7.29 (d, *J* = 8.1 Hz, 2H), 5.80 (ddt, *J* = 16.9, 10.2, 6.7 Hz, 1H), 5.03 – 4.86 (m, 2H), 4.19 – 4.08 (m, 2H), 3.45 – 3.36 (m, 3H), 3.11 – 3.08 (m, 1H), 3.04 – 2.90 (m, 2H), 2.74 – 2.69 (m, 1H), 2.57 – 2.47 (m, 4H), 2.41 (s, 3H), 2.07 – 1.97 (m, 2H), 1.80 – 1.73 (m, 2H), 1.42 – 1.31 (m, 4H), 1.27 – 1.24 (m, 13H); ^13^C NMR (125 MHz, CDCl_3_) δ 172.3, 143.3, 139.4, 135.7, 129.8, 127.2, 114.2, 60.5, 58.5, 51.3, 50.8, 48.4, 47.7, 36.3, 34.0, 29.7, 29.6, 29.3, 29.1, 28.5, 27.3, 26.7, 21.6, 14.4; IR (neat): 1732, 1338, 1161, 1091, 911, 81, 664, 549 cm^-1^; HRMS (ESI) Calculated for C_27_H_45_N_2_O_4_S [M+H]^+^ 493.3095 found 493.3095.

**97: Method A**, 36.8 mg, 75% yield, colorless oil; ^1^H NMR (500 MHz, CDCl_3_) δ^1^H NMR (500 MHz, CDCl_3_) δ 7.65 (d, *J* = 8.2 Hz, 2H), 7.33 – 7.25 (m, 3H), 6.98 – 6.84 (m, 3H), 5.20 – 5.01 (m, 2H), 3.83 (s, 3H), 3.50 – 3.42 (m, 3H), 3.14 – 3.12 (m, 1H), 3.06 – 2.91 (m, 2H), 2.78 – 2.68 (m, 1H), 2.63 –2.54 (m, 4H), 2.43 (s, 3H), 1.78 (s, 2H), 1.39 – 1.19 (m, 4H), 0.88 (t, *J* = 7.3 Hz, 3H); ^13^C NMR (125 MHz, CDCl_3_) δ 172.1, 159.8, 143.3, 137.6, 135.8, 1298, 129.6, 127.2, 120.5, 113.9, 113.7, 66.3, 58.5, 55.4, 51.1, 50.8, 48.4, 47.5, 36.2, 30.6, 26.6, 21.6, 20.4, 14.1; IR (neat): 1733, 1598, 1457, 1335, 1267, 1159, 1091, 1051, 976, 783, 665, 549 cm^-1^; HRMS (ESI) Calculated for C_26_H_37_N_2_O_5_S [M+H]^+^ 489.2418 found 489.2409.

**98: Method A**, 21.3 mg, 61% yield, colorless oil; ^1^H NMR (500 MHz, CDCl_3_) δ 7.65 (d, *J* = 8.2 Hz, 2H), 7.31 (d, *J* = 8.1 Hz, 2H), 3.38 – 3.19 (m, 4H), 3.14 – 3.08 (m, 1H), 2.98 – 2.93 (m, 1H), 2.86 – 2.81 (m, 1H), 2.65 – 2.54 (m, 4H), 2.42 (s, 3H), 1.93 – 1.82 (m, 1H), 1.74 – 1.66 (m, 1H), 1.42 – 1.38 (m, 2H), 1.36 – 1.28 (m, 2H), 0.90 (t, *J* = 7.3 Hz, 3H); ^13^C NMR (125 MHz, CDCl_3_) δ 143.7, 135.5, 130.0, 127.2, 118.8, 59.0, 53.4, 51.0, 49.1, 46.9, 30.7, 26.7, 21.6, 20.8, 20.3, 14.1; IR (neat): 1458, 1336, 1160, 1091, 1050, 963, 816, 664, 549 cm^-1^; HRMS (ESI) Calculated for C_18_H_28_N_3_O_2_S [M+H]^+^ 350.1897 found 350.1895.

**99: Method A**, 32.3 mg, 61% yield, colorless oil; ^1^H NMR (500 MHz, CDCl_3_) δ 7.83 – 7.78 (m, 4H), 7.61 (d, *J* = 8.2 Hz, 2H), 7.53– 7.46 (m, 6H), 7.28 (d, *J* = 7.8 Hz, 2H), 3.59 – 3.37 (m, 3H), 3.09 – 3.06 (m, 1H), 2.97 – 2.80 (m, 2H), 2.80 – 2.61 (m, 1H), 2.57 – 2.33 (m, 7H), 1.81 – 1.62 (m, 2H), 1.17 – 1.02 (m, 4H), 0.80 (t, *J* = 7.0 Hz, 3H); ^13^C NMR (125 MHz, CDCl_3_) δ 143.3, 135.6, 134.3 (d, *J* = 98.8 Hz), 133.3 (d, *J* = 99.9 Hz), 131.6 (d, *J* = 16.0 Hz), 131.1 (d, *J* = 9.2 Hz), 130.9 (d, *J* = 9.3 Hz), 129.8, 128.7 (d, *J* = 11.6 Hz), 128.5 (d, *J* = 11.7 Hz), 127.2, 56.2, 53.0 (d, *J* = 6.5 Hz), 51.1, 48.4, 46.6, 31.5 (d, *J* = 70.8 Hz) 30.1, 26.9, 21.6, 20.5, 14.1; ^31^P NMR (202 MHz, CDCl_3_) δ 30.73; IR (neat): 1437, 1334, 1183, 1159, 1118, 1090, 905, 815, 718, 659, 549 cm^-1^; HRMS (ESI) Calculated for C_29_H_38_N_2_O_3_PS [M+H]^+^ 525.2335 found 525.2325.

**100: Method A**, 14.5 mg, 32% yield, colorless oil; ^1^H NMR (500 MHz, CDCl_3_) δ 8.00 (d, *J* = 8.8 Hz, 2H), 7.64 (d, *J* = 8.2 Hz, 2H), 7.29 (d, *J* = 8.2 Hz, 2H), 6.96 (d, *J* = 8.8 Hz, 2H), 3.89 (s, 3H), 3.77 – 3.55 (m, 4H), 3.37 – 3.32 (m, 1H), 3.17 – 3.07 (m, 2H), 3.03 – 2.92 (m, 2H), 2.79– 2.75 (m, 1H), 2.58– 2.54 (m, 2H), 2.42 (s, 3H), 1.86– 1.83 (m, 2H), 1.38 – 1.22 (m, 4H), 0.87 (t, *J* = 7.3 Hz, 3H); ^13^C NMR (125 MHz, CDCl_3_) δ 197.8, 163.5, 143.3, 135.7, 130.7, 130.5, 129.8, 127.2, 113.8, 57.5, 55.6, 52.1, 51.3, 48.5, 47.8, 39.2, 30.6, 27.2, 21.6, 20.5, 14.2; IR (neat): 1674, 1599, 1334, 1258, 1160, 1090, 815, 549 cm^-1^; HRMS (ESI) Calculated for C_25_H_35_N_2_O_4_S [M+H]^+^ 459.2312 found 459.2302.

**101: Method A**, 18.0 mg, 39% yield, colorless oil; ^1^H NMR (500 MHz, CDCl_3_) δ 7.65 (d, *J* = 8.2 Hz, 2H), 7.28 (d, *J* = 8.2 Hz, 2H), 4.22 – 4.00 (m, 4H), 3.49 – 3.37 (m, 2H), 3.36 – 3.27 (m, 1H), 3.21 – 3.17 (m, 1H), 3.09 – 2.99 (m, 1H), 2.94 – 2.89 (m, 1H), 2.75 – 2.65 (m, 1H), 2.61 – 2.48 (m, 2H), 2.40 (s, 3H), 2.11 – 1.93 (m, 2H), 1.79 – 1.75 (m, 2H), 1.43 – 1.23 (m, 11H), 0.88 (t, *J* = 7.3 Hz, 3H); ^13^C NMR (125 MHz, CDCl_3_) δ 143.3, 135.9, 129.8, 127.2, 61.7 (d, *J* = 6.3 Hz), 61.6 (d, *J* = 6.3 Hz), 56.9, 52.6 (d, *J* = 8.4 Hz), 51.5, 48.6, 47.3, 30.6, 27.7 (d, *J* = 137.5 Hz), 27.0, 21.6, 20.5, 16.61 (d, *J* = 3.4 Hz), 16.59 (d, *J* = 3.4 Hz) 14.2; ^31^P NMR (202 MHz, CDCl_3_) δ 30.72; IR (neat): 1337, 1240, 1161, 1054, 1029, 963, 548 cm^-1^; HRMS (ESI) Calculated for C_21_H_38_N_2_O_5_PS [M+H]^+^ 461.2234 found 461.2235.

**102: Method A**, 25.2 mg, 54% yield, colorless oil; ^1^H NMR (500 MHz, CDCl_3_) δ 8.00 (d, *J* = 7.6 Hz, 2H), 7.66 – 7.64 (m, 3H), 7.57 (t, *J* = 7.6 Hz, 2H), 7.30 (d, *J* = 8.0 Hz, 2H), 3.73 – 3.48 (m, 4H), 3.23 – 3.20 (m, 1H), 3.08 – 3.05 (m, 1H), 2.85 (dt, *J* = 12.7, 6.3 Hz, 1H), 2.76 – 2.66 (m, 1H), 2.64 – 2.55 (m, 1H), 2.45 – 2.40 (m, 5H), 1.75 – 1.73 (m, 2H), 1.25 – 2.00 (m, 4H), 0.84 (t, *J* = 7.0 Hz, 3H);^13^C NMR (125 MHz, CDCl_3_) δ 143.6, 140.4, 135.4, 133.6, 129.9, 129.3, 128.2, 127.3, 56.5, 56.3, 52.9, 52.4, 48.7, 46.9, 30.2, 27.6, 21.7, 20.4, 14.1; IR (neat): 1447, 1336, 1304, 1159, 1087, 745, 662, 549 cm^-1^; HRMS (ESI) Calculated for C_23_H_33_N_2_O_4_S_2_ [M+H]^+^ 465.1876 found 465.1869.

**103: Method C**, 29.7 mg, 68% yield, colorless oil; ^1^H NMR (500 MHz, CDCl_3_) δ 7.63 (d, *J* = 8.2 Hz, 2H), 7.28 (d, *J* = 8.0 Hz, 2H), 4.19 – 4.11 (m, 2H), 3.64 (d, *J* = 13.4 Hz, 1H), 3.50 (d, *J* = 13.3 Hz, 1H), 3.28 – 3.21 (m, 1H), 2.85 – 2.71 (m, 2H), 2.59 (d, *J* = 14.6 Hz, 1H), 2.55 – 2.49 (m, 1H), 2.48 – 2.33 (m, 6H), 2.24 – 2.21 (m, 1H), 1.54 – 1.18 (m, 17H), 0.87 (t, *J* = 7.2 Hz, 3H); ^13^C NMR (125 MHz, CDCl_3_) δ 173.2, 143.2, 136.0, 129.8, 127.3, 60.5, 59.5, 58.6, 58.0, 57.2, 55.9, 38.8, 35.0, 33.6, 32.0, 31.1, 26.4, 22.2, 21.9, 21.6, 20.4, 14.4, 14.2; IR (neat): 1727, 1452, 1339, 1159, 1091, 927, 814, 763, 656, 549 cm^-1^; HRMS (ESI) Calculated for C_25_H_41_N_2_O_4_S [M+H]^+^ 465.2782 found 465.2772.

**104: Method C**, 31.3 mg, 70% yield, colorless oil; ^1^H NMR (500 MHz, CDCl_3_) δ 7.62 (d, *J* = 8.2 Hz, 2H), 7.28 (d, *J* = 8.0 Hz, 2H), 4.22 – 4.10 (m, 2H), 3.66 – 3.63 (m, 1H), 3.34 – 3.32 (m, 1H), 3.26 – 3.22 (m, 1H), 2.83 – 2.73 (m, 2H), 2.60 – 2.58 (m, 1H), 2.52 – 2.33 (m, 8H), 1.83 – 1.71 (m, 1H), 1.65 – 1.49 (m, 4H), 1.42 – 1.19 (m, 10H), 0.87 (t, *J* = 7.2 Hz, 3H); ^13^C NMR (125 MHz, CDCl_3_) δ 173.0, 143.3, 135.7, 129.8, 127.3, 60.5, 60.0, 58.9, 58.3, 54.9, 53.7, 48.9, 36.5, 35.2, 34.1, 30.7, 24.7, 24.5, 21.6, 20.4, 14.4, 14.2; IR (neat): 1729, 1453, 1339, 1161, 1091, 1030, 814, 764, 661, 549 cm^-1^; HRMS (ESI) Calculated for C_24_H_39_N_2_O_4_S [M+H]^+^ 451.2625 found 451.2639.

**105: Method C**, 34.3 mg, 79% yield, colorless oil; ^1^H NMR (500 MHz, CDCl_3_) δ 7.65 (d, *J* = 8.2 Hz, 2H), 7.30 (d, *J* = 8.0 Hz, 2H), 4.20 – 4.08 (m, 2H), 3.68 – 3.54 (m, 2H), 3.34 – 3.23 (m, 1H), 2.87 – 2.71 (m, 3H), 2.62 – 2.39 (m, 8H), 2.11 – 2.02 (m, 1H), 1.96 – 1.73 (m, 4H), 1.62 – 1.53 (m, 1H), 1.45 – 1.34 (m, 2H), 1.30 – 1.22 (m, 5H), 0.88 (t, *J* = 7.3 Hz, 3H); ^13^C NMR (125 MHz, CDCl_3_) δ 172.6, 143.3, 135.5, 129.8, 127.3, 60.5, 59.3, 58.4, 58.2, 52.4, 52.2, 43.6, 34.8, 30.7, 30.3, 30.2, 21.6, 20.4, 15.5, 14.3, 14.2; IR (neat): 1730, 1452, 1340, 1161, 1091, 1030, 815, 663, 549 cm^-1^; HRMS (ESI) Calculated for C_23_H_37_N_2_O_4_S [M+H]^+^ 437.2469 found 437.2469.

**106: Method C**, 30.2 mg, 69% yield, colorless oil; ^1^H NMR (500 MHz, CDCl_3_) δ 7.62 (d, *J* = 8.2 Hz, 2H), 7.28 (d, *J* = 8.1 Hz, 2H), 4.22 – 4.08 (m, 2H), 3.63 – 3.60 (m, 1H), 3.24 – 3.20 (m, 2H), 2.88 – 2.75 (m, 2H), 2.60 – 2.38 (m, 8H), 2.18 – 2.15 (m, 1H), 1.41 – 1.24 (m, 8H), 0.97 (s, 3H), 0.91 – 0.77 (m, 6H); ^13^C NMR (125 MHz, CDCl_3_) δ 173.1, 143.2, 135.9, 129.8, 127.3, 62.0, 60.5, 59.8, 58.4, 56.5, 55.2, 36.6, 32.7, 31.1, 26.1, 25.5, 21.6, 20.4, 14.4, 14.2; IR (neat): 1728, 1466, 1339, 1159, 1091, 1027, 814, 765, 661, 576, 549 cm^-1^; HRMS (ESI) Calculated for C_22_H_37_N_2_O_4_S [M+H]^+^ 425.2469 found 425.2462.

**107: Method B**, 15.7 mg, 38% yield, colorless oil; ^1^H NMR (500 MHz, CDCl_3_) δ 7.64 (d, *J* = 8.2 Hz, 2H), 7.28 (d, *J* = 8.0 Hz, 2H), 4.21 – 4.08 (m, 2H), 3.43 – 3.32 (m, 3H), 3.04 – 2.99 (m, 3H), 2.57 – 2.49 (m, 4H), 2.42 (s, 3H), 1.96 – 1.83 (m, 1H), 1.73 – 1.66 (m, 1H), 1.60 – 1.48 (m, 2H), 1.36 – 1.22 (m, 8H), 0.87 (t, *J* = 7.2 Hz, 3H); ^13^C NMR (125 MHz, CDCl_3_) δ 172.9, 143.2, 135.5, 129.7, 127.5, 60.6, 57.8, 53.8, 51.5, 49.9, 48.4, 33.5, 31.4, 26.8, 26.5, 21.6, 20.4, 14.4, 14.2; IR (neat): 1730, 1461, 1339, 1160, 1092, 815, 669, 549 cm^-1^; HRMS (ESI) Calculated for C_21_H_35_N_2_O_4_S [M+H]^+^ 411.2312 found 411.2311.

**108: Method B**, 21.6 mg, 45% yield, colorless oil; ^1^H NMR (500 MHz, CDCl_3_) δ 8.10 – 8.01 (m, 2H), 7.69 (d, *J* = 8.2 Hz, 2H), 7.65 (t, *J* = 7.4 Hz, 1H), 7.58 (t, *J* = 7.6 Hz, 2H), 7.31 (d, *J* = 8.0 Hz, 2H), 3.99 – 3.95 (m, 1H), 3.76 – 3.72 (m, 1H), 3.59 – 3.47 (m, 2H), 3.07 – 2.81 (m, 4H), 2.58 – 2.40 (m, 5H), 2.35 – 2.31 (m, 1H), 2.00 – 1.95 (m, 1H), 1.74 – 1.59 (m, 2H), 1.57 – 1.46 (m, 2H), 1.34 – 1.25 (m, 3H), 0.85 (t, *J* = 7.1 Hz, 3H); ^13^C NMR (125 MHz, CDCl_3_) δ 143.6, 140.3, 134.4, 133.6, 129.8, 129.4, 128.2, 127.8, 109.8, 56.4, 55.0, 53.4, 53.2, 51.1, 48.3, 30.9, 27.8, 25.1, 21.7, 20.4, 14.1; IR (neat): 1447, 1337, 1303, 1161, 1097, 717, 549 cm^-1^; HRMS (ESI) Calculated for C_24_H_35_N_2_O_4_S_2_ [M+H]^+^ 479.2033 found 479.2019.

**109: Method B**, 31.2 mg, 59% yield, colorless oil; ^1^H NMR (500 MHz, CDCl_3_) δ 8.08 – 7.99 (m, 2H), 7.66 (d, *J* = 8.2 Hz, 3H), 7.58 (t, *J* = 7.7 Hz, 2H), 7.31 – 7.24 (m, 4H), 6.95 (t, *J* = 8.7 Hz, 2H), 4.01 –3.97 (m, 1H), 3.78 – 3.47 (m, 5H), 3.13 – 2.85 (m, 2H), 2.51 – 2.47 (m, 1H), 2.41 (s, 3H), 2.05 – 1.93 (m, 1H), 1.80 – 1.69 (m, 1H), 1.51 – 1.47 (m, 2H); ^13^C NMR (125 MHz, CDCl_3_) δ162.1 (d, *J* = 244.9 Hz), 143.7, 140.2, 135.1, 134.3, 133.8, 130.7 (d, *J* = 7.9 Hz), 129.8, 129.4, 128.2, 127.7, 115.2 (d, *J* = 21.2 Hz) 59.6, 53.7, 53.6, 51.2, 48.6, 29.8, 26.9, 25.5, 21.6; ^19^F NMR (470 MHz, CDCl_3_) δ -115.63; IR (neat): 1508, 1336, 1304, 1219, 1154, 1087, 815, 752, 721, 549 cm^-1^; HRMS (ESI) Calculated for C_27_H_32_FN_2_O_4_S_2_ [M+H]^+^ 531.1782 found 531.1771.

**110: Method B**, 24.6 mg, 45% yield, colorless oil; ^1^H NMR (500 MHz, CDCl_3_) δ 8.07 – 8.00 (m, 2H), 7.67 (t, *J* = 8.0 Hz, 3H), 7.58 (t, *J* = 7.7 Hz, 2H), 7.30 (d, *J* = 8.1 Hz, 2H), 7.23 (br s, 4H), 4.00 – 3.95 (m, 1H), 3.74 (d, *J* = 13.6 Hz, 1H), 3.67 – 3.57 (m, 2H), 3.57 – 3.48 (m, 2H), 3.13 – 2.86 (m, 4H), 2.51 – 2.47 (m, 1H), 2.42 (s, 3H), 2.03 – 1.93 (m, 1H), 1.81 – 1.69 (m, 1H), 1.54 – 1.44 (m, 2H); ^13^C NMR (125 MHz, CDCl_3_) δ 143.7, 140.1, 137.0, 134.2, 133.8, 133.0, 130.5, 129.8, 129.5, 128.6, 128.2, 127.7, 59.7, 53.7, 53.6, 51.1, 48.7, 29.8, 26.9, 25.6, 21.7; IR (neat): 1336, 1303, 1161, 1086, 726, 549 cm^-1^; HRMS (ESI) Calculated for C_27_H_32_ClN_2_O_4_S_2_ [M+H]^+^ 547.1487 found 547.1485.

**111: Method B**, 29.3 mg, 54% yield, colorless oil; ^1^H NMR (500 MHz, CDCl_3_) δ 8.05 (d, *J* = 7.7 Hz, 2H), 7.68 – 7.64 (m, 3H), 7.58 (t, *J* = 7.7 Hz, 2H), 7.29 (d, *J* = 8.1 Hz, 2H), 7.17 (t, *J* = 7.8 Hz, 1H), 6.92 – 6.84 (m, 2H), 6.87 – 6.75 (m, 1H), 4.01 – 3.97 (m, 1H), 3.80 (s, 3H), 3.76 – 3.49 (m, 5H), 3.14 (d, *J* = 13.6 Hz, 1H), 3.03 – 2.85 (m, 3H), 2.52 – 2.48 (m, 1H), 2.41 (s, 3H), 2.09 – 1.96 (m, 1H), 1.81 – 1.71 (m, 1H), 1.53 – 1.48 (m, 2H); ^13^C NMR (125 MHz, CDCl_3_) δ 159.7, 143.6, 141.1, 140.2, 134.4, 133.7, 129.8, 129.4, 129.3, 128.2, 127.7, 121.4, 114.6, 112.8, 60.5, 55.3, 53.9, 53.8, 53.6, 51.3, 48.6, 26.9, 25.7, 21.6; IR (neat): 1598, 1447, 1336, 1304, 1155, 1087, 750, 719, 690, 549 cm^-1^; HRMS (ESI) Calculated for C_28_H_35_N_2_O_5_S_2_ [M+H]^+^ 543.1982 found 543.1970.

**112: Method B**, 19.9 mg, 43% yield, colorless oil; ^1^H NMR (500 MHz, CDCl_3_) δ 7.65 (d, *J* = 8.1 Hz, 2H), 7.30 (d, *J* = 8.0 Hz, 2H), 7.29– 7.24 (m, 2H), 6.96 (t, *J* = 8.6 Hz, 2H), 4.17 (q, *J* = 7.1 Hz, 2H), 3.83 (d, *J* = 13.8 Hz, 1H), 3.74 (d, *J* = 13.8 Hz, 1H)., 3.56 – 3.54 (m, 1H), 3.41 – 3.33 (m, 2H), 3.07 – 2.85 (m, 3H), 2.64 – 2.51 (m, 3H), 2.42 (s, 3H), 1.88 – 1.72 (m, 2H), 1.61 – 1.54 (m, 1H), 1.46 – 1.41 (m, 1H), 1.27 (t, *J* = 7.1 Hz, 3H); ^13^C NMR (125 MHz, CDCl_3_) δ 172.5, 162.0 (d, *J* = 244.4 Hz), 143.4, 135.9, 135.3, 130.3 (d, *J* = 7.7 Hz), 129.8, 127.4, 115.0 (d, *J* = 21.2 Hz) 60.7, 57.4, 56.3, 51.8, 49.5, 48.9, 34.4, 27.0, 25.1, 21.6, 14.4; ^19^F NMR (470 MHz, CDCl_3_) δ -116.33; IR (neat): 1728, 1342, 1219, 1161, 1091, 817, 549 cm^-1^; HRMS (ESI) Calculated for C_24_H_32_FN_2_O_4_S [M+H]^+^ 463.2061 found 463.2060.

**113: Method B**, 20.6 mg, 43% yield, colorless oil;^1^H NMR (500 MHz, CDCl_3_) δ 7.65 (d, *J* = 8.2 Hz, 2H), 7.30 (d, *J* = 8.0 Hz, 2H), 7.24 (br s, 4H), 4.16 (q, *J* = 7.1 Hz, 2H), 3.84 (d, *J* = 14.0 Hz, 1H), 3.75 (d, *J* = 14.0 Hz, 1H), 3.60 – 3.50 (m, 1H), 3.42 – 3.31 (m, 2H), 3.03 – 2.89 (m, 3H), 2.60 – 2.50 (m, 3H), 2.42 (s, 3H), 1.86 – 1.74 (m, 2H), 1.62 – 1.55 (m, 1H), 1.48 – 1.38 (m, 1H), 1.27 (t, *J* = 7.1 Hz, 3H); ^13^C NMR (125 MHz, CDCl_3_) δ 172.5, 143.4, 138.8, 135.3, 132.6, 130.1, 129.8, 128.4, 127.4, 60.7, 57.5, 56.2, 51.8, 49.5, 49.1, 34.6, 27.0, 25.0, 21.6, 14.4; IR (neat): 1732, 1338, 1232, 1158, 1090, 1042, 813, 730, 549 cm^-1^; HRMS (ESI) Calculated for C_24_H_32_ClN_2_O_4_S [M+H]^+^ 479.1766 found 479.1755.

**114: Method B**, 24.2 mg, 35% yield, colorless oil; ^1^H NMR (500 MHz, CDCl_3_) δ 7.66 (d, *J* = 8.2 Hz, 2H), 7.31 – 7.26 (m, 4H), 6.96 (t, *J* = 8.7 Hz, 2H), 6.70 – 6.78 (m, 1H), 6.73 – 6.69 (m, 2H), 3.90 (d, *J* = 13.7 Hz, 1H), 3.82 – 3.76 (m, 4H), 3.64 – 3.41 (m, 3H), 3.15 – 3.04 (m, 1H), 3.03 – 2.87 (m, 3H), 2.83 – 2.80 (m, 1H), 2.65 – 2.60 (m, 1H), 2.42 (s, 3H), 1.95 – 1.84 (m, 1H), 1.83 – 1.72 (m, 1H), 1.63 – 1.58 (m, 1H), 1.51 – 1.40 (m, 1H); ^13^C NMR (125 MHz, CDCl_3_) δ 171.2, 162.1 (d, *J* = 244.5 Hz), 160.7, 151.9, 143.5, 135.7, 135.0, 130.4 (d, *J* = 7.8 Hz), 130.0, 129.8, 127.5, 115.1 (d, *J* = 21.1 Hz) 114.0, 112.0, 107.7, 57.3, 56.8, 55.5, 51.4, 49.7, 48.6, 33.8, 26.43, 25.4, 21.6; ^19^F NMR (470 MHz, CDCl_3_) δ -116.07; IR (neat): 1731, 1507, 1338, 1269, 1219, 1158, 549 cm^-1^; HRMS (ESI) Calculated for C_29_H_34_FN_2_O_5_S [M+H]^+^ 541.2167 found 541.2175.

**115: Method B**, 20.2 mg, 37% yield, colorless oil; ^1^H NMR (500 MHz, CDCl_3_) δ 7.65 (d, *J* = 8.2 Hz, 2H), 7.36 – 7.18 (m, 5H), 7.01 – 6.87 (m, 5H), 5.16 (d, *J* = 12.8 Hz, 1H), 5.15 (s, 2H), 3.87 – 3.80 (m, 4H), 3.74 (d, *J* = 13.6 Hz, 1H), 3.59 – 3.53 (m, 1H), 3.48 – 3.34 (m, 2H), 3.08 – 2.90 (m, 3H), 2.67 (d, *J* = 6.9 Hz, 2H), 2.59 – 2.55 (m, 1H), 2.44 (s, 3H), 1.91 – 1.74 (m, 2H), 1.65 – 1.56 (m, 1H), 1.50 – 1.38 (m, 1H); ^13^C NMR (125 MHz, CDCl_3_) δ 172.3, 162.0 (d, *J* = 248.2 Hz), 161.0, 159.9, 143.4, 137.5, 135.8, 135.2, 130.3 (d, *J* = 7.7 Hz), 129.8, 129.7, 127.5, 120.7, 115.04 (d, *J* = 21.2 Hz), 114.1, 113.8, 66.5, 57.4, 56.4, 55.4, 51.8, 49.6, 48.8, 34.3, 26.9, 25.2, 21.6; ^19^F NMR (470 MHz, CDCl_3_) δ -116.28; IR (neat): 1754, 1605, 1507, 1489, 1337, 1219, 1157, 1136, 1040, 815, 728, 548 cm^-1^; HRMS (ESI) Calculated for C_30_H_36_FN_2_O_5_S [M+H]^+^ 555.2323 found 555.2311.

**116: Method B**, 17.5 mg, 43% yield, colorless oil; ^1^H NMR (500 MHz, CDCl_3_) δ 7.65 (d, *J* = 8.2 Hz, 2H), 7.33 – 7.30 (m, 4H), 6.99 (t, *J* = 8.7 Hz, 2H), 3.95 (d, *J* = 13.7 Hz, 1H), 3.84 (d, *J* = 13.7 Hz, 1H), 3.46 – 3.29 (m, 3H), 3.15 – 2.96 (m, 3H), 2.77 – 2.60 (m, 3H), 2.42 (s, 3H), 1.99 – 1.87 (m, 1H), 1.81 – 1.69 (m, 1H), 1.58 – 1.46 (m, 2H); ^13^C NMR (125 MHz, CDCl_3_) δ 162.20 (d, *J* = 244.9 Hz), 143.8, 135.1, 134.7, 130.4 (d, *J* = 7.9 Hz), 129.9, 127.5, 119.1, 115.4 (d, *J* = 21.2 Hz) 58.17, 57.40, 51.66, 50.53, 47.42, 29.84, 26.12, 25.97, 21.65, 17.39; ^19^F NMR (470 MHz, CDCl_3_) δ -115.48; IR (neat): 1600, 1507, 1338, 1219, 1158, 1091, 817, 556, 547 cm^-1^; HRMS (ESI) Calculated for C_22_H_27_FN_3_O_2_S [M+H]^+^ 416.1803 found 416.1809.

**Determination of relative stereochemistry of 76 and 81^[2]^**


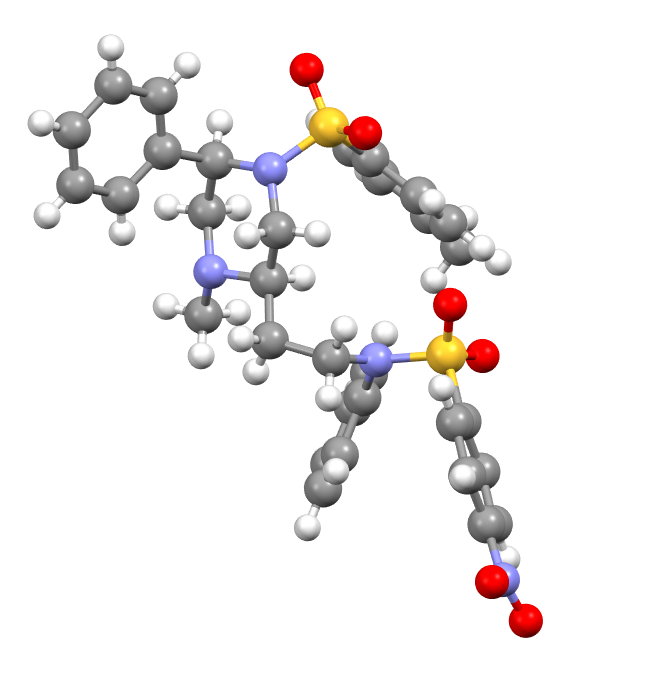


X-ray of **76b**

To a stirred solution of **76** (0.03 mmol) in THF, LiAlH_4_ (0.06 mmol) was added slowly. The reaction was slowly warmed to 0 ^o^C. After full conversion, the reaction was quenched with water. Then DCM was added. The organic layer was separated and the aqueous phase was further extracted with DCM for three times. The combined organic extracts were dried over Na_2_SO_4_, filtered, and concentrated in vacuo. The crude product **76a** was used directly in next step.

To a stirred solution of **76a**, 4-nitro-*N*-phenylbenzenesulfonamide (1.1 equiv) and PPh_3_ (1.5 equiv) in THF was added before dropwise addition of diisopropyl azodicarboxylate (DIAD) (2.0 equiv) at 0 °C. The reaction was stirred at room temperature until full conversion as judged by TLC analysis. The reaction was then quenched with saturated aqueous NH_4_Cl. The organic layer was separated and the aqueous phase was further extracted with DCM for three times. The combined organic extracts were dried over Na_2_SO_4_, filtered, and concentrated in vacuo. Purification was quickly performed with a silica gel column to give desired product **76b** as a foam solid (9.7 mg, 72% yield). Single crystal was grown in refrigerator with acetone and hexane as co-solvent (**CCDC Number: 1561429**).

^1^H NMR (500 MHz, CDCl_3_) δ 8.31 (d, *J* = 8.7 Hz, 2H), 7.74 – 7.71 (m, 4H), 7.49 – 7.48 (m, 2H), 7.38 – 7.26 (m, 9H), 6.98 – 6.97 (m, 2H), 5.03 (s, 1H), 3.62 – 3.53 (m, 3H), 3.14 (d, *J* = 12.3 Hz, 1H), 2.95 – 2.73 (m, 1H), 2.47 (s, 3H), 2.38 (dd, *J* = 12.3, 4.2 Hz, 1H), 2.06 (br s, 4H), 1.69 – 1.48 (m, 3H); ^13^C NMR (125 MHz, CDCl_3_) δ 150.2, 143.7, 143.5, 138.9, 138.0, 137.9, 129.9, 129.6, 129.0, 128.8, 128.6, 128.4, 128.2, 127.5, 127.4, 124.2, 58.6, 57.2, 55.2, 46.9, 45.1, 42.7, 29.2, 21.7; IR (neat): 1530, 1348, 1306, 1161, 1088, 845, 738, 687, 618, 564 cm^-1^; HRMS (ESI) Calculated for C_32_H_35_N_4_O_6_S_2_ [M+H]^+^ 635.1993 found 635.1993.


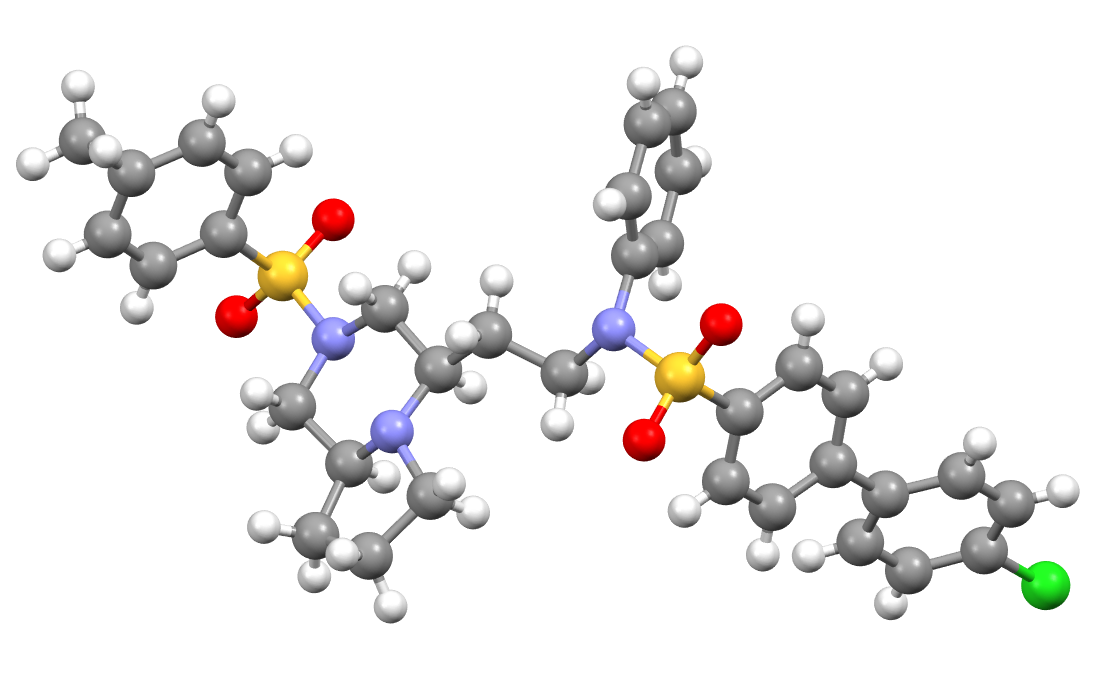


X-ray of **81b**

To a stirred solution of **81** (0.08 mmol) in THF, LiAlH_4_ (0.16 mmol) was added slowly. The reaction was slowly warmed to 0 ^o^C. After full conversion, the reaction was quenched with water. Then DCM was added. The organic layer was separated and the aqueous phase was further extracted with DCM for three times. The combined organic extracts were dried over Na_2_SO_4_, filtered, and concentrated in vacuo. The crude product **81a** was used directly in next step.

To a stirred solution of **81a**, 4'-chloro-*N*-phenylbiphenyl-4-sulfonamide (1.1 equiv.) and PPh_3_ (1.5 equiv) in THF was added before dropwise addition of diisopropyl azodicarboxylate (DIAD) (2.0 equiv) at 0 °C. The reaction was stirred at room temperature until full conversion as judged by TLC analysis. The reaction was then quenched with saturated aqueous NH_4_Cl. The organic layer was separated and the aqueous phase was further extracted with DCM for three times. The combined organic extracts were dried over Na_2_SO_4_, filtered, and concentrated in vacuo. Purification was quickly performed with a silica gel column to give desired product **81b** as a foam solid (27.0 mg, 53% yield). Single crystal was grown in refrigerator with acetone and hexane as co-solvent (**CCDC Number: 1561431**).

^1^H NMR (500 MHz, CDCl_3_) δ 7.64 – 7.62 (m, 6H), 7.55 (d, *J* = 8.5 Hz, 2H), 7.46 (d, *J* = 8.5 Hz, 2H), 7.34 – 7.31 (m, 5H), 7.07 – 7.05 (m, 2H), 3.80 – 3.78 (m, 1H), 3.66 – 3.57 (m, 3H), 2.98 – 2.93 (m, 1H), 2.42 (s, 3H), 2.31 – 2.29 (m, 1H), 2.20 – 2.12 (m, 1H), 2.06 – 2.00 (m, 2H), 1.95 – 1.90 (m, 1H), 1.79 – 1.76 (m, 2H), 1.69 – 1.64 (m, 2H), 1.49 – 1.47 (m, 1H), 1.31 – 1.24 (m, 1H); ^13^C NMR (125 MHz, CDCl_3_) δ 144.4, 143.7, 138.8, 137.7, 137.1, 134.9, 133.0, 129.8, 129.4, 128.9, 128.7, 128.4, 128.3, 127.8, 127.4, 62.1, 58.6, 50.3, 50.2, 49.5, 47.6, 31.0, 27.2, 21.6, 20.8; IR (neat): 1596, 1491, 1346, 1164, 1092, 816, 731, 606 cm^-1^; HRMS (ESI) Calculated for C_34_H_37_ClN_3_O_4_S_2_ [M+H]^+^ 650.1909 found 650.1905.

**Diversification**

**Trifluoromethylation**^[3]^: Under argon and dark condition (wrapped with aluminum foil), the reaction mixture of **11** (0.1 mmol) and NIS (1.05 mmol) in dry THF (1 mL) was stirred for 1 h. Allene **12** (0.15 mmol) and Cs_2_CO_3_ (0.15 mmol) was added to the reaction mixture. After the reaction mixture was stirred for 24 h, the reaction was quenched with water and DCM. The organic layer was separated and the aqueous phase was further extracted with DCM for three times. The combined organic extracts were dried over Na_2_SO_4_. After a quick filtration through a plug of silica gel and concentration in vacuo, the crude intermediate **13** was used directly in the next step.

Triflic acid (0.16 mmol) was added to a mixture of crude **13** and KHF_2_ (0.15 mmol) in acetonitrile (2 mL) and DMF (0.2 mmol) at 0 ^o^C. The suspension was stirred at 0 ^o^C for 5 min before Me_3_SiCF_3_ (0.3 mmol) was added. The cooling bath was then removed and the mixture was stirred for 18 h at room temperature. The reaction was quenched with saturated aqueous Na_2_CO_3_. The organic layer was separated and the aqueous phase was further extracted with DCM for three times. The combined organic extracts were dried over Na_2_SO_4_, filtered, and concentrated in vacuo. Purification was performed on a silica gel column to give desired product as colorless oil (20.1 mg, 42% yield).

^1^H NMR (500 MHz, CDCl_3_) δ 7.65 (d, *J* = 8.2 Hz, 2H), 7.34 (d, *J* = 8.0 Hz, 2H), 4.19 – 3.97 (m, 2H), 3.59 – 3.57 (m, 1H), 3.45 – 3.28 (m, 2H), 2.97 – 2.80 (m, 4H), 2.62 – 2.57 (m, 1H), 2.43 – 2.40 (m, 5H), 1.50 – 1.37 (m, 1H), 1.30 – 1.08 (m, 7H), 0.87 (t, *J* = 7.2 Hz, 3H); ^13^C NMR (125 MHz, CDCl_3_) δ 168.6, 144.0, 132.5, 129.9, 128.0, 127.0 (q, *J* = 297.5 Hz), 62.1 (q, *J* = 22.2 Hz), 61.2, 50.0, 48.4, 45.9, 45.6, 35.7, 31.0, 21.7, 20.4, 14.2, 14.0; ^19^F NMR (470 MHz, CDCl_3_) δ -66.46; IR (neat): 1737, 1463, 1359, 1225, 1166, 1121, 999, 549 cm^-1^; HRMS (ESI) Calculated for C_20_H_30_F_3_N_2_O_4_S [M+H]^+^ 451.1873 found 451.1872.

**Intramolecular Mannich Reaction^[4]^**: Under argon and dark condition (wrapped with aluminum foil), the reaction mixture of **118** or **119** (0.1 mmol) and NIS (1.05 mmol) in dry THF (1 mL) was stirred for 1 h. Allene **12** (0.15 mmol) and Cs_2_CO_3_ (0.15 mmol) was added to the reaction mixture. After the reaction mixture was stirred for 24 h, the reaction was quenched with water and DCM. The organic layer was separated and the aqueous phase was further extracted with DCM three times. The combined organic extracts were dried over Na_2_SO_4_. After a quick filtration through a plug of silica gel and concentration in vacuo, the crude intermediate **120** or **121** was used directly in the next step.

TfOH (0.6 mmol) was added to a mixture of the crude intermediate **120** or **121** in DCM (1 mL) at 0 ^o^C. The reaction was monitored by TLC. Upon full conversion, the reaction was quenched with saturated aqueous Na_2_CO_3_. The organic layer was separated and the aqueous phase was further extracted with DCM for three times. The combined organic extracts were dried over Na_2_SO_4_, filtered, and concentrated in vacuo. Purification was performed on a silica gel column to give desired product.

**122:** 26.4 mg, 54% yield, colorless oil; ^1^H NMR (500 MHz, CDCl_3_) δ 7.63 (d, *J* = 8.2 Hz, 2H), 7.29 (d, *J* = 8.0 Hz, 2H), 6.73 (s, 1H), 6.53 (s, 1H), 4.03 – 3.87 (m, 6H), 3.83 (s, 3H), 3.35 – 3.33 (m, 1H), 3.23 – 3.05 (m, 2H), 2.99 (d, *J* = 13.6 Hz, 1H), 2.91 (d, *J* = 13.6 Hz, 1H), 2.88 – 2.65 (m, 6H), 2.40 (s, 3H), 1.08 (t, *J* = 7.1 Hz, 3H); ^13^C NMR (125 MHz, CDCl_3_) δ 171.1, 148.2, 147.3, 143.8, 132.7, 129.8, 129.1, 127.9, 127.1, 111.7, 108.9, 60.4, 58.4, 56.4, 55.9, 52.3, 48.1, 45.7, 44.6, 29.8, 27.7, 21.6, 14.1; IR (neat): 1724, 1515, 1337, 1251, 1164, 1113, 992, 730, 656, 549 cm^-1^; HRMS (ESI) Calculated for C_25_H_33_N_2_O_6_S [M+H]^+^ 489.2054 found 489.2069.

**123:** 21.3 mg, 42% yield, colorless oil; ^1^H NMR (500 MHz, CDCl_3_) δ 7.63 (d, *J* = 8.2 Hz, 2H), 7.27 (d, *J* = 8.2 Hz, 2H), 6.82 (s, 1H), 6.50 (s, 1H), 3.95 – 3.86 (m, 6H), 3.84 (s, 3H), 3.67 – 3.55 (m, 2H), 3.50 (d, *J* = 14.6 Hz, 1H), 3.17 (d, *J* = 14.8 Hz, 1H), 3.03 – 2.81 (m, 4H), 2.78 – 2.72 (m, 2H), 2.53 – 2.50 (m, 1H), 2.40 (s, 3H), 2.00 – 1.93 (m, 1H), 1.76 – 1.73 (m, 1H), 0.99 (t, *J* = 7.1 Hz, 3H); ^13^C NMR (125 MHz, CDCl_3_) δ 170.8, 147.8, 147.3, 143.6, 135.4, 130.2, 129.8, 129.5, 127.2, 111.0, 110.6, 63.1, 59.7, 56.5, 55.9, 54.9, 49.0, 47.7, 45.4, 42.1, 29.9, 23.7, 21.6, 14.1; IR (neat): 1723, 1514, 1335, 1250, 1159, 1095, 1034, 815, 734, 658, 549 cm^-1^; HRMS (ESI) Calculated for C_26_H_35_N_2_O_6_S [M+H]^+^ 503.2210 found 503.2211.

**Removal of tosyl^5^ and nosyl groups^6^**

To a round-bottom flask was added **20** (30 mg, 0.075 mmol) and 0.15 M solution of methanesulfonicacid in trifluoroacetic acid/thioanosole (9/1, 1.0 mL) at room temperature under N_2_ atmosphere. The mixture was stirred for 24 h. After removal of volatiles under reduced pressure, a saturated aqueous solution of NaHCO_3_ was added. The mixture was extracted with dichloromethane (3×10 mL). The combined organic extracts were dried over Na_2_SO_4_, filtered, and concentrated in vacuo. The crude mixture was purified on silica gel column with DCM/MeOH as eluents to give the desired product as light-yellow oil (76%, 17 mg).

^1^H NMR (400 MHz, CDCl_3_) δ 7.32 – 7.21 (m, 4H), 4.14 (q, *J* = 7.0 Hz, 2H), 3.80 (d, *J* = 13.5 Hz, 1H), 3.31 (d, *J* = 13.5 Hz, 1H), 3.11 – 2.95 (m, 2H), 2.86 –2.59 (m, 7H), 2.24 – 2.20 (m, 1H), 1.25 (t, *J* = 6.9 Hz, 3H); ^13^C NMR (100MHz, CDCl_3_) δ 172.4, 137.4, 132.8, 130.1, 128.5, 60.7, 58.1, 57.1, 50.4, 50.1, 45.8, 33.7, 14.3; IR (neat): 3357, 1727, 1489, 1368, 1240, 1086, 1030, 803; HRMS (ESI) Calculated for C_15_H_22_ClN_2_O_2_ [M+H]^+^ 297.1364 found 297.1366.

To a round-bottom flask was added **90** (70 mg, 0.14 mmol), Cs_2_CO_3_ (137 mg, 3 equiv), PhSH (30 mg, 2 equiv), and 2 mL DMF under N_2_ atmosphere. The reaction mixture was stirred at 50 ^o^C for overnight. The reaction was quenched with a saturated aqueous solution of NaHCO_3_, extracted with CH_2_Cl_2_ for three times. The combined organic extracts were dried over Na_2_SO_4_, filtered, and concentrated in vacuo. The crude mixture was purified on silica gel column with DCM/MeOH as eluents to give the desired product as light-yellow oil (80%, 35 mg).

^1^H NMR (400 MHz, CDCl_3_) δ 7.24 (s, 4H), 4.10 (q, *J* = 7.1 Hz, 2H), 3.85 (d, *J* = 14.1 Hz, 1H), 3.76 (d, *J* = 14.1 Hz, 1H), 3.44 – 3.30 (m, 1H), 3.13 – 2.94 (m, 2H), 2.89 – 2.73 (m, 3H), 2.68 –2.39 (m, 4H), 1.80 – 1.70 (m, 1H), 1.61 – 1.48 (m, 1H), 1.22 (t, *J* = 7.1 Hz, 3H); ^13^C NMR (100 MHz, CDCl_3_) δ 172.6, 138.8, 132.4, 129.8, 128.4, 61.2, 60.5, 56.2, 53.0, 49.3, 47.6, 37.5, 28.9, 14.3; IR (neat): 3357, 1729, 1371, 1240, 1174, 1086, 1045, 1014, 838, 731; HRMS (ESI) Calculated for C_16_H_24_ClN_2_O_2_ [M+H]^+^ 311.1521 found 311.1524.

**Mechanistic study experiments**

Under argon and dark conditions, the reaction mixture of diamine **135** (0.1 mmol), NIS (1.05 mmol) in dry THF (1 mL) was stirred for 1 h before Cs_2_CO_3_ (0.15 mmol) was added. After the reaction mixture was stirred for 12 h, the reaction was quenched with a saturated aqueous solution of NaHCO_3_. DCM and water were then added. The organic layer was separated and the aqueous phase was further extracted with DCM three times. The combined organic extracts were dried over Na_2_SO_4_ and concentrated in vacuo. Purification was performed by a silica gel column eluted with hexane/EtOAc to give **137** (15.2 mg, 37% yield) as a white solid.

CCDC: 1582123; ^1^H NMR (500 MHz, CDCl_3_) δ 7.73 (d, *J* = 8.2 Hz, 2H), 7.31 (d, *J* = 8.1 Hz, 2H), 5.16 – 5.14 (m, 1H), 3.72 – 3.51 (m, 1H), 3.26 – 2.98 (m, 3H), 2.72 – 2.56 (m, 6H), 2.44 (s, 3H), 2.17 – 1.86 (m, 3H), 1.71 – 1.41 (m, 5H), 0.75 – 0.71 (m, 1H), 0.40 – 0.37 (m, 2H), 0.09 (br s, 1H); ^13^C NMR (125MHz, CDCl_3_) δ 179.0, 143.5, 136.2, 129.8, 127.3, 65.5, 62.6, 56.0, 52.9, 41.9, 28.6, 28.4, 24.4, 21.7, 12.0, 8.0, 2.3; IR(neat): 3262, 1693, 1396, 1326, 1160, 1092, 815, 653, 551 cm^-1^; HRMS Calculated for C_21_H_30_N_3_O_4_S [M+H]^+^ 420.1952 found 420.1954.

Under argon and dark condition, the reaction mixture of **135** (0.1 mmol), NIS (1.05 mmol) in dry THF (1 mL) was stirred for 1 h before allene **12** (0.15 mmol) and Cs_2_CO_3_ (0.15 mmol) was added to the reaction mixture. After the reaction mixture was stirred at 60 ^o^C for 12 h, NaBH_3_CN (0.2 mmol) and MeOH/AcOH (pH = 4, 1mL) co-solvent were added successively. After 3 h, the reaction was quenched by a saturated aqueous solution of NaHCO_3_. DCM and water were then added. The organic layer was separated and the aqueous phase was further extracted with DCM three times. The combined organic extracts were dried over Na_2_SO_4_ and concentrated in vacuo. The crude NMR showed the reaction is very mess. Purification of the reaction showed no desired product.

Under argon and dark condition, the reaction mixture of **135** (0.1 mmol), NCS (1.05 mmol) in dry THF (1 mL) was stirred for 1 h before allene **12** (0.15 mmol), KI (0.2 mmol) and Cs_2_CO_3_ (0.15 mmol) was added to the reaction mixture. After the reaction mixture was stirred at 100 ^o^C for 12 hours, the NaBH_3_CN (0.2 mmol) and MeOH/AcOH (pH = 4, 1mL) co-solvent were added successively. After 3 hours, the reaction was quenched by the saturated solution of NaHCO_3_. Then the DCM and water were added. The organic layer was separated, and the aqueous phase was further extracted with DCM three times, and the combined organic extracts were dried over Na_2_SO_4_ and concentrated in vacuo. Purification was performed by a silica gel column eluted with hexane/EtOAc to give **136** (20.2 mg, 47% yield) and **137** (6.3 mg, 15% yield).

**136:** ^1^H NMR (500 MHz, CDCl_3_) δ 7.64 (d, *J* = 8.2 Hz, 2H), 7.34 (d, *J* = 8.0 Hz, 2H), 5.23 (dt, *J* = 10.6, 7.4 Hz, 1H), 4.73 (t, *J* = 10.2 Hz, 1H), 4.29 – 4.10 (m, 2H), 3.38 – 3.20 (m, 3H), 2.88 – 2.36 (m, 11H), 2.13 (q, *J* = 7.2 Hz, 2H), 1.59 – 1.42 (m, 3H), 1.30 (t, *J* = 7.1 Hz, 3H), 0.78 – 0.62 (m, 2H), 0.34 – 0.23 (m, 2H); ^13^C NMR (125 MHz, CDCl_3_) δ 172.4, 143.8, 134.7, 132.6, 129.8, 128.0, 127.3, 60.8, 54.4, 53.1, 49.9, 47.2, 46.0, 30.3, 27.0, 25.2, 21.6, 14.3, 9.7, 6.9; IR (neat): 1728, 1352, 1304, 1167, 1120, 945, 815, 750, 664, 549 cm^-1^; HRMS Calculated for C_23_H_35_N_2_O_4_S [M+H]^+^ 435.2312 found 435.2306.

Under argon and dark condition, the reaction mixture of **138** (0.1 mmol), NCS (1.05 mmol) in dry THF (1 mL) was stirred for 1 h before allene **12** (0.15 mmol), KI (0.2 mmol) and Cs_2_CO_3_ (0.15 mmol) was added to the reaction mixture. After the reaction mixture was stirred at 100 ^o^C for 12 h, NaBH_3_CN (0.2 mmol) and MeOH/AcOH (pH = 4, 1mL) co-solvent were added successively. After 3 h, the reaction was quenched with a saturated aqueous solution of NaHCO_3_. DCM and water were then added. The organic layer was separated and the aqueous phase was further extracted with DCM three times. The combined organic extracts were dried over Na_2_SO_4_ and concentrated in vacuo. Purification was performed by a silica gel column eluted with hexane/EtOAc to give desire product (19.1 mg, 38% yield).

^1^H NMR (500 MHz, CDCl_3_) δ 7.66 – 7.59 (m, 2H), 7.34 (d, *J* = 8.0 Hz, 2H), 7.23 – 7.18 (m, 2H), 7.18 – 7.09 (m, 2H), 6.34 (d, *J* = 11.6 Hz, 1H), 5.59 (dt, *J* = 11.6, 7.4 Hz, 1H), 4.21 – 4.07 (m, 2H), 3.26 – 3.16 (m, 3H), 2.75 – 2.55 (m, 4H), 2.52 – 2.40 (m, 6H), 2.39 – 2.31 (m, 1H), 2.26 – 2.21 (m, 2H), 1.55 – 1.49 (m, 2H), 1.26 (t, *J* = 7.1 Hz, 3H); ^13^C NMR (125 MHz, CDCl_3_) δ 172.3, 143.8, 136.0, 132.9, 132.5, 132.3, 130.1, 129.8, 128.5, 128.4, 127.9, 60.9, 54.3, 52.9, 49.9, 47.1, 46.0, 30.1, 27.0, 26.0, 21.7, 14.3; IR(neat): 1728, 1490, 1351, 1167, 1091, 953, 843, 664, 549 cm^-1^; HRMS Calculated for C_26_H_34_ClN_2_O_4_S [M+H]^+^ 505.1922 found 505.1915.

Under nitrogen and dark condition, the reaction mixture of diamine (0.1 mmol), allene (1.5 equiv.) and Cs_2_CO_3_ (1.5 equiv.) in dry THF (1 mL) was stirred for 3 h before NaBH_3_CN (2.0 equiv.) and MeOH/AcOH (pH = 4, 1 mL) co-solvent were added successively. After 3 h, the reaction was quenched with a saturated aqueous solution of NaHCO_3_. DCM and water were then added and the organic layer was separated. The aqueous phase was extracted with DCM three times and the combined organic extracts were dried over Na_2_SO_4_ and concentrated in vacuo. Purification was performed by a silica gel column eluted with hexane/EtOAc to give products **143** and **144**.

**143**, 28 mg, 50% yield based on **140**; colorless oil; ^1^H NMR (400 MHz, CDCl_3_) δ 7.54 (d, *J* = 8.2 Hz, 2H), 7.33 – 7.16 (m, 6H), 5.61 (s, 1H), 4.25 – 4.03 (m, 4H), 3.62 (d, *J* = 13.9 Hz, 1H), 3.48 (d, *J* = 13.9 Hz, 1H), 3.38 – 3.24 (m, 2H), 3.20 – 3.09 (m, 1H), 2.71 – 2.46 (m, 3H), 2.42 (s, 3H), 2.30 – 2.26 (m, 1H), 2.09 (s, 3H), 1.36 – 1.19 (m, 7H), 1.07 (d, *J* = 6.7 Hz, 3H); ^13^C NMR (100 MHz, CDCl_3_) δ 172.2, 166.3, 152.8, 144.0, 138.7, 135.9, 132.8, 130.1, 129.8, 128.5, 127.5, 116.6, 60.5, 60.3, 54.7, 54.3, 49.5, 48.0, 39.7, 21.7, 18.3, 14.9, 14.4; IR(neat): 1727, 1715, 1636, 1352, 1164, 1142, 1088, 1015, 710, 654, 548; HRMS (ESI) Calculated for C_28_H_38_ClN_2_O_6_S [M+H]^+^ 565.2134 found 565.2137.

**144**, 3.0 mg, 7% yield based on **140**; colorless oil; ^1^H NMR (400 MHz, CDCl_3_) δ 7.70 (d, *J* = 8.2 Hz, 2H), 7.36 – 7.25 (m, 2H), 7.23 – 7.12 (m, 2H), 7.00 (d, *J* = 8.3 Hz, 2H), 5.73 (dd, *J* = 6.3, 4.3 Hz, 1H), 4.20 (dq, *J* = 10.8, 7.0 Hz, 1H), 4.05 (dq, *J* = 10.8, 7.1 Hz, 1H), 3.54 (d, *J* = 13.8 Hz, 1H), 3.25 (d, *J* = 13.9 Hz, 1H), 3.22 – 3.12 (m, 1H), 2.95 – 2.78 (m, 2H), 2.66 – 2.37 (m, 6H), 2.25 – 2.21 (m, 1H), 1.21 (t, *J* = 7.1 Hz, 3H), 0.98 (d, *J* = 6.7 Hz, 3H); ^13^C NMR (100 MHz, CDCl_3_) δ 172.8, 143.1, 138.0, 137.2, 132.9, 130.0, 129.6, 128.5, 127.3, 61.0, 53.5, 52.4, 48.9, 41.3, 39.8, 21.7, 14.2, 13.3; IR(neat): 3276, 1717, 1330, 1303, 1193, 1159, 1090, 1015, 814, 662, 551; HRMS (ESI) Calculated for C_22_H_30_ClN_2_O_4_S [M+H]^+^ 453.1609 found 453.1610.

Note: When a 1:1 ratio of **140** and **12** was used for the above transformation, **143** and **144** were produced in 55% and 17% yield, respectively.

Under nitrogen and dark condition, the reaction mixture of diamine (0.1 mmol), allene (1.5 equiv.) and Cs_2_CO_3_ (1.5 equiv.) in dry THF (1 mL) was stirred for 3 h before NIS (1.05 equiv.) or ICl (1.05 equiv.) was added. After the reaction was stirred for 12 h, NaBH_3_CN (2.0 equiv.) and MeOH/AcOH (pH = 4, 1 mL) co-solvent were added successively. After 3 h, the reaction was quenched with a saturated aqueous solution of NaHCO_3_. DCM and water were then added and the organic layer was separated. The aqueous phase was extracted with DCM three times and the combined organic extracts were dried over Na_2_SO_4_ and concentrated in vacuo. Purification was performed by a silica gel column eluted with hexane/EtOAc to give products **143** in 33% yield with NIS and 27% yield with ICl, but desired product **20** was observed.

Under argon and dark condition, the reaction mixture of **140** (0.1 mmol), ICl (1.05 mmol) in dry THF (1 mL) was stirred for 1 h before allene **12** (0.15 mmol) and Cs_2_CO_3_ (0.15 mmol) was added. After the reaction mixture was stirred for 12 h, NaBH_3_CN (0.2 mmol) and MeOH/AcOH (pH = 4, 1mL) co-solvent were added successively. After 3 h, the reaction was quenched with a saturated aqueous solution of NaHCO_3_. DCM and water were then added. The organic layer was separated and the aqueous phase was further extracted with DCM three times. The combined organic extracts were dried over Na_2_SO_4_ and concentrated in vacuo. Purification was performed by a silica gel column eluted with hexane/EtOAc to give desire product **20** (6.0 mg, 13% yield) and byproduct **144** (14 mg, 31% yield).

Under argon and dark condition, the reaction mixture of **12** (0.15 mmol), ICl (1.05 mmol) or NIS (0.105 mmol) in dry THF (1 mL) was stirred for 1 h before diamine **140** (0.1 mmol) and Cs_2_CO_3_ (0.15 mmol) was added. After the reaction mixture was stirred for 12 h, NaBH_3_CN (0.2 mmol) and MeOH/AcOH (pH = 4, 1mL) co-solvent were added successively. After 3 h, the reaction was quenched with a saturated aqueous solution of NaHCO_3_. DCM and water were then added. The organic layer was separated and the aqueous phase was further extracted with DCM three times. The combined organic extracts were dried over Na_2_SO_4_ and concentrated in vacuo. Purification was performed by a silica gel column eluted with hexane/EtOAc to give desire product **20** (24 mg, 53% yield for NIS; 7.0 mg, 15% yield for ICl) and byproduct **144** (trace for NIS; 13 mg, 29% yield for ICl).

Under argon and dark condition, diamine **140** (0.1 mmol), allene **12** (0.15 mmol), ICl (1.05 mmol) or NIS (0.105 mmol), and Cs_2_CO_3_ (0.15 mmol) were mixed in dry THF (1 mL) at the same time. After the reaction mixture was stirred for 12 h, NaBH_3_CN (0.2 mmol) and MeOH/AcOH (pH = 4, 1mL) co-solvent were added successively. After 3 h, the reaction was quenched with a saturated aqueous solution of NaHCO_3_. DCM and water were then added. The organic layer was separated and the aqueous phase was further extracted with DCM three times. The combined organic extracts were dried over Na_2_SO_4_ and concentrated in vacuo. Purification was performed by a silica gel column eluted with hexane/EtOAc to give desire product **20** (25 mg, 56% yield for NIS; 1.0 mg, 2% yield for ICl) and byproduct **144** (none for NIS; 17 mg, 38% yield for ICl).


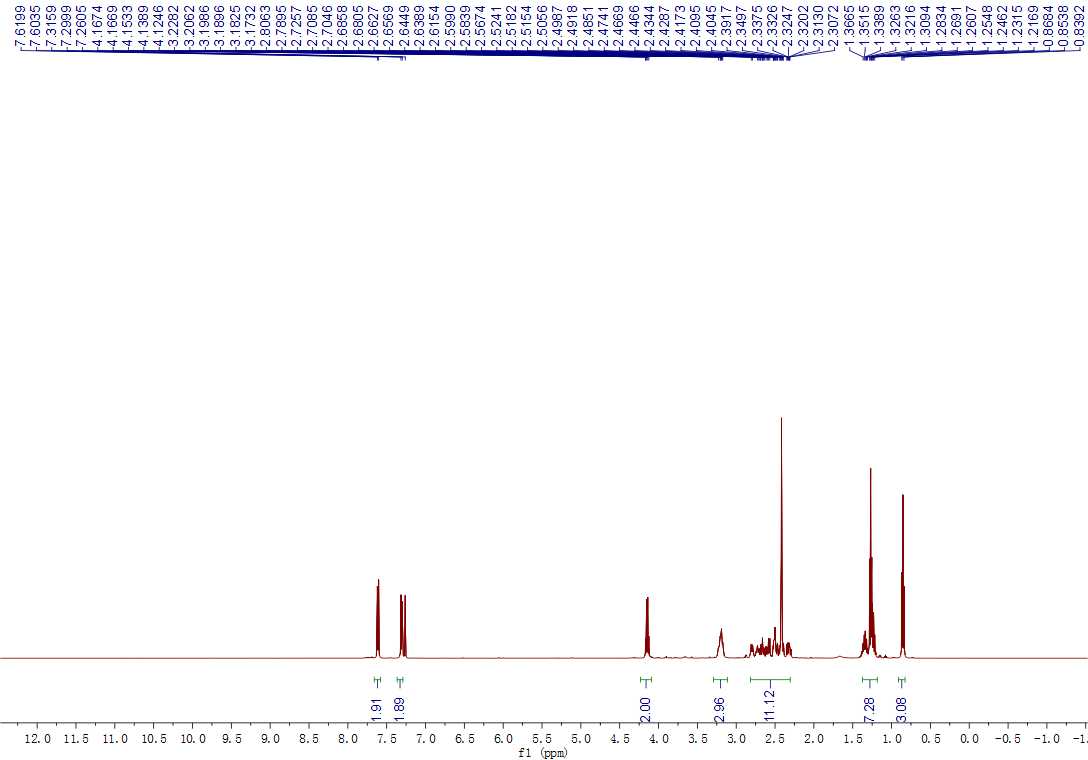


**Supplementary Figure 9.** ^1^H NMR spectrum of compound **14**.


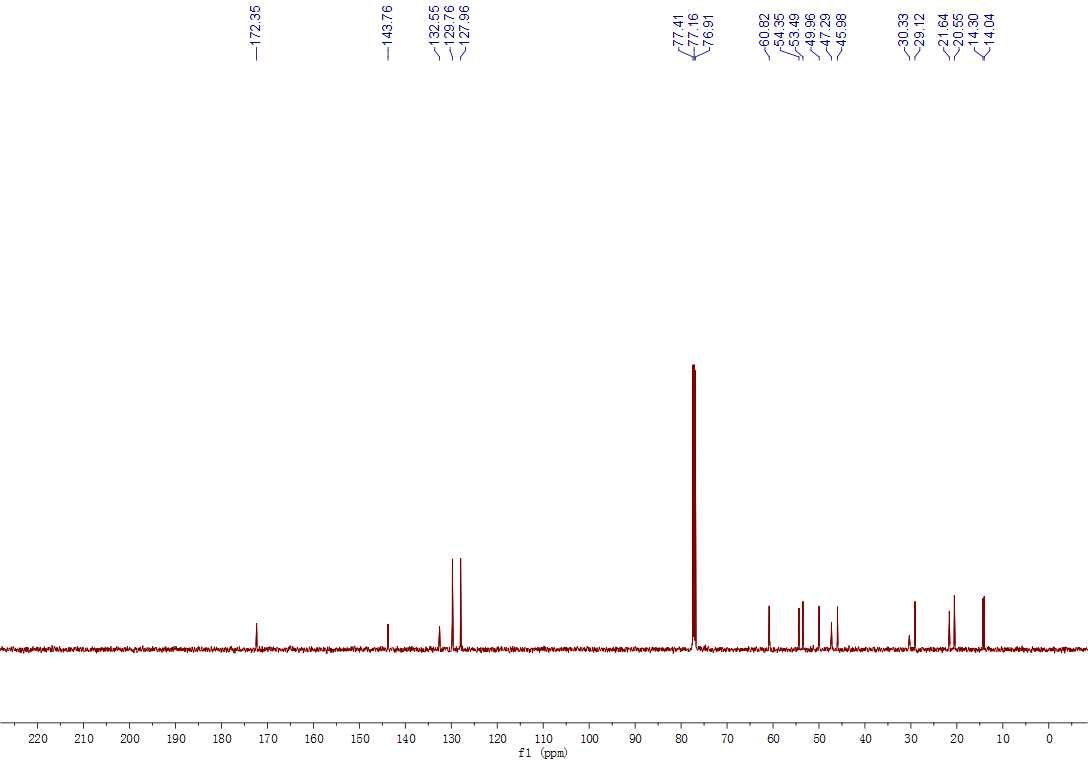


**Supplementary Figure 10.** ^13^C NMR spectrum of compound **14**.


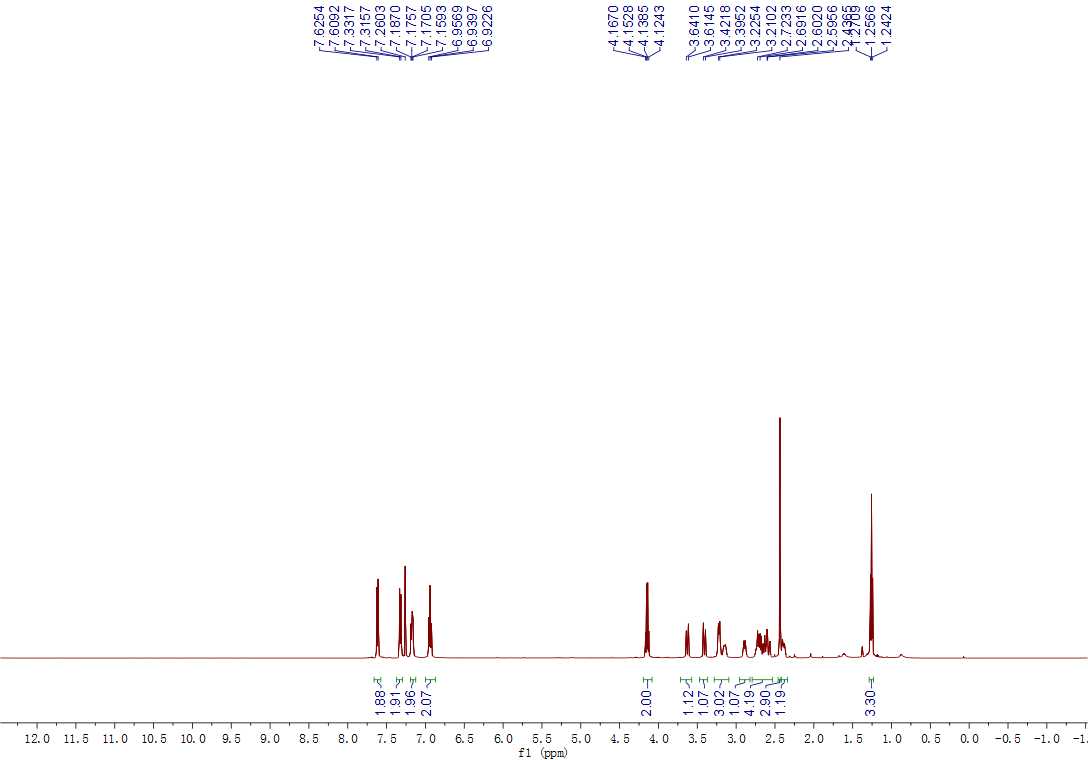


**Supplementary Figure 11.** ^1^H NMR spectrum of compound **19**.


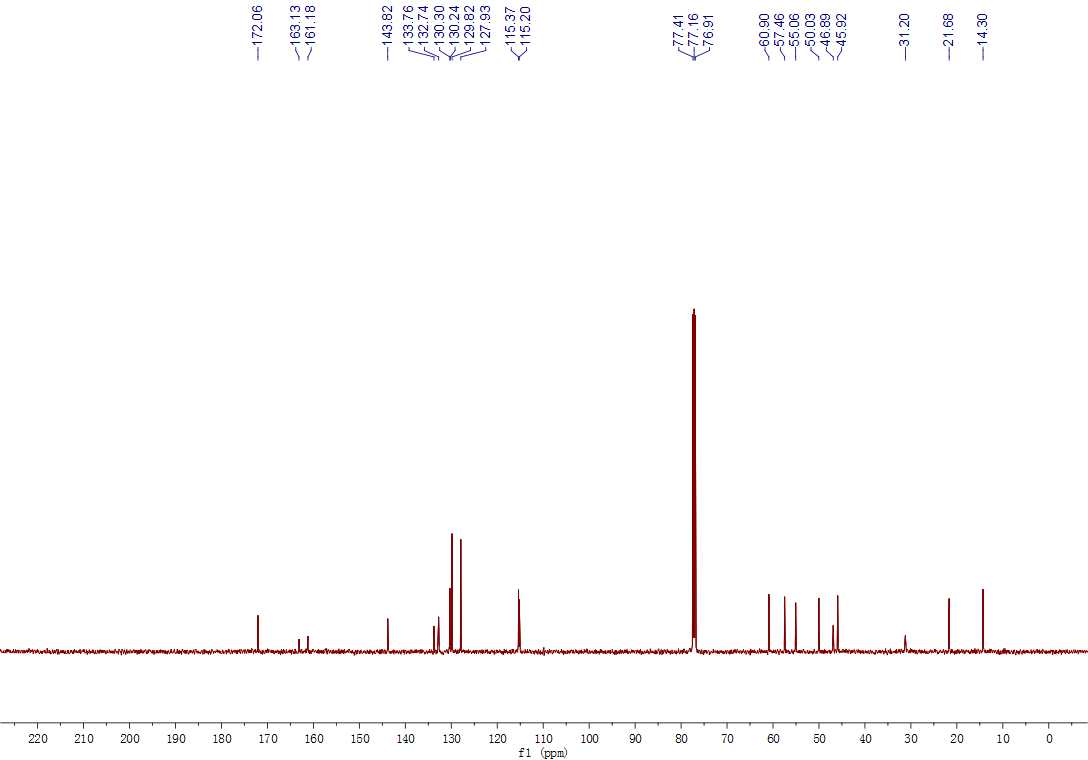


**Supplementary Figure 12.** ^13^C NMR spectrum of compound **19**.


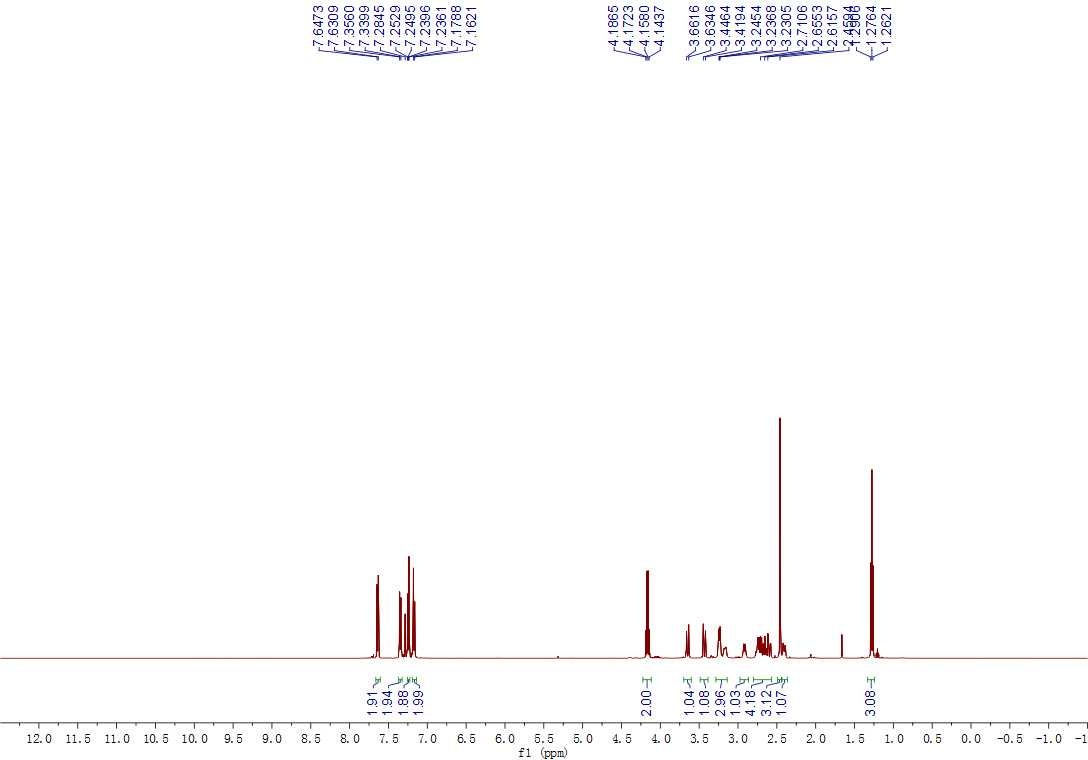


**Supplementary Figure 13.** ^1^H NMR spectrum of compound **20**.


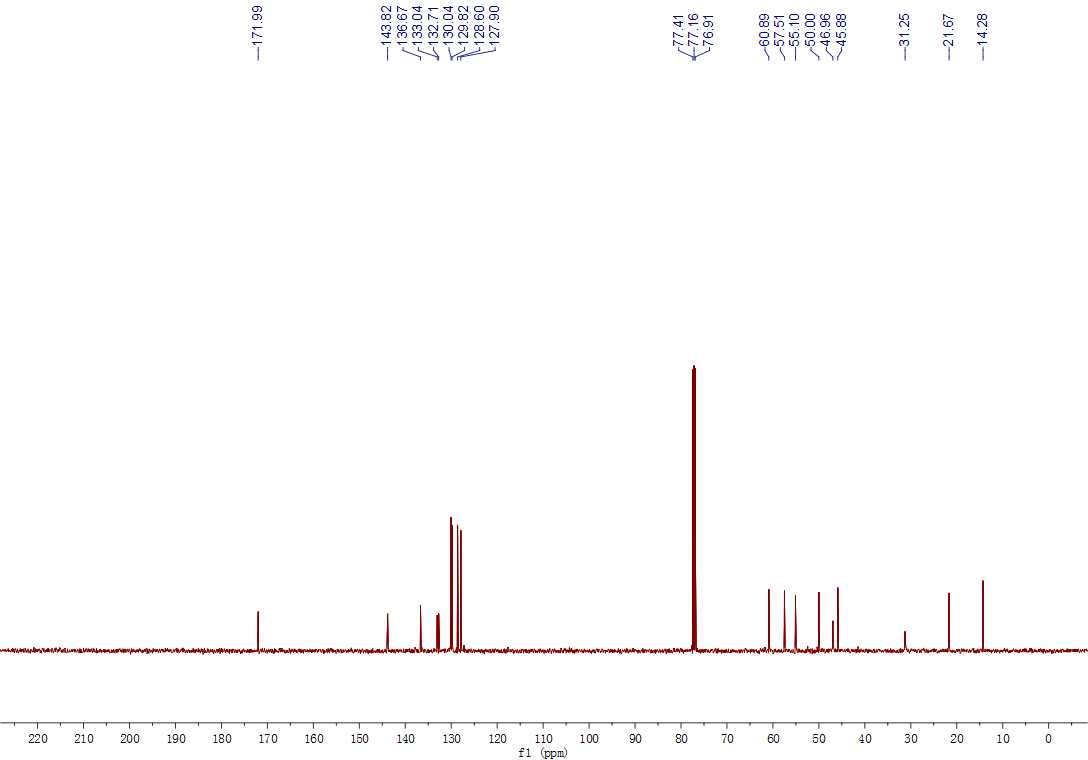


**Supplementary Figure 14.** ^13^C NMR spectrum of compound **20**.


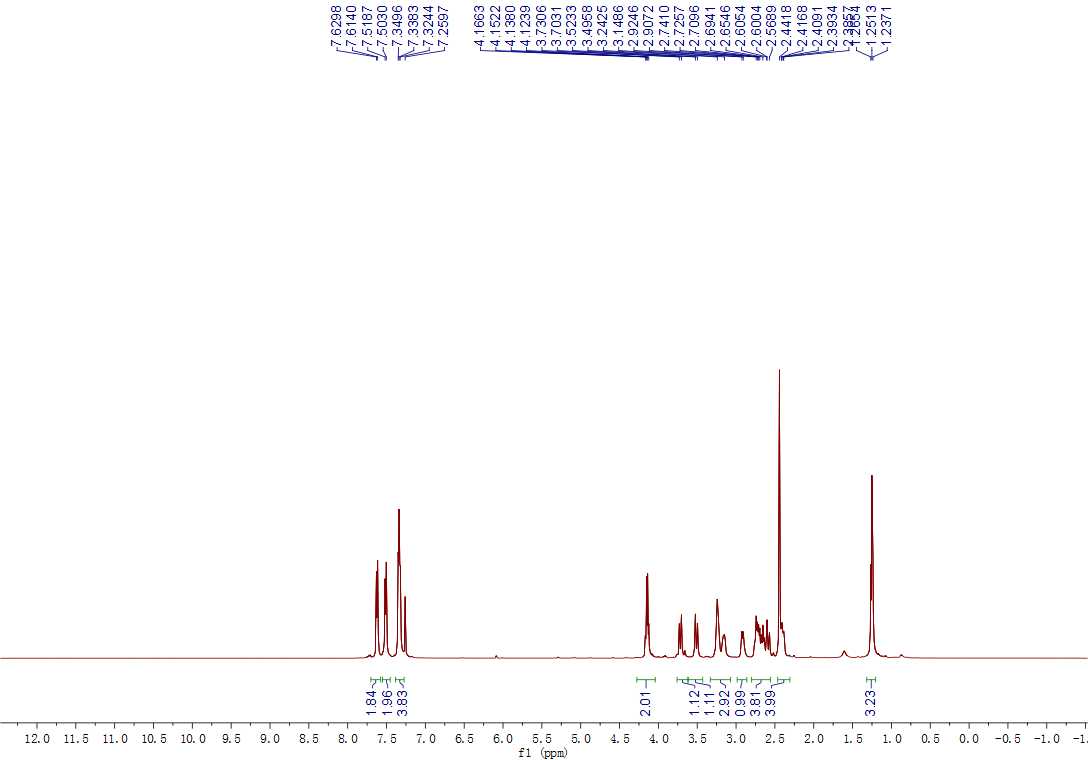


**Supplementary Figure 15.** ^1^H NMR spectrum of compound **21**.


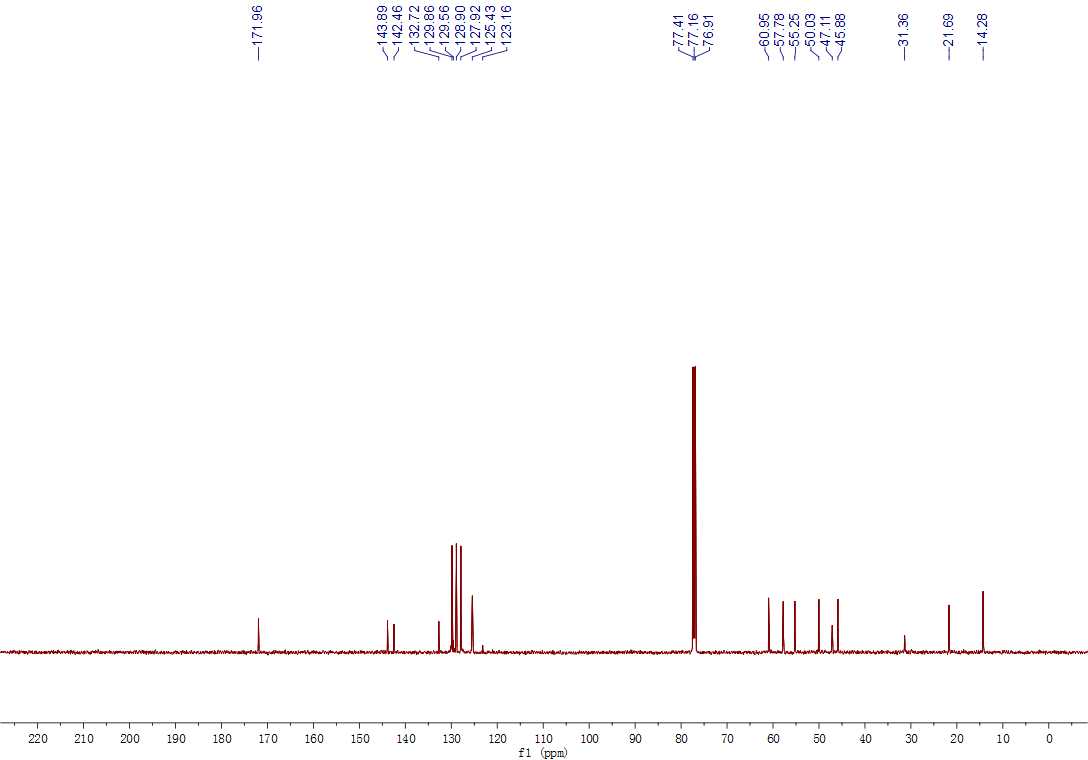


**Supplementary Figure 16.** ^13^C NMR spectrum of compound **21**.


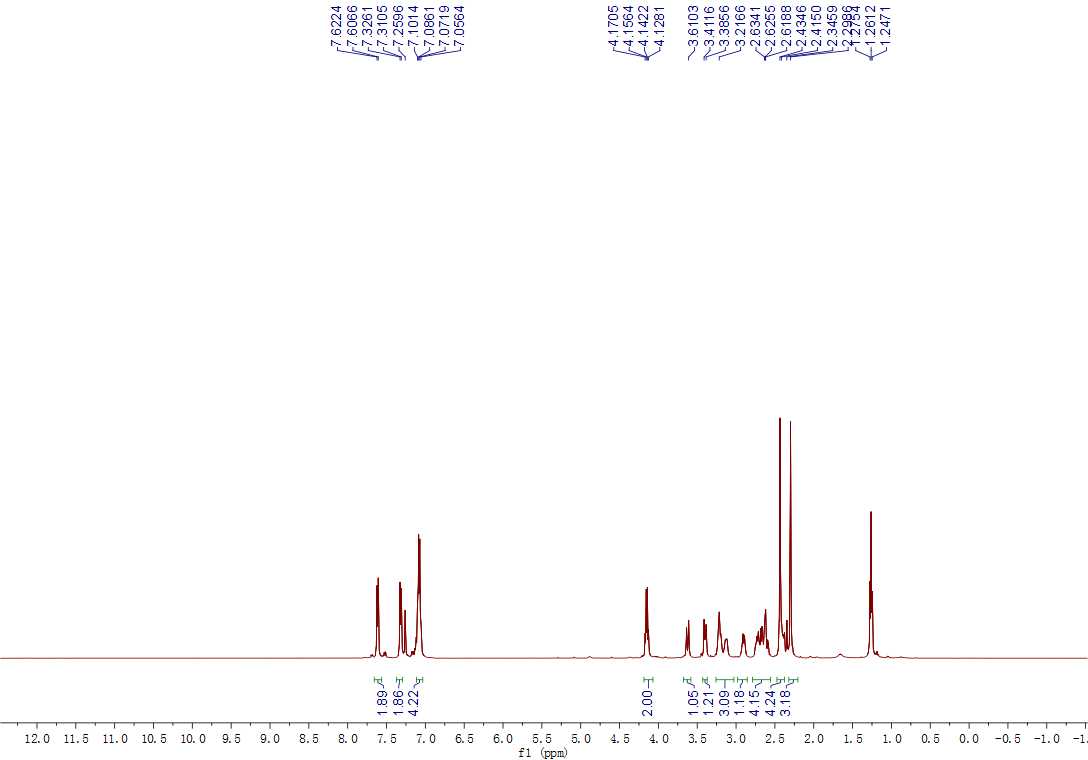


**Supplementary Figure 17.** ^1^H NMR spectrum of compound **22**.


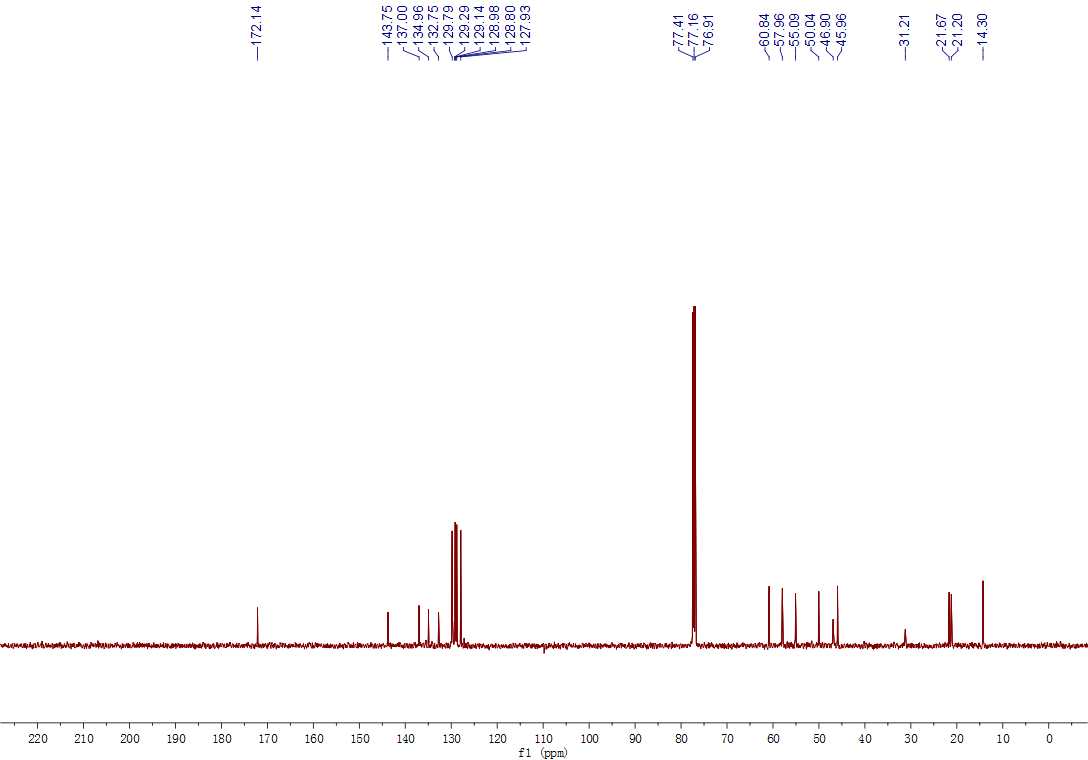


**Supplementary Figure 18.** ^13^C NMR spectrum of compound **22**.


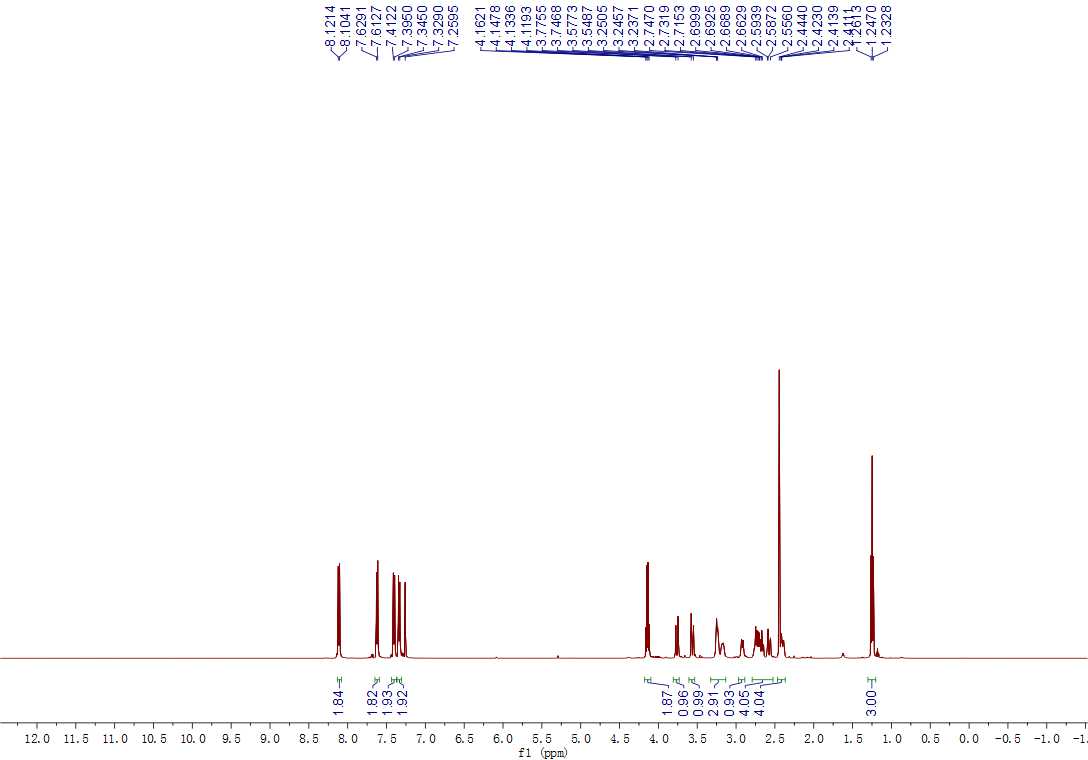


**Supplementary Figure 19.** ^1^H NMR spectrum of compound **23**.


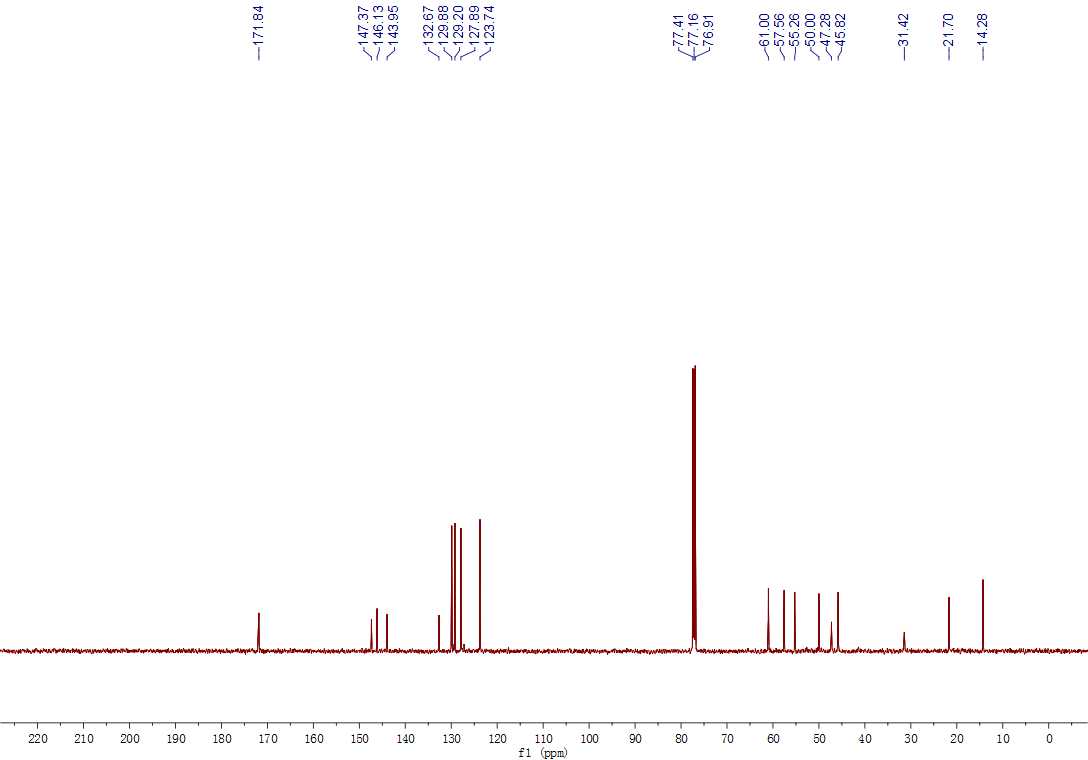


**Supplementary Figure 20.** ^13^C NMR spectrum of compound **23**.


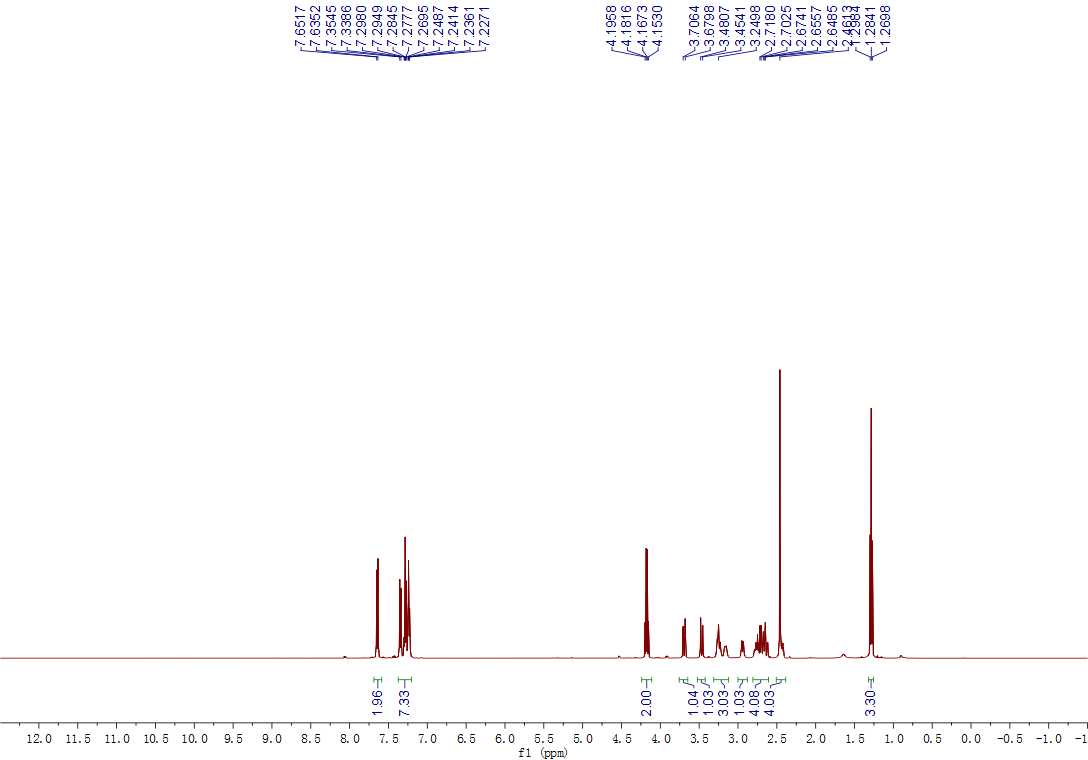


**Supplementary Figure 21.** ^1^H NMR spectrum of compound **24**.


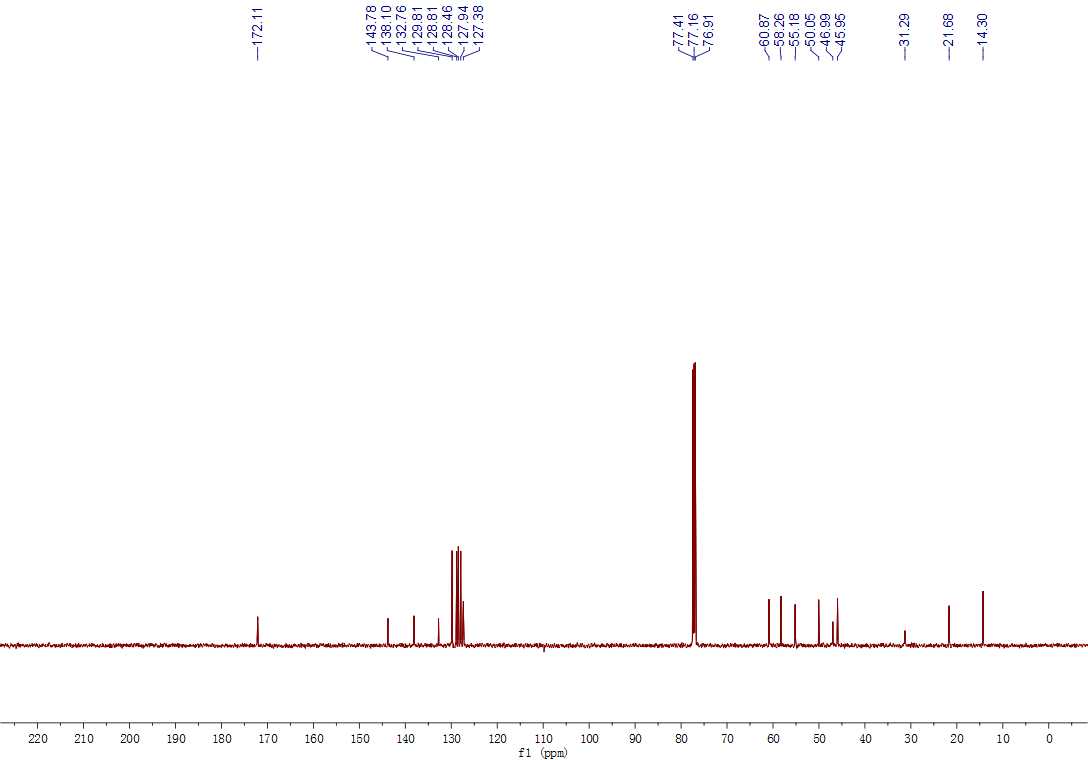


**Supplementary Figure 22.** ^13^C NMR spectrum of compound **24**.


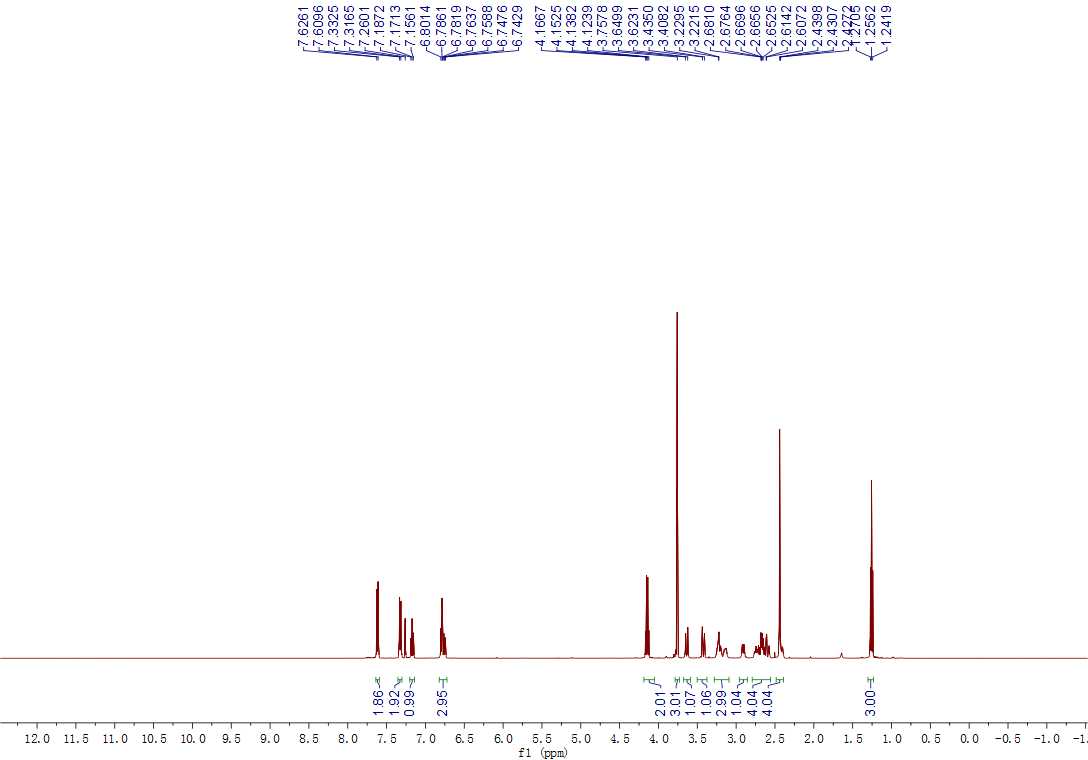


**Supplementary Figure 23.** ^1^H NMR spectrum of compound **25**.


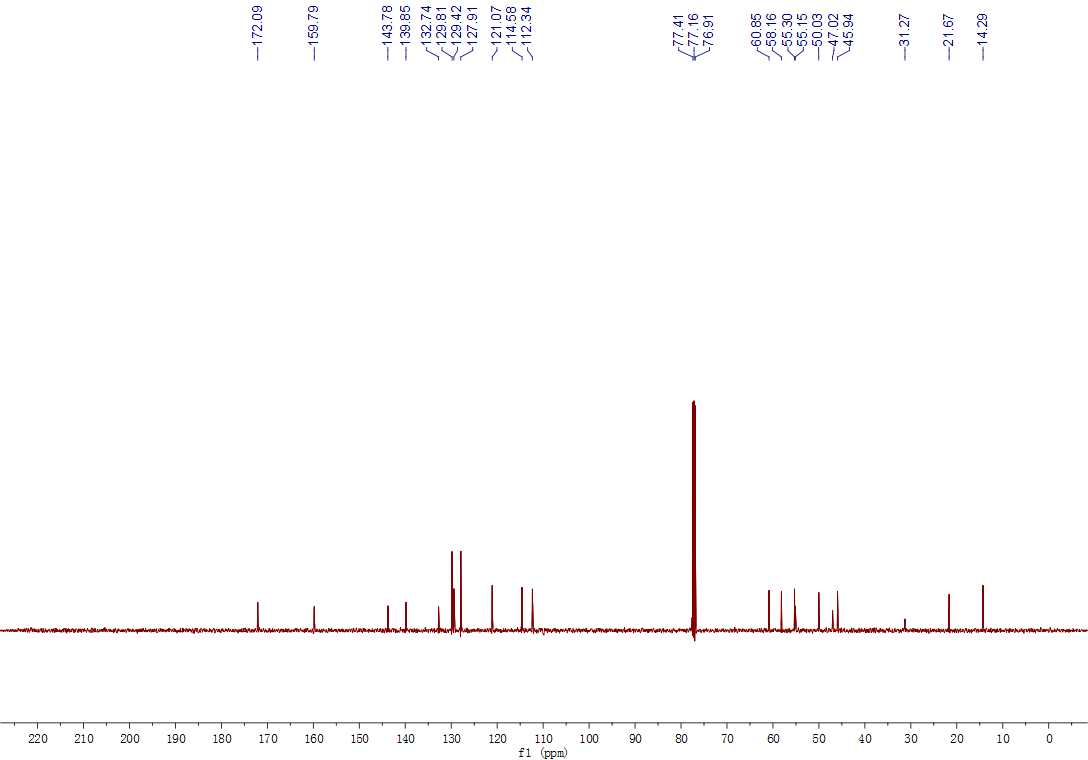


**Supplementary Figure 24.** ^13^C NMR spectrum of compound **25**.


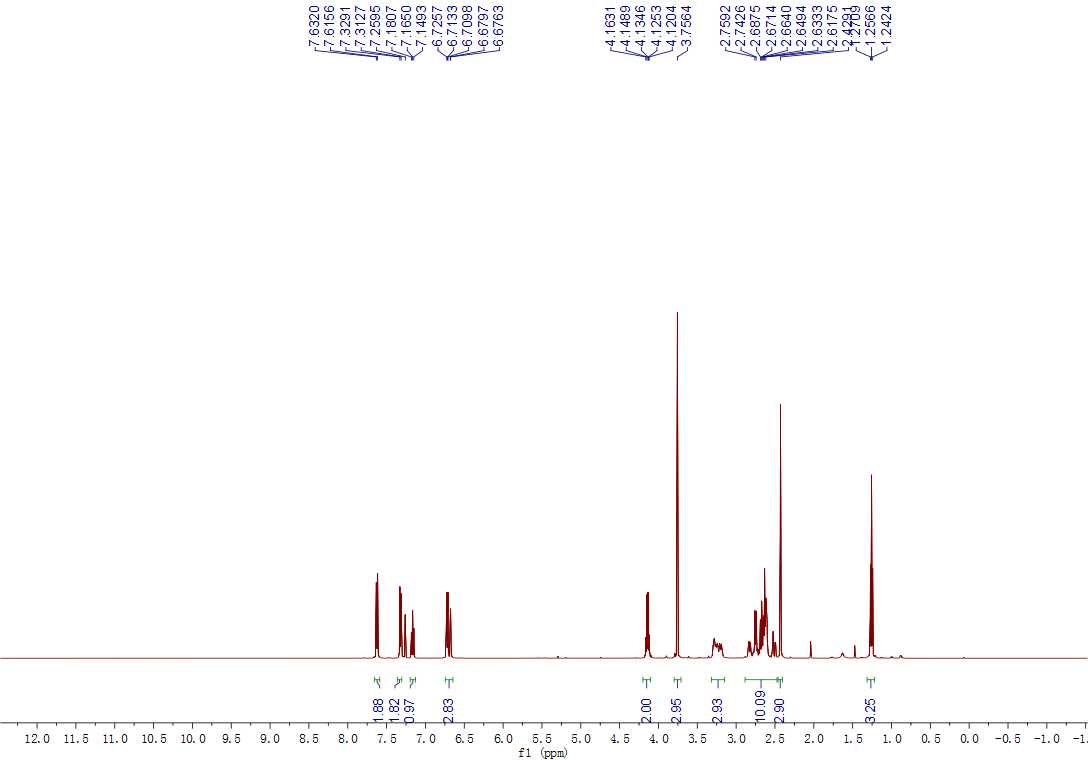


**Supplementary Figure 25.** ^1^H NMR spectrum of compound **26**.


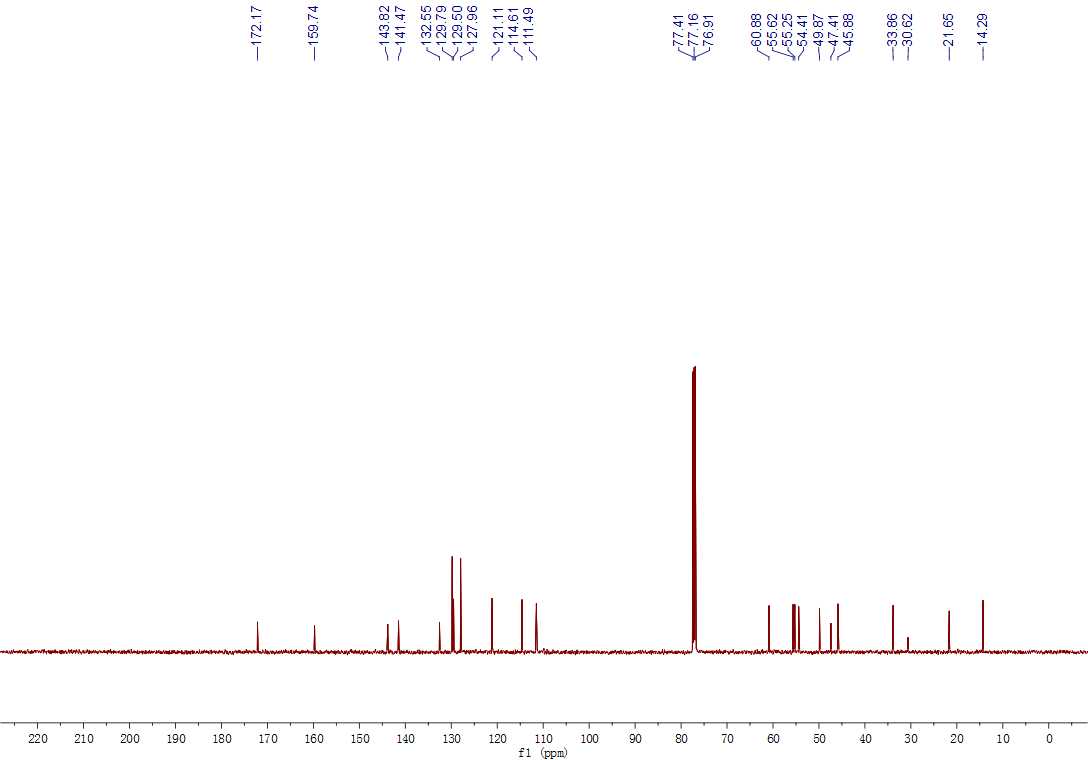


**Supplementary Figure 26.** ^13^C NMR spectrum of compound **26**.


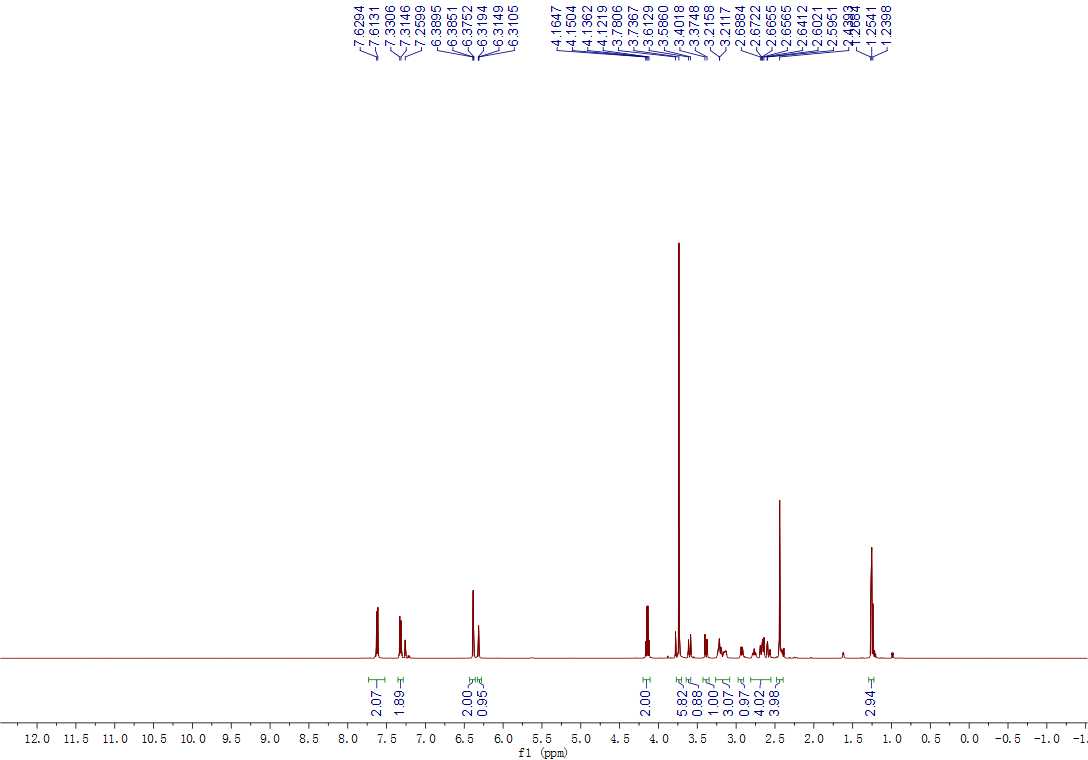


**Supplementary Figure 27.** ^1^H NMR spectrum of compound **27**.


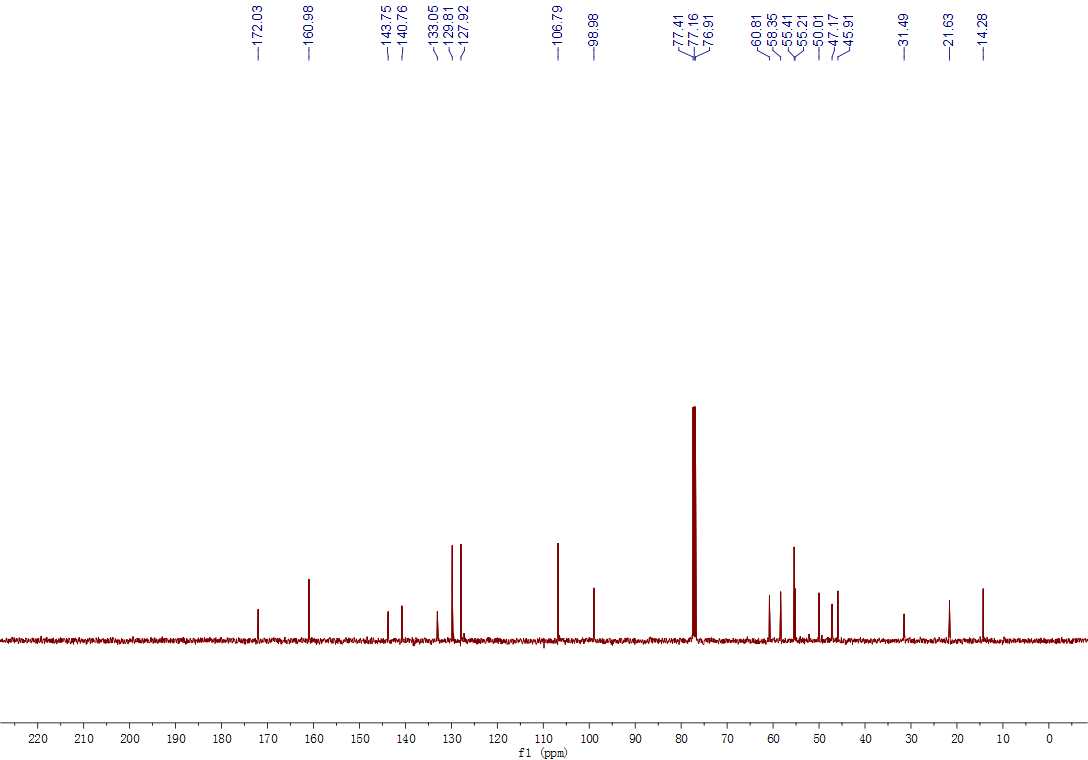


**Supplementary Figure 28.** ^13^C NMR spectrum of compound **27**.


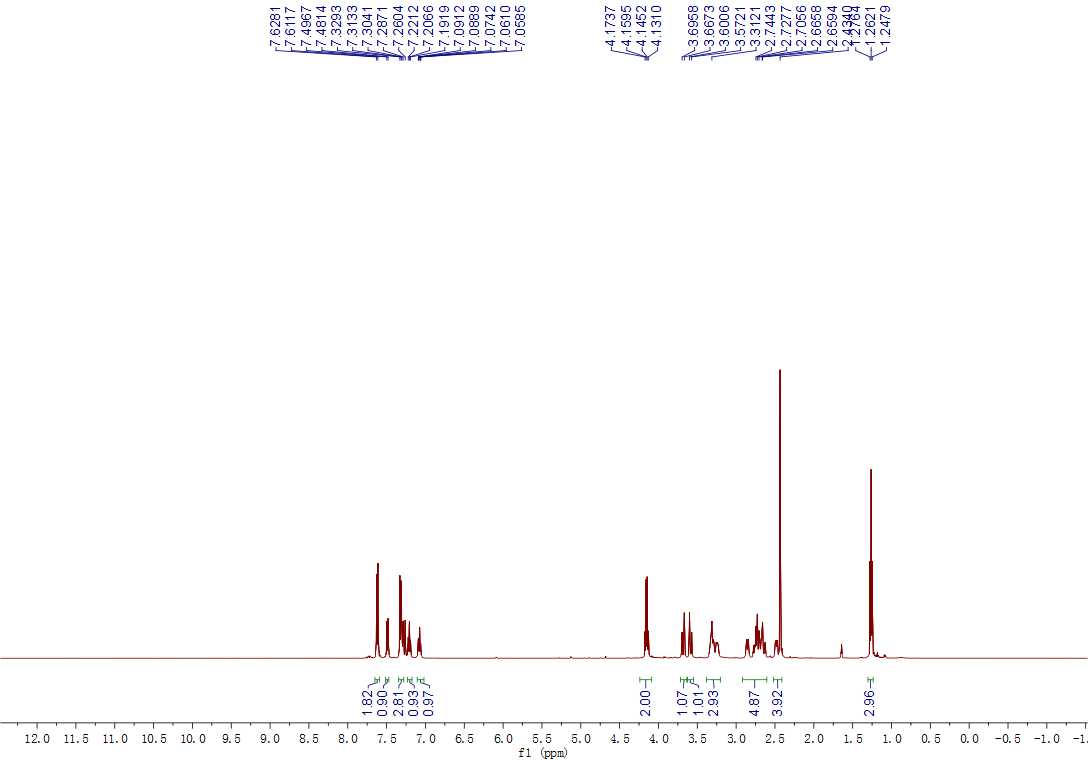


**Supplementary Figure 29.** ^1^H NMR spectrum of compound **28**.


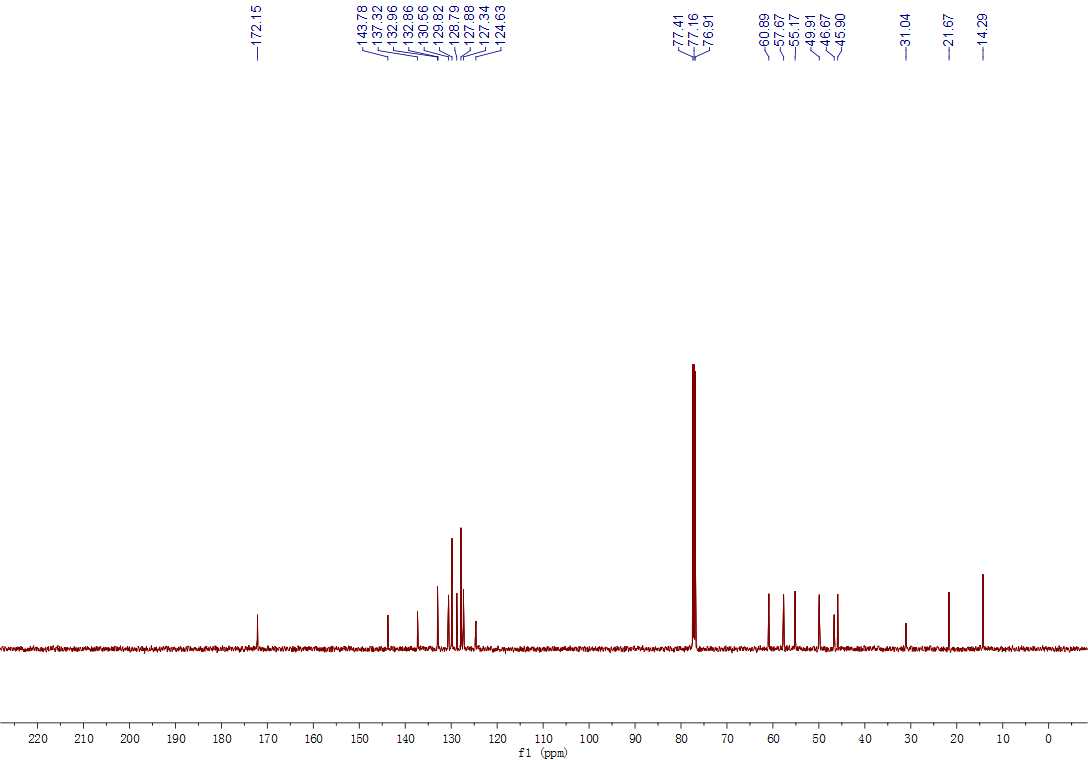


**Supplementary Figure 30.** ^13^C NMR spectrum of compound **28**.


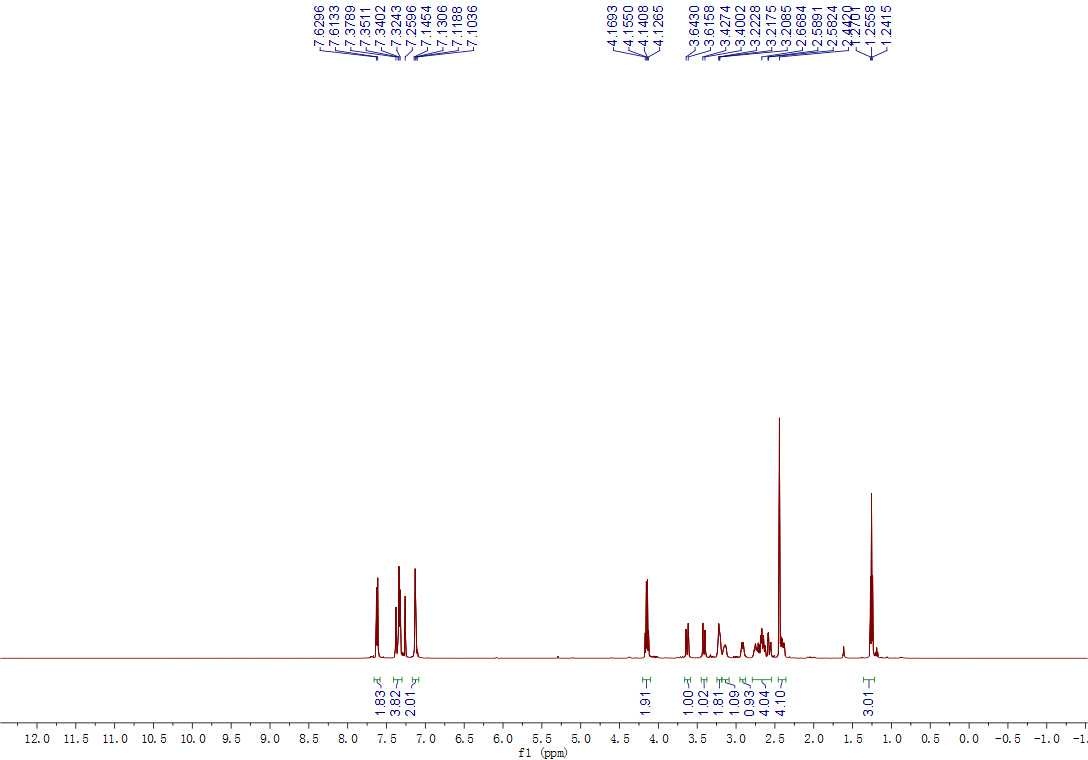


**Supplementary Figure 31.** ^1^H NMR spectrum of compound **29**.


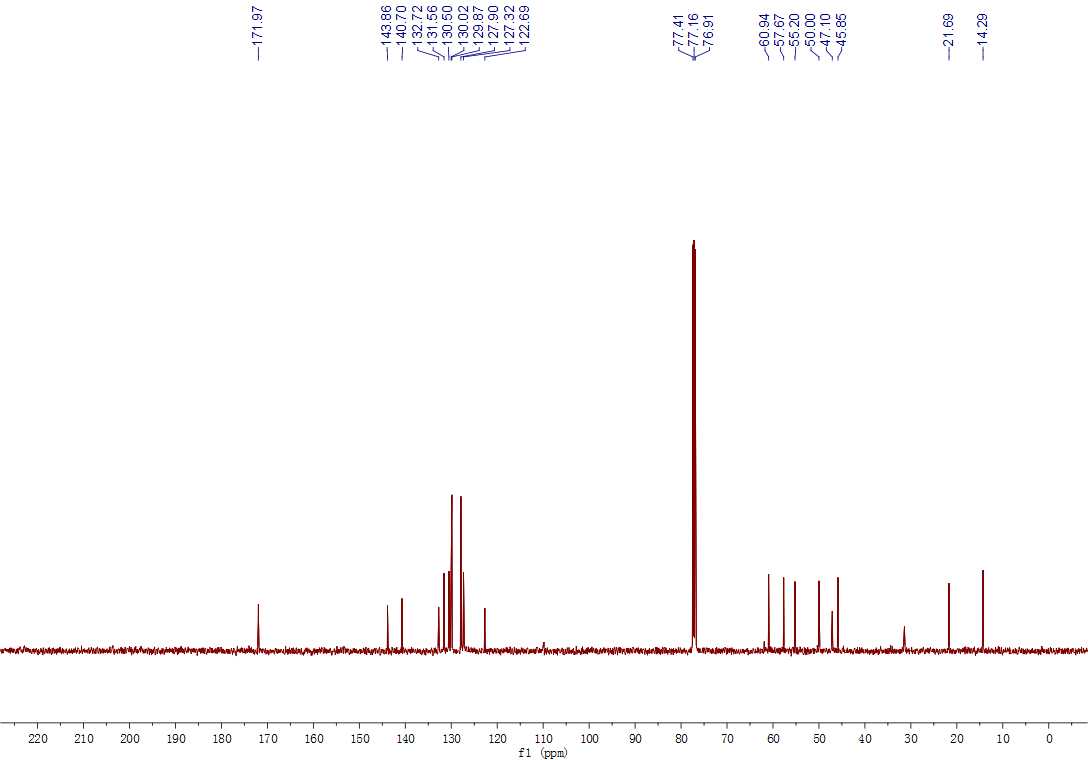


**Supplementary Figure 32.** ^13^C NMR spectrum of compound **29**.


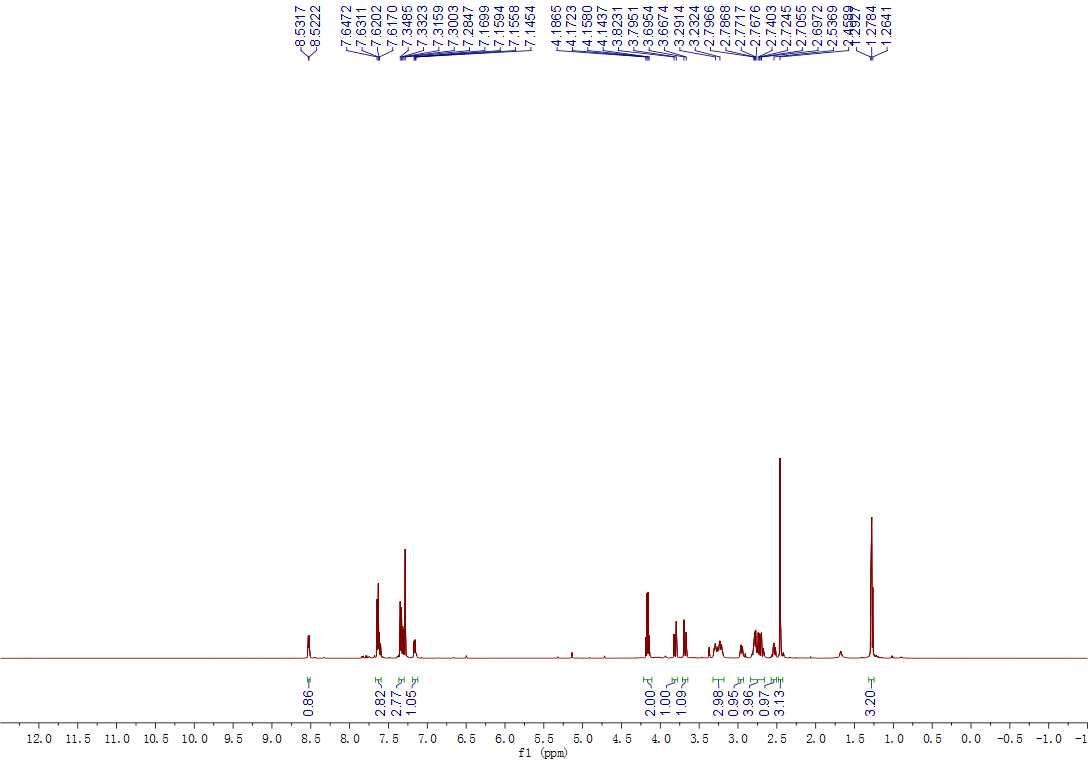


**Supplementary Figure 33.** ^1^H NMR spectrum of compound **30**.


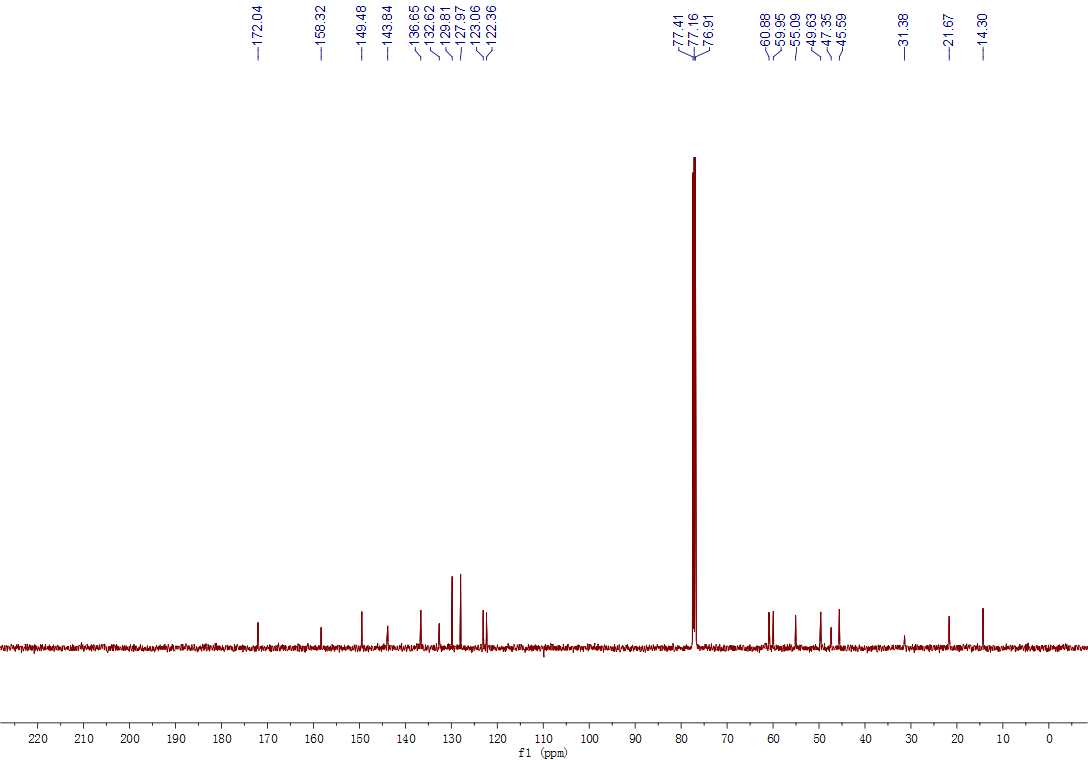


**Supplementary Figure 34.** ^13^C NMR spectrum of compound **30**.


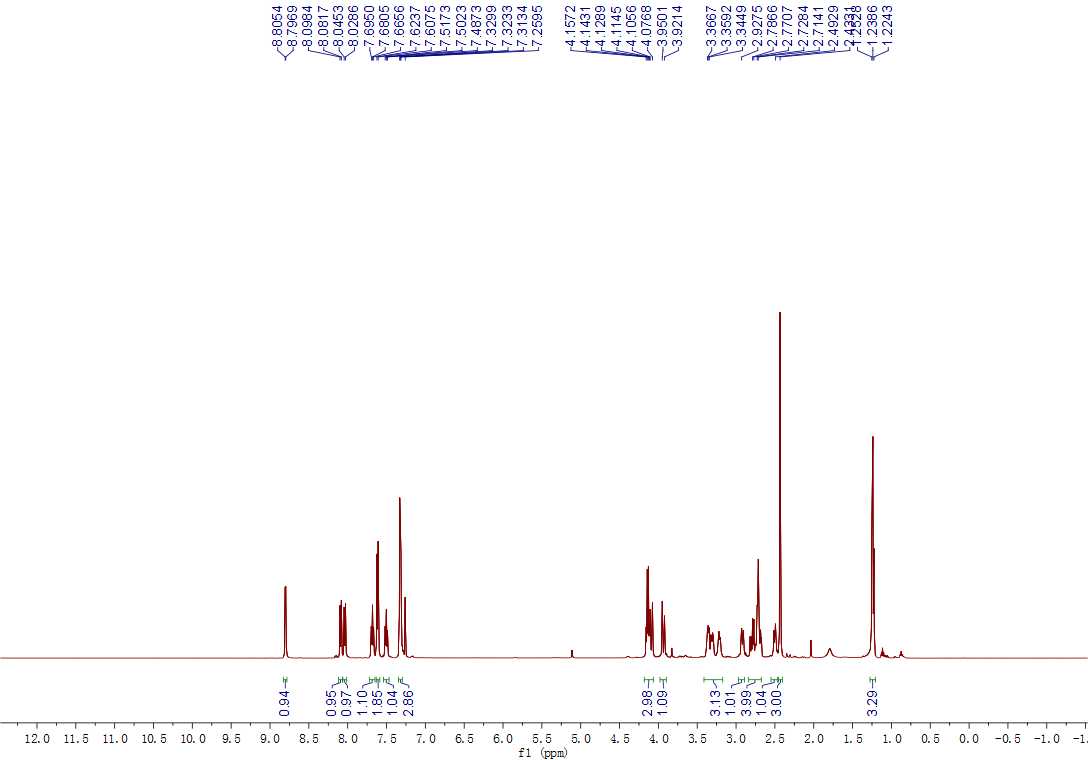


**Supplementary Figure 35.** ^1^H NMR spectrum of compound **31**.


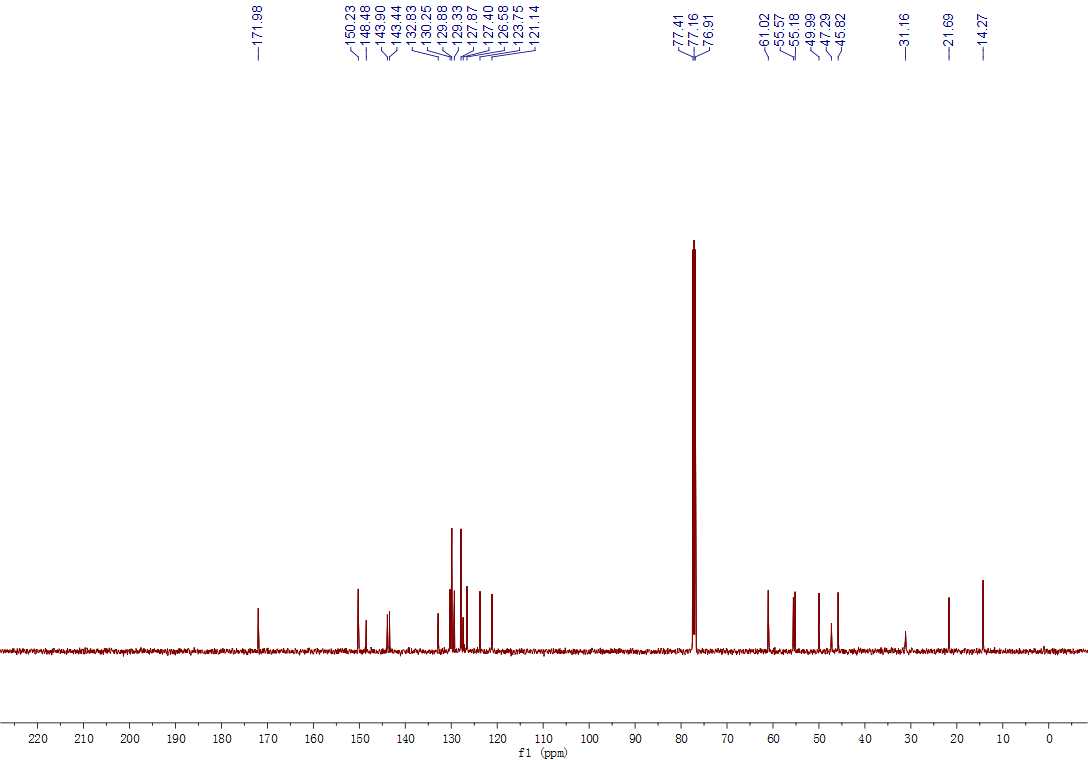


**Supplementary Figure 36.** ^13^C NMR spectrum of compound **31**.


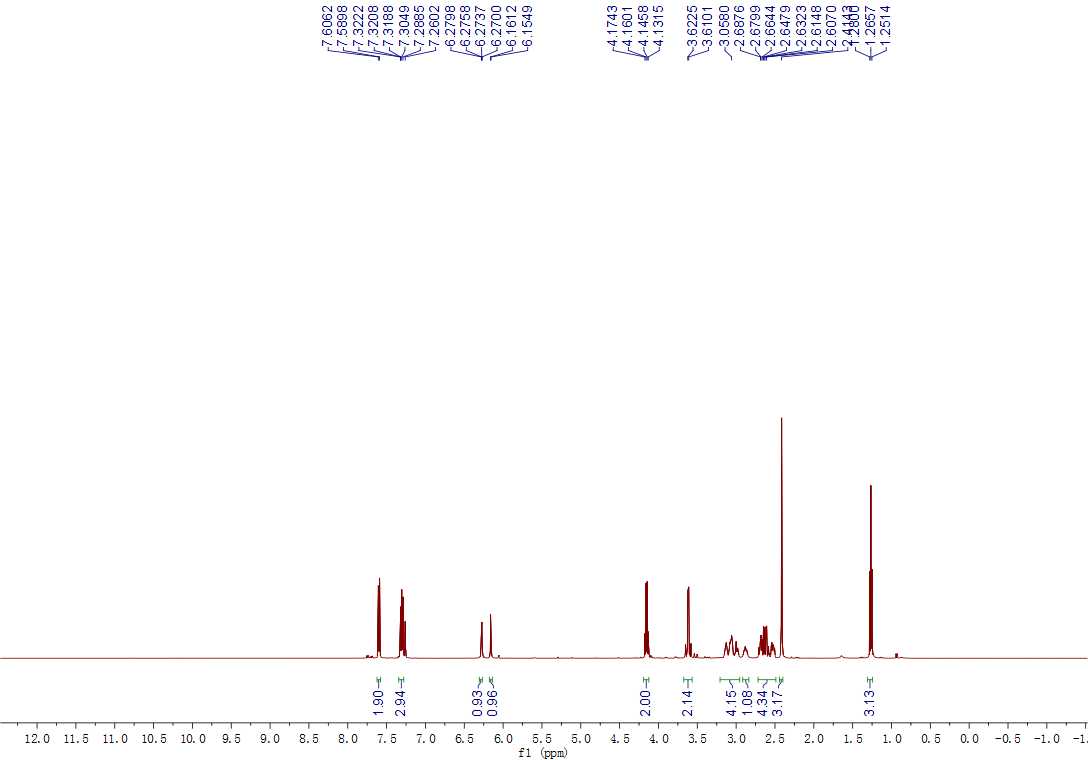


**Supplementary Figure 37.** ^1^H NMR spectrum of compound **32**.


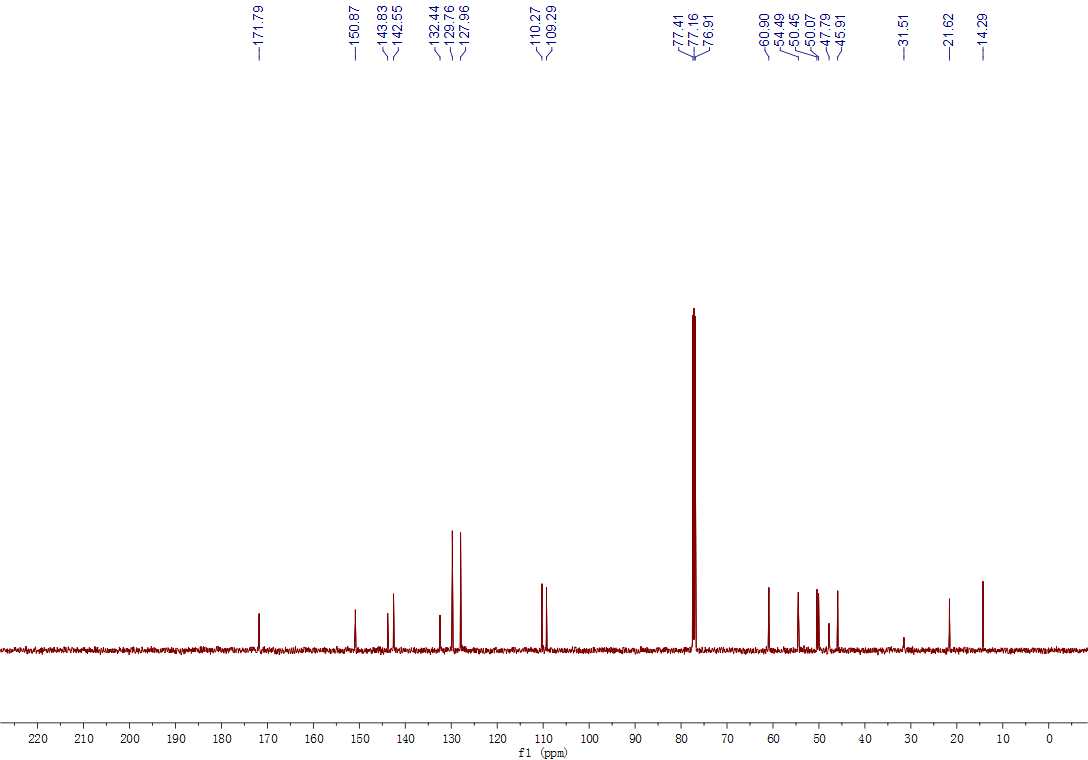
**Supplementary Figure 38.** ^13^C NMR spectrum of compound **32**.


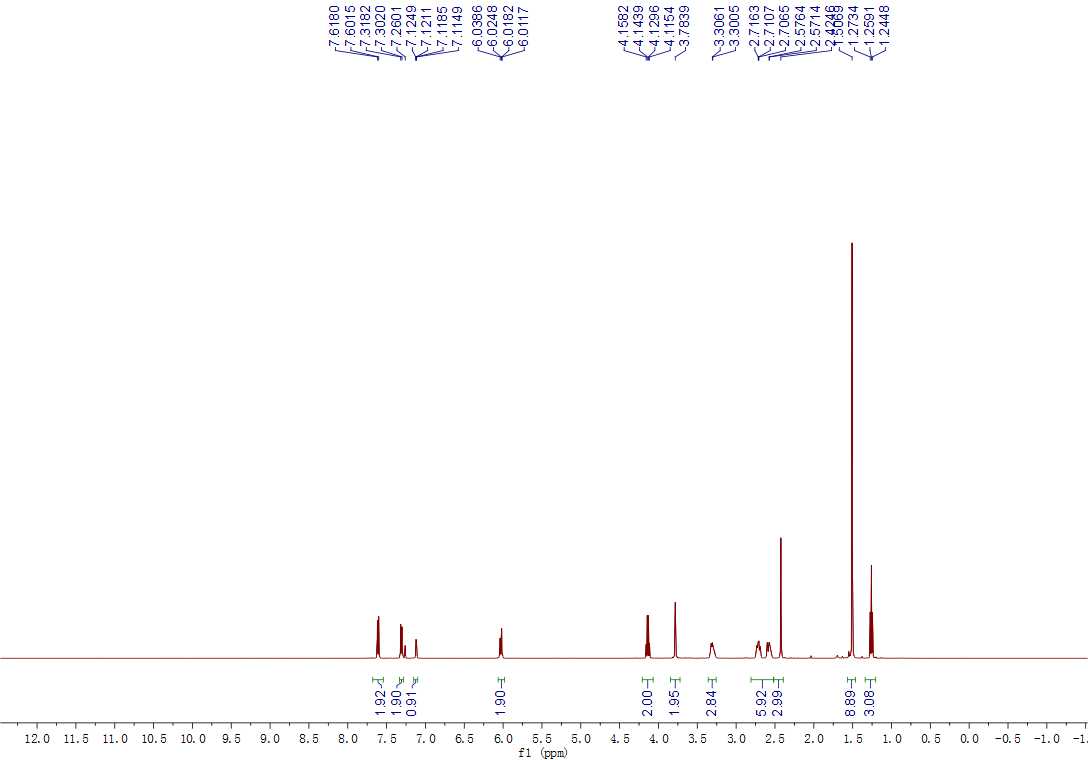


**Supplementary Figure 39.** ^1^H NMR spectrum of compound **33**.


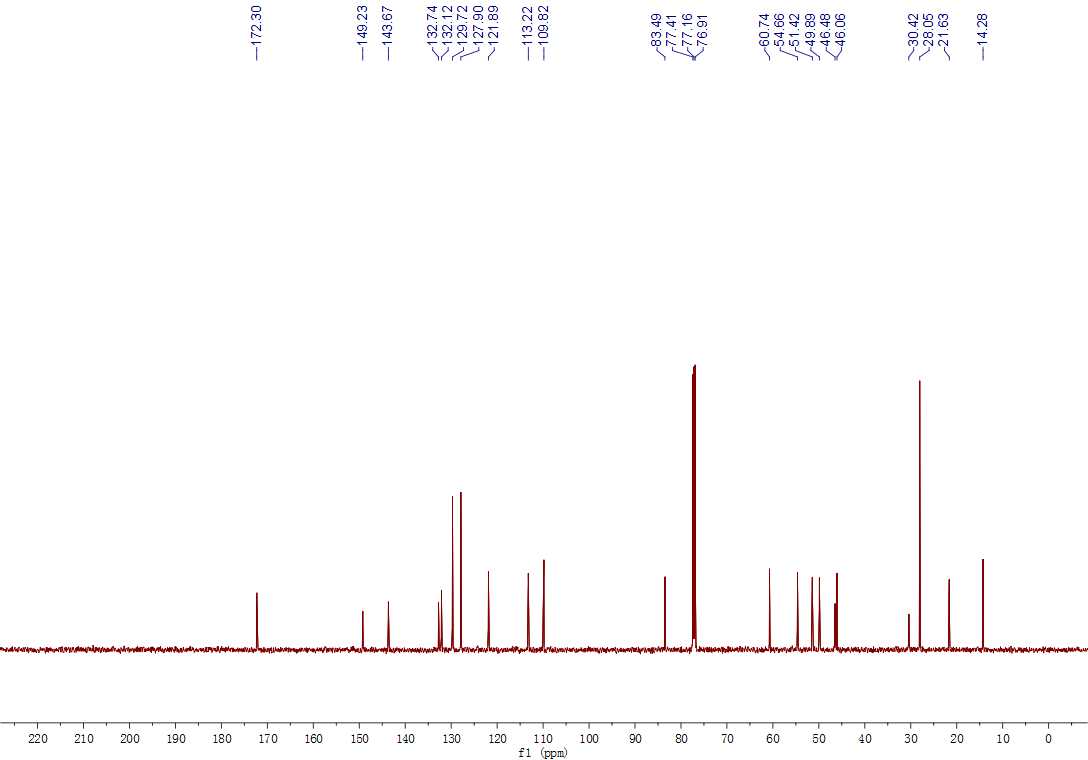


**Supplementary Figure 40.** ^13^C NMR spectrum of compound **33**.


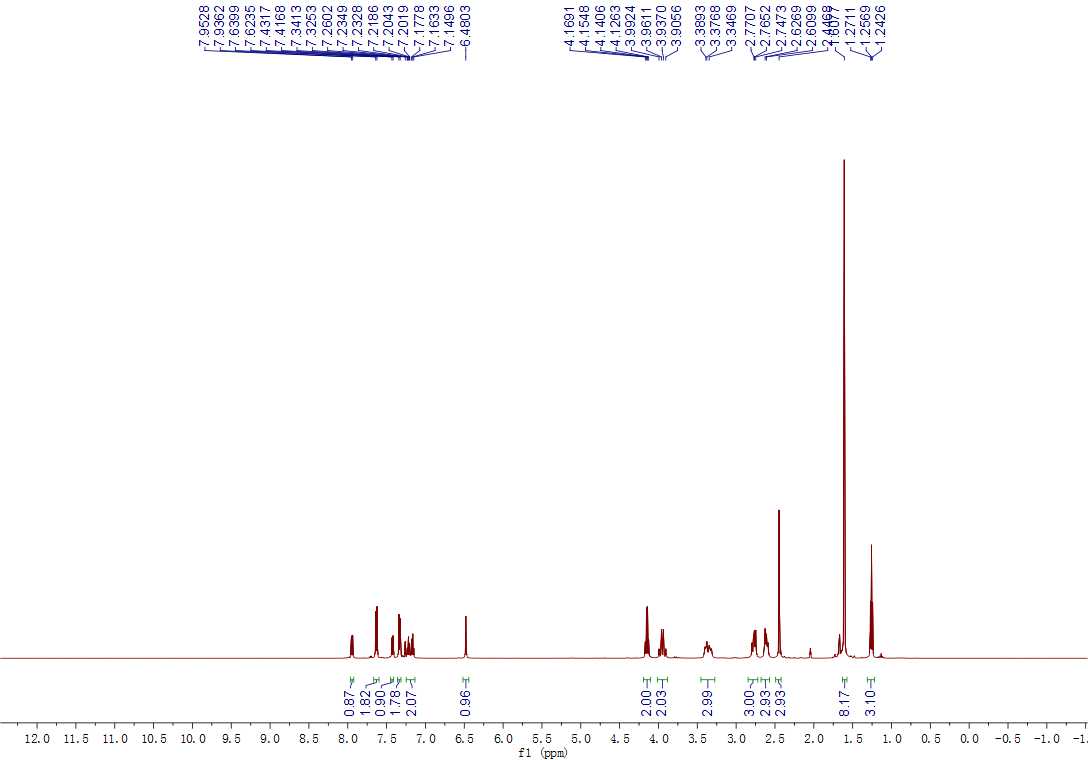


**Supplementary Figure 41.** ^1^H NMR spectrum of compound **34**.


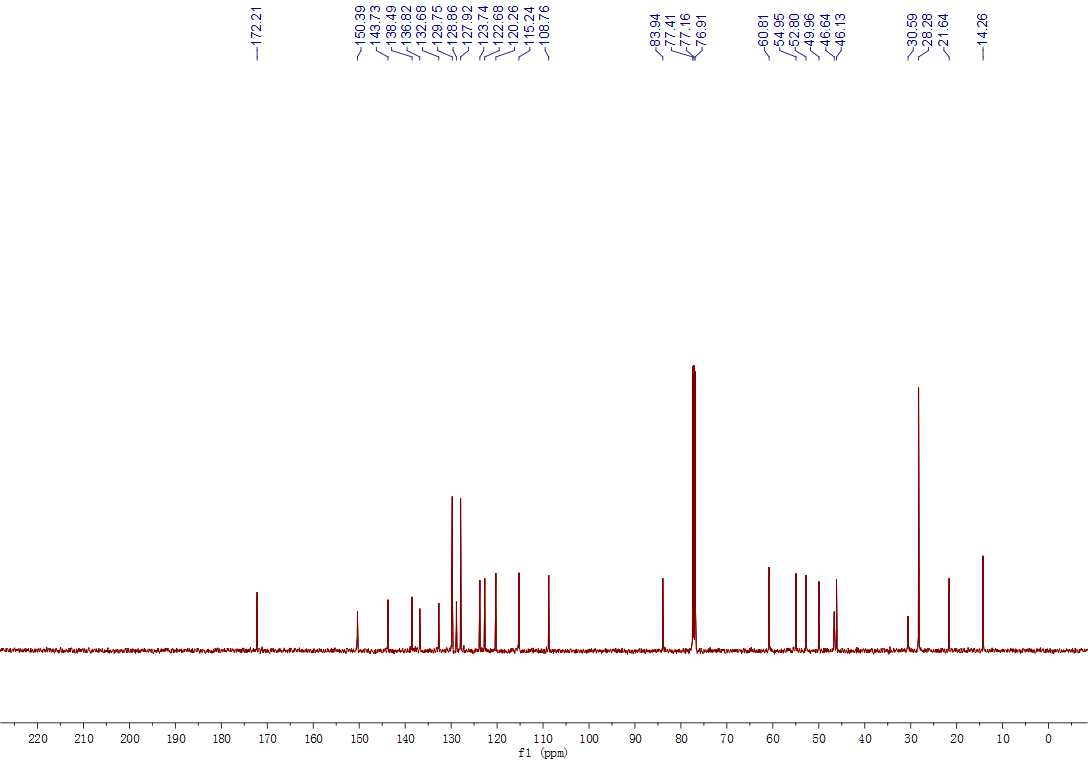


**Supplementary Figure 42.** ^13^C NMR spectrum of compound **34**.


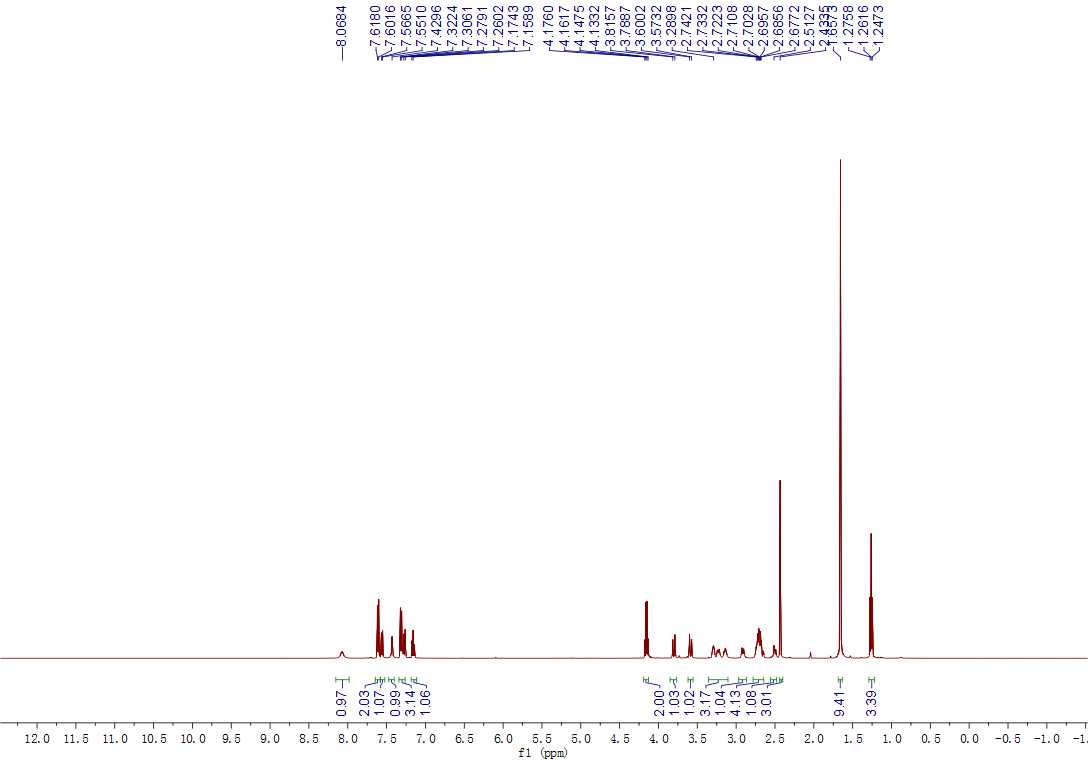


**Supplementary Figure 43.** ^1^H NMR spectrum of compound **35**.


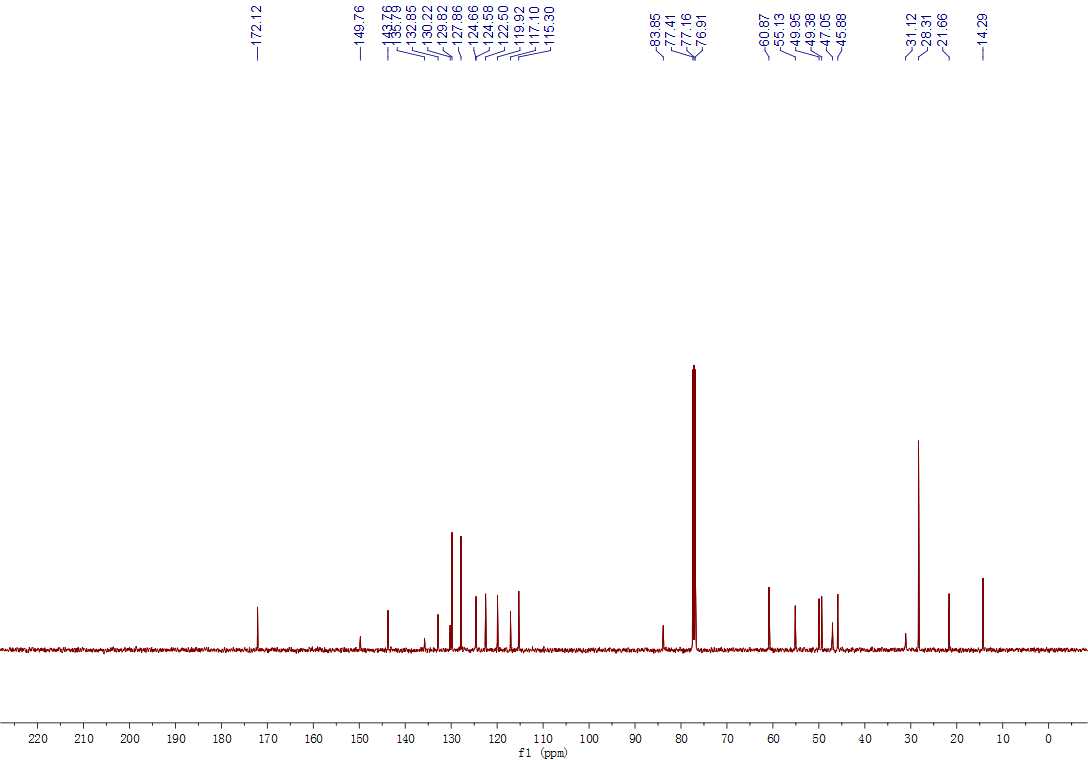


**Supplementary Figure 44.** ^13^C NMR spectrum of compound **35**.


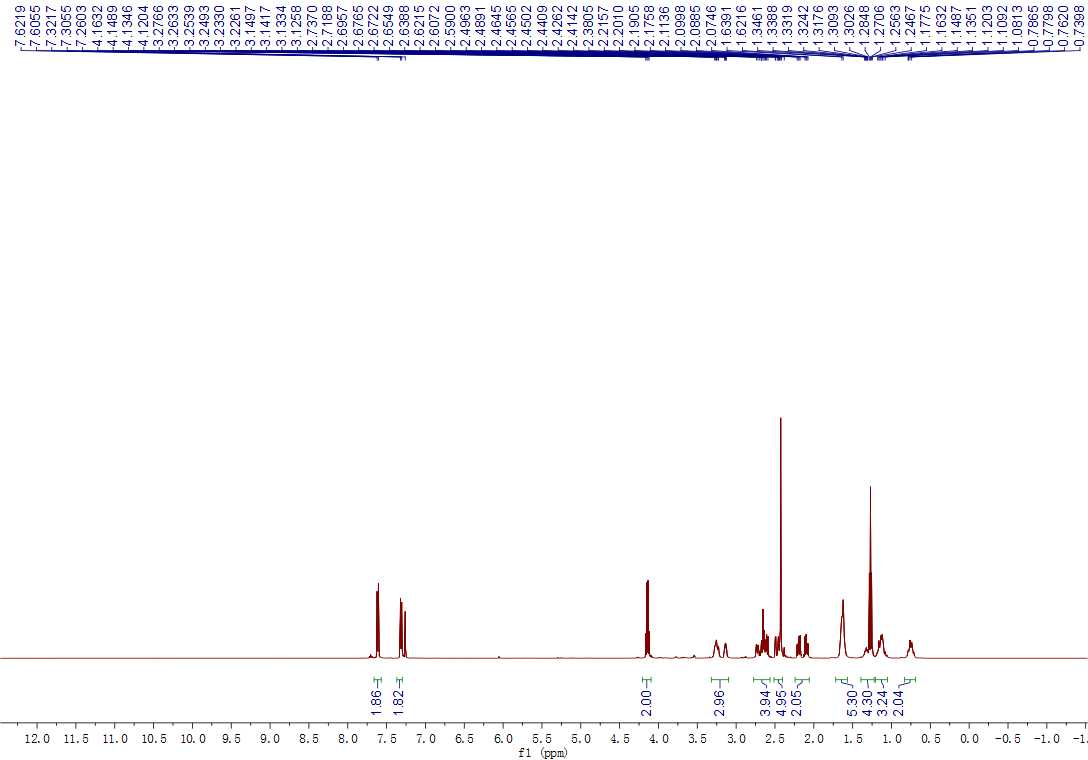


**Supplementary Figure 45.** ^1^H NMR spectrum of compound **36**.


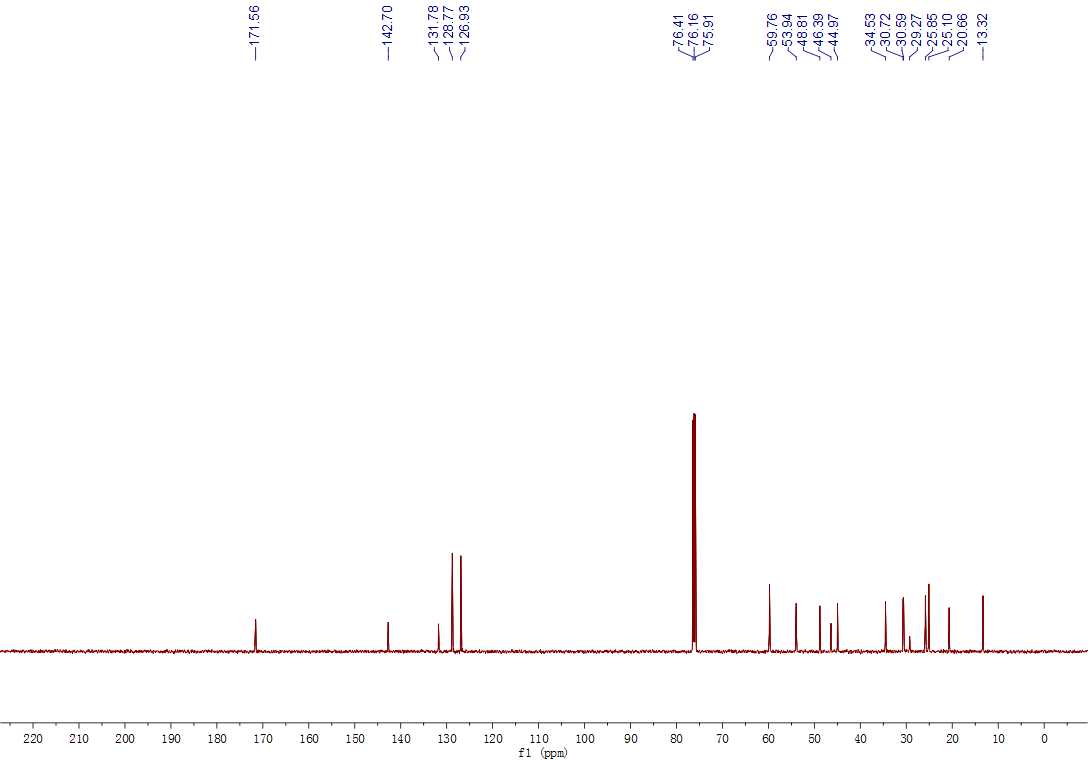


**Supplementary Figure 46.** ^13^C NMR spectrum of compound **36**.


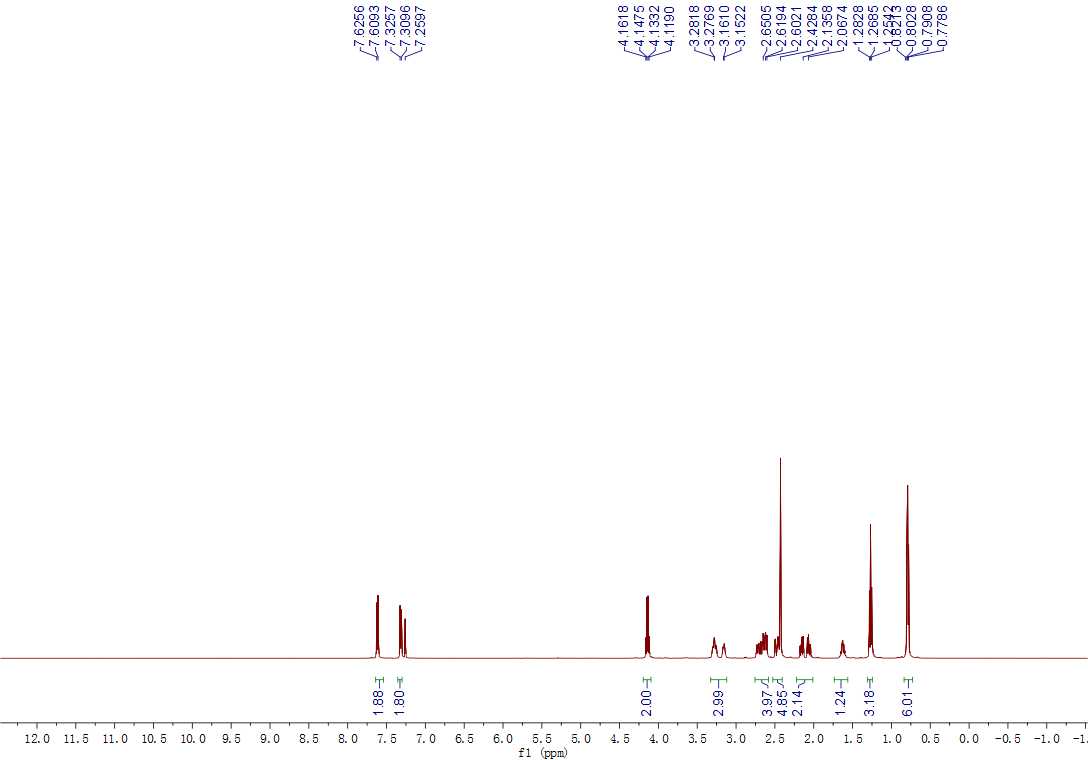


**Supplementary Figure 47.** ^1^H NMR spectrum of compound **37**.


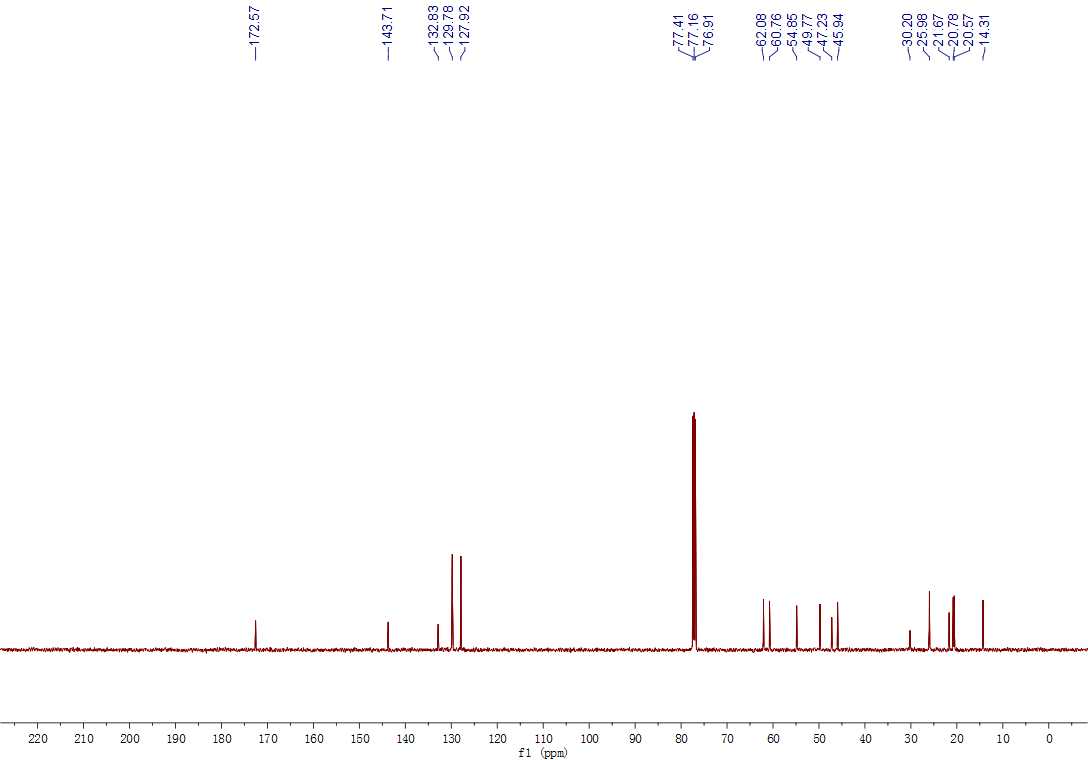


**Supplementary Figure 48.** ^13^C NMR spectrum of compound **37**.


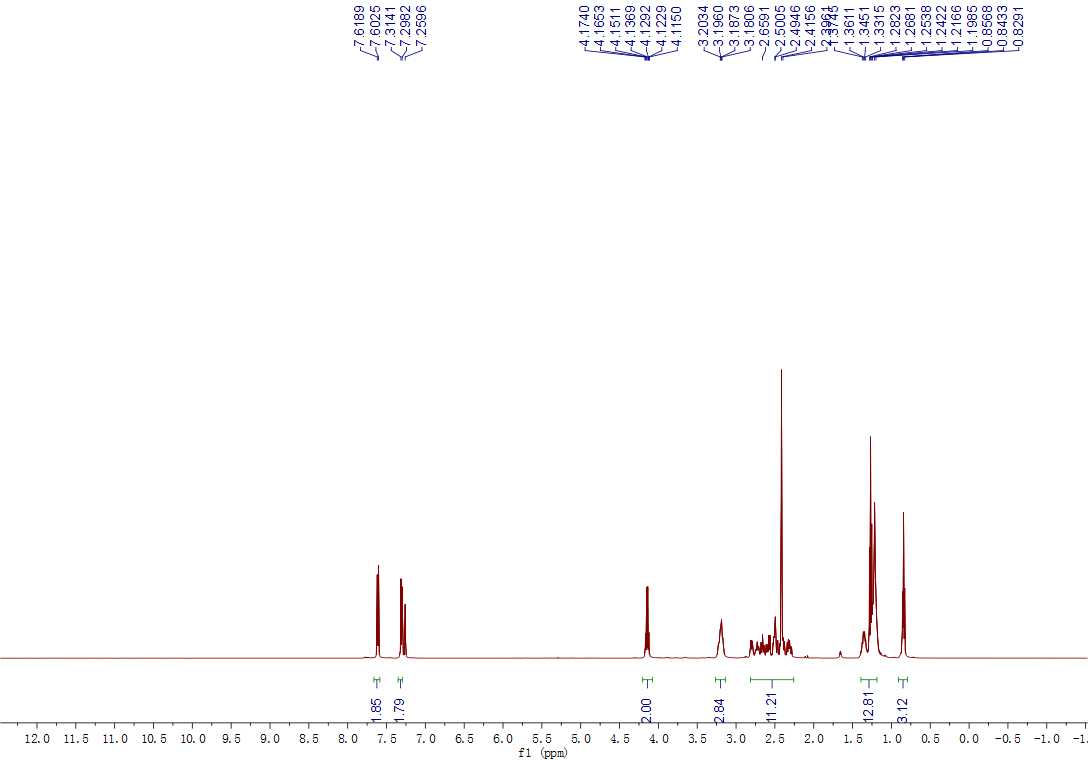


**Supplementary Figure 49.** ^1^H NMR spectrum of compound **38**.


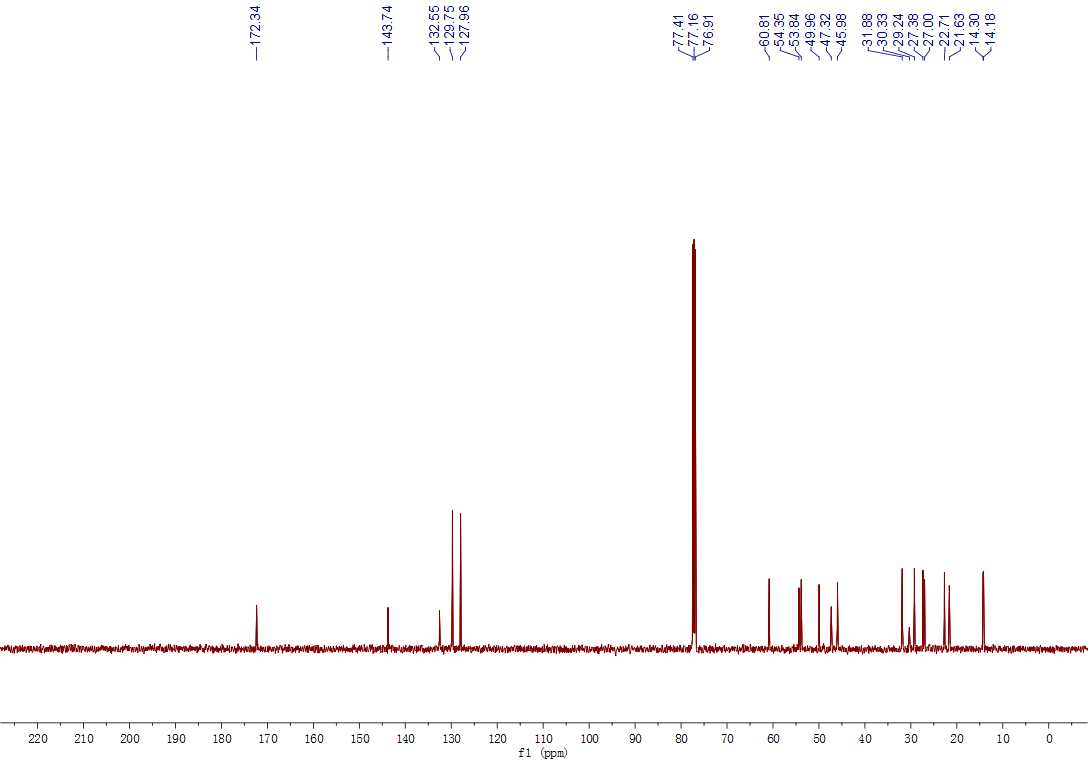


**Supplementary Figure 50.** ^13^C NMR spectrum of compound **38**.


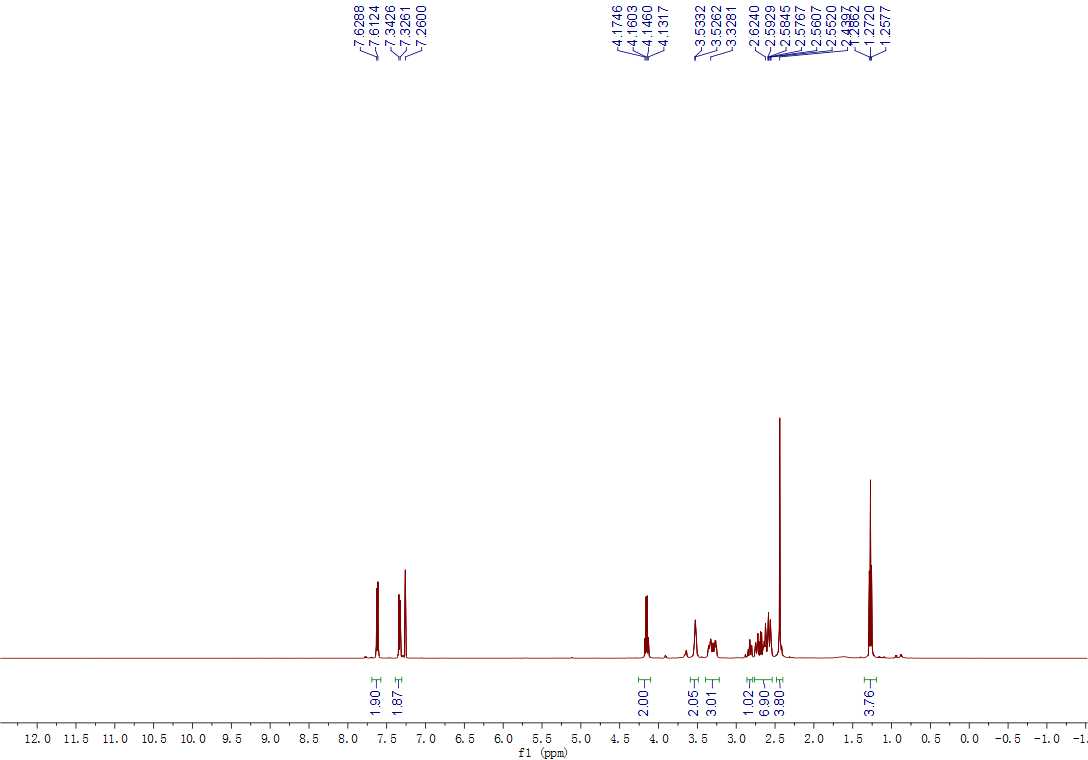


**Supplementary Figure 51.** ^1^H NMR spectrum of compound **39**.


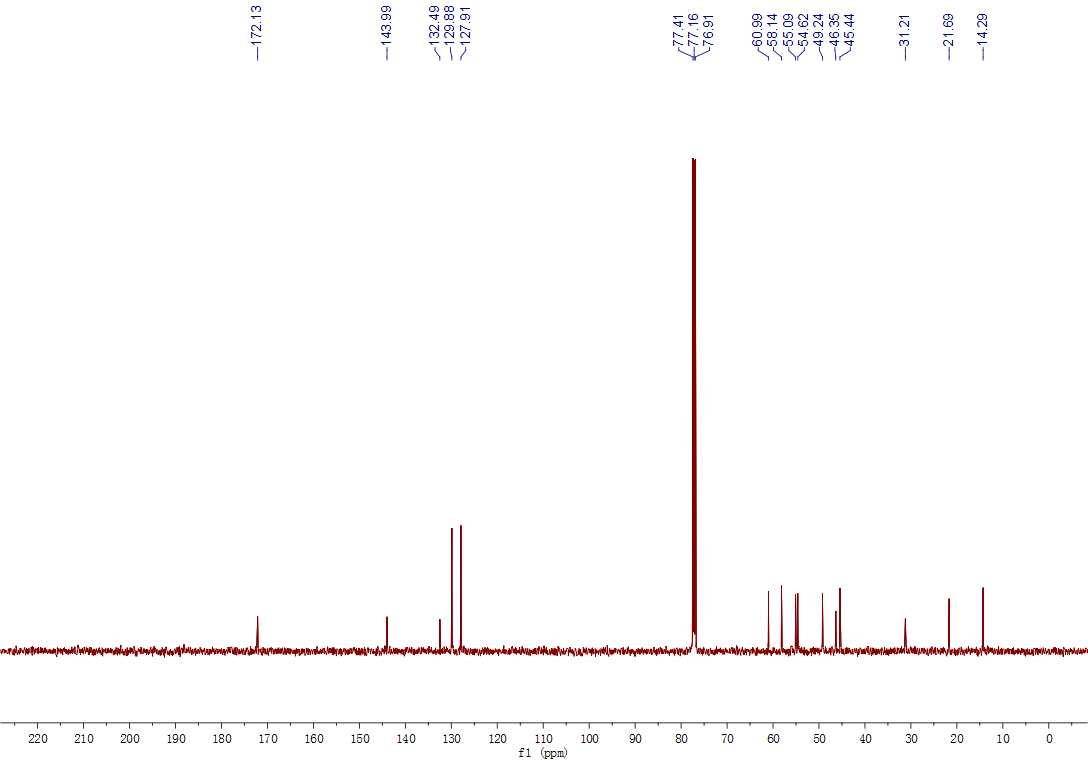


**Supplementary Figure 52.** ^13^C NMR spectrum of compound **39**.


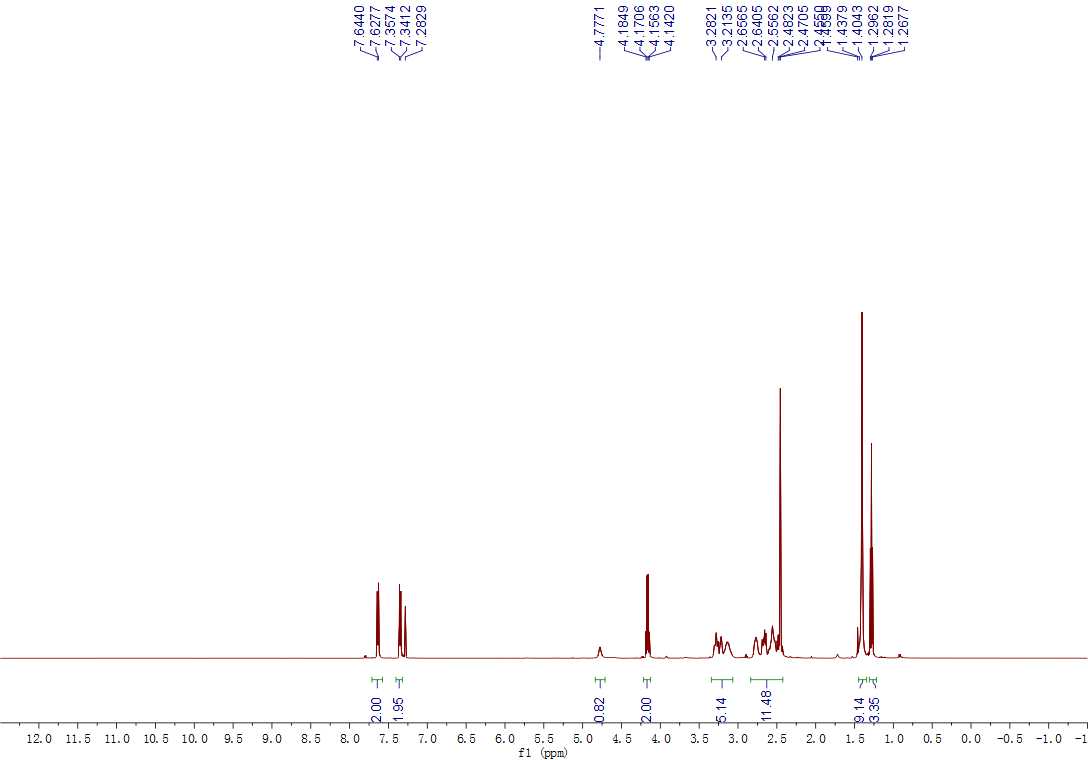


**Supplementary Figure 53.** ^1^H NMR spectrum of compound **40**.


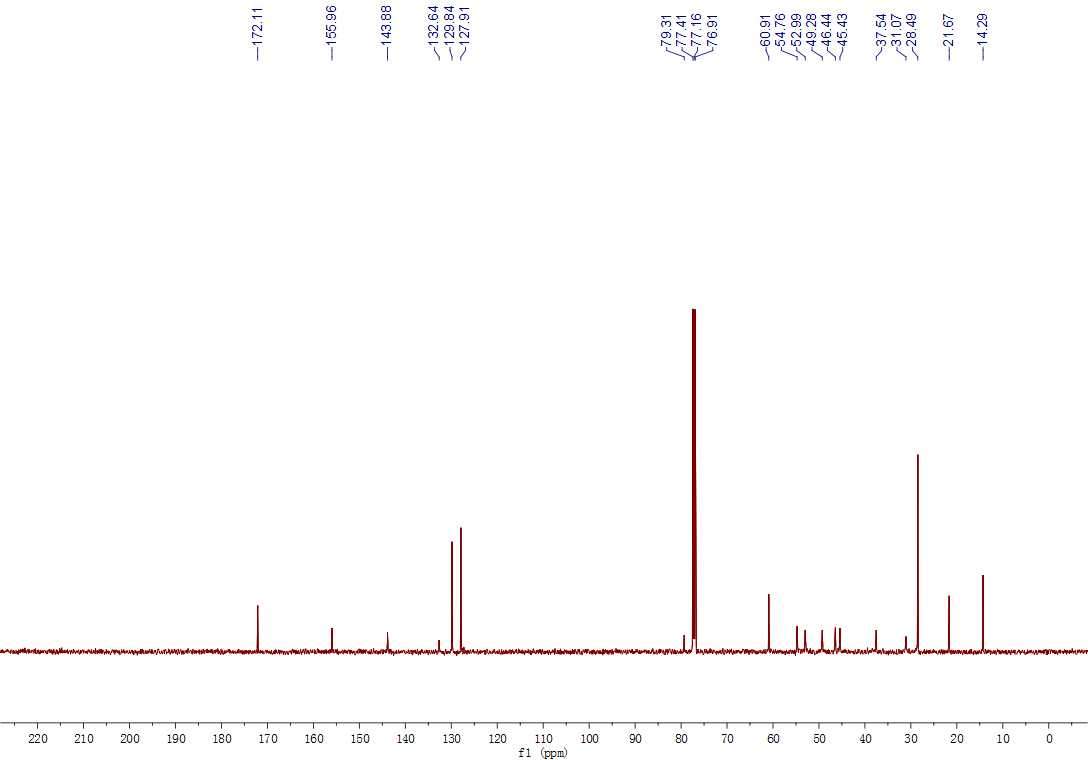


**Supplementary Figure 54.** ^13^C NMR spectrum of compound **40**.


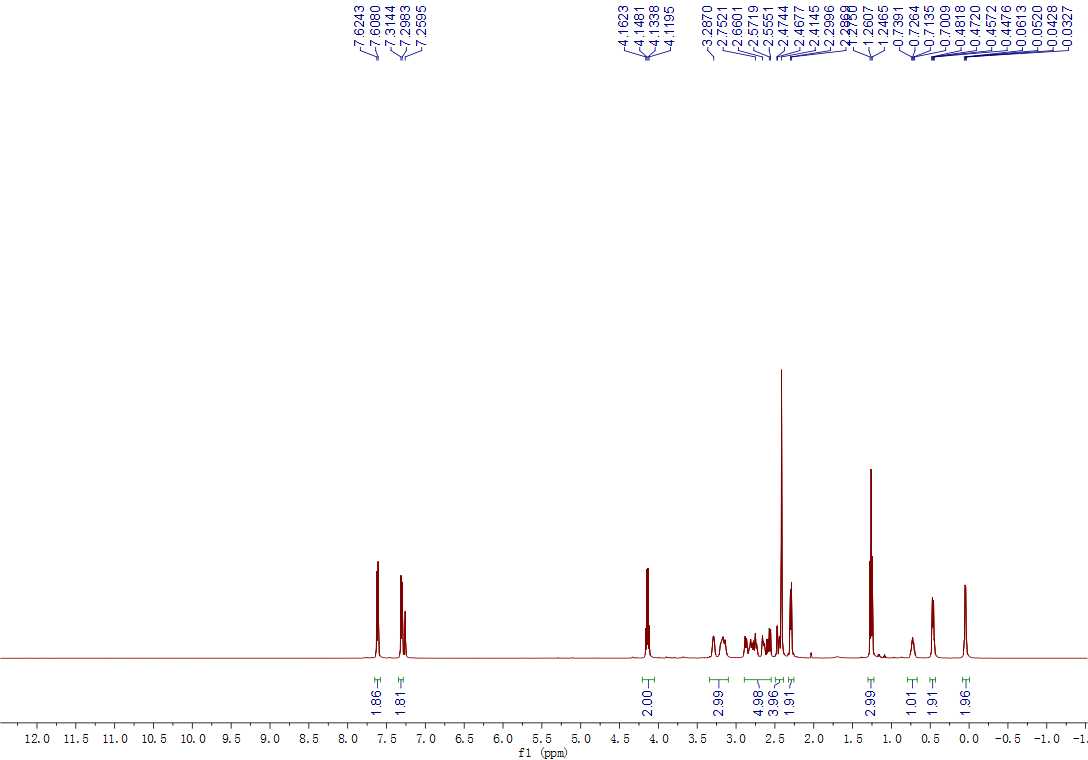


**Supplementary Figure 55.** ^1^H NMR spectrum of compound **41**.


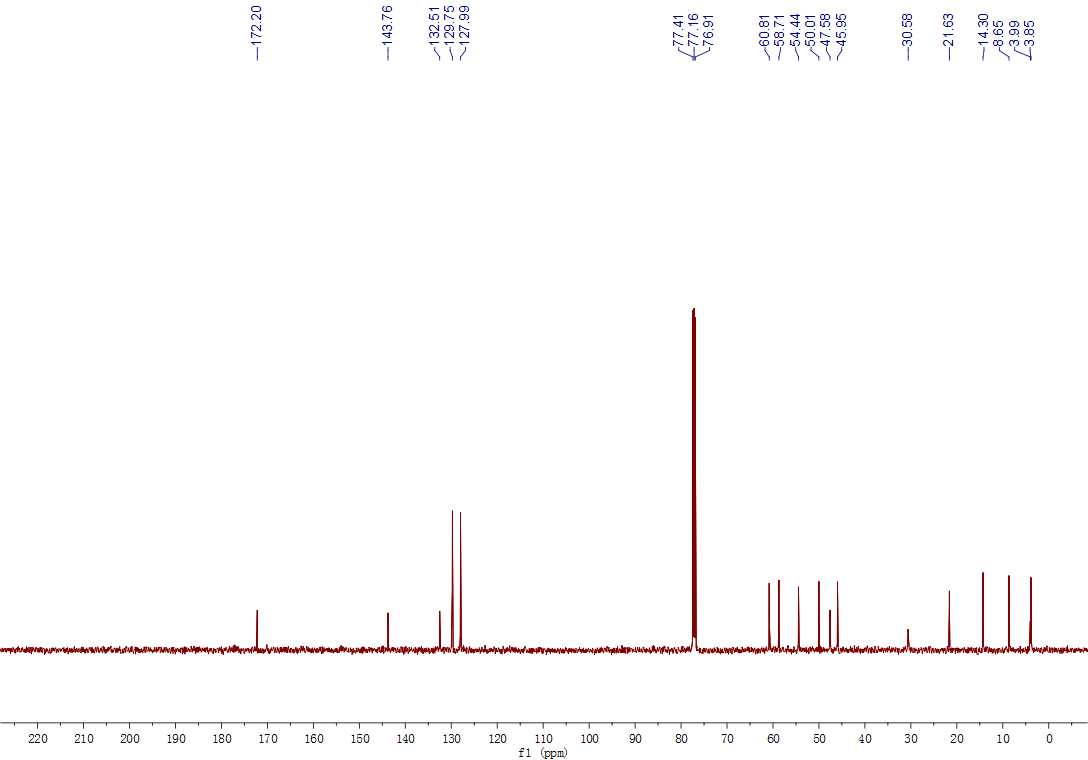


**Supplementary Figure 56.** ^13^C NMR spectrum of compound **41**.


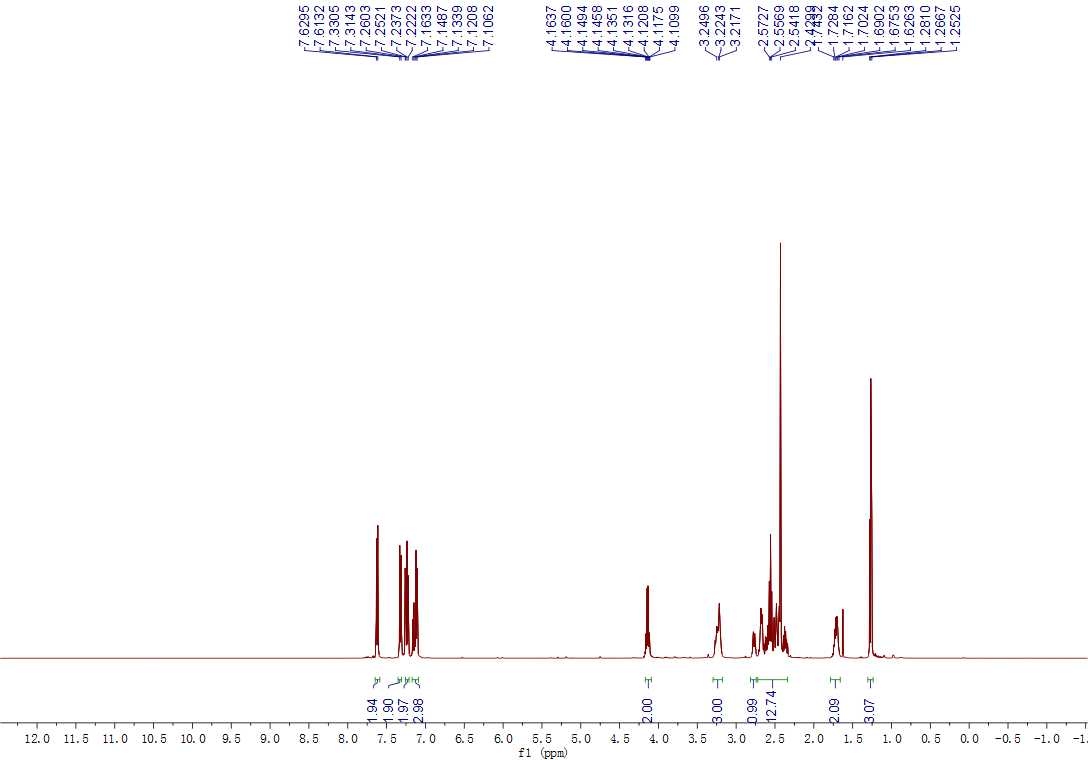


**Supplementary Figure 57.** ^1^H NMR spectrum of compound **42**.


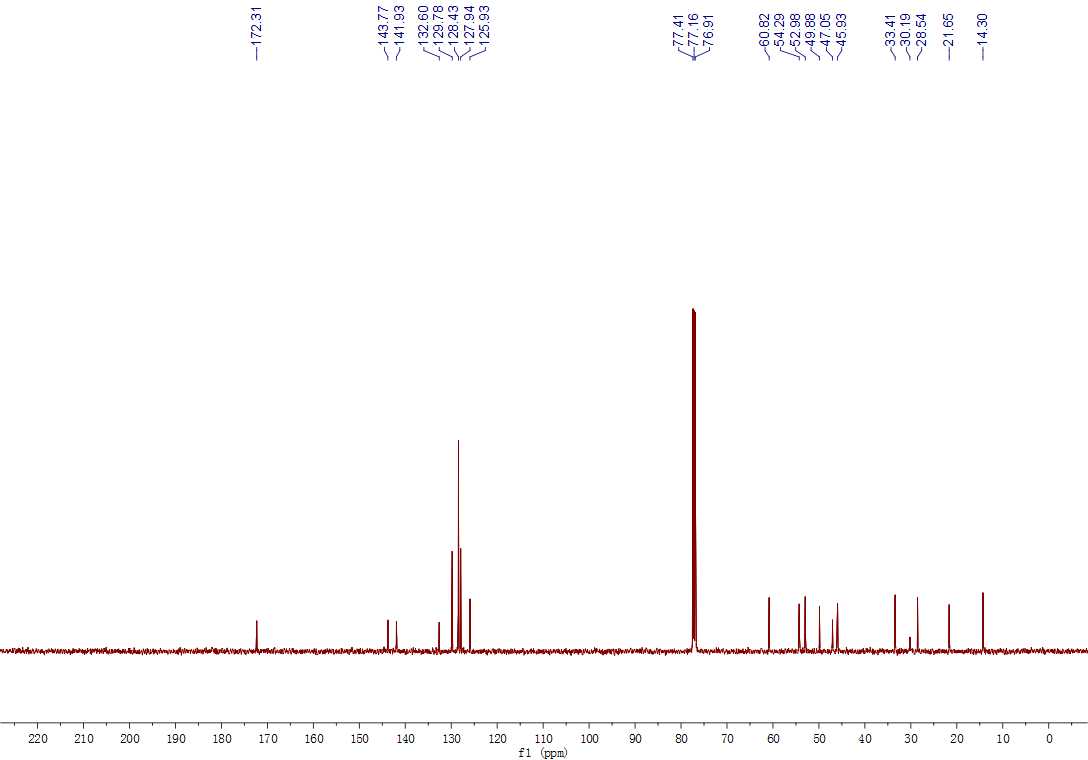
**Supplementary Figure 58.** ^13^C NMR spectrum of compound **42**.


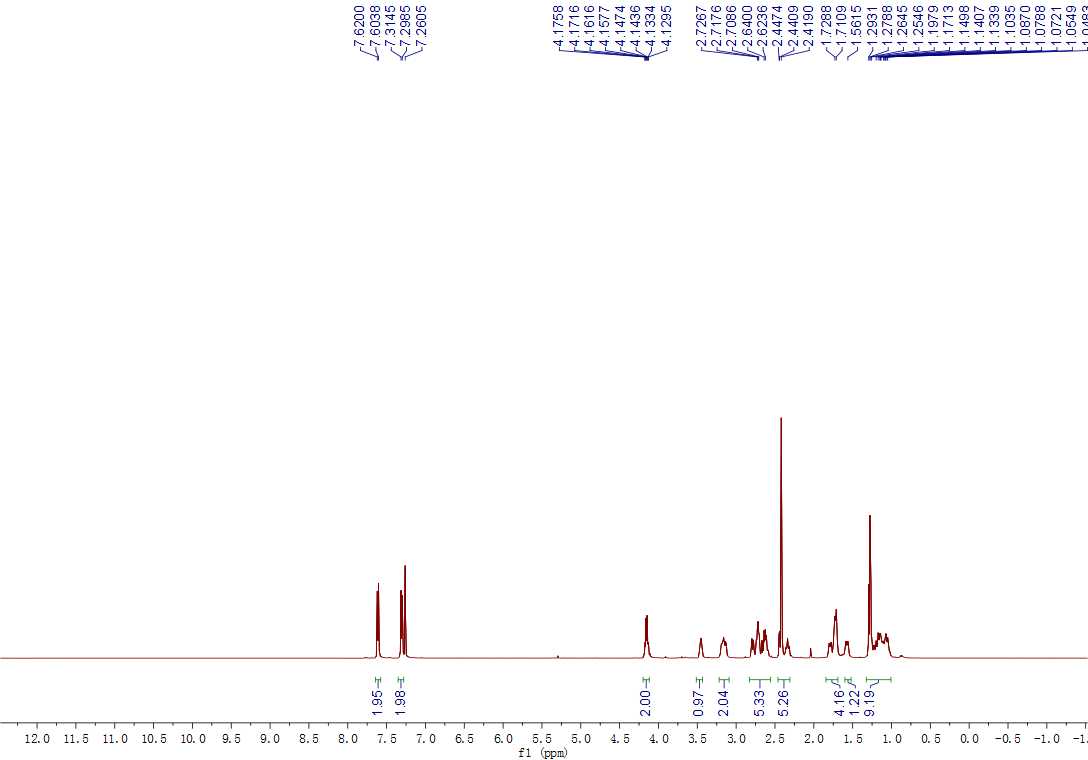


**Supplementary Figure 59.** ^1^H NMR spectrum of compound **43**.


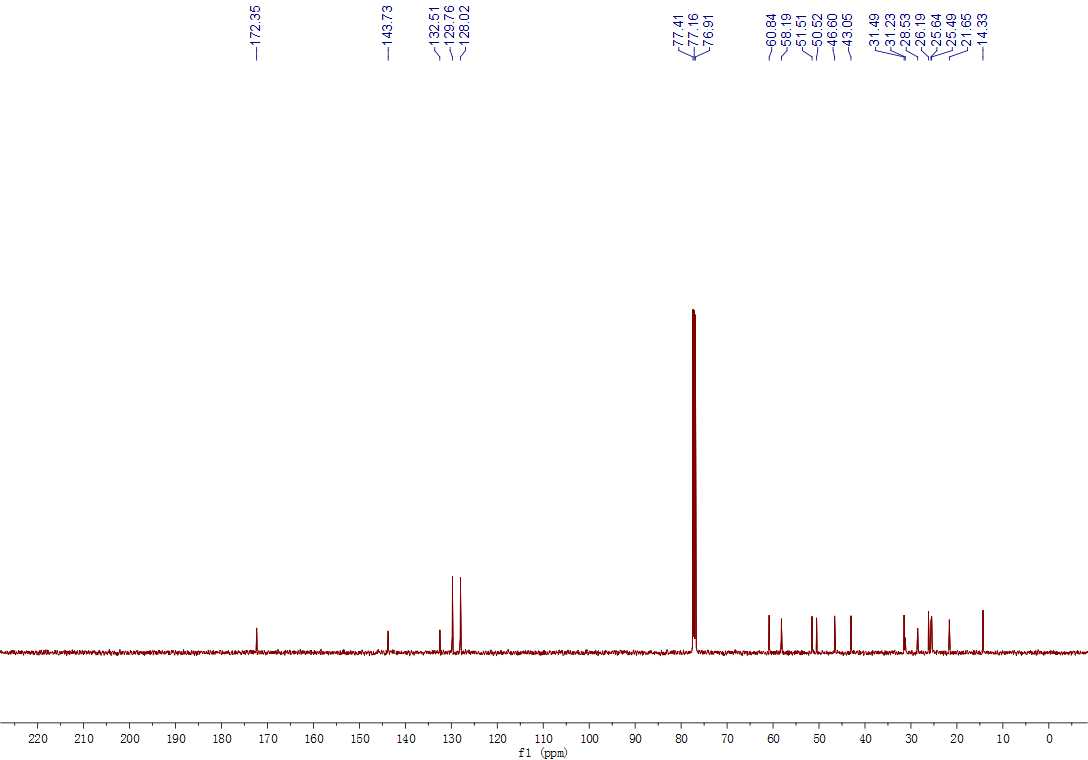


**Supplementary Figure 60.** ^13^C NMR spectrum of compound **43**.


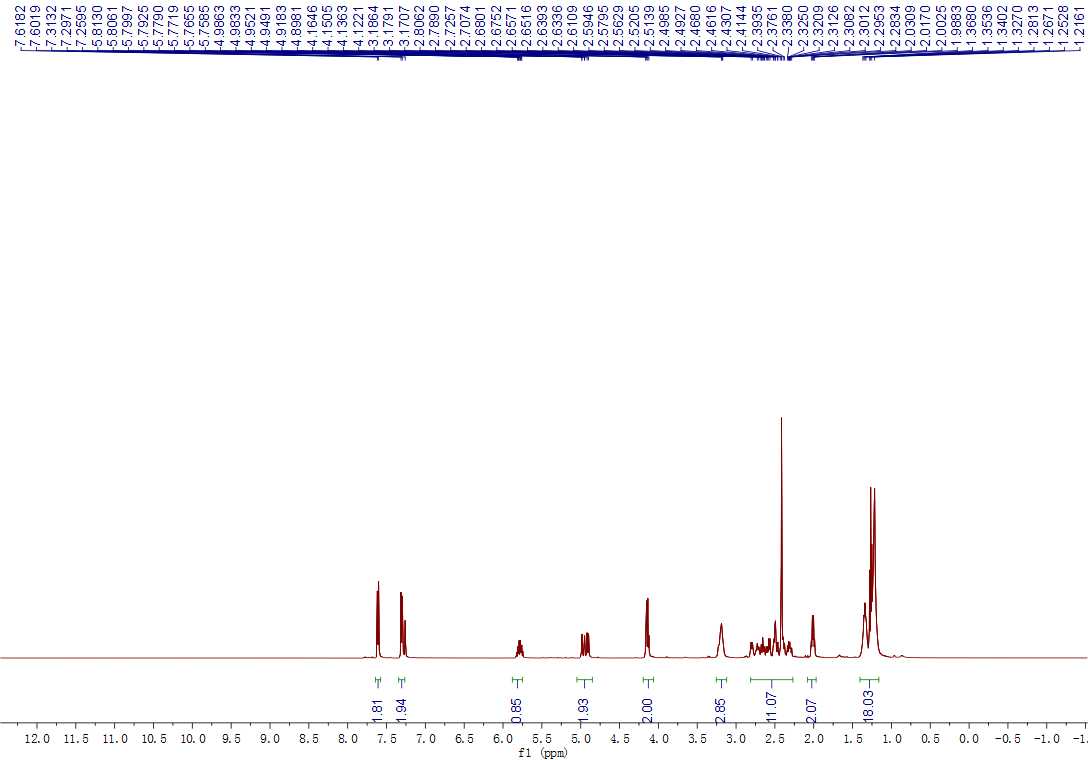


**Supplementary Figure 61.** ^1^H NMR spectrum of compound **44**.


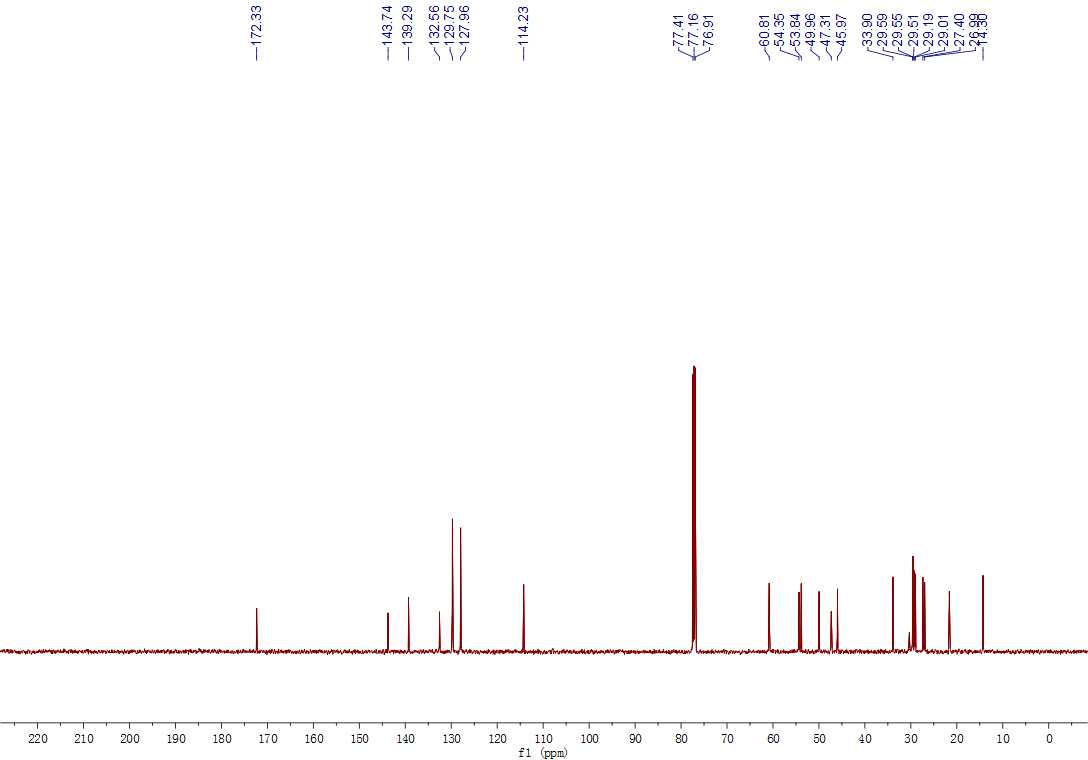


**Supplementary Figure 62.** ^13^C NMR spectrum of compound **44**.


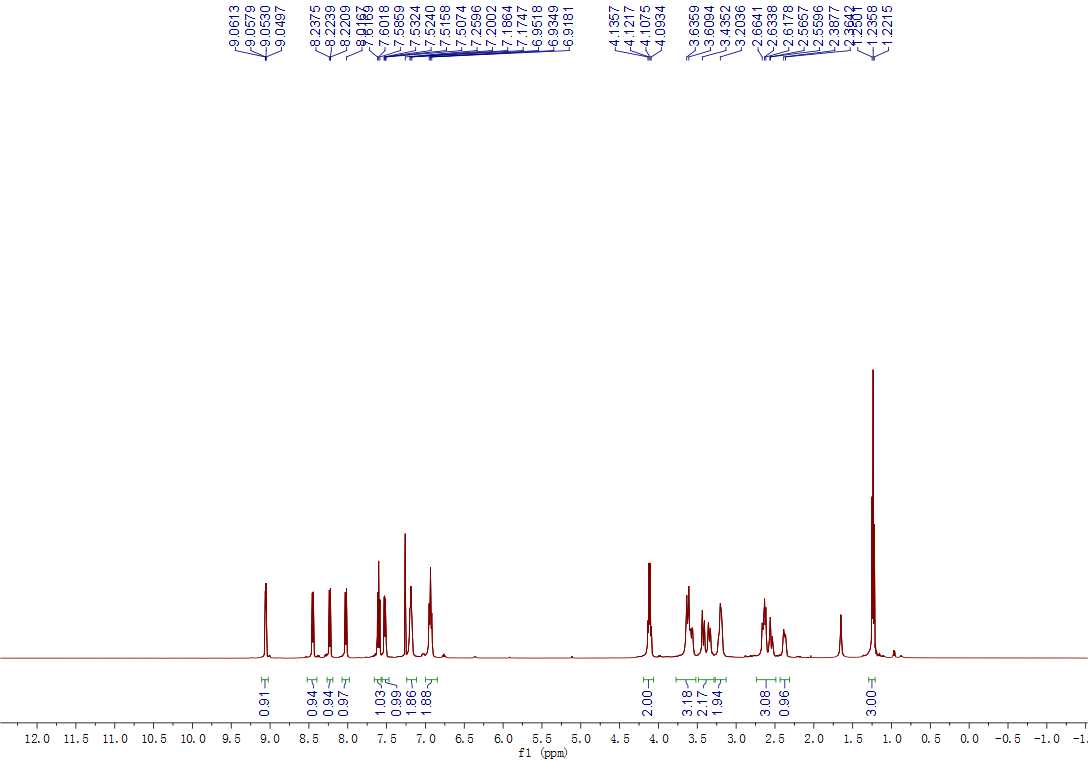


**Supplementary Figure 63.** ^1^H NMR spectrum of compound **45**.


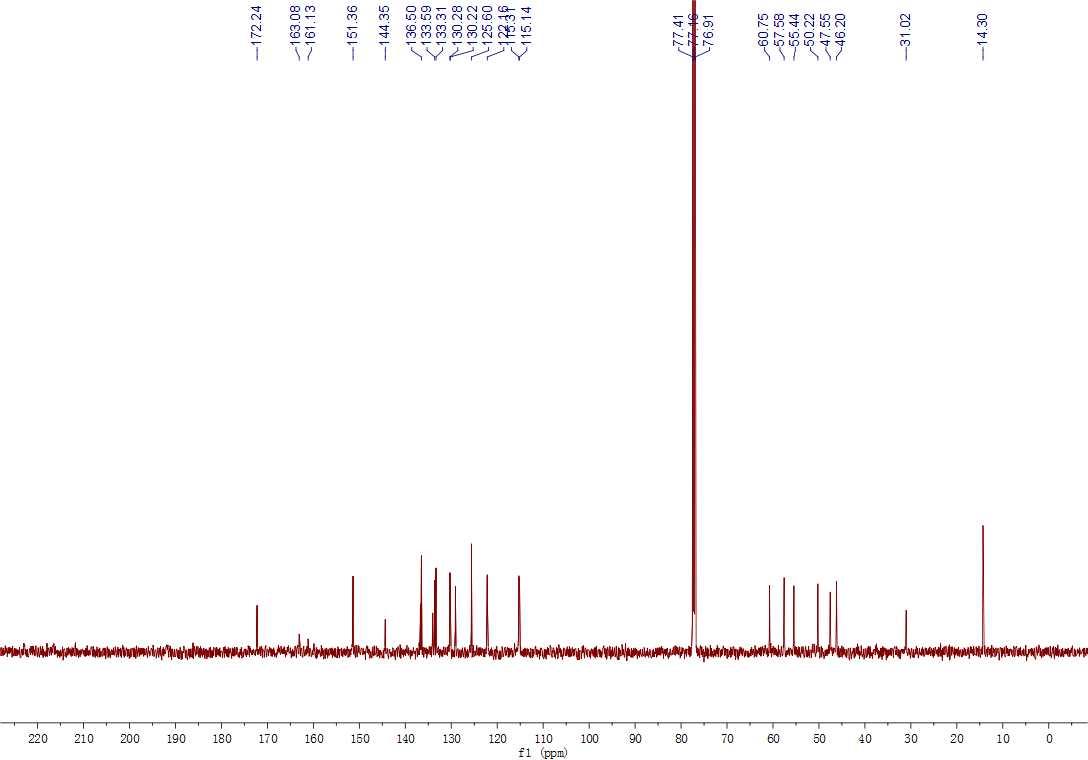


**Supplementary Figure 64.** ^13^C NMR spectrum of compound **45**.


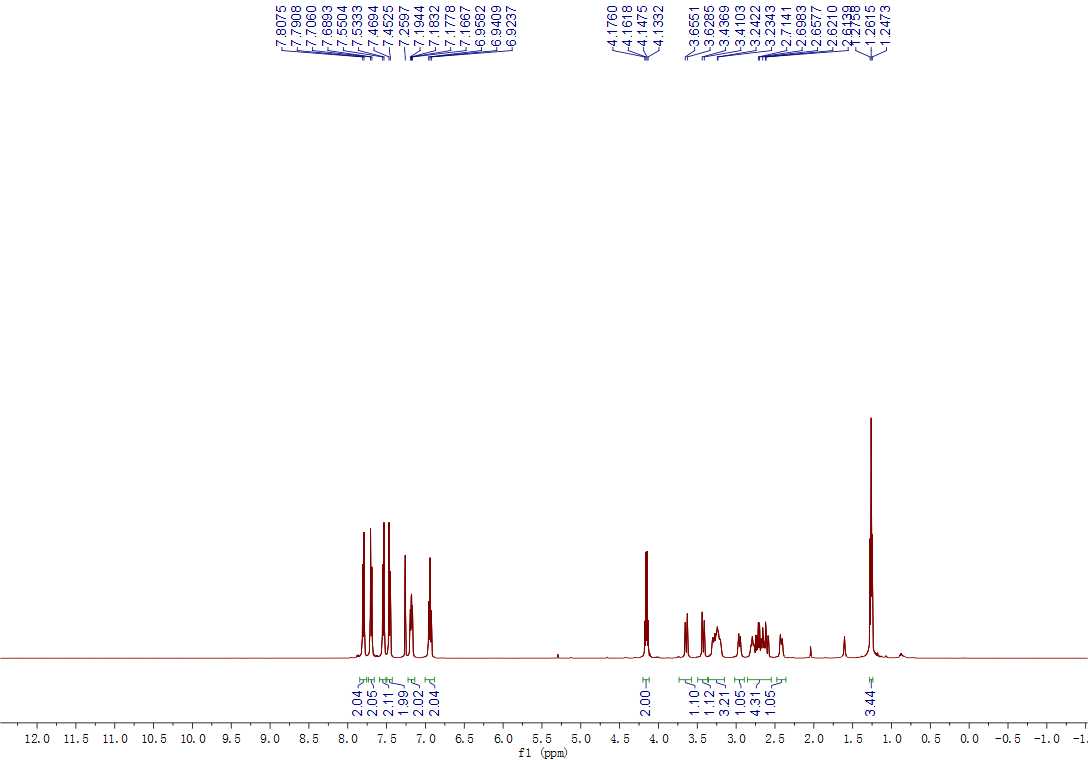


**Supplementary Figure 65.** ^1^H NMR spectrum of compound **46**.


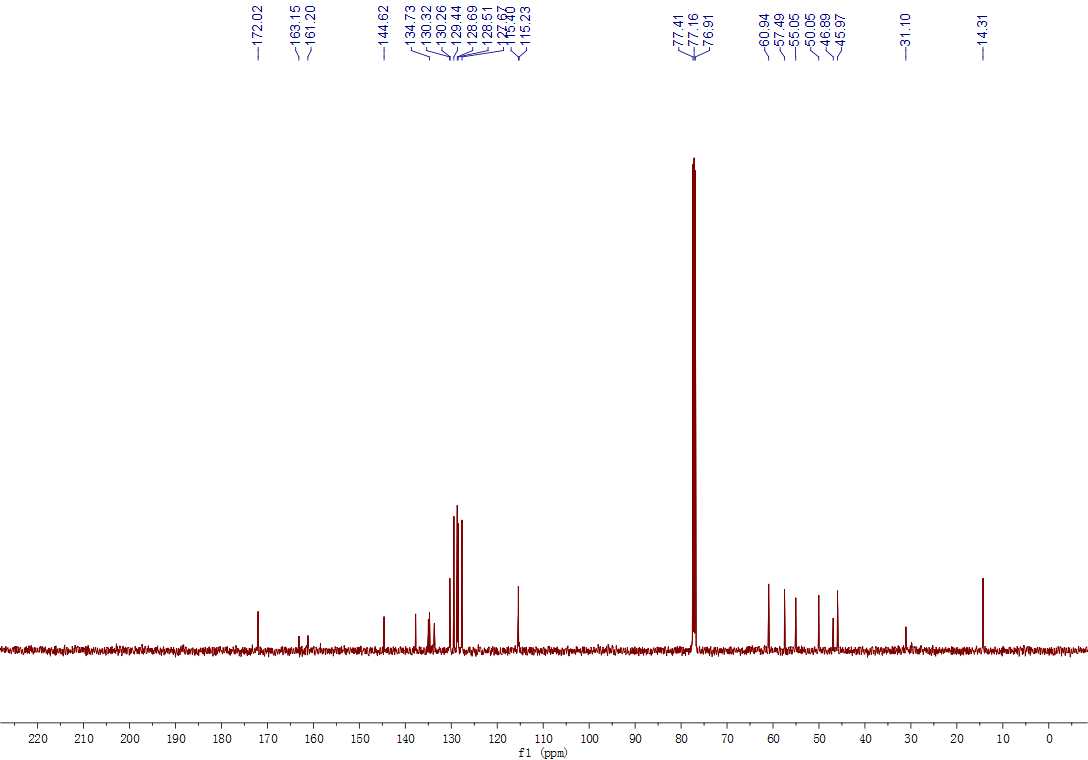


**Supplementary Figure 66.** ^13^C NMR spectrum of compound **46**.


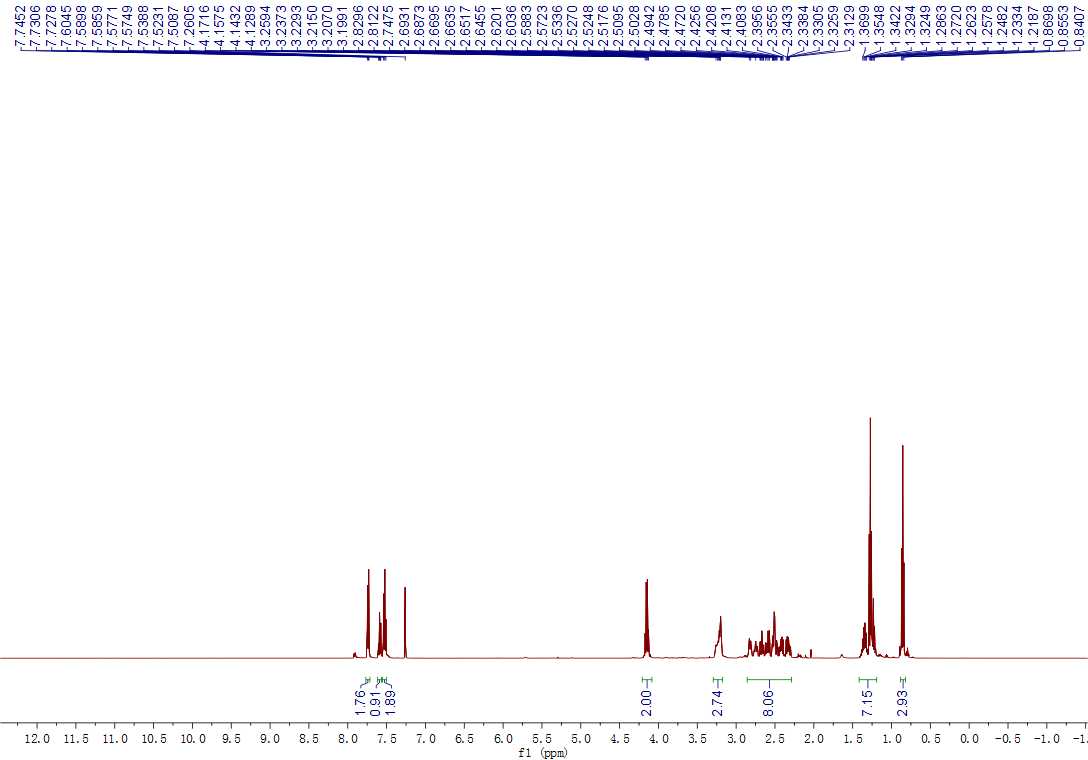


**Supplementary Figure 67.** ^1^H NMR spectrum of compound **47**.


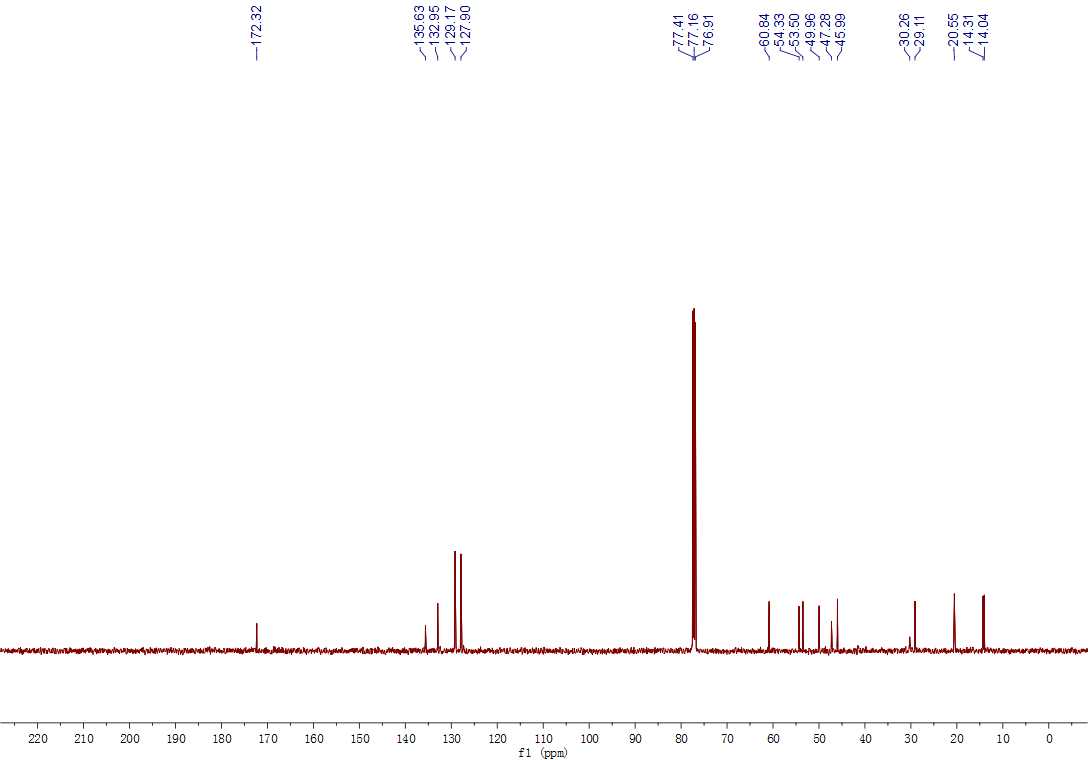


**Supplementary Figure 68.** ^13^C NMR spectrum of compound **47**.


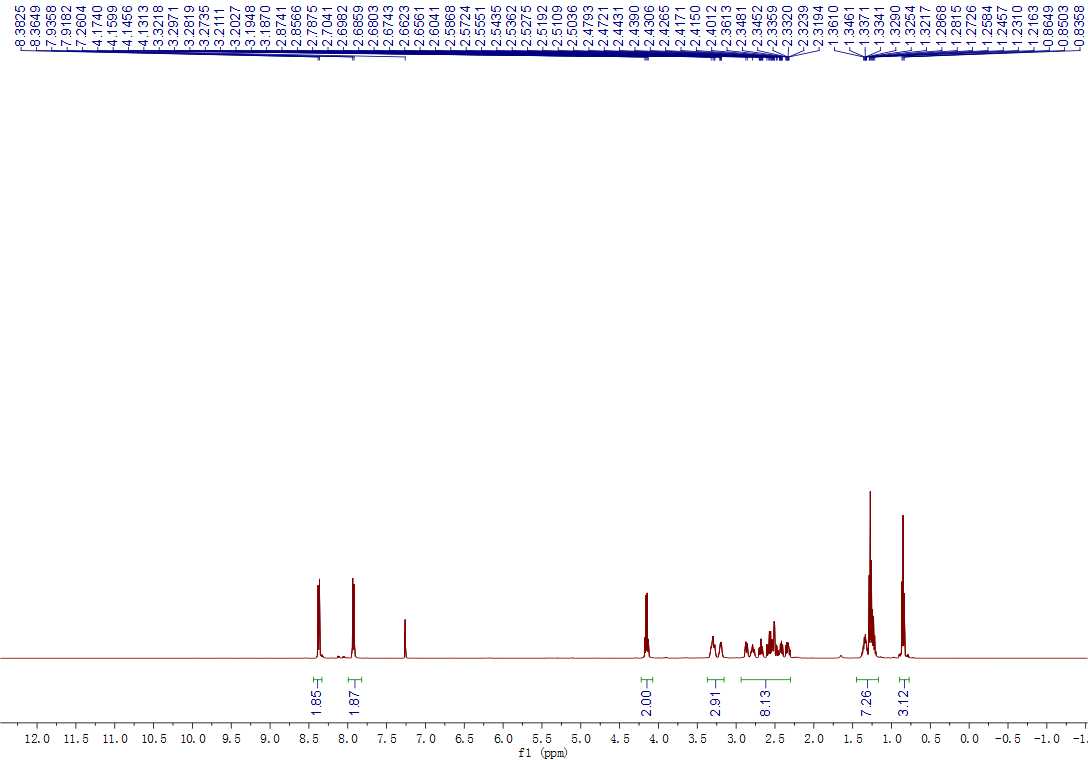


**Supplementary Figure 69.** ^1^H NMR spectrum of compound **48**.


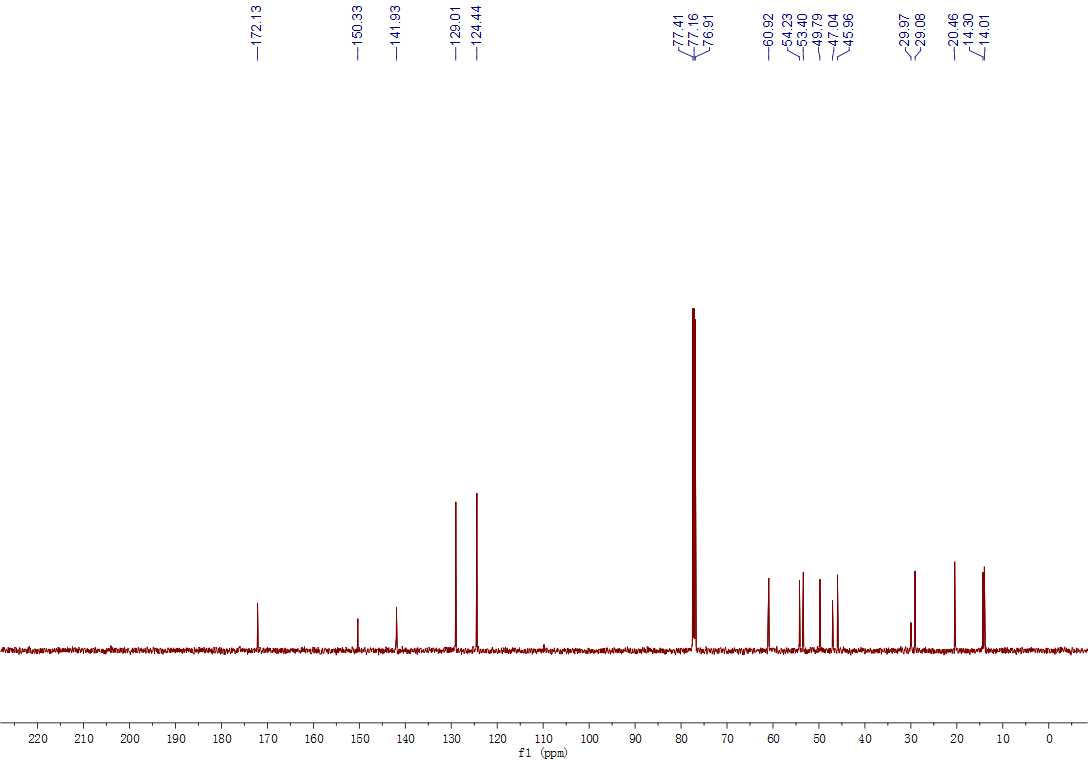


**Supplementary Figure 70.** ^13^C NMR spectrum of compound **48**.


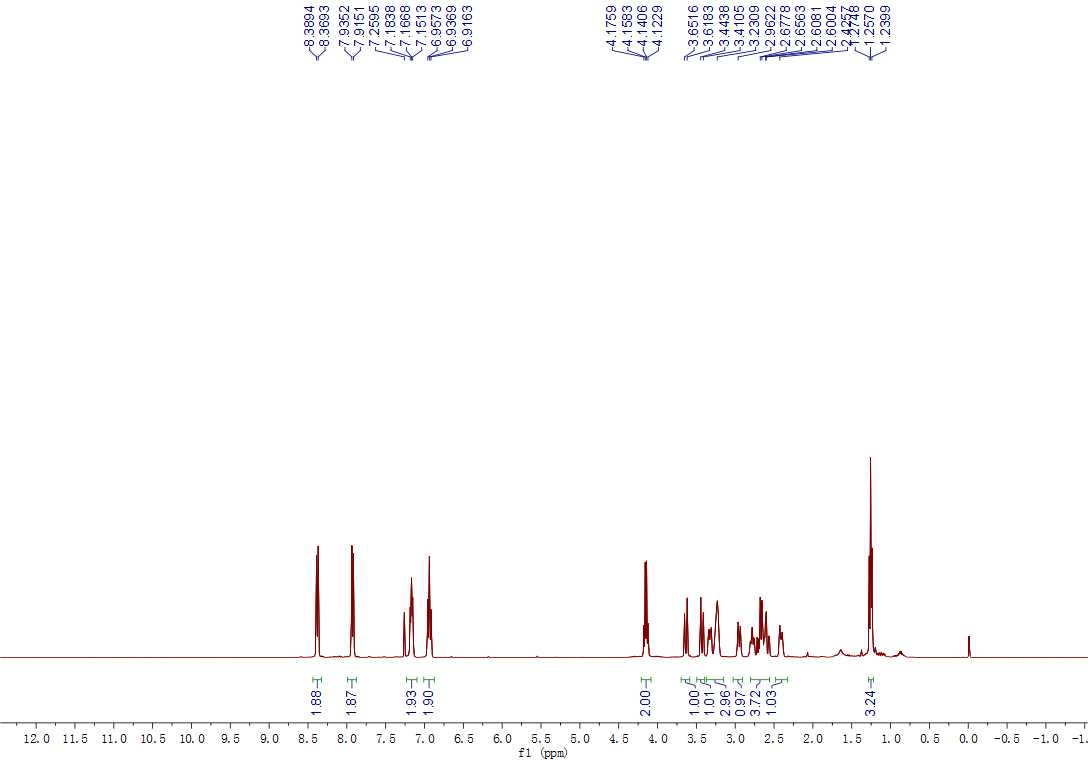


**Supplementary Figure 71.** ^1^H NMR spectrum of compound **49**.


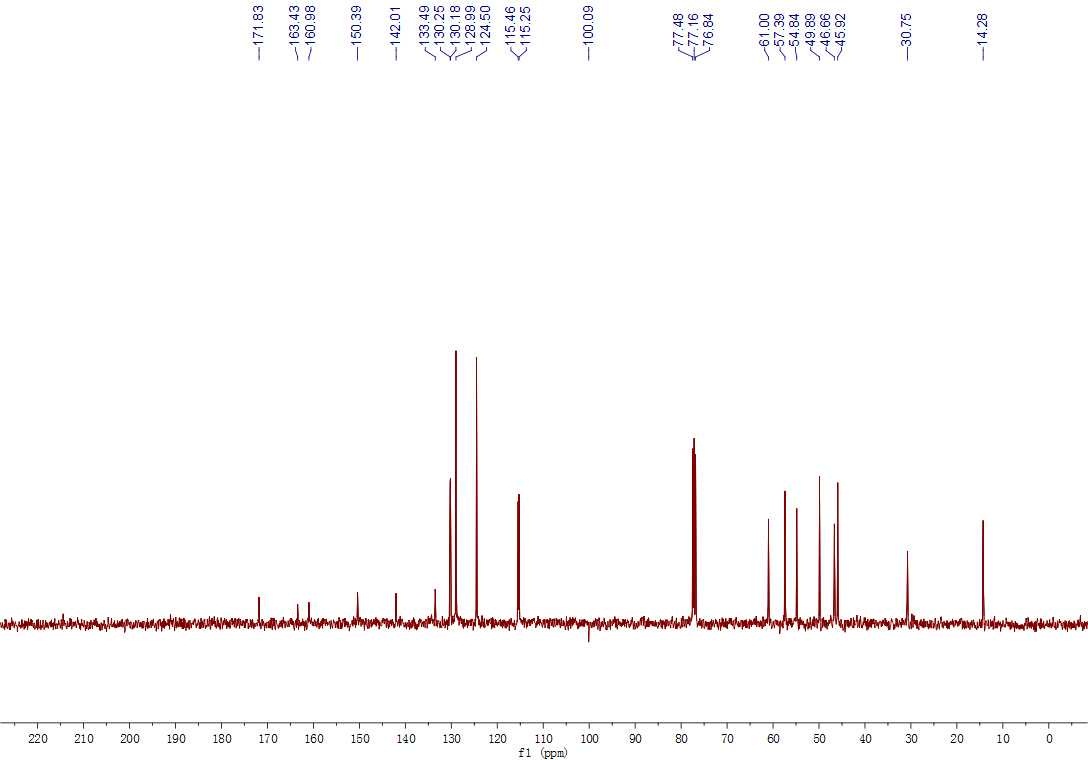


**Supplementary Figure 72.** ^13^C NMR spectrum of compound **49**.


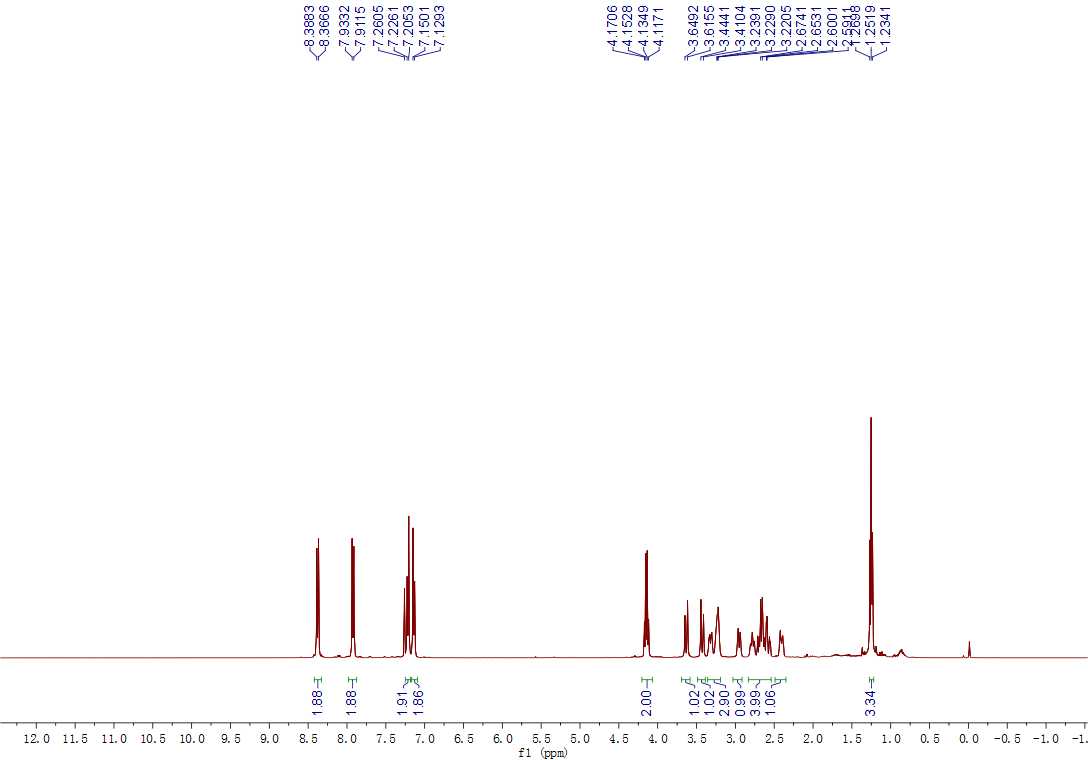


**Supplementary Figure 73.** ^1^H NMR spectrum of compound **50**.


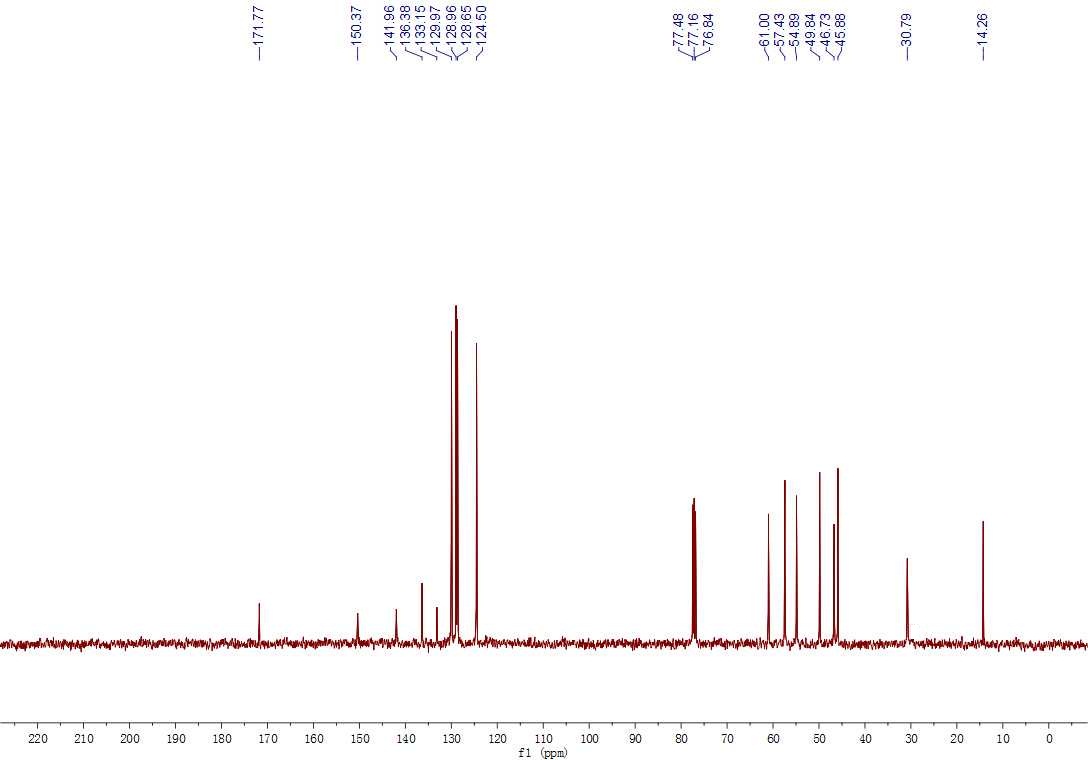


**Supplementary Figure 74.** ^13^C NMR spectrum of compound **50**.


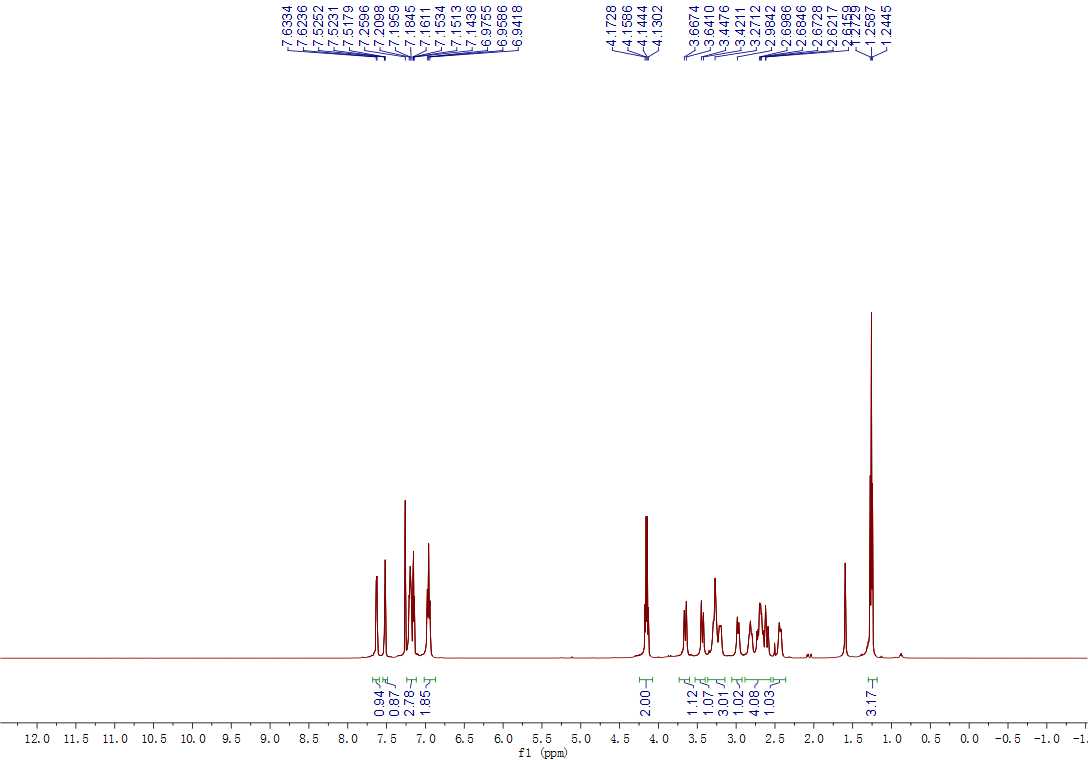


**Supplementary Figure 75.** ^1^H NMR spectrum of compound **51**.


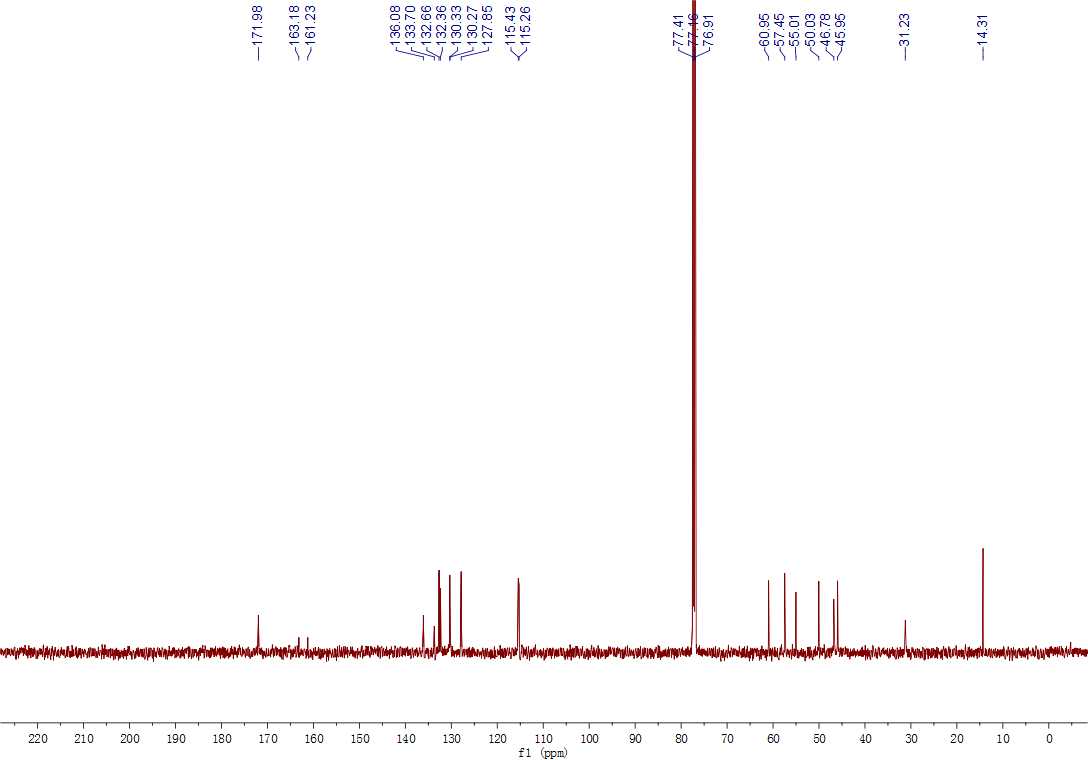


**Supplementary Figure 76.** ^13^C NMR spectrum of compound **51**.


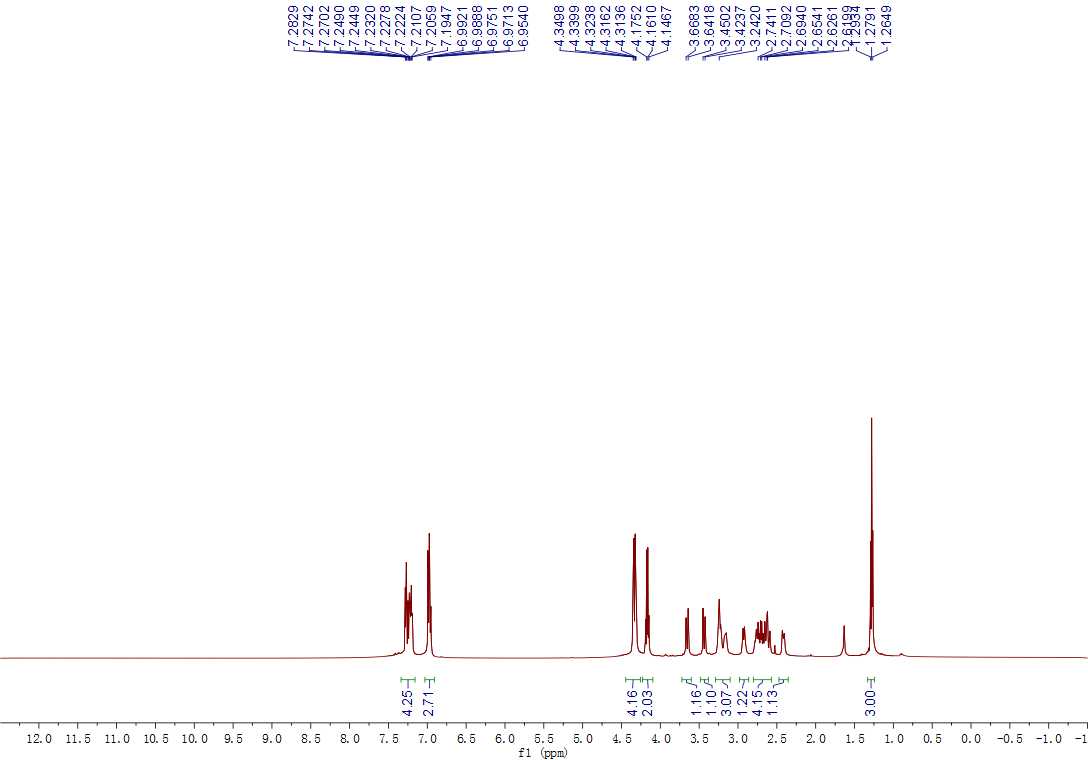


**Supplementary Figure 77.** ^1^H NMR spectrum of compound **52**.


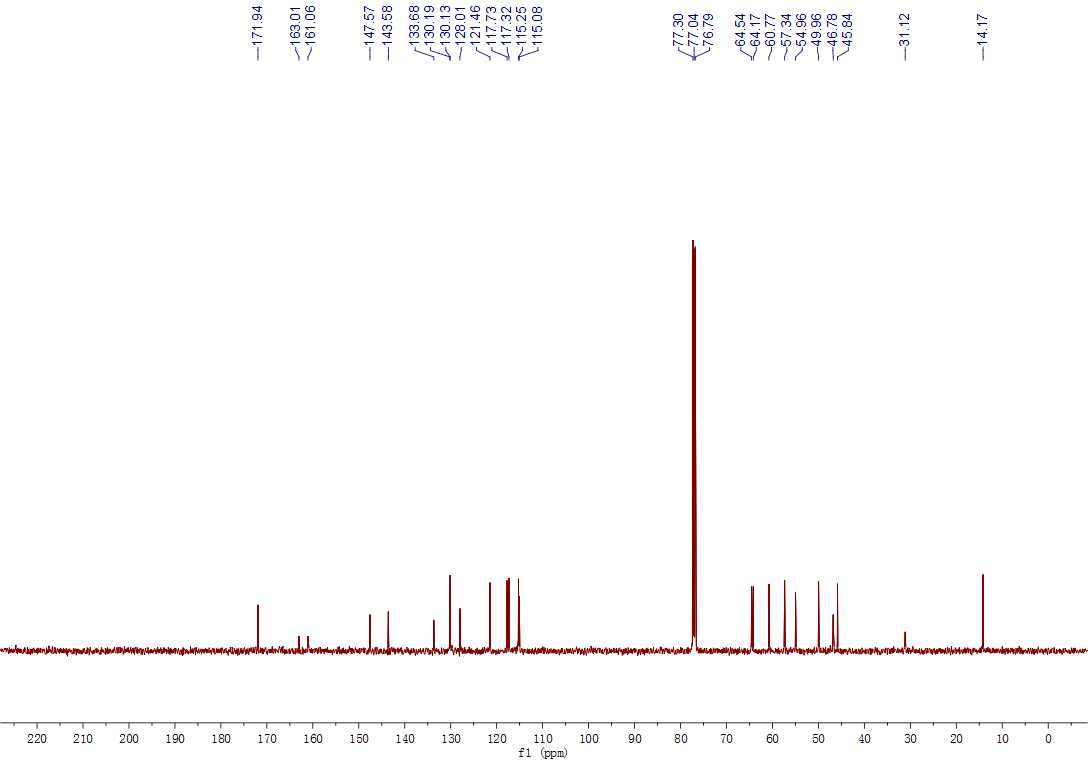


**Supplementary Figure 78.** ^13^C NMR spectrum of compound **52**.


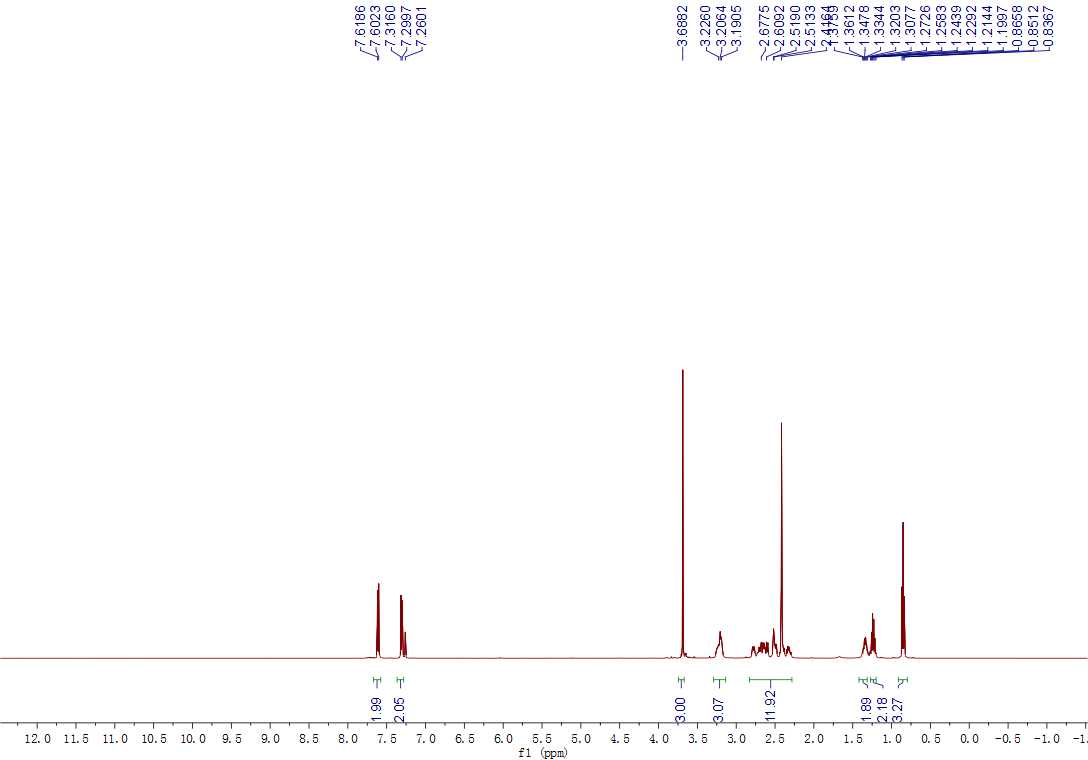


**Supplementary Figure 79.** ^1^H NMR spectrum of compound **53**.


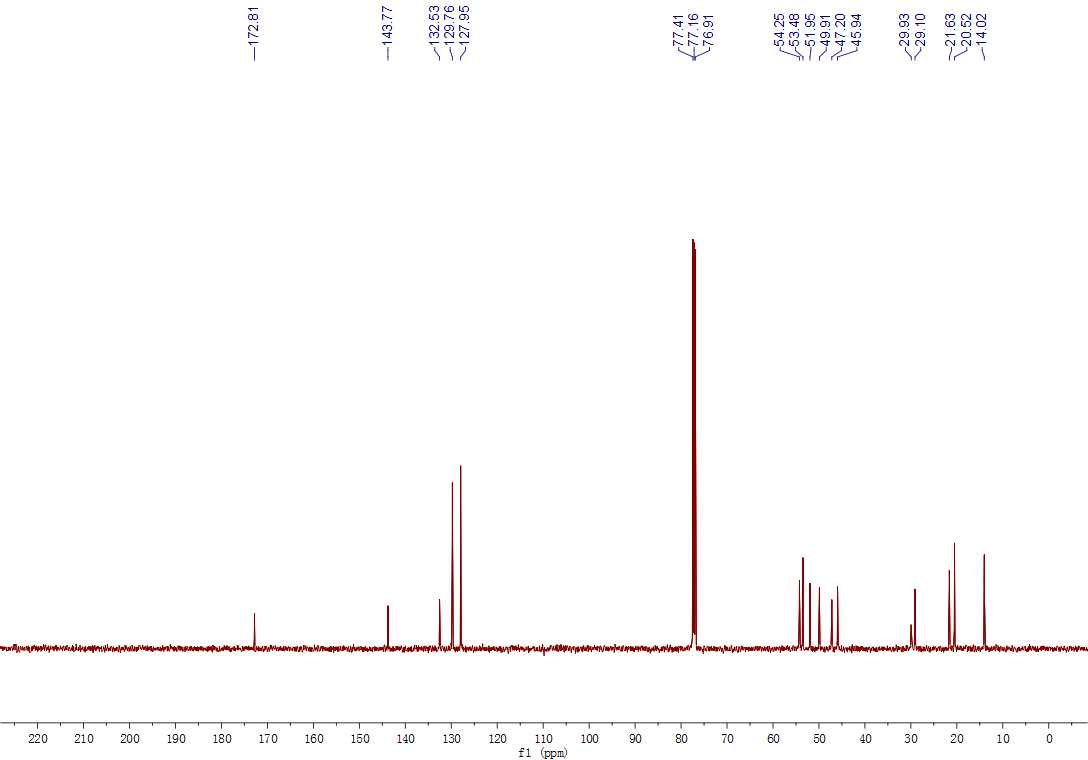


**Supplementary Figure 80.** ^13^C NMR spectrum of compound **53**.


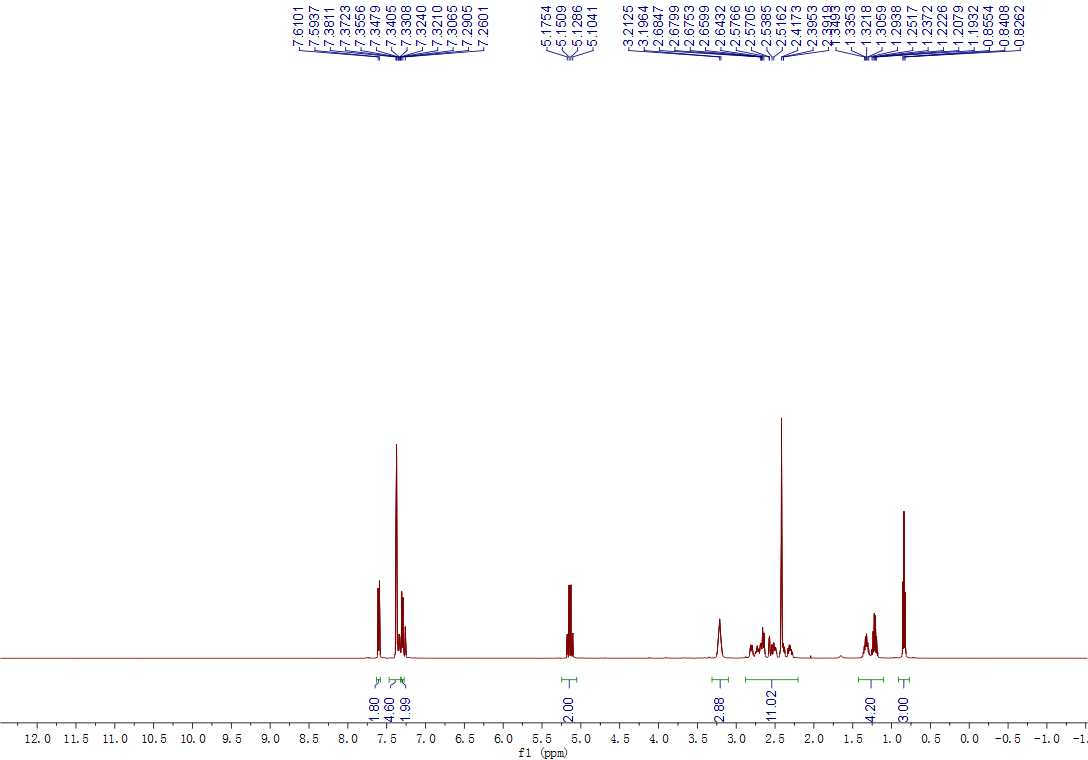


**Supplementary Figure 81.** ^1^H NMR spectrum of compound **54**.


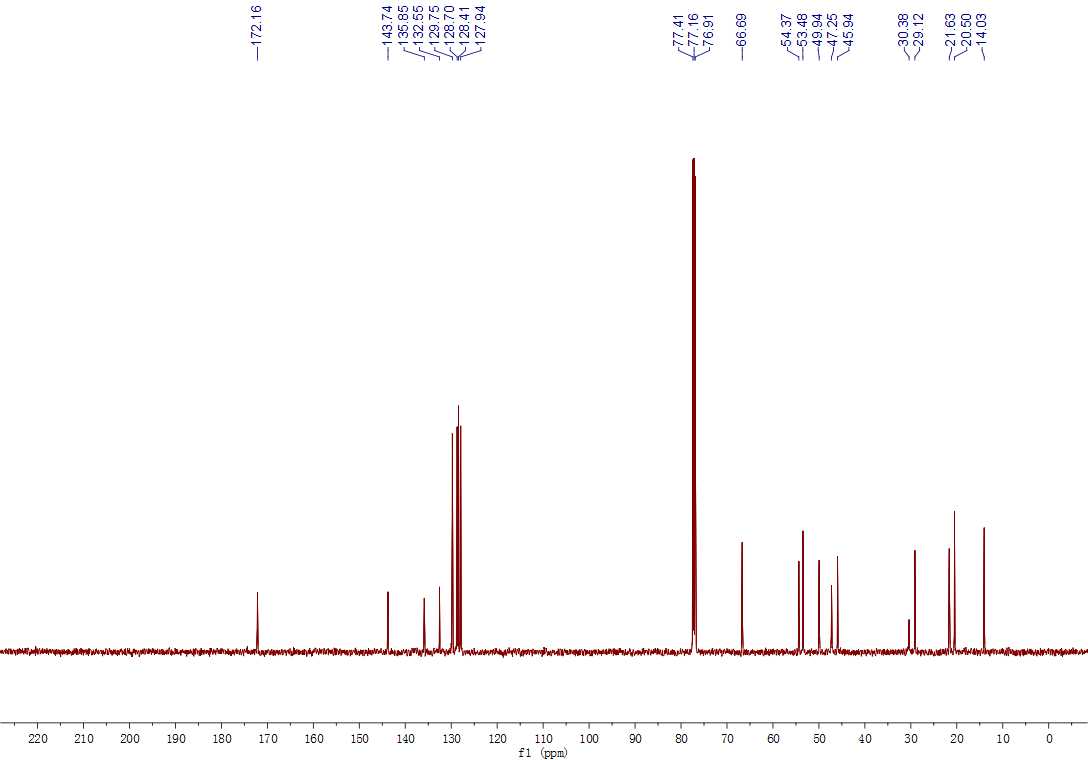


**Supplementary Figure 82.** ^13^C NMR spectrum of compound **54**.


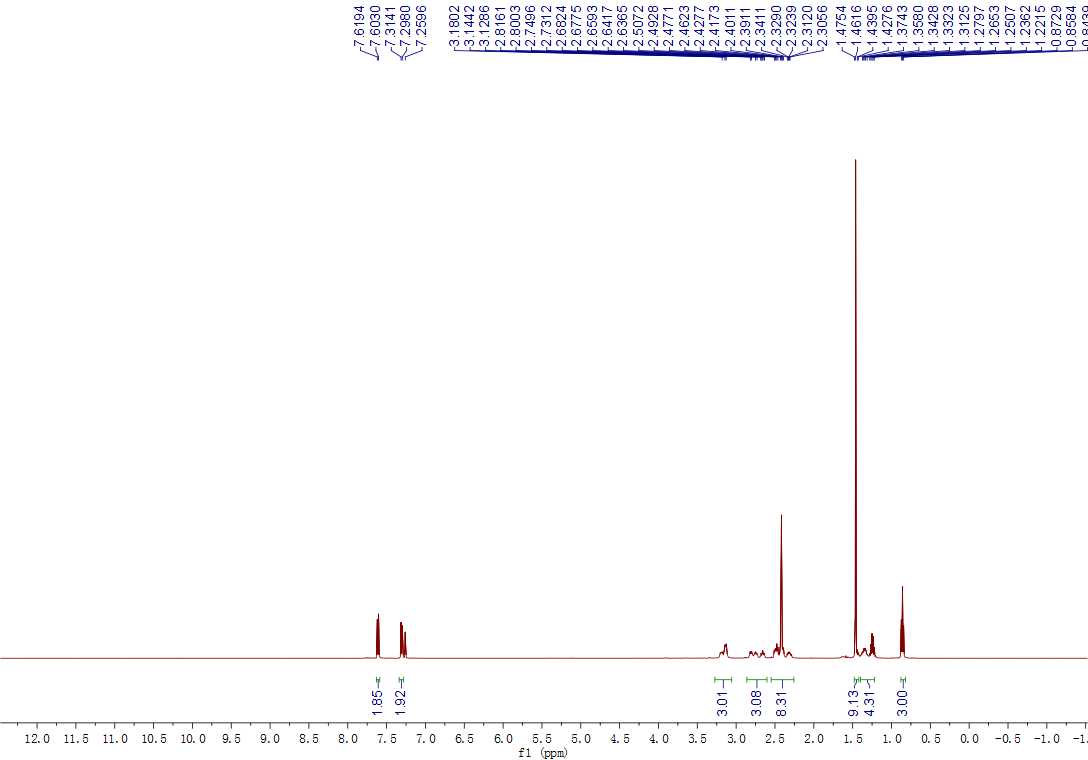


**Supplementary Figure 83.** ^1^H NMR spectrum of compound **55**.


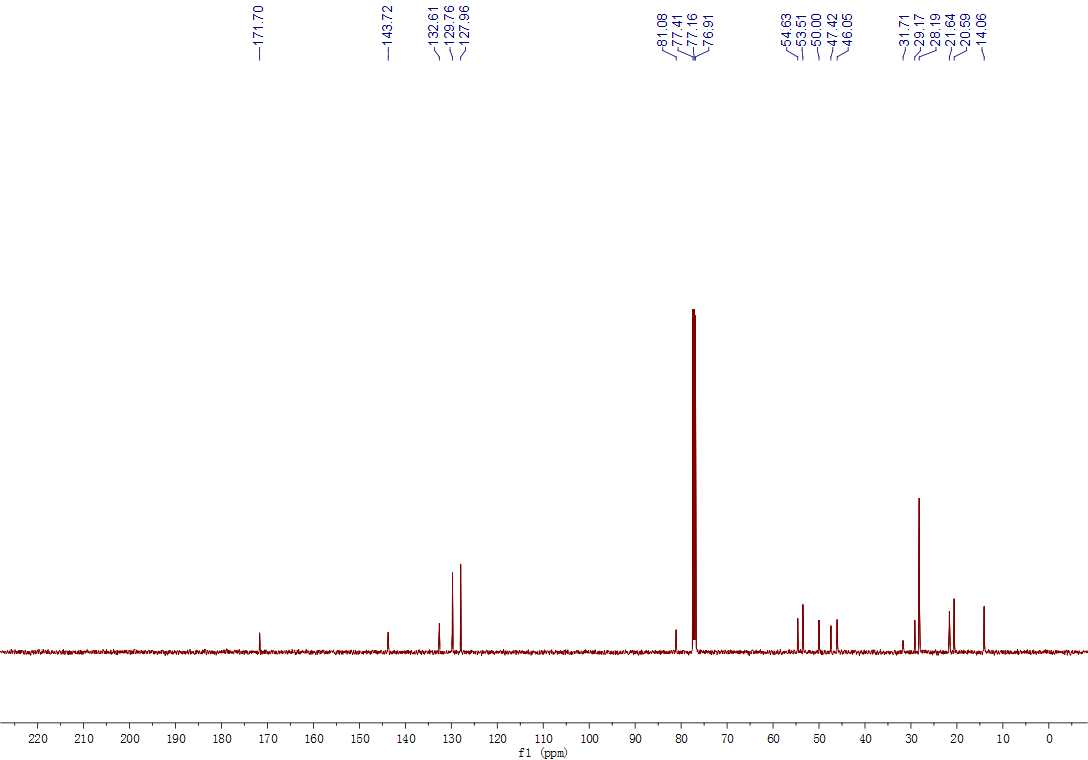


**Supplementary Figure 84.** ^13^C NMR spectrum of compound **55**.


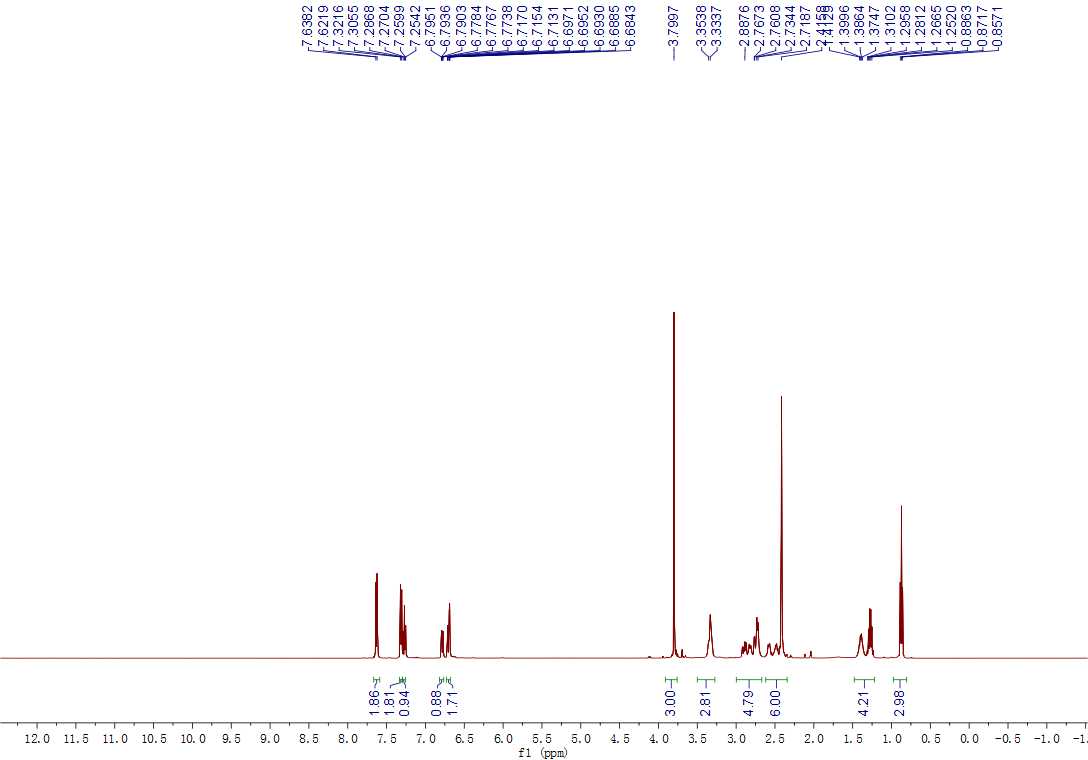


**Supplementary Figure 85.** ^1^H NMR spectrum of compound **56**.


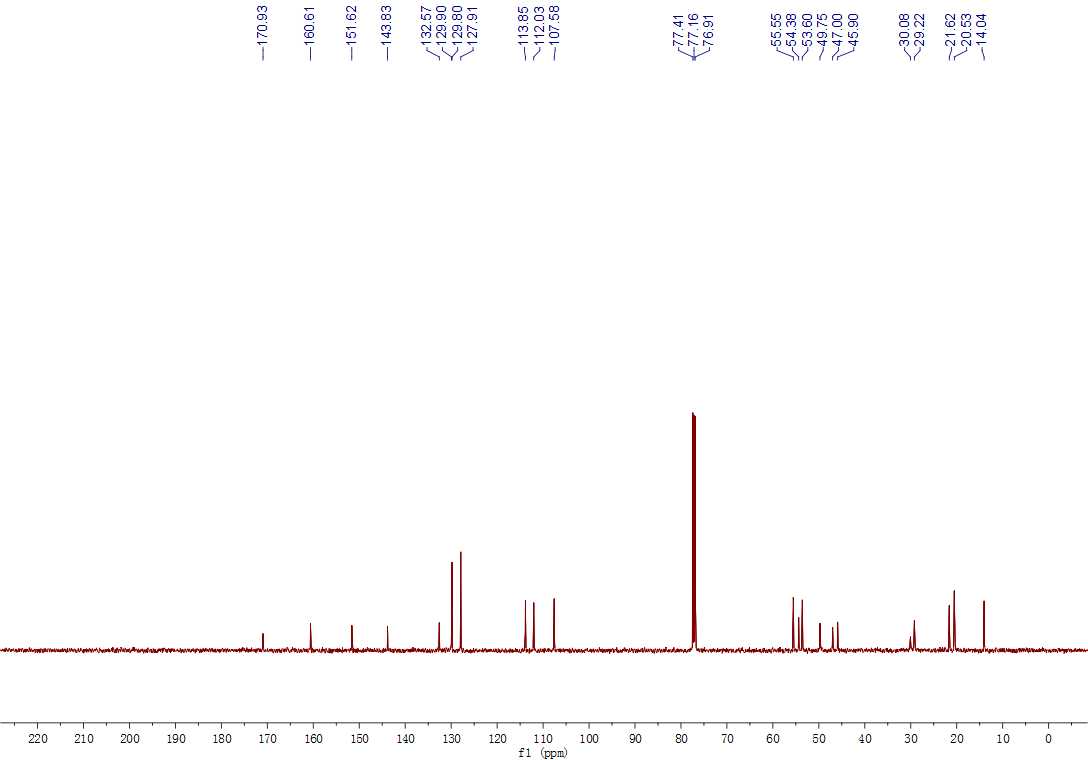


**Supplementary Figure 86.** ^13^C NMR spectrum of compound **56**.


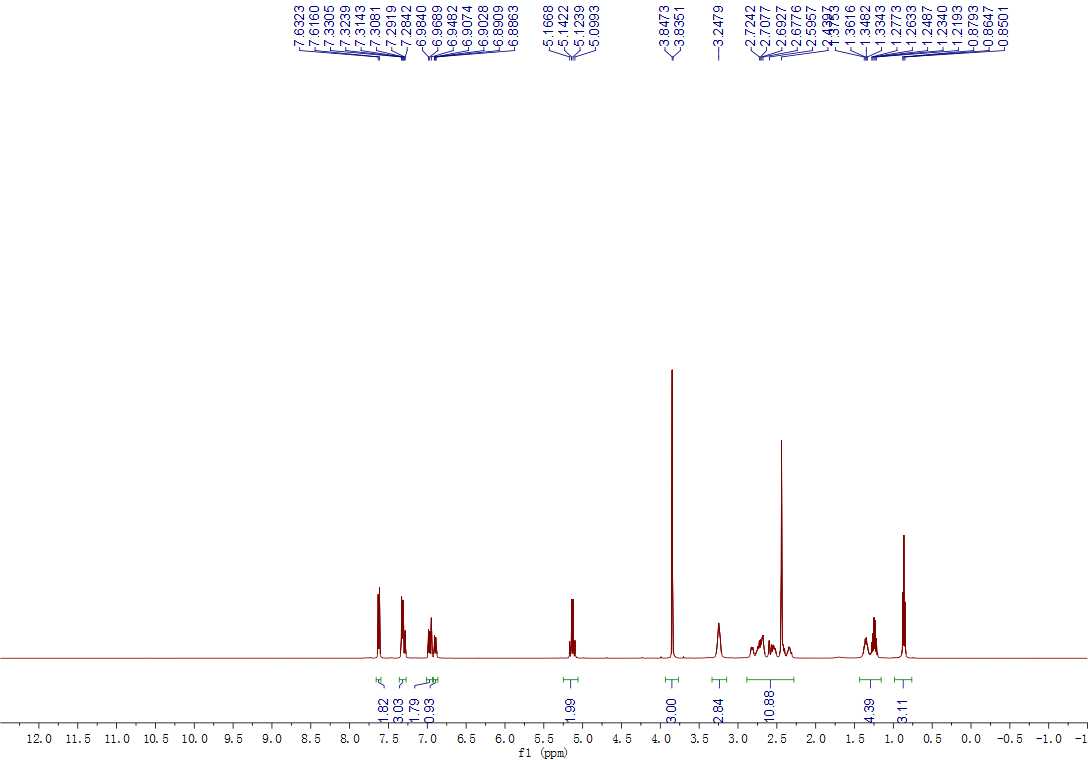


**Supplementary Figure 87.** ^1^H NMR spectrum of compound **57**.


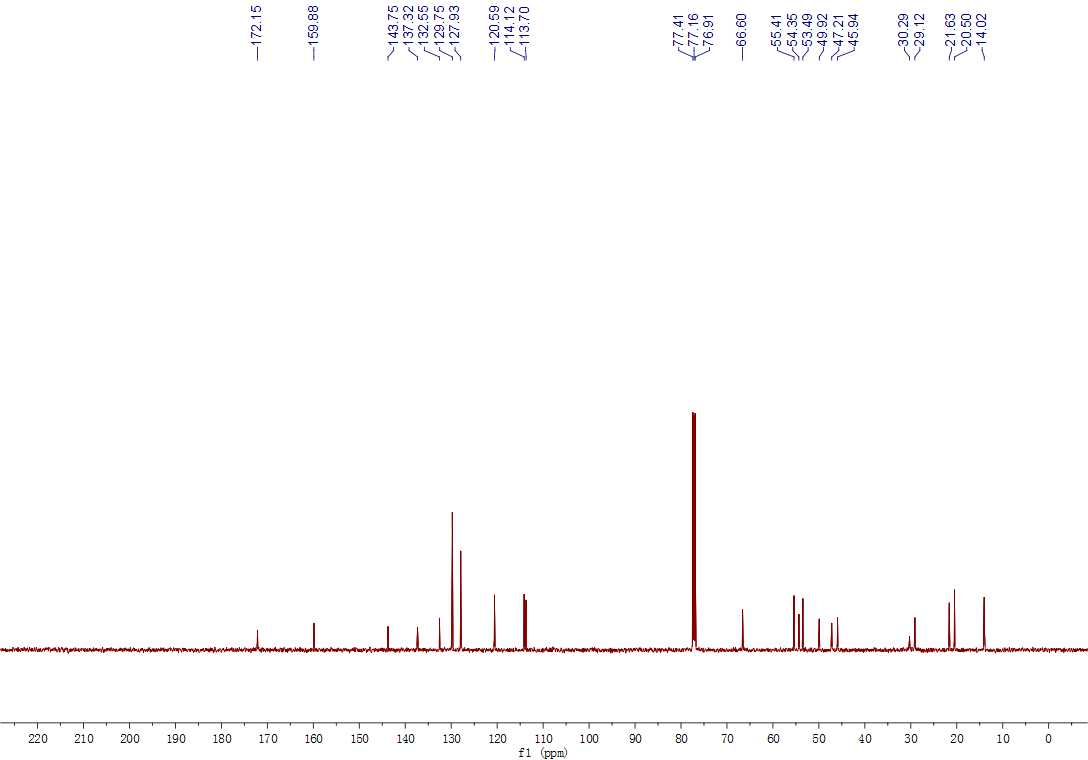


**Supplementary Figure 88.** ^13^C NMR spectrum of compound **57**.


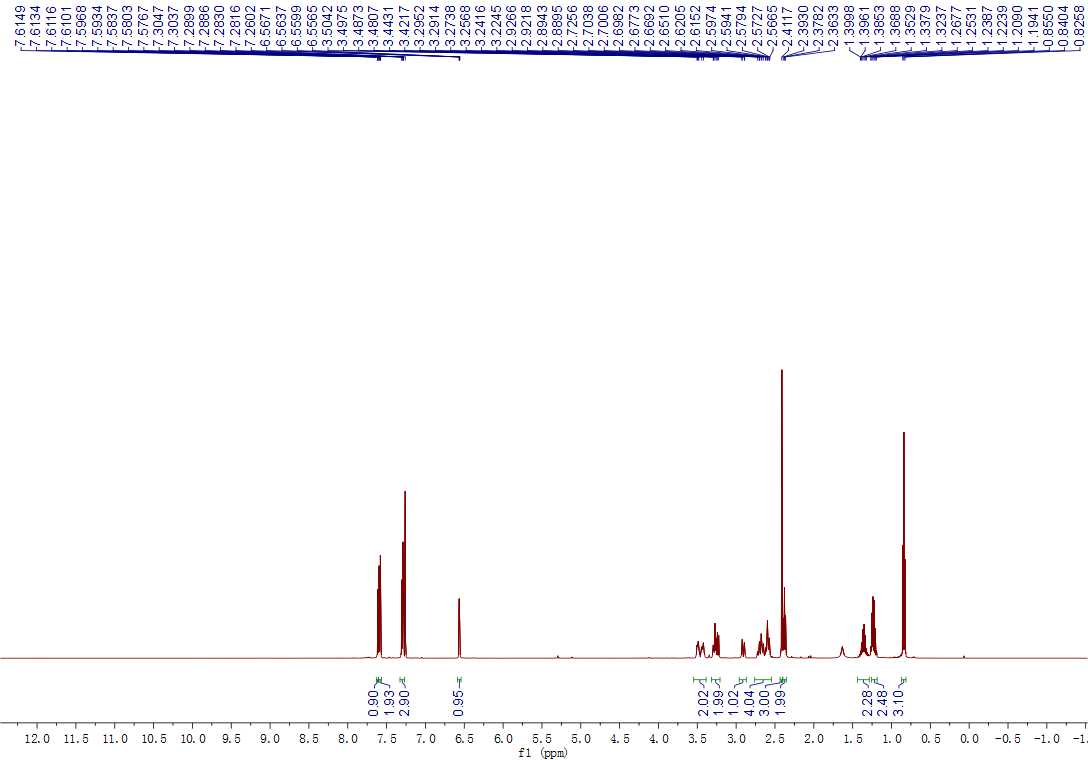


**Supplementary Figure 89.** ^1^H NMR spectrum of compound **58**.


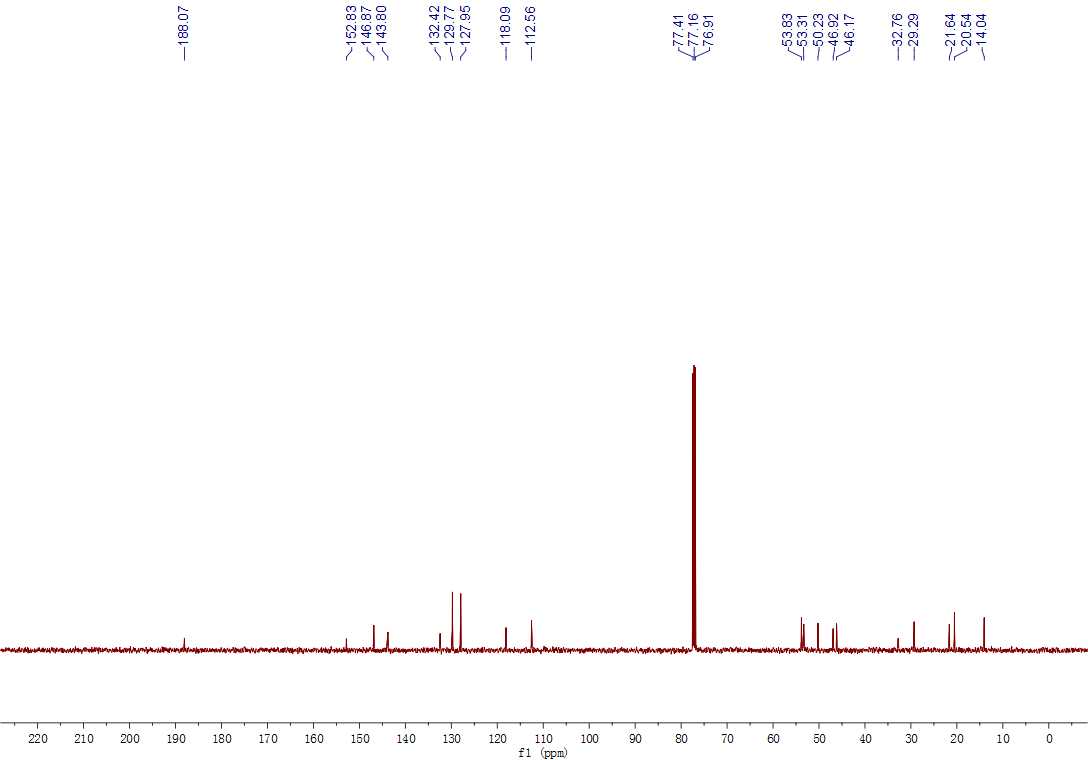


**Supplementary Figure 90.** ^13^C NMR spectrum of compound **58**.


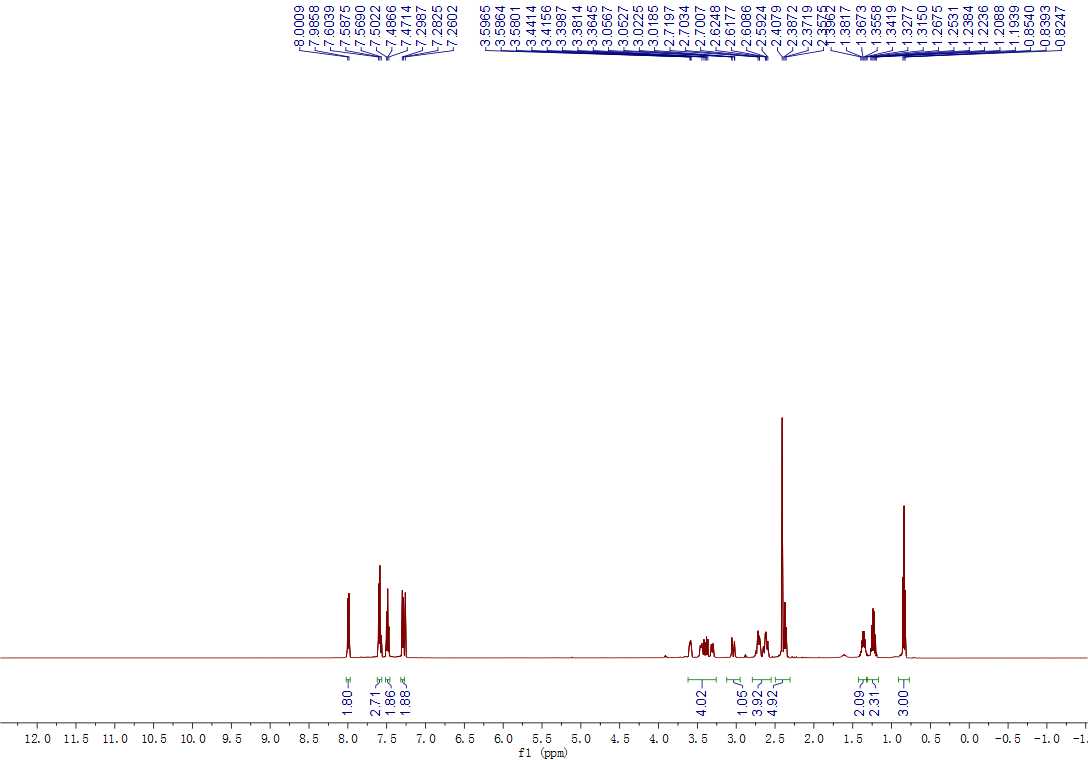


**Supplementary Figure 91.** ^1^H NMR spectrum of compound **59**.


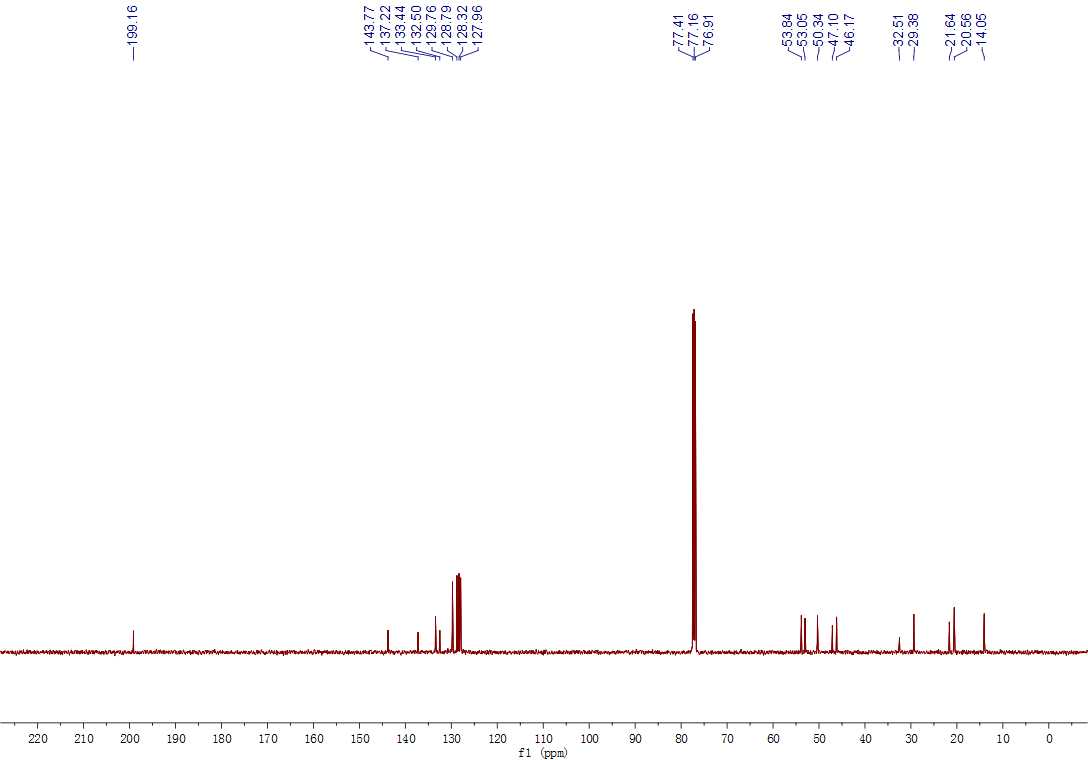


**Supplementary Figure 92.** ^13^C NMR spectrum of compound **59**.

**Supplementary Figure 93.** ^1^H NMR spectrum of compound **60**.

**Supplementary Figure 94.** ^13^C NMR spectrum of compound **60**.

**Supplementary Figure 95.** ^1^H NMR spectrum of compound **61**.

**Supplementary Figure 96.** ^13^C NMR spectrum of compound **61**.

**Supplementary Figure 97.** ^1^H NMR spectrum of compound **62**.

**Supplementary Figure 98.** ^13^C NMR spectrum of compound **62**.

**Supplementary Figure 99.** ^1^H NMR spectrum of compound **63**.

**Supplementary Figure 100.** ^13^C NMR spectrum of compound **63**.

**Supplementary Figure 101.** ^1^H NMR spectrum of compound **64**.

**Supplementary Figure 102.** ^13^C NMR spectrum of compound **64**.

**Supplementary Figure 103.** ^1^H NMR spectrum of compound **65**.

**Supplementary Figure 104.** ^13^C NMR spectrum of compound **65**.

**Supplementary Figure 105.** ^1^H NMR spectrum of compound **66**.

**Supplementary Figure 106.** ^13^C NMR spectrum of compound **66**.

**Supplementary Figure 107.** ^1^H NMR spectrum of compound **67**.

**Supplementary Figure 108.** ^13^C NMR spectrum of compound **67**.

**Supplementary Figure 109.** ^1^H NMR spectrum of compound **68**.

**Supplementary Figure 110.** ^13^C NMR spectrum of compound **68**.

**Supplementary Figure 111.** ^1^H NMR spectrum of compound **69**.

**Supplementary Figure 112.** ^13^C NMR spectrum of compound **69**.

**Supplementary Figure 113.** ^1^H NMR spectrum of compound **70**.

**Supplementary Figure 114.** ^13^C NMR spectrum of compound **70**.

**Supplementary Figure 115.** ^1^H NMR spectrum of compound **71**.

**Supplementary Figure 116.** ^13^C NMR spectrum of compound **71**.

**Supplementary Figure 117.** ^1^H NMR spectrum of compound **72**.

**Supplementary Figure 118.** ^13^C NMR spectrum of compound **72**.

**Supplementary Figure 119.** ^1^H NMR spectrum of compound **73**.

**Supplementary Figure 120.** ^13^C NMR spectrum of compound **73**.

**Supplementary Figure 121.** ^1^H NMR spectrum of compound **74**.

**Supplementary Figure 122.** ^13^C NMR spectrum of compound **74**.

**Supplementary Figure 123.** ^1^H NMR spectrum of compound **75**.

**Supplementary Figure 124.** ^13^C NMR spectrum of compound **75**.

**Supplementary Figure 125.** ^1^H NMR spectrum of compound **76**.

**Supplementary Figure 126.** ^13^C NMR spectrum of compound **76**.

**Supplementary Figure 127.** ^1^H NMR spectrum of compound **77**.

**Supplementary Figure 128.** ^13^C NMR spectrum of compound **77**.

**Supplementary Figure 129.** ^1^H NMR spectrum of compound **78**.

**Supplementary Figure 130.** ^13^C NMR spectrum of compound **78**.

**Supplementary Figure 131.** ^1^H NMR spectrum of compound **79**.

**Supplementary Figure 132.** ^13^C NMR spectrum of compound **79**.

**Supplementary Figure 133.** ^1^H NMR spectrum of compound **80**.

**Supplementary Figure 134.** ^13^C NMR spectrum of compound **80**.

**Supplementary Figure 135.** ^1^H NMR spectrum of compound **81**.

**Supplementary Figure 136.** ^13^C NMR spectrum of compound **81**.

**Supplementary Figure 137.** ^1^H NMR spectrum of compound **86**.

**Supplementary Figure 138.** ^13^C NMR spectrum of compound **86**.

**Supplementary Figure 139.** ^1^H NMR spectrum of compound **87**.

**Supplementary Figure 140.** ^13^C NMR spectrum of compound **87**.

**Supplementary Figure 141.** ^1^H NMR spectrum of compound **88**.

**Supplementary Figure 142.** ^13^C NMR spectrum of compound **88**.

**Supplementary Figure 143.** ^1^H NMR spectrum of compound **89**.

**Supplementary Figure 144.** ^13^C NMR spectrum of compound **89**.

**Supplementary Figure 145.** ^1^H NMR spectrum of compound **90**.

**Supplementary Figure 146.** ^13^C NMR spectrum of compound **90**.

**Supplementary Figure 147.** ^1^H NMR spectrum of compound **91**.

**Supplementary Figure 148.** ^13^C NMR spectrum of compound **91**.

**Supplementary Figure 149.** ^1^H NMR spectrum of compound **92**.

**Supplementary Figure 150.** ^13^C NMR spectrum of compound **92**.

**Supplementary Figure 151.** ^1^H NMR spectrum of compound **93**.

**Supplementary Figure 152.** ^13^C NMR spectrum of compound **93**.

**Supplementary Figure 153.** ^1^H NMR spectrum of compound **94**.

**Supplementary Figure 154.** ^13^C NMR spectrum of compound **94**.

**Supplementary Figure 155.** ^1^H NMR spectrum of compound **95**.

**Supplementary Figure 156.** ^13^C NMR spectrum of compound **95**.

**Supplementary Figure 157.** ^1^H NMR spectrum of compound **96**.

**Supplementary Figure 158.** ^13^C NMR spectrum of compound **96**.

**Supplementary Figure 159.** ^1^H NMR spectrum of compound **97**.

**Supplementary Figure 160.** ^13^C NMR spectrum of compound **97**.

**Supplementary Figure 161.** ^1^H NMR spectrum of compound **98**.

**Supplementary Figure 162.** ^13^C NMR spectrum of compound **98**.

**Supplementary Figure 163.** ^1^H NMR spectrum of compound **99**.

**Supplementary Figure 164.** ^13^C NMR spectrum of compound **99**.

**Supplementary Figure 165.** ^1^H NMR spectrum of compound **100**.

**Supplementary Figure 166.** ^13^C NMR spectrum of compound **100**.

**Supplementary Figure 167.** ^1^H NMR spectrum of compound **101**.

**Supplementary Figure 168.** ^13^C NMR spectrum of compound **101**.

**Supplementary Figure 169.** ^1^H NMR spectrum of compound **102**.

**Supplementary Figure 170.** ^13^C NMR spectrum of compound **102**.

**Supplementary Figure 171.** ^1^H NMR spectrum of compound **103**.

**Supplementary Figure 172.** ^13^C NMR spectrum of compound **103**.

**Supplementary Figure 173.** ^1^H NMR spectrum of compound **104**.

**Supplementary Figure 174.** ^13^C NMR spectrum of compound **104**.

**Supplementary Figure 175.** ^1^H NMR spectrum of compound **105**.

**Supplementary Figure 176.** ^13^C NMR spectrum of compound **105**.

**Supplementary Figure 177.** ^1^H NMR spectrum of compound **106**.

**Supplementary Figure 178.** ^13^C NMR spectrum of compound **106**.

**Supplementary Figure 179.** ^1^H NMR spectrum of compound **107**.

**Supplementary Figure 180.** ^13^C NMR spectrum of compound **107**.

**Supplementary Figure 181.** ^1^H NMR spectrum of compound **108**.

**Supplementary Figure 182.** ^13^C NMR spectrum of compound **108**.

**Supplementary Figure 183.** ^1^H NMR spectrum of compound **109**.

**Supplementary Figure 184.** ^13^C NMR spectrum of compound **109**.

**Supplementary Figure 185.** ^1^H NMR spectrum of compound **110**.

**Supplementary Figure 186.** ^13^C NMR spectrum of compound **110**.

**Supplementary Figure 187.** ^1^H NMR spectrum of compound **111**.

**Supplementary Figure 188.** ^13^C NMR spectrum of compound **111**.

**Supplementary Figure 189.** ^1^H NMR spectrum of compound **112**.

**Supplementary Figure 190.** ^13^C NMR spectrum of compound **112**.

**Supplementary Figure 191.** ^1^H NMR spectrum of compound **113**.

**Supplementary Figure 192.** ^13^C NMR spectrum of compound **113**.

**Supplementary Figure 193.** ^1^H NMR spectrum of compound **114**.

**Supplementary Figure 194.** ^13^C NMR spectrum of compound **114**.

**Supplementary Figure 195.** ^1^H NMR spectrum of compound **115**.

**Supplementary Figure 196.** ^13^C NMR spectrum of compound **115**.

**Supplementary Figure 197.** ^1^H NMR spectrum of compound **116**.

**Supplementary Figure 198.** ^13^C NMR spectrum of compound **116**.

**Supplementary Figure 199.** ^1^H NMR spectrum of compound **76b**.

**Supplementary Figure 200.** ^13^C NMR spectrum of compound **76b**.

**Supplementary Figure 201.** ^1^H NMR spectrum of compound **81b**.

**Supplementary Figure 202.** ^13^C NMR spectrum of compound **81b**.

**Supplementary Figure 203.** ^1^H NMR spectrum of compound **117**.

**Supplementary Figure 204.** ^13^C NMR spectrum of compound **117**.

**Supplementary Figure 205.** ^1^H NMR spectrum of compound **122**.

**Supplementary Figure 206.** ^13^C NMR spectrum of compound **122**.

**Supplementary Figure 207.** ^1^H NMR spectrum of compound **123**.

**Supplementary Figure 208.** ^13^C NMR spectrum of compound **123**.

**Supplementary Figure 209.** ^1^H NMR spectrum of compound **124**.

**Supplementary Figure 210.** ^13^C NMR spectrum of compound **124**.

**Supplementary Figure 211.** ^1^H NMR spectrum of compound **125**.

**Supplementary Figure 212.** ^13^C NMR spectrum of compound **125**.

**Supplementary Figure 213.** ^1^H NMR spectrum of compound **137**.

**Supplementary Figure 214.** ^13^C NMR spectrum of compound **137**.

**Supplementary Figure 215.** ^1^H NMR spectrum of compound **136**.

**Supplementary Figure 216.** ^13^C NMR spectrum of compound **136**.

**Supplementary Figure 217.** ^1^H NMR spectrum of compound **139**.

**Supplementary Figure 218.** ^13^C NMR spectrum of compound **139**.

**Supplementary Figure 219.** ^1^H NMR spectrum of compound **11a**.

**Supplementary Figure 220.** ^13^C NMR spectrum of compound **11a**.

**Supplementary Figure 221.** ^1^H NMR spectrum of compound **143**.

**Supplementary Figure 222.** ^13^C NMR spectrum of compound **143**.

**Supplementary Figure 223.** ^1^H NMR spectrum of compound **144**.

**Supplementary Figure 224.** ^13^C NMR spectrum of compound **144**.

**Supplementary References**

(1) Squires, C., Baxter, C. W., Campbell, J., Lindoy, L. F., McNab, H., Parkin, A., Parsons, S., Tasker, P. A., Wei, G. & White, D. J. Design of base metal extractants. Part 1. Inter-ligand hydrogen bonding in the assembly of pseudo-macrocyclic bis(aminosulfonamidato)M(II) complexes. *Dalton Trans.* 2026-2034 (2006)*.*

(2) Fu, L. & Davies H. M. L. Scope of the reactions of indolyl- and pyrrolyl-tethered *N*-sulfonyl-1,2,3-triazoles: rhodium(II)-catalyzed synthesis of indole- and pyrrole-fused polycyclic compounds *Org. Lett.*, **19**, 1504-1507 (2017).

(3) Gritsenko, R. T., Levin, V. V., Dilman, A. D., Belyakov, P. A., Struchkova, M. I. & Tartakovsky, V. A. Trifluoromethylation of enamines under acidic conditions. *Tetrahedron Lett.* 50, 2994-2997 (2009).

(4) Seo, S.-Y. & Kim, G. A synthetic study of magallanesine by cyclization of a benzamidoacrylate intermediate. *Tetrahedron Lett.* **56**, 3835 (2015).

(5) Yan, Z., Wu, B., Gao, X., Chen, M.-W. & Zhou. Y.-G. Enantioselective Synthesis of α-Amino Phosphonates via Pd-Catalyzed Asymmetric Hydrogenation. *Org. Lett.* **18**, 692-695 (2016).

(6) Moria, Y., Ogawab, Y., Mochizukic, A., Nakamurac, Y., Fujimotoc, T., Sugitac, C., Miyazakic, S., Tamakic, K., Nagayamad, T., Nagaie, Y. & Inouee, S.-i. Synthesis and optimization of novel (3*S,*5*R*)-5-(2,2-dimethyl-5-oxo-4-phenylpiperazin-1-yl)piperidine-3-carboxamides as orally active renin inhibitors. *Bio. Med. Chem.* **21**, 5907-5922 (2013).
